# Supplementary material for: The effectiveness of physical activity in asthma management: An overview of systematic reviews
Source: PLoS One. 2025 Jul 3;20(7):e0325488. doi: 10.1371/journal.pone.0325488 (PMC12225870; doi:10.1371/journal.pone.0325488)
Supplement: S1 Appendix — (DOCX) [file pone.0325488.s001.docx]

**Appendix 1: Search Strategy**

**PubMed**

1. Asthma[MeSH Terms]
2. Asthma[Title/Abstract] OR Bronchial Asthma[Title/Abstract]
3. Or/1-2
4. physical activity[MeSH Terms] OR Physical Activit*[Title/Abstract] OR physical training[Title/Abstract] or Physical Therapy[Title/Abstract]
5. Exercise[MeSH Terms] OR exercis*[Title/Abstract] OR aerobic exercise[Title/Abstract] OR exercise therapy[Title/Abstract] OR anaerobic exercise[Title/Abstract]
6. Muscle Stretching Exercise[MeSH Terms] OR muscle stretching exercis*[Title/Abstract]OR Muscle Contraction[MeSH Terms] OR muscle contraction[Title/Abstract]
7. Resistance Training[MeSH Terms] OR resistance training[Title/Abstract]
8. Strength Training[Title/Abstract]
9. weight lifting exercise [Title/Abstract] OR Weight Bearing Exercise Program[Title/Abstract]
10. Endurance Training[MeSH Terms] OR Endurance Training[Title/Abstract]
11. Running[MeSH Terms] OR running[Title/Abstract]
12. Jogging[MeSH Terms] OR jogging[Title/Abstract]
13. Swimming[MeSH Terms] OR swimming[Title/Abstract]
14. Walking[MeSH Terms] OR walking[Title/Abstract] OR Ambulation[Title/Abstract]
15. Qigong[MeSH Terms] OR Qi Gong[Title/Abstract]
16. Ch'i Kung[Title/Abstract] OR Tai-Chi[Title/Abstract]
17. Yoga[MeSH Terms] OR Yoga[Title/Abstract]
18. Neuromuscular Electrical Stimulation[Title/Abstract]
19. Pilates[Title/Abstract]
20. Gymnastics[MeSH Terms] OR Gymnastics[Title/Abstract] OR calisthenics[Title/Abstract]
21. Physiotherapy[Title/Abstract]
22. Or/4-21
23. Meta analysis[Publication Type] OR Systematic Review[Publication Type] OR Literature Review [Publication Type] OR Meta analy*[Title/Abstract] OR systematic review*[Title/Abstract]
24. 3 and 22 and 23

**Embase**

1. 'asthma'/exp OR 'asthma'
2. 'physical activity':ti,ab
3. 'physical training':ti,ab
4. 'physical therapy':ti,ab
5. 'exercise'/exp OR 'exercise'
6. 'exercis*':ti,ab
7. 'exercise therapy':ti,ab
8. 'anaerobic exercise':ti,ab
9. 'aerobic exercise'/exp OR 'aerobic exercise'
10. 'stretching exercise'/exp OR 'stretching exercise'
11. 'muscle stretching exercis*':ti,ab
12. 'muscle contraction'/exp OR 'muscle contraction'
13. 'resistance training'/exp OR 'resistance training'
14. 'strength training':ti,ab
15. 'weight lifting exercis* program':ti,ab
16. 'weight bearing strengthening program':ti,ab
17. 'weight bearing exercise program':ti,ab
18. 'running'/exp OR 'running'
19. 'jogging'/exp OR 'jogging'
20. 'swimming'/exp OR 'swimming'
21. 'qigong'/exp OR 'qigong'
22. 'chi kung':ti,ab
23. 'tai chi':ti,ab
24. 'endurance training'/exp OR 'endurance training'
25. 'yoga'/exp OR 'yoga'
26. 'neuromuscular electrical stimulation':ti,ab
27. 'pilates'/exp OR 'pilates'
28. 'pilates':ti,ab
29. 'physiotherapy':ti,ab
30. 'gymnastics':ti,ab
31. 'calisthenics':ti,ab
32. Or/ 2-31
33. 'systematic review'/exp OR 'systematic review'
34. ''meta analysis'/exp OR 'meta analysis'
35. 33 or 34
36. #1 AND #32 AND #35

**Cochrane library**

1. MeSH descriptor: [Asthma] explode all trees
2. (Asthma):ti,ab,kw OR (Bronchial Asthma):ti,ab,kw
3. 1 or 2
4. MeSH descriptor: [exercise] explode all trees
5. (physical activit*):ti,ab,kw OR (physical training):ti,ab,kw OR (exercise*):ti,ab,kw OR (exercise therapy):ti,ab,kw OR (anaerobic exercise*):ti,ab,kw OR (aerobic exercise*):ti,ab,kw
6. MeSH descriptor: [Muscle Stretching Exercises] explode all trees
7. (Muscle Stretching Exercises):ti,ab,kw
8. MeSH descriptor: [Muscle Contraction] explode all trees
9. (muscle contraction):ti,ab,kw
10. MeSH descriptor: [Resistance Training] explode all trees
11. MeSH descriptor: [endurance training] explode all trees
12. (resistance training):ti,ab,kw OR (Strength Training):ti,ab,kw OR (weight lifting exercise):ti,ab,kw OR (weight bearing strengthening program):ti,ab,kw OR (Weight Bearing Exercise):ti,ab,kw
13. MeSH descriptor: [Running] explode all trees
14. MeSH descriptor: [Jogging] explode all trees
15. MeSH descriptor: [Swimming] explode all trees
16. MeSH descriptor: [Walking] explode all trees
17. (running):ti,ab,kw OR (jogging):ti,ab,kw OR (Swimming):ti,ab,kw OR (Walking):ti,ab,kw OR (Ambulation):ti,ab,kw
18. MeSH descriptor: [Qigong] explode all trees
19. (Qi Gong):ti,ab,kw OR (Ch'i Kung):ti,ab,kw OR (Tai-Chi):ti,ab,kw
20. (Physical Therapy):ti,ab,kw OR (Endurance Training):ti,ab,kw OR (Neuromuscular Electrical Stimulation):ti,ab,kw OR (Physiotherapy):ti,ab,kw
21. MeSH descriptor: [Gymnastics] explode all trees
22. (Gymnastics):ti,ab,kw OR (calisthenics):ti,ab,kw
23. MeSH descriptor: [yoga] explode all trees
24. (yoga):ti,ab,kw
25. Or/4-24
26. 3 AND 25 in Cochranes Reviews

**Web of science**

1. TS=(asthma)
2. TS=("physical activit*")OR TS=("physical training")
3. TS=("exercis*")OR TS=("aerobic exercise*")OR TS=("anaerobic exercise*") OR TS=("exercise therapy")OR TS=("exercise therapy")
4. TS=("muscle stretching exercise*")OR TS=("muscle contraction")OR TS=("resistance training")OR TS=("Strength Training")
5. TS=("weight lifting exercise")OR TS=("weight bearing strengthening")OR TS=("Weight Bearing Exercise")
6. TS=("Running")OR TS=("Jogging")
7. TS=("Swimming")OR TS=("Walking")OR TS=("Ambulation")
8. TS=("Qigong")OR TS=("Ch'i Kung")OR TS=("Tai-Chi")
9. TS=("Endurance Training")
10. TS=("Yoga")
11. TS=("Neuromuscular Electrical Stimulation")
12. TS=("Physical Therapy") OR TS=("Physiotherapy")
13. TS=("Gymnastics")OR TS=("calisthenics") OR TS=("Pilates"))
14. Or/2-13
15. (TS=(meta analy*)) OR TS=(systematic review*)) OR TS=(meta-analysis)) OR TS=(systematic review)
16. 1 and 14 and 15

**CINAHL**

1. SU asthma
2. SU (exercise or physical fitness or physical activity or aerobic exercise or physical exercise) OR SU (anaerobic exercise or anaerobic training or resistance training or strength training or weight training or resistance exercise or strength training or weight lifting or muscle stretching exercises) OR SU (weight bearing exercise or weight bearing) OR SU (qigong or qi gong or chi kung or qigong/taichi) OR SU (yoga or yoga therapy or yoga exercise or pilates or pilates exercise or pilates training) OR SU (neuromuscular electrical stimulation or physical therapy or physiotherapy) OR SU (gymnastics or gymnasts or gymnast or calisthenic)
3. PT (meta-analysis or systematic review or literature review) OR SU (meta-analysis or systematic review or literature review)
4. #1 AND #2 AND #3

**PEDro**

1. Abstract&Title: asthma
2. Therapy: fitness training, respiratory therapy, skill training, strength training,stretching, mobilisation, mannipulation, massage
3. Method: systematic review

**Sportdiscus**

1. SU asthma
2. SU (exercise or physical fitness or physical activity or aerobic exercise or physical exercise) OR SU (anaerobic exercise or anaerobic training or resistance training or strength training or weight training or resistance exercise or strength training or weight lifting or muscle stretching exercises) OR SU (weight bearing exercise or weight bearing) OR SU (qigong or qi gong or chi kung or qigong/taichi) OR SU (yoga or yoga therapy or yoga exercise or pilates or pilates exercise or pilates training) OR SU (neuromuscular electrical stimulation or physical therapy or physiotherapy) OR SU (gymnastics or gymnasts or gymnast or calisthenic)
3. PT (meta-analysis or systematic review or literature review) OR SU (meta-analysis or systematic review or literature review)
4. #1 AND #2 AND #3

**Scopus**

1. TITLE-ABS-KEY ( asthma )
2. ( TITLE-ABS-KEY ( physical AND activit* ) OR TITLE-ABS-KEY ( physical AND training ) OR TITLE-ABS-KEY ( exercise ) OR TITLE-ABS-KEY ( aerobic AND exercise ) OR TITLE-ABS-KEY ( exercise AND therapy ) OR TITLE-ABS-KEY ( anaerobic AND exercise ) OR TITLE-ABS-KEY ( muscle AND stretching AND exercise ) OR TITLE-ABS-KEY ( muscle AND contraction ) OR TITLE-ABS-KEY ( resistance AND training ) OR TITLE-ABS-KEY ( strength AND training ) OR TITLE-ABS-KEY ( weight AND lifting AND exercise ) OR TITLE-ABS-KEY ( weight AND bearing AND strengthening ) OR TITLE-ABS-KEY ( running ) OR TITLE-ABS-KEY ( jogging ) OR TITLE-ABS-KEY ( swimming ) OR TITLE-ABS-KEY ( walking ) OR TITLE-ABS-KEY ( ambulation ) OR TITLE-ABS-KEY ( qigong ) OR TITLE-ABS-KEY ( ch'i AND kung ) OR TITLE-ABS-KEY ( tai-chi ) OR TITLE-ABS-KEY ( endurance AND training ) OR TITLE-ABS-KEY ( yoga ) OR TITLE-ABS-KEY ( neuromuscular AND electrical AND stimulation ) OR TITLE-ABS-KEY ( pilates ) OR TITLE-ABS-KEY ( physical AND therapy ) OR TITLE-ABS-KEY ( physiotherapy ) OR TITLE-ABS-KEY ( gymnastics ) OR TITLE-ABS-KEY ( calisthenics ) )
3. ( TITLE-ABS-KEY ( meta AND analy* ) OR TITLE-ABS-KEY ( systematic AND review ) OR TITLE-ABS-KEY ( literature AND review ) )
4. #1 AND #2 AND #3

**CNKI**

（主题：哮喘）AND （主题：运动 + 运动疗法 + 身体活动 + 跑步 + 瑜伽 + 气功 + 运动训练 + 呼吸训练 + 普拉提 + 物理疗法 + 太极 + 八段锦 + 游泳 + 健身）AND （Meta分析 + 系统评价 + 系统综述）

**Wanfang**

（题名或关键词：哮喘）AND （题名或关键词：运动 + 运动疗法 + 身体活动 + 跑步 + 瑜伽 + 气功 + 运动训练 + 呼吸训练 + 普拉提 + 物理疗法 + 太极 + 八段锦 + 游泳 + 健身） AND （题名或关键词：Meta分析 + 系统评价 + 系统综述）

**维普**

（题名或关键词：哮喘）AND （题名或关键词：运动 + 运动疗法 + 身体活动 + 跑步 + 瑜伽 + 气功 + 运动训练 + 呼吸训练 + 普拉提 + 物理疗法 + 太极 + 八段锦 + 游泳 + 健身） AND （题名或关键词：Meta分析 + 系统评价 + 系统综述）

**CBM**

（题名或关键词：哮喘）AND （题名或关键词：运动 + 运动疗法 + 身体活动 + 跑步 + 瑜伽 + 气功 + 运动训练 + 呼吸训练 + 普拉提 + 物理疗法 + 太极 + 八段锦 + 游泳 + 健身） AND （题名或关键词：Meta分析 + 系统评价 + 系统综述）
