# Supplementary material for: The effectiveness of physical activity in asthma management: An overview of systematic reviews
Source: PLoS One. 2025 Jul 3;20(7):e0325488. doi: 10.1371/journal.pone.0325488 (PMC12225870; doi:10.1371/journal.pone.0325488)
Supplement: S2 Appendix — (DOCX) [file pone.0325488.s002.docx]

**Appendix 2: Studies identified in the literature search**

| **Number** | **Reference** | **Article status** | **Description** |
| --- | --- | --- | --- |
|  | Anshu, Singh N, Deka S, Saraswati P, Sindhwani G, et al. The effect of yoga on pulmonary function in patients with asthma: A meta-analysis. Complementary therapies in clinical practice. 2023;50:101682.<https://doi.org/10.1016/j.ctcp.2022.101682> | Included | Included in the final analysis |
|  | Burgess J, Ekanayake B, Lowe A, Dunt D, Thien F, et al. Systematic review of the effectiveness of breathing retraining in asthma management. Expert Review of Respiratory Medicine. 2011;5(6):789-807.<https://doi.org/10.1586/ers.11.69> | Included | Included in the final analysis |
|  | Chen Y, Fu H. Inspiratory Muscle Training for Asthmatic Patients: A Meta-Analysis of Randomized Controlled Studies. Physikalische Medizin Rehabilitationsmedizin Kurortmedizin. 2022;32(05):285-90.<https://doi.org/10.1055/a-1510-3422> | Included | Included in the final analysis |
|  | Cramer H, Posadzki P, Dobos G, Langhorst J. Yoga for asthma: A systematic review and meta-analysis. Annals of Allergy, Asthma and Immunology. 2014;112(6):503-10.e5.https://doi.org/10.1016/j.anai.2014.03.014 | Included | Included in the final analysis |
|  | Eichenberger P, Diener S, Kofmehl R, Spengler C. Effects of Exercise Training on Airway Hyperreactivity in Asthma: A Systematic Review and Meta-Analysis. Sports Medicine. 2013;43(11):1157-70 | Included | Included in the final analysis |
|  | Feng Z, Wang J, Xie Y, Li J. Effects of exercise-based pulmonary rehabilitation on adults with asthma: a systematic review and meta-analysis. Respiratory Research. 2021;22(1).<https://doi.org/10.1186/s12931-021-01627-w> | Included | Included in the final analysis |
|  | Freitas DA, Holloway EA, Bruno SS, Chaves GSS, Fregonezi GAF, et al. Breathing exercises for adults with asthma. Cochrane Database of Systematic Reviews. 2013;2013(10).<https://doi.org/10.1002/14651858.CD001277.pub3> | Included | Included in the final analysis |
|  | Holloway EA, Ram FSF. Breathing exercises for asthma. Cochrane Database of Systematic Reviews. 2009(4).<https://doi.org/10.1002/14651858.CD001277.pub2> | Included | Included in the final analysis |
|  | Jing Z, Wang X, Zhang P, Huang J, Jia Y, et al. Effects of physical activity on lung function and quality of life in asthmatic children: An updated systematic review and meta-analysis. Frontiers in Pediatrics. 2023;11.<https://doi.org/10.3389/fped.2023.1074429> | Included | Included in the final analysis |
|  | Li X, Mao C, Pan Y. Effect of Routine Therapy Assisted by Physical Exercise on Pulmonary Function in Patients with Asthma in Stable Stage: A Systematic Review and Meta-analysis of Randomized Clinical Trials. Computational and Mathematical Methods in Medicine. 2022;2022.<https://doi.org/10.1155/2022/2350297> | Included | Included in the final analysis |
|  | Lista-Paz A, Bouza Cousillas L, Jácome C, Fregonezi G, Labata-Lezaun N, et al. Effect of respiratory muscle training in asthma: A systematic review and meta-analysis. Annals of Physical and Rehabilitation Medicine. 2023;66(3).<https://doi.org/10.1016/j.rehab.2022.101691> | Included | Included in the final analysis |
|  | Liu Y, Zhao Y, Liu F, Liu L. Effects of Physical Exercises on Pulmonary Rehabilitation, Exercise Capacity, and Quality of Life in Children with Asthma: A Meta-Analysis. Evidence-based Complementary and Alternative Medicine. 2021;2021.<https://doi.org/10.1155/2021/5104102> | Included | Included in the final analysis |
|  | Ma Q, Lu M, Yang Q, Gong F, Zhou L, et al. Effects of aerobic exercise-based pulmonary rehabilitation on quality of life in pediatric asthma: A systematic review and meta-analysis. Heart & lung : the journal of critical care. 2025;69:11-30.<https://doi.org/10.1016/j.hrtlng.2024.09.005> | Included | Included in the final analysis |
|  | McLoughlin RF, Clark VL, Urroz PD, Gibson PG, McDonald VM. Increasing physical activity in severe asthma: a systematic review and meta-analysis. European Respiratory Journal. 2022;60(6).<https://doi.org/10.1183/13993003.00546-2022> | Included | Included in the final analysis |
|  | Ram FS, Robinson SM, Black PN. Effects of physical training in asthma: a systematic review [with consumer summary]. British Journal of Sports Medicine 2000 Jun;34(3):162-167. 2000 | Included | Included in the final analysis |
|  | Ram FSF, Holloway EA, Jones PW. Breathing retraining for asthma. Respiratory Medicine. 2003;97(5):501-7.<https://doi.org/10.1053/rmed.2002.1472> | Included | Included in the final analysis |
|  | Santino TA, Chaves GS, Freitas DA, Fregonezi GA, Mendonça KM. Breathing exercises for adults with asthma. The Cochrane database of systematic reviews. 2020;3(3):Cd001277.<https://doi.org/10.1002/14651858.CD001277.pub4> | Included | Included in the final analysis |
|  | Shi S, Huang H, Zheng R, Zhang N, Dai W, et al. Can long-term regular physical activity improve health outcomes among adults with asthma—evidence from a systematic review and Bayesian meta-analysis. Annals of Translational Medicine. 2022;10(6).<https://doi.org/10.21037/atm-22-1170> | Included | Included in the final analysis |
|  | Wang Q, Yang F, Gao L, Gao W. Effects of Inspiratory Muscle Training and High-Intensity Interval Training on Lung Function and Respiratory Muscle Function in Asthma. Respiratory Care. 2022;67(11):1465-75.<https://doi.org/10.4187/respcare.09813> | Included | Included in the final analysis |
|  | Wu X, Gao S, Lian Y. Effects of continuous aerobic exercise on lung function and quality of life with asthma: A systematic review and meta-analysis. Journal of Thoracic Disease. 2020;12(9):4781-95.<https://doi.org/10.21037/jtd-19-2813> | Included | Included in the final analysis |
|  | Xiang Y, Luo T, Chen X, Zhang H, Zeng L. Effect of inspiratory muscle training in children with asthma: a systematic review and meta-analysis of randomized controlled trials. Frontiers in Pediatrics. 2024;12.<https://doi.org/10.3389/fped.2024.1367710> | Included | Included in the final analysis |
|  | Yin G, Xie Z, Wu P, Zeng Q, Xu C, et al. Appropriate physical training helps to relieve clinical symptoms of pediatric asthma: A meta-analysis. International Journal of Clinical and Experimental Medicine. 2019;12(3):2079-87 | Included | Included in the final analysis |
|  | Zhou L, Xu H. Feasibility of exercise therapy for children with asthma: a meta-analysis. Frontiers in Cell and Developmental Biology. 2023;11.<https://doi.org/10.3389/fcell.2023.1192929> | Included | Included in the final analysis |
|  | Zhu Q, Zhu J, Wang X, Xu Q. A Meta Analysis of Physical Exercise on Improving Lung Function and Quality of Life Among Asthma Patients. Journal of Asthma and Allergy. 2022;15:939-55.<https://doi.org/10.2147/JAA.S369811> | Included | Included in the final analysis |
|  | Liu Fang, LIU Yiran, LIU Lin. A systematic evaluation of the effect of exercise rehabilitation training on exercise ability and quality of life in children with bronchial asthma. %J Chinese Journal of Contemporary Pediatrics. 2021; 23 (10) : 1050-7.https://doi.org/10.7499/j.issn.1008-8830.2104124 | Included | Included in the final analysis |
|  | Yang Shuhui, LIU Yulin, WANG Qian, WANG Lijingzi, CHE Xiaoyan, et al. Meta-analysis of the effects of exercise rehabilitation on lung function, aerobic capacity and quality of life in children with bronchial asthma. 2022; 28(21% J Chinese Journal of Modern Nursing) | Included | Included in the final analysis |
|  | Ram FS, Robinson SM, Black PN, Picot J. Physical training for asthma. Cochrane database of systematic reviews (Online). 2005(4):CD001116 | Included | Included in the final analysis |
|  | Yi Mo, JIA Yuanmin, ZHAO Baosheng, WANG Haixia, LI Yizhang, et al. Meta-analysis of the effects of threshold pressure load inspiratory muscle training on pulmonary rehabilitation and quality of life in asthmatic patients. 2021; 56(7% J Chinese Journal of Nursing) | Included | Included in the final analysis |
|  | You Huangjun, Liu Fei, Sun Weiming, Chen Zhaojun, Shuai Lang. A meta-analysis of the effects of inspiratory muscle training on lung function in patients with bronchial asthma. 2024; 39(2% J Chinese rehabilitation) | Included | Included in the final analysis |
|  | Carson KV, Chandratilleke MG, Picot J, Brinn MP, Esterman AJ, et al. Physical training for asthma (Cochrane review) [with consumer summary]. Cochrane Database of Systematic Reviews 2013;Issue 9. 2013 | Included | Included in the final analysis |
|  | Yang Z-Y, Zhong H-B, Mao C, Yuan J-Q, Huang Y-F, et al. Yoga for asthma. Cochrane Database of Systematic Reviews. 2016(4).<https://doi.org/10.1002/14651858.CD010346.pub2> | Included | Included in the final analysis |
|  | Angelo Deus F, Castro C, Oliveira VC, Figueiredo PHS, Costa HS, Xavier DM, et al. Aquatic exercise for people with asthma: a systematic review with meta-analysis of randomized controlled trials. The Journal of asthma : official journal of the Association for the Care of Asthma. 2024;61(8):780-92.<https://doi.org/10.1080/02770903.2024.2303776> | Included | Included in the final analysis |
|  | Osadnik CR, Gleeson C, McDonald VM, Holland AE. Pulmonary rehabilitation versus usual care for adults with asthma. The Cochrane database of systematic reviews. 2022;8(8):Cd013485.<https://doi.org/10.1002/14651858.CD013485.pub2> | Included | Included in the final analysis |
|  | Silva IS, Fregonezi GAF, Dias FAL, Ribeiro CTD, Guerra RO, Ferreira GMH. Inspiratory muscle training for asthma. Cochrane Database of Systematic Reviews. 2013;2013(9).<https://doi.org/10.1002/14651858.CD003792.pub2> | Included | Included in the final analysis |
|  | Breathing exercises for adults with asthma. Drug and Therapeutics Bulletin. 2015;53(11):126-9.https://doi.org/10.1136/dtb.2015.11.0361 | Excluded | Not a systematic review |
|  | Andersson H, Fredrikson A, Lindström I. Physical exercise for patients with asthma -- a systematic review. Nordisk Fysioterapi. 2005;9(2):82-9 | Excluded | Target outcome indicators were not reported |
|  | Andrade C, Martins R, Carvalho N. RESPIRATORY REHABILITATION IN CHILDREN WITH ASTHMA: SYSTEMATIC REVIEW. Millenium: Journal of Education, Technologies, and Health. 2023;2023(12).<https://doi.org/10.29352/mill0212e.30090> | Excluded | Without quantitative synthesis |
|  | Beggs S, Foong YC, Le HC, Noor D, Wood-Baker R, et al. Swimming training for asthma in children and adolescents aged 18 years and under. Paediatric Respiratory Reviews. 2013;14(2):96-7.<https://doi.org/10.1016/j.prrv.2013.03.002> | Excluded | Full text not available |
|  | Birdee GS, Yeh GY, Wayne PM, Phillips RS, Davis RB, et al. Clinical Applications of Yoga for the Pediatric Population: A Systematic Review. Academic Pediatrics. 2009;9(4):212-20.<https://doi.org/10.1016/j.acap.2009.04.002> | Excluded | Without quantitative synthesis |
|  | Ang J, Moussa R, Shaikh S, Mele S. Effects of aerobic exercise on asthma control and quality of life in adults: a systematic review. Journal of Asthma. 2023;60(5):845-55.<https://doi.org/10.1080/02770903.2022.2103429> | Excluded | Without quantitative synthesis |
|  | rinn M, Carson K, Chandratilleke M, Picot J, Esterman A, et al. Physical training for asthma: A cochrane systematic review. Respirology. 2014;19:41.<https://doi.org/10.1111/resp.12262> | Excluded | Conference Abstract without full text |
|  | Cabradilla JM, Balbuena JML, Quiambao JL, Siasoco MBR. Efficacy of yoga in improving clinical outcomes among patients with asthma: A meta-analysis. Respirology. 2011;16:199.https://doi.org/10.1111/j.1400-1843.2011.02071.x | Excluded | Conference Abstract without full text |
|  | Carson KV, Chandratilleke MG, Picot J, Brinn MP, Esterman AJA, et al. Physical training for asthma. Sao Paulo Medical Journal. 2014;132(3):193-4.<https://doi.org/10.1590/1516-3180.20141323t1> | Excluded | Full text not available |
|  | Cassim R, Dharmage SC, Koplin JJ, Milanzi E, Paro FM, et al. Does physical activity strengthen lungs and protect against asthma in childhood? A systematic review. Pediatric Allergy and Immunology. 2019;30(7):739-51.<https://doi.org/10.1111/pai.13105> | Excluded | Non-rct studies were included |
|  | Castilho T, Itaborahy BDH, Hoepers A, de Brito JN, da S. Almeida AC, et al. Effects of inspiratory muscle training and breathing exercises in children with asthma: A systematic review. Journal of Human Growth and Development. 2020;30(2):291-300.<https://doi.org/10.7322/JHGD.V30.10381> | Excluded | Contra to protocol: not a systematic review |
|  | Chandratilleke MG, Carson KV, Picot J, Brinn M, Smith BJ. Physical training for asthma a meta-analysis (Cochrane review update). Respirology. 2011;16:12.<https://doi.org/10.1111/j.1440-1843.2011.01936.x> | Excluded | Without quantitative synthesis |
|  | Chaves G, Macêdo T, Freitas D, Britto R, Mendonc¸a K. Breathing exercises for children with asthma. European Respiratory Journal. 2015;46.<https://doi.org/10.1183/13993003.congress2015.PA965> | Excluded | Conference Abstract without full text |
|  | Cicutto L. Review: physical training increases cardiopulmonary fitness in asthma and does not decrease lung function. Evidence Based Nursing. 2006;9(2):44 | Excluded | Not a systematic review |
|  | Cordova-Rivera L, Gibson PG, Gardiner PA, McDonald VM. A Systematic Review of Associations of Physical Activity and Sedentary Time with Asthma Outcomes. Journal of Allergy and Clinical Immunology: In Practice. 2018;6(6):1968-81.e2.https://doi.org/10.1016/j.jaip.2018.02.027 | Excluded | Target outcome indicators were not reported |
|  | Crosbie A. The effect of physical training in children with asthma on pulmonary function, aerobic capacity and health-related quality of life: a systematic review of randomized control trials. Pediatric exercise science. 2012;24(3):472-89 | Excluded | Without quantitative synthesis |
|  | da Silva RA. People with asthma: care during the COVID-19 pandemic and the importance of regular exercise for the immune system. Motriz Revista de Educacao Fisica. 2022;28.<https://doi.org/10.1590/S1980-657420220021121> | Excluded | Target outcome indicators were not reported |
|  | Das RR, Sankar J, Kabra SK. Role of breathing exercises and yoga/pranayama in childhood asthma: A systematic review. Current Pediatric Reviews. 2019;15(3):175-83.<https://doi.org/10.2174/1573396315666190121122452> | Excluded | Without quantitative synthesis |
|  | de Oliveira Francisco C, Bhatawadekar SA, Babineau J, Reid WD, Yadollahi A. Effects of physical exercise training on nocturnal symptoms in asthma: systematic review. PLoS ONE 2018 Oct;13(10):e0204953. 2018 | Excluded | Target outcome indicators were not reported |
|  | Dowell S, Vining I, Berry B, Young C, Lauderdale M, et al. EFFECTS OF BREATHING EXERCISES ON QUALITY OF LIFE IN ASTHMA PATIENTS: A SYSTEMATIC REVIEW. Biomedical Sciences Instrumentation. 2021;57(4):441-5.<https://doi.org/10.34107/KSZV7781.10439> | Excluded | Conference Abstract without full text |
|  | Eijkemans M, Mommers M, Draaisma JM, Thijs C, Prins MH. Physical activity and asthma: a systematic review and meta-analysis. PLoS One. 2012;7(12):e50775.<https://doi.org/10.1371/journal.pone.0050775> | Excluded | Non-rct studies were included |
|  | El-Khuffash MO, Devlin C, Hickey R. Physical therapy and lung function in children with asthma: is it helpful? Pediatric Research. 2021;89(6):1580-1.<https://doi.org/10.1038/s41390-021-01462-1> | Excluded | Not a systematic review |
|  | Francisco CDO, Bhatawadekar SA, Babineau J, Darlene Reid W, Yadollahi A. Effects of physical exercise training on nocturnal symptoms in asthma: Systematic review. PLoS ONE. 2018;13(10).<https://doi.org/10.1371/journal.pone.0204953> | Excluded | Target outcome indicators were not reported |
|  | Galantino ML, Galbavy R, Quinn L. Therapeutic effects of yoga for children: a systematic review of the literature. Pediatric physical therapy : the official publication of the Section on Pediatrics of the American Physical Therapy Association. 2008;20(1):66-80.<https://doi.org/10.1097/PEP.0b013e31815f1208> | Excluded | Non-rct studies were included |
|  | Godoy Fernandes AL. Yoga for asthma. Sao Paulo Medical Journal. 2016;134(4):368-.<https://doi.org/10.1590/1516-3180.20161344t2> | Excluded | Conference Abstract without full text |
|  | Gómez DG, Barbosa FÁ. Effects of physical exercise on aerobic capacity and quality of life in patients diagnosed with asthma: A systematic review and meta-analysis. Archivos de Medicina del Deporte. 2022;39(6):342-52.<https://doi.org/10.18176/archmeddeporte.00113> | Excluded | Unable to obtain sufficient information |
|  | Goodman M, Hays S. Asthma and swimming: A meta-analysis. Journal of Asthma. 2008;45(8):639-47.<https://doi.org/10.1080/02770900802165980> | Excluded | Target outcome indicators were not reported |
|  | Hansen ESH, Pitzner-Fabricius A, Toennesen LL, Rasmusen HK, Hostrup M, et al. Effect of aerobic exercise training on asthma in adults -- a systematic review and meta-analysis. The European Respiratory Journal 2020 Jul;56(1):2000146. 2020 | Excluded | Target outcome indicators were not reported |
|  | Harper V, Trayer J. Breathing exercises for adults with asthma. Clinical and Experimental Allergy. 2022;52(6):732-4.<https://doi.org/10.1111/cea.14141> | Excluded | Duplicate publication |
|  | Heikkinen SA, Quansah R, Jaakkola JJ, Jaakkola MS. Effects of regular exercise on adult asthma. Eur J Epidemiol. 2012;27(6):397-407.<https://doi.org/10.1007/s10654-012-9684-8> | Excluded | Non-rct studies were included |
|  | Heikkinen SAM, Mäkikyrö EMS, Hugg TT, Jaakkola MS, Jaakkola JJK. Effects of regular exercise on asthma control in young adults. Journal of Asthma. 2018;55(7):726-33.<https://doi.org/10.1080/02770903.2017.1366510> | Excluded | There has been an updated systematic review |
|  | Holloway E, Ram FS. Breathing exercises for asthma. The Cochrane database of systematic reviews. 2000(3):Cd001277.<https://doi.org/10.1002/14651858.Cd001277> | Excluded | There has been an updated systematic review |
|  | Holloway E, Ram FS. Breathing exercises for asthma. The Cochrane database of systematic reviews. 2004(1):Cd001277.<https://doi.org/10.1002/14651858.CD001277.pub2> | Excluded | There has been an updated systematic review |
|  | Jiang J, Zhang D, Huang Y, Wu Z, Zhang W. Exercise rehabilitation in pediatric asthma: A systematic review and network meta-analysis. Pediatric Pulmonology. 2022;57(12):2915-27.<https://doi.org/10.1002/ppul.26134> | Excluded | Network meta-analysis |
|  | Kadam S, Prem V, Ughreja RA. Effect of breathing re-training on end-tidal carbon-di-oxide in patients with asthma: a systematic review and meta-analysis. Journal of Asthma. 2023;60(8):1493-502.<https://doi.org/10.1080/02770903.2022.2162413> | Excluded | Without quantitative synthesis |
|  | Kuder MM, Clark M, Cooley C, Prieto-Centurion V, Danley A, et al. A Systematic Review of the Effect of Physical Activity on Asthma Outcomes. Journal of Allergy and Clinical Immunology: In Practice. 2021;9(9):3407-21.e8.https://doi.org/10.1016/j.jaip.2021.04.048 | Excluded | Without quantitative synthesis |
|  | Lack S, Brown R, Kinser PA. An Integrative Review of Yoga and Mindfulness-Based Approaches for Children and Adolescents with Asthma. Journal of Pediatric Nursing. 2020;52:76-81.<https://doi.org/10.1016/j.pedn.2020.03.006> | Excluded | Without quantitative synthesis |
|  | Lin N, Huang Z, Li J, Dong J, Yan X, et al. Effects of high-intensity interval training on exercise capacity and asthma-related outcomes in children: A systematic review. Allergy: European Journal of Allergy and Clinical Immunology. 2024.<https://doi.org/10.1111/all.16336> | Excluded | Full text not available |
|  | Macedo TMF, Freitas DA, Chaves GSS, Holloway EA, Mendonca K. Breathing exercises for children with asthma (Cochrane review) [with consumer summary]. Cochrane Database of Systematic Reviews 2016;Issue 4. 2016 | Excluded | Target outcome indicators were not reported |
|  | Mendonc¸a K, Freitas D, Holloway E, Chaves G, Bruno S, et al. The effects of breathing exercises in adults with asthma: A systematic review. European Respiratory Journal. 2013;42 | Excluded | Conference Abstract without full text |
|  | Molina-Gómez P, Girón-Damas C, López-Méndez N, Osuna-Pérez MC. Evidence of therapeutic exercise and alternative therapies versus respiratory exercises in children with asthma: Systematic review. Cuestiones de Fisioterapia. 2021;50(2):150-62 | Excluded | Without quantitative synthesis |
|  | Pacheco DRR, Silva MJB, Alexandrino AMS, Torres RMT. Exercise-related quality of life in subjects with asthma: A systematic review. Journal of Asthma. 2012;49(5):487-95.<https://doi.org/10.3109/02770903.2012.680636> | Excluded | Without quantitative synthesis |
|  | Pakhale S, Luks V, Burkett A, Turner L. Effect of physical training on airway inflammation in bronchial asthma: A systematic review. BMC Pulmonary Medicine. 2013;13(1).<https://doi.org/10.1186/1471-2466-13-38> | Excluded | Target outcome indicators were not reported |
|  | Posadzki P, Ernst E. Yoga for asthma? A systematic review of randomized clinical trials. Journal of Asthma. 2011;48(6):632-9.<https://doi.org/10.3109/02770903.2011.584358> | Excluded | Non-rct studies were included |
|  | Ramachandran HJ, Jiang Y, Shan CH, San Tam WW, Wang W. A systematic review and meta-analysis on the effectiveness of swimming on lung function and asthma control in children with asthma. International Journal of Nursing Studies. 2021;120.<https://doi.org/10.1016/j.ijnurstu.2021.103953> | Excluded | Without quantitative synthesis |
|  | Ritz T, Roth WT. Behavioral interventions in asthma - Breathing training. Behavior Modification. 2003;27(5):710-30.<https://doi.org/10.1177/0145445503256323> | Excluded | Without quantitative synthesis |
|  | Rosimini C. Benefits of swim training for children and adolescents with asthma. Journal of the American Academy of Nurse Practitioners. 2003;15(6):247-52.<https://doi.org/10.1111/j.1745-7599.2003.tb00394.x> | Excluded | Not a systematic review |
|  | Sánchez-Lozano J, Martínez-Pizarro S. Efficacy of yoga as an adjuvant therapy in patients with asthma. Bibliographic review. Revista de Patologia Respiratoria. 2024;27(2):72-9.<https://doi.org/10.24875/RPR.24000008> | Excluded | Without quantitative synthesis |
|  | Santino TA, Chaves GSS, Freitas DA, Fregonezi GAF, Mendonca KPP. Breathing Exercises for Adults with Asthma: An Updated Cochrane Systematic Review. American Journal of Respiratory and Critical Care Medicine. 2021;203(9).<https://doi.org/10.1164/ajrccm-conference.2021.TP102> | Excluded | Conference Abstract without full text |
|  | Santos JD. The Impact of Tai Chi Chuan on Childhood Asthma: A Systematic Review. Global Advances in Health and Medicine. 2022;11:123.<https://doi.org/10.1177/2164957X221096590> | Excluded | Conference Abstract without full text |
|  | Sharma M, Haider T, Bose PP. Yoga as an alternative and complementary treatment for asthma: a systematic review. Journal of Evidence-Based Complementary & Alternative Medicine 2012 Oct;17(3):212-217. 2012 | Excluded | Non-rct studies were included |
|  | Silva I, Fregonezi G, Dias F, Ribeiro C, Guerra R, et al. Systematic review of inspiratory muscle training for asthma. European Respiratory Journal. 2013;42 | Excluded | Without quantitative synthesis |
|  | Singh N, Deka S, Saraswati P, Sindhwani G, Goel A, et al. The effect of yoga on pulmonary function in patients with asthma: A meta-analysis. Complementary Therapies in Clinical Practice. 2023;50.<https://doi.org/10.1016/j.ctcp.2022.101682> | Excluded | Duplicate publication |
|  | Suárez-Pazos E, Justo-Cousiño LA, Alonso-Calvete A, González-González Y, Da Cuña-Carrera I. Effects of therapeutic exercise in minor asmathic patients. A systematic review. Cuestiones de Fisioterapia. 2023;52(3):256-71 | Excluded | Without quantitative synthesis |
|  | Thomas M, Bruton A. Breathing exercises for asthma. Breathe. 2014;10(4):312-22.<https://doi.org/10.1183/20734735.008414> | Excluded | Not a systematic review |
|  | Valkenborghs S, Anderson S, Scott H, Callister R. The characteristics and effects of exercise interventions on improving physical fitness in adults with asthma: a systematic review and meta-analysis. Journal of Science and Medicine in Sport. 2021;24:S71.<https://doi.org/10.1016/j.jsams.2021.09.175> | Excluded | Conference Abstract without full text |
|  | Valkenborghs SR, Anderson SL, Scott HA, Callister R. Exercise Training Programs Improve Cardiorespiratory and Functional Fitness in Adults With Asthma: A SYSTEMATIC REVIEW AND META-ANALYSIS. Journal of Cardiopulmonary Rehabilitation and Prevention. 2022;42(6):423-33.<https://doi.org/10.1097/HCR.0000000000000698> | Excluded | Non-rct studies were included |
|  | Walters J, Foong YC, Le HCT, Wan D, Wood-Baker R, et al. Swimming training benefits children and adolescents with asthma: Results of a systematic review. Respirology. 2013;18:14.<https://doi.org/10.1111/resp.12045> | Excluded | Conference Abstract without full text |
|  | Wanrooij VH, Willeboordse M, Dompeling E, van de Kant KD. Exercise training in children with asthma: a systematic review. British journal of sports medicine. 2014;48(13):1024-31.<https://doi.org/10.1136/bjsports-2012-091347> | Excluded | Without quantitative synthesis |
|  | Wanrooij VHM, Willeboordse M, Dompeling E, van de Kant KDG. Exercise training in children with asthma: a systematic review [with consumer summary]. British Journal of Sports Medicine 2013 Jul;48(13):1024-1031. 2013 | Excluded | Without quantitative synthesis |
|  | Wise J. Yoga may improve asthma symptoms, Cochrane review finds. BMJ (Clinical research ed). 2016;353:i2462.<https://doi.org/10.1136/bmj.i2462> | Excluded | Conference Abstract without full text |
|  | Yassaee A, Pike K, Hart C, Manikam L, Hamlyn-Williams C, et al. Barriers and facilitators to physical activity for children and young people with asthma: An evidence synthesis. Archives of Disease in Childhood. 2017;102:A172.<https://doi.org/10.1136/archdischild-2017-313087.430> | Excluded | Conference Abstract without full text |
|  | Zafar SN, Khalid M, Jamil A, Butt SK, Manzoor A, et al. The role of breathing techniques in the management of asthma: a systematic review. The Journal of the Pakistan Medical Association 2024 Jul;74(7):1296-1299. 2024 | Excluded | Without quantitative synthesis |
|  | Zhang W, Wang Q, Liu L, Yang W, Liu H. Effects of physical therapy on lung function in children with asthma: a systematic review and meta-analysis. Pediatric Research. 2021;89(6):1343-51.<https://doi.org/10.1038/s41390-020-0874-x> | Excluded | Target outcome indicators were not reported |
|  | Ernst E. Breathing techniques - adjunctive treatment modalities for asthma? A systematic review. European Respiratory Journal. 2000;15(5):969-72.<https://doi.org/10.1183/09031936.00.15596900> | Excluded | Without quantitative synthesis |
|  | Ertürk G, Günday Ç, Evrendilek H, Sağır K, Aslan GK. Effects of high intensity interval training and sprint interval training in patients with asthma: a systematic review. Journal of Asthma. 2022;59(11):2292-304.<https://doi.org/10.1080/02770903.2021.1999470> | Excluded | Without quantitative synthesis |
|  | Grande AJ, Silva V, Andriolo BNG, Riera R, Parra SA, et al. Water-based exercise for adults with asthma. Cochrane Database of Systematic Reviews. 2014(7).<https://doi.org/10.1002/14651858.CD010456.pub2> | Excluded | Conference Abstract without full text |
|  | Prem V, Sahoo RC, Adhikari P. Effect of diaphragmatic breathing exercise on quality of life in subjects with asthma: A systematic review. Physiotherapy Theory &amp; Practice. 2013;29(4):271-7.<https://doi.org/10.3109/09593985.2012.731626> | Excluded | Without quantitative synthesis |
|  | Bruurs MLJ, van der Giessen LJ, Moed H. The effectiveness of physiotherapy in patients with asthma: A systematic review of the literature. Respiratory Medicine. 2013;107(4):483-94.<https://doi.org/10.1016/j.rmed.2012.12.017> | Excluded | Not a systematic review |
|  | Lochte L, Nielsen KG, Petersen PE, Platts-Mills TAE. Childhood asthma and physical activity: A systematic review with meta-analysis and graphic appraisal tool for epidemiology assessment. BMC Pediatrics. 2016;16(1).<https://doi.org/10.1186/s12887-016-0571-4> | Excluded | Non-rct studies were included |
|  | Macêdo TMF, Freitas DA, Chaves GSS, Holloway EA, Mendonça KM. Breathing exercises for children with asthma. Cochrane Database of Systematic Reviews. 2014;2014(3).<https://doi.org/10.1002/14651858.CD011017> | Excluded | Not a systematic review |
|  | Dennis J. Alexander technique for chronic asthma. The Cochrane database of systematic reviews. 2000(2):Cd000995.<https://doi.org/10.1002/14651858.Cd000995> | Excluded | No original studies were included |
|  | Dennis JA, Cates CJ. Alexander technique for chronic asthma. Cochrane database of systematic reviews (Online). 2012;9:CD000995 | Excluded | No original studies were included |
|  | Liu Jiaming, Sun Xiaorong, Wen Jian, HE Xiaoli, Ren Maoling. A meta-analysis of the effects of aerobic exercise on lung function, quality of life and asthma control in adults with asthma J Chinese Journal of Pulmonary Diseases2023;16(4):592-5.https://doi.org/10.3877/cma.j.issn.1674-6902.2023.04.040 | Excluded | Full text not available |
|  | Liu Weizhu. Swimming improves the health status of children with asthma %J British Medical Journal. 2013; 16 (4) | Excluded | Full text not available |
|  | Wang Xingzhi, WU Cheng, ZHANG Juan, Sun Xin. The influence of exercise on asthma and the progress of exercise prescription in children with asthma %J Chinese Journal of Practical Pediatrics. 2021; 36 (09) : 714-20.https://doi.org/10.19538/j.ek2021090617 | Excluded | Not a systematic review |
|  | Wang Zijuan Liu A Liu A Yang A Wei A. A systematic Review of the intervention effect of exercise training on children with bronchial asthma %J Journal of Practical Cardio-cerebro-pulmonary Vascular Diseases 2024; 32 (7) | Excluded | Not a systematic review |
|  | Yang Yiyun, SHANG Xingchen, Chen Rong, SUN Xinxin, LIAO Yuexia. Effects of physical exercise on lung function, aerobic capacity, and quality of life in children with asthma: a meta-analysis of a randomized controlled trial. 2021; 40 (5) : 435-41 | Excluded | There are obvious errors in statistical analysis |
|  | Xing ShuangTaofeng, Zhang Yifan, Wang LAN, YU Guoying. Mesh meta-analysis of the effects of different exercise modes on pulmonary function in children with asthma %J Journal of Henan Normal University (Natural Science Edition). 2023; 51 (2) | Excluded | Network meta-analysis |
|  | Verrastro G. Yoga as therapy: When is it helpful? Journal of Family Practice. 2014;63(9):E1-E6 | Excluded | Lack of relation between the title and the abstract of the article with the subject under study. |
|  | Adams D, Lyszczyk M, Vohra S. Systematic review of the safety of mind-body interventions in children. Journal of Complementary and Integrative Medicine. 2015;12(1):eA12.https://doi.org/10.1515/jcim-2014-6000 | Excluded | Lack of relation between the title and the abstract of the article with the subject under study. |
|  | Bhagel P, Saha M. Effects of yogic intervention on pulmonary function and respiratory muscle strength parameters: A systematic literature review and meta-analysis. Journal of Biosciences. 2021;46(3).https://doi.org/10.1007/s12038-021-00192-0 | Excluded | Lack of relation between the title and the abstract of the article with the subject under study. |
|  | Billany RE, Vadaszy N, Lightfoot CJ, Graham-Brown MP, Smith AC, Wilkinson TJ. Characteristics of effective home-based resistance training in patients with noncommunicable chronic diseases: a systematic scoping review of randomised controlled trials. Journal of Sports Sciences. 2021;39(10):1174-85.https://doi.org/10.1080/02640414.2020.1861741 | Excluded | Lack of relation between the title and the abstract of the article with the subject under study. |
|  | Boehm K, Ostermann T, Milazzo S, Büssing A. Effects of yoga interventions on fatigue: A meta-analysis. Evidence-based Complementary and Alternative Medicine. 2012;2012.https://doi.org/10.1155/2012/124703 | Excluded | Lack of relation between the title and the abstract of the article with the subject under study. |
|  | Burge AT, Gadowski AM, Romero L, Vagheggini G, Spathis A, Smallwood NE, et al. The effect of graded exercise therapy on fatigue in people with serious respiratory illness: a systematic review. European Respiratory Review. 2024;33(174).https://doi.org/10.1183/16000617.0027-2024 | Excluded | Lack of relation between the title and the abstract of the article with the subject under study. |
|  | Büssing A, Michalsen A, Khalsa SBS, Telles S, Sherman KJ. Effects of yoga on mental and physical health: A short summary of reviews. Evidence-based Complementary and Alternative Medicine. 2012;2012.https://doi.org/10.1155/2012/165410 | Excluded | Lack of relation between the title and the abstract of the article with the subject under study. |
|  | Chobisa CA, Lalwani L, Vardhan V, Nathani H. Effect of Respiratory Muscle Training on Improving Respiratory Muscle Strength in Younger Population: A Systematic Review. Journal of Clinical and Diagnostic Research. 2024;18(8):YE01-YE5.https://doi.org/10.7860/JCDR/2024/69640.19783 | Excluded | Lack of relation between the title and the abstract of the article with the subject under study. |
|  | Edouard P, Gautheron V, D'Anjou MC, Pupier L, Devillard X. Training programs for children: literature review. Annales de Readaptation et de Medecine Physique. 2007;50(6):510-9.https://doi.org/10.1016/j.annrmp.2007.04.015 | Excluded | Lack of relation between the title and the abstract of the article with the subject under study. |
|  | Field T. Exercise research on children and adolescents. Complementary Therapies in Clinical Practice. 2012;18(1):54-9.https://doi.org/10.1016/j.ctcp.2011.04.002 | Excluded | Lack of relation between the title and the abstract of the article with the subject under study. |
|  | Gimeno-Santos E, Torres-Castro R, Caicedo-Trujillo S, Gutierrez-Arias R, Alsina-Restoy X, Vasconcello-Castillo L, et al. Effectiveness of inspiratory muscle training in chronic respiratory diseases: an overview of systematic reviews of clinical randomised trials. European Respiratory Journal. 2023;62:PA347.https://doi.org/10.1183/13993003.congress-2023.PA347 | Excluded | Lack of relation between the title and the abstract of the article with the subject under study. |
|  | Goldenberg RB. Singing Lessons for Respiratory Health: A Literature Review. Journal of Voice. 2018;32(1):85-94.https://doi.org/10.1016/j.jvoice.2017.03.021 | Excluded | Lack of relation between the title and the abstract of the article with the subject under study. |
|  | Heredia-Rizo AM, Martinez-Calderon J, Piña-Pozo F, González-García P, García-Muñoz C. Effectiveness of mind–body exercises in chronic respiratory diseases: an overview of systematic reviews with meta-analyses. Disability &amp; Rehabilitation. 2024;46(12):2496-511 | Excluded | Lack of relation between the title and the abstract of the article with the subject under study. |
|  | Joschtel B, Gomersall SR, Tweedy S, Petsky H, Chang AB, Trost SG. Effects of exercise training on physical and psychosocial health in children with chronic respiratory disease: a systematic review and meta-analysis [with consumer summary]. BMJ Open Sport & Exercise Medicine 2018;4(1):e000409. 2018 | Excluded | Lack of relation between the title and the abstract of the article with the subject under study. |
|  | Karmisholt K, Gotzcshe PC. Physical activity for secondary prevention of disease - Systematic reviews of randomised clinical trials. Danish Medical Bulletin. 2005;52(2):90-4 | Excluded | Lack of relation between the title and the abstract of the article with the subject under study. |
|  | Leitl D, Gloeckl R. Overview on pulmonary rehabilitation. Pneumologe. 2022;19(3):130-41.https://doi.org/10.1007/s10405-021-00431-z | Excluded | Lack of relation between the title and the abstract of the article with the subject under study. |
|  | Manifield J, Chaudhry Y, Singh SJ, Ward TJC, Whelan ME, Orme MW. Changes in physical activity, sedentary behaviour and sleep following pulmonary rehabilitation: a systematic review and network meta-analysis. European Respiratory Review. 2024;33(172).https://doi.org/10.1183/16000617.0225-2023 | Excluded | Lack of relation between the title and the abstract of the article with the subject under study. |
|  | McCall MC, Ward A, Roberts NW, Heneghan C. Overview of systematic reviews: Yoga as a therapeutic intervention for adults with acute and chronic health conditions. Evidence-based Complementary and Alternative Medicine. 2013;2013.https://doi.org/10.1155/2013/945895 | Excluded | Lack of relation between the title and the abstract of the article with the subject under study. |
|  | Mishra B, Agarwal A, George JA, Upadhyay AD, Nilima N, Mishra R, et al. Effectiveness of Yoga in Modulating Markers of Immunity and Inflammation: A Systematic Review and Meta-Analysis. Cureus. 2024;16(4):e57541.https://doi.org/10.7759/cureus.57541 | Excluded | Lack of relation between the title and the abstract of the article with the subject under study. |
|  | Niranjan V, Tarantino G, Kumar J, Stokes D, O'Connor R, O'Regan A. The Impact of Dance Interventions on Patients with Noninfectious Pulmonary Diseases: A Systematic Review. Int J Environ Res Public Health. 2022;19(17).https://doi.org/10.3390/ijerph191711115 | Excluded | Lack of relation between the title and the abstract of the article with the subject under study. |
|  | Ochmann U, Jorres RA, Nowak D. Long-term efficacy of pulmonary rehabilitation: a state-of-the-art review. Journal of Cardiopulmonary Rehabilitation and Prevention 2012 May-Jun;32(3):117-126. 2012 | Excluded | Lack of relation between the title and the abstract of the article with the subject under study. |
|  | Oja P, Memon AR, Titze S, Jurakic D, Chen ST, Shrestha N, et al. Health Benefits of Different Sports: a Systematic Review and Meta-Analysis of Longitudinal and Intervention Studies Including 2.6 Million Adult Participants. Sports Medicine - Open. 2024;10(1).https://doi.org/10.1186/s40798-024-00692-x | Excluded | Lack of relation between the title and the abstract of the article with the subject under study. |
|  | Onu I, Iordan DA, Codreanu CM, Matei D, Galaction AI. Anti-inflammatory effects of exercise training. A systematic review. Balneo and PRM Research Journal. 2021;12(4):418-25.https://doi.org/10.12680/balneo.2021.473 | Excluded | Lack of relation between the title and the abstract of the article with the subject under study. |
|  | Pedersen BK, Saltin B. Evidence for prescribing exercise as therapy in chronic disease. Scandinavian Journal of Medicine & Science in Sports 2006 Feb;1(16 Suppl):3-63. 2006 | Excluded | Lack of relation between the title and the abstract of the article with the subject under study. |
|  | Pedersen BK, Saltin B. Exercise as medicine - Evidence for prescribing exercise as therapy in 26 different chronic diseases. Scandinavian Journal of Medicine and Science in Sports. 2015;25:1-72.https://doi.org/10.1111/sms.12581 | Excluded | Lack of relation between the title and the abstract of the article with the subject under study. |
|  | Pérez-Gisbert L, Torres-Sánchez I, Ortiz-Rubio A, Calvache-Mateo A, López-López L, Cabrera-Martos I, et al. Effects of the COVID-19 pandemic on physical activity in chronic diseases: A systematic review and meta-analysis. International Journal of Environmental Research and Public Health. 2021;18(23).https://doi.org/10.3390/ijerph182312278 | Excluded | Lack of relation between the title and the abstract of the article with the subject under study. |
|  | Petrescu S, Pitigoi G, Pǎunescu C, Pǎunescu M. Physical activity and pulmonary diseases. A systematic review. Revista de Cercetare si Interventie Sociala. 2014;45:132-43 | Excluded | Lack of relation between the title and the abstract of the article with the subject under study. |
|  | Plante WA, Lobato D, Engel R. Review of group interventions for pediatric chronic conditions. Journal of Pediatric Psychology. 2001;26(7):435-53.https://doi.org/10.1093/jpepsy/26.7.435 | Excluded | Lack of relation between the title and the abstract of the article with the subject under study. |
|  | Poureslami I, Shum J, Boulet L, Poirier C, Goldstein RS, Gupta SK, et al. Involvement of patients and professionals in the development of a conceptual framework on functional health literacy to improve chronic airways disease outcomes. American Journal of Respiratory and Critical Care Medicine. 2019;199(9) | Excluded | Lack of relation between the title and the abstract of the article with the subject under study. |
|  | Raub JA. Psychophysiologic effects of Hatha Yoga on musculoskeletal and cardiopulmonary function: a literature review. Journal of Alternative &amp; Complementary Medicine. 2002;8(6):797-812.https://doi.org/10.1089/10755530260511810 | Excluded | Lack of relation between the title and the abstract of the article with the subject under study. |
|  | Reilly C, Sails J, Birch R, Clifton I, Peckham D, McKenna J, et al. Physical activity promotion interventions in chronic airways disease: preliminary findings from a scoping review and meta-analysis. European Respiratory Journal. 2022;60.https://doi.org/10.1183/13993003.congress-2022.1015 | Excluded | Lack of relation between the title and the abstract of the article with the subject under study. |
|  | Rosenberg CE, Khoury P. Approach to Eosinophilia Presenting With Pulmonary Symptoms. Chest. 2021;159(2):507-16.https://doi.org/10.1016/j.chest.2020.09.247 | Excluded | Lack of relation between the title and the abstract of the article with the subject under study. |
|  | Salcedo PA, Lindheimer JB, Klein-Adams JC, Sotolongo AM, Falvo MJ. Effects of Exercise Training on Pulmonary Function in Adults With Chronic Lung Disease: A Meta-Analysis of Randomized Controlled Trials. Archives of Physical Medicine and Rehabilitation. 2018;99(12):2561-9.https://doi.org/10.1016/j.apmr.2018.03.014 | Excluded | Lack of relation between the title and the abstract of the article with the subject under study. |
|  | Santino TA, Chaves GS, Mendonça KM. Letter to the editor: The effect of yoga on pulmonary function in patients with asthma: A meta-analysis. Complementary therapies in clinical practice. 2023;52:101778.https://doi.org/10.1016/j.ctcp.2023.101778 | Excluded | Lack of relation between the title and the abstract of the article with the subject under study. |
|  | Shahin W, Stupans I, Kennedy G. Health beliefs and chronic illnesses of refugees: a systematic review. Ethnicity &amp; Health. 2021;26(5):756-68.https://doi.org/10.1080/13557858.2018.1557118 | Excluded | Lack of relation between the title and the abstract of the article with the subject under study. |
|  | Sheng N, Ma J, Ding W, Zhang Y. Effects of caregiver-involved interventions on the quality of life of children and adolescents with chronic conditions and their caregivers: a systematic review and meta-analysis. Quality of Life Research. 2019;28(1):13-33.https://doi.org/10.1007/s11136-018-1976-3 | Excluded | Lack of relation between the title and the abstract of the article with the subject under study. |
|  | Smidt N, de Vet HC, Bouter LM, Dekker J. Effectiveness of exercise therapy: a best-evidence summary of systematic reviews. Australian Journal of Physiotherapy 2005;51(2):71-85. 2005 | Excluded | Lack of relation between the title and the abstract of the article with the subject under study. |
|  | Swan F, Newey A, Bland M, Allgar V, Booth S, Bausewein C, et al. Airflow relieves chronic breathlessness in people with advanced disease: An exploratory systematic review and meta-analyses. Palliative Medicine. 2019;33(6):618-33.https://doi.org/10.1177/0269216319835393 | Excluded | Lack of relation between the title and the abstract of the article with the subject under study. |
|  | Taylor A, DeBoard Z, Gauvin JM. Prevention of Postoperative Pulmonary Complications. Surgical Clinics of North America. 2015;95(2):237-54.https://doi.org/10.1016/j.suc.2014.11.002 | Excluded | Lack of relation between the title and the abstract of the article with the subject under study. |
|  | Tregobov N, Poureslami I, FitzGerald JM. Involving patients and professionals in the development of a conceptual framework for functional health literacy to improve chronic lung disease outcomes. Canadian Journal of Respiratory, Critical Care, and Sleep Medicine. 2020;4:22.https://doi.org/10.1080/24745332.2020.1750227 | Excluded | Lack of relation between the title and the abstract of the article with the subject under study. |
|  | Turner SW, Friend AJ, Okpapi A. Asthma and other recurrent wheezing disorders in children (chronic). BMJ clinical evidence. 2012;2012 | Excluded | Lack of relation between the title and the abstract of the article with the subject under study. |
|  | Van Remoortel H, Giavedoni S, Raste Y, Burtin C, Louvaris Z, Gimeno-Santos E, et al. Validity of activity monitors in health and chronic disease: a systematic review. International Journal of Behavioral Nutrition and Physical Activity. 2012;9.https://doi.org/10.1186/1479-5868-9-84 | Excluded | Lack of relation between the title and the abstract of the article with the subject under study. |
|  | Wang J, Barth J, Göttgens I, Emchi K, Pach D, Oertelt-Prigione S. An opportunity for patient-centered care: Results from a secondary analysis of sex- and gender-based data in mobile health trials for chronic medical conditions. Maturitas. 2020;138:1-7.https://doi.org/10.1016/j.maturitas.2020.05.003 | Excluded | Lack of relation between the title and the abstract of the article with the subject under study. |
|  | Wark P, Wilson AW, Gibson PG. Azoles for allergic bronchopulmonary aspergillosis. The Cochrane database of systematic reviews. 2000(3):Cd001108.https://doi.org/10.1002/14651858.Cd001108 | Excluded | Lack of relation between the title and the abstract of the article with the subject under study. |
|  | Whear R, Thompson‐Coon J, Rogers M, Abbott RA, Anderson L, Ukoumunne O, et al. Patient‐initiated appointment systems for adults with chronic conditions in secondary care. Cochrane Database of Systematic Reviews. 2020(4).https://doi.org/10.1002/14651858.CD010763.pub2 | Excluded | Lack of relation between the title and the abstract of the article with the subject under study. |
|  | Whear R, Thompson-Coon J, Rogers M, Abbott RA, Anderson L, Ukoumunne O, et al. Patient-initiated appointment systems for adults with chronic conditions in secondary care. Cochrane Database of Systematic Reviews. 2020;2020(4).https://doi.org/10.1002/14651858.CD010763.pub2 | Excluded | Lack of relation between the title and the abstract of the article with the subject under study. |
|  | Winski A, Gilbert PAUL, Jao G, Gupta A. VENOVENOUS ECMO AS SALVAGE THERAPY FOR PULMONARY HEMORRHAGE. 2024. p. A3507-A8.10.1016/j.chest.2024.06.2087 | Excluded | Lack of relation between the title and the abstract of the article with the subject under study. |
|  | Wu P, Qian X, Hu Y, Yan X. Effectiveness of Threshold-Pressure Inspiratory Muscle Training on Pulmonary Rehabilitation in Children and Adolescents with Asthma. Journal of Asthma and Allergy. 2024;17:1073-82.https://doi.org/10.2147/JAA.S479398 | Excluded | Lack of relation between the title and the abstract of the article with the subject under study. |
|  | Xavier WDS, Abreu MP, Nunes MDR, Silva-Rodrigues FM, da Silva LF, de Araújo BBM, et al. The Sleep Patterns of Children and Adolescents with Chronic Conditions and Their Families: An Integrative Literature Review. Children. 2024;11(2).https://doi.org/10.3390/children11020207 | Excluded | Lack of relation between the title and the abstract of the article with the subject under study. |
|  | Yohannes AM. Psychosocial Support in Pulmonary Rehabilitation. Respiratory Care. 2024;69(6):664-7.https://doi.org/10.4187/respcare.11850 | Excluded | Lack of relation between the title and the abstract of the article with the subject under study. |
|  | Zhu R, Jia C, Yan J, Luo Y, Huo Z. Primary pulmonary choriocarcinoma in a male that was successfully diagnosed and treated. Medicine (United States). 2016;95(52).https://doi.org/10.1097/MD.0000000000005693 | Excluded | Lack of relation between the title and the abstract of the article with the subject under study. |
|  | Harris K, Kneale D, Lasserson TJ, McDonald VM, Grigg J, Thomas J. School‐based self‐management interventions for asthma in children and adolescents: a mixed methods systematic review. Cochrane Database of Systematic Reviews. 2019(1).https://doi.org/10.1002/14651858.CD011651.pub2 | Excluded | Lack of relation between the title and the abstract of the article with the subject under study. |
|  | Huntley A, White AR, Ernst E. Relaxation therapies for asthma: a systematic review. Thorax 2002 Feb;57(2):127-131. 2002 | Excluded | Lack of relation between the title and the abstract of the article with the subject under study. |
|  | Ahnert J, Loeffler S, Mueller J, Vogel H. Systematic Literature Review on Interventions in Rehabilitation for Children and Adolescents with Asthma Bronchiale. Rehabilitation. 2010;49(3):147-59.https://doi.org/10.1055/s-0030-1254081 | Excluded | Lack of relation between the title and the abstract of the article with the subject under study. |
|  | Andrews J, Sathe NA, Krishnaswami S, McPheeters ML. Nonpharmacologic Airway Clearance Techniques in Hospitalized Patients: A Systematic Review. Respiratory Care. 2013;58(12):2160-86.https://doi.org/10.4187/respcare.02704 | Excluded | Lack of relation between the title and the abstract of the article with the subject under study. |
|  | Ernst E. Systematische reviews zu biofeedback (Systematic reviews of biofeedback) [German]. Physikalische Medizin, Rehabilitationsmedizin, Kurortmedizin 2003 Dec;13(6):321-324. 2003 | Excluded | Lack of relation between the title and the abstract of the article with the subject under study. |
|  | Garagorri-Gutierrez D, Leiros-Rodriguez R. Effects of physiotherapy treatment in patients with bronchial asthma: a systematic review. Physiotherapy Theory and Practice 2022;38(4):493-503. 2022 | Excluded | Lack of relation between the title and the abstract of the article with the subject under study. |
|  | Jin G, Jiang Y, Shao H, Zhu J. The effect of pulmonary rehabilitation on childhood asthma: a systematic review and meta-analysis. Minerva Pediatrics. 2023;75(4):604-13.https://doi.org/10.23736/S2724-5276.21.06656-8 | Excluded | Lack of relation between the title and the abstract of the article with the subject under study. |
|  | Korzh GZ, Korzh NV, Ostrovskyy MM, Kulynych-Miskiv MO, Varunkiv OI, Savelikhina IO, et al. Pulmonary Rehabilitation of Post-COVID-19 Patients with Bronchial Asthma (Review). Tuberculosis, Lung Diseases, HIV Infection. 2024;2024(2):78-83.https://doi.org/10.30978/TB2024-2-78 | Excluded | Lack of relation between the title and the abstract of the article with the subject under study. |
|  | Schuers M, Chapron A, Guihard H, Bouchez T, Darmon D. Impact of non-drug therapies on asthma control: A systematic review of the literature. European Journal of General Practice. 2019;25(2):65-76.https://doi.org/10.1080/13814788.2019.1574742 | Excluded | Lack of relation between the title and the abstract of the article with the subject under study. |
|  | This journal issue contains abstracts that have been accepted for presentation as the 8th Annual Conference of the International Society for Quality of Life Research (ISOQOL). Quality of Life Research. 2001;10(3):193-306.https://doi.org/10.1023/A:1016836728226 | Excluded | Lack of relation between the title and the abstract of the article with the subject under study. |
|  | 21st Annual International Integrative Medicine Conference. Advances in Integrative Medicine. 2015;2(2) | Excluded | Lack of relation between the title and the abstract of the article with the subject under study. |
|  | Abstracts of the 31st Annual Scientific Meeting of the Australasian College for Emergency Medicine. EMA - Emergency Medicine Australasia. 2015;27 | Excluded | Lack of relation between the title and the abstract of the article with the subject under study. |
|  | Meeting Abstracts from the 2nd European Emergency Medical Services Congress, EMS2017. BMJ Open. 2017;7 | Excluded | Lack of relation between the title and the abstract of the article with the subject under study. |
|  | Won P, Choe D, Gomez LH, Justin Gillenwater T, Yenikomshian HA. Minority pediatric burn survivors undergo more burn operations: A single center's five-year experience. Burns. 2024;50(3):760-6.https://doi.org/10.1016/j.burns.2023.10.005 | Excluded | Lack of relation between the title and the abstract of the article with the subject under study. |
|  | Clinical Practice Guidelines. Therapeutic education of the adult and adolescent asthmatic patient. June 2001. Revue de pneumologie clinique. 2002;58(3 Pt 1):169-84 | Excluded | Lack of relation between the title and the abstract of the article with the subject under study. |
|  | American Thoracic Society/European Respiratory Society statement: Standards for the diagnosis and management of individuals with alpha-1 antitrypsin deficiency. American Journal of Respiratory and Critical Care Medicine. 2003;168(7):818-900.https://doi.org/10.1164/rccm.168.7.818 | Excluded | Lack of relation between the title and the abstract of the article with the subject under study. |
|  | Two Cochrane reviews: prevention of asthma attacks, and cancer-related fatigue. HealthFacts. 2008;33(5):4- | Excluded | Lack of relation between the title and the abstract of the article with the subject under study. |
|  | When the child has a fever. Drug and Therapeutics Bulletin. 2008;46(3):17-20.https://doi.org/10.1136/dtb.2008.03.0005 | Excluded | Lack of relation between the title and the abstract of the article with the subject under study. |
|  | European Respiratory Society Annual Congress 2012. European Respiratory Journal. 2012;40 | Excluded | Lack of relation between the title and the abstract of the article with the subject under study. |
|  | Article summaries for october 2012 psychosomatic medicine, vol. 74, issue 8: Http://www.psychosomaticmedicine.org/content/74/8.toc. Psychosomatic Medicine. 2012;74(8):785.https://doi.org/10.1097/PSY.0b013e3182754207 | Excluded | Lack of relation between the title and the abstract of the article with the subject under study. |
|  | Chronic obstructive pulmonary disease (COPD) evidentiary framework. Ontario Health Technology Assessment Series. 2012;12(2):1-97 | Excluded | Lack of relation between the title and the abstract of the article with the subject under study. |
|  | Assessment of asthma control: which instrument is right for the New Zealand context?...Abstracts from the Physiotherapy New Zealand Conference, held in Wellington on 5th - 6th May 2012. New Zealand Journal of Physiotherapy. 2012;40(2):92- | Excluded | Lack of relation between the title and the abstract of the article with the subject under study. |
|  | 2013 CAEP/ACMU Scientific Abstracts, CAEP 2013. Canadian Journal of Emergency Medicine. 2013;15:S1 | Excluded | Lack of relation between the title and the abstract of the article with the subject under study. |
|  | The cochrane database of systematic reviews - issue 4 2013. Journal of Evidence-Based Medicine. 2013;6(2):115-6.https://doi.org/10.1111/jebm.12045 | Excluded | Lack of relation between the title and the abstract of the article with the subject under study. |
|  | Editorial introductions. Current Opinion in Pulmonary Medicine. 2013;19(4):v-vi.https://doi.org/10.1097/MCP.0b013e32836278ec | Excluded | Lack of relation between the title and the abstract of the article with the subject under study. |
|  | Targinact for restless legs syndrome. Drug and Therapeutics Bulletin. 2016;54(4):42-5.https://doi.org/10.1136/dtb.2016.4.0393 | Excluded | Lack of relation between the title and the abstract of the article with the subject under study. |
|  | Marker AM, Steele RG, Noser AE. Physical activity and health-related quality of life in children and adolescents: A systematic review and meta-analysis. Health Psychology. 2018;37(10):893-903.https://doi.org/10.1037/hea0000653 | Excluded | Lack of relation between the title and the abstract of the article with the subject under study. |
|  | Qimin W, Feng Y, Lianjun G, Wei G. Effects of Inspiratory Muscle Training and High-Intensity Interval Training on Lung Function and Respiratory Muscle Function in Asthma. Respiratory Care. 2022;67(11):1465-75.https://doi.org/10.4187/respcare.09813 | Excluded | Lack of relation between the title and the abstract of the article with the subject under study. |
|  | Zampogna E, Oliva FM, Del Furia MJ, Cordani C, Lazzarini SG, et al. Effectiveness of rehabilitation interventions in adults with asthma: a systematic review and meta-analysis. American journal of physical medicine & rehabilitation. 2024.https://doi.org/10.1097/PHM.0000000000002552 | Excluded | Lack of relation between the title and the abstract of the article with the subject under study. |
|  | Aaron SD, de Oca MM, Celli B, Bhatt SP, Bourbeau J, Criner GJ, et al. Early Diagnosis and Treatment of Chronic Obstructive Pulmonary Disease The Costs and Benefits of Case Finding. American Journal of Respiratory and Critical Care Medicine. 2024;209(8):928-37.https://doi.org/10.1164/rccm.202311-2120PP | Excluded | Lack of relation between the title and the abstract of the article with the subject under study. |
|  | Abrams TE, Blevins A, Vander Weg MW. Chronic obstructive lung disease and posttraumatic stress disorder: Current perspectives. International Journal of COPD. 2015;10(1):2219-33.https://doi.org/10.2147/COPD.S71449 | Excluded | Lack of relation between the title and the abstract of the article with the subject under study. |
|  | Abramson MJ, Schattner RL, Holton C, Simpson P, Briggs N, Beilby J, et al. Spirometry and Regular Follow-up do not Improve Quality of Life in Children or Adolescents With Asthma: Cluster Randomized Controlled Trials. Pediatric Pulmonology. 2015;50(10):947-54.https://doi.org/10.1002/ppul.23096 | Excluded | Lack of relation between the title and the abstract of the article with the subject under study. |
|  | Abu Abed M, Himmel W, Vormfelde S, Koschack J. Video-assisted patient education to modify behavior: A systematic review. Patient Education and Counseling. 2014;97(1):16-22.https://doi.org/10.1016/j.pec.2014.06.015 | Excluded | Lack of relation between the title and the abstract of the article with the subject under study. |
|  | Aburayya BI, Obeidat LR, Kitana FI, Al Khatib O, Romman S, Hamed OH. Complete Common Bile Duct Injury after Laparoscopic Cholecystectomy in Situs Inversus Totalis: A Case Report, Review of the Literature and Illustrative Case Video. International Journal of Surgery Case Reports. 2024;115.https://doi.org/10.1016/j.ijscr.2024.109265 | Excluded | Lack of relation between the title and the abstract of the article with the subject under study. |
|  | Aburub A, Ledger SJ, Sim J, Hunter SM. Cardiopulmonary Function and Aerobic Exercise in Parkinson's: A Systematic Review of the Literature. Movement disorders clinical practice. 2020;7(6):599-606.https://doi.org/10.1002/mdc3.13011 | Excluded | Lack of relation between the title and the abstract of the article with the subject under study. |
|  | Aceves SS, Wasserman SI. Evaluating and treating asthma. Emergency Medicine. 2005;37(4):20-9 | Excluded | Lack of relation between the title and the abstract of the article with the subject under study. |
|  | Acuna-Izcaray A, Sanchez-Angarita E, Plaza V, Rodrigo G, Montes de Oca M, Gich I, et al. Quality assessment of asthma clinical practice guidelines: a systematic appraisal. Chest 2013 Aug;144(2):390-397. 2013 | Excluded | Lack of relation between the title and the abstract of the article with the subject under study. |
|  | Adams BK, Cydulka RK. Asthma evaluation and management. Emergency Medicine Clinics of North America. 2003;21(2):315-30.https://doi.org/10.1016/S0733-8627(03)00015-4 | Excluded | Lack of relation between the title and the abstract of the article with the subject under study. |
|  | Adawi M, Watad A, Brown S, Aazza K, Aazza H, Zouhir M, et al. Ramadan fasting exerts immunomodulatory effects: Insights from a systematic review. Frontiers in Immunology. 2017;8(NOV).https://doi.org/10.3389/fimmu.2017.01144 | Excluded | Lack of relation between the title and the abstract of the article with the subject under study. |
|  | Adeboye B, Bermano G, Rolland C. Obesity and its health impact in Africa: A systematic review. Cardiovascular Journal of Africa. 2012;23(9):512-21.https://doi.org/10.5830/CVJA-2012-040 | Excluded | Lack of relation between the title and the abstract of the article with the subject under study. |
|  | Adeniyi FB, Young T. Weight loss interventions for chronic asthma. Cochrane database of systematic reviews (Online). 2012;7:CD009339 | Excluded | Lack of relation between the title and the abstract of the article with the subject under study. |
|  | Adir Y, Bove AA. Can asthmatic subjects dive? European Respiratory Review. 2016;25(140):214-20.https://doi.org/10.1183/16000617.0006-2016 | Excluded | Lack of relation between the title and the abstract of the article with the subject under study. |
|  | Aditama TY. Smoking problem in Indonesia. Medical Journal of Indonesia. 2002;11(1):56-65.https://doi.org/10.13181/mji.v11i1.52 | Excluded | Lack of relation between the title and the abstract of the article with the subject under study. |
|  | Afrin K, Neelam B. A Systematic Review & Meta-Analysis on Interrelating Comorbid Conditions and Breathing Practices. International Journal of Current Pharmaceutical Review and Research. 2022;14(4):40-9 | Excluded | Lack of relation between the title and the abstract of the article with the subject under study. |
|  | Agard C, Rendu E, Leguern V, Ponge T, Masseau A, Barrier JH, et al. Churg-Strauss Syndrome Revealed by Granulomatous Acute Pericarditis: Two Case Reports and a Review of the Literature. Seminars in Arthritis and Rheumatism. 2007;36(6):386-91.https://doi.org/10.1016/j.semarthrit.2006.12.002 | Excluded | Lack of relation between the title and the abstract of the article with the subject under study. |
|  | Agence Nationale d'Accreditation et d'Evaluation en S. Clinical Practice Guidelines. Therapeutic education of the adult and adolescent asthmatic patient. June 2001. Revue de pneumologie clinique. 2002;58(3 Pt 1):169-84 | Excluded | Lack of relation between the title and the abstract of the article with the subject under study. |
|  | Aggarwal AN, Agarwal R. Bronchodilator responsiveness in chronic obstructive pulmonary disease: prevalence, significance, and clinical implications. Current Opinion in Pulmonary Medicine. 2025;31(2):126-34.https://doi.org/10.1097/MCP.0000000000001143 | Excluded | Lack of relation between the title and the abstract of the article with the subject under study. |
|  | Aghili SMM, Ebrahimpur M, Arjmand B, Shadman Z, Pejman Sani M, Qorbani M, et al. Obesity in COVID-19 era, implications for mechanisms, comorbidities, and prognosis: a review and meta-analysis. International Journal of Obesity. 2021;45(5):998-1016.https://doi.org/10.1038/s41366-021-00776-8 | Excluded | Lack of relation between the title and the abstract of the article with the subject under study. |
|  | Agrawal A, Baird BJ, Madariaga MLL, Blair EA, Murgu S. Multi-disciplinary management of patients with benign airway strictures: A review: Management of Benign Airway Strictures. Respiratory Medicine. 2021;187.https://doi.org/10.1016/j.rmed.2021.106582 | Excluded | Lack of relation between the title and the abstract of the article with the subject under study. |
|  | Ahmad S, Ismail NE. Stigma in the lives of asthma patients: A review from the literature. International Journal of Pharmacy and Pharmaceutical Sciences. 2015;7(7):40-6 | Excluded | Lack of relation between the title and the abstract of the article with the subject under study. |
|  | Ahmad SR, Iyer VN. The Evolving Clinical Practice of Chronic Cough. Mayo Clinic Proceedings. 2022;97(6):1164-75.https://doi.org/10.1016/j.mayocp.2022.02.005 | Excluded | Lack of relation between the title and the abstract of the article with the subject under study. |
|  | Ahmadikia K, Hashemi SJ, Khodavaisy S, Getso MI, Alijani N, Badali H, et al. The double-edged sword of systemic corticosteroid therapy in viral pneumonia: A case report and comparative review of influenza-associated mucormycosis versus COVID-19 associated mucormycosis. Mycoses. 2021;64(8):798-808.https://doi.org/10.1111/myc.13256 | Excluded | Lack of relation between the title and the abstract of the article with the subject under study. |
|  | Ahmadzai H, Huang S, Hettiarachchi R, Lin JL, Thomas PS, Zhang Q. Exhaled breath condensate: A comprehensive update. Clinical Chemistry and Laboratory Medicine. 2013;51(7):1343-61.https://doi.org/10.1515/cclm-2012-0593 | Excluded | Lack of relation between the title and the abstract of the article with the subject under study. |
|  | Ahmed S, Steed L, Harris K, Taylor SJC, Pinnock H. Interventions to enhance the adoption of asthma self-management behaviour in the South Asian and African American population: A systematic review. npj Primary Care Respiratory Medicine. 2018;28(1).https://doi.org/10.1038/s41533-017-0070-6 | Excluded | Lack of relation between the title and the abstract of the article with the subject under study. |
|  | Ahmetaj L, Martínez-Torres AE, Ahmetaj Y, Gashi V, Kurhasani X, Lokaj-Berisha V, et al. Prevalence of atopic eczema in adolescents from a very low prevalence area (Kosovo): role of wheezing, gender, exercise, and paracetamol. Allergologia et Immunopathologia. 2024;52(6):40-50.https://doi.org/10.15586/aei.v52i6.1155 | Excluded | Lack of relation between the title and the abstract of the article with the subject under study. |
|  | Ahmetaj L, Martinez-Torres AE, Ahmetaj Y, Ismajli I, Gashi V, Kurhasani X, et al. The role of Human Development Index in the epidemiology of asthma in adolescents in Kosovo: A cross-sectional multicentre Global Asthma Network (GAN) study. Allergologia Et Immunopathologia. 2023;51(2):59-70.https://doi.org/10.15586/aei.v51i2.781 | Excluded | Lack of relation between the title and the abstract of the article with the subject under study. |
|  | Aigon A, Billecocq S. Prevalence and impact on quality of life of urinary incontinence in an adult population with chronic obstructive pulmonary diseases, literature review. Progres en Urologie. 2018;28(17):962-72.https://doi.org/10.1016/j.purol.2018.08.016 | Excluded | Lack of relation between the title and the abstract of the article with the subject under study. |
|  | Akuzum F, Senel A, Polat B, Kardes K, Aslan GK. Physiotherapy interventions on chest wall mobility in obstructive lung diseases: A systematic review. Journal of Bodywork and Movement Therapies. 2024;38:368-74.https://doi.org/10.1016/j.jbmt.2023.11.046 | Excluded | Lack of relation between the title and the abstract of the article with the subject under study. |
|  | al Aloola NA, Naik-Panvelkar P, Nissen L, Saini B. Asthma interventions in primary schools -- a review. The Journal of Asthma 2014;51(8):779-798. 2014 | Excluded | Lack of relation between the title and the abstract of the article with the subject under study. |
|  | Al Hammadi A, Parmar NV, Aljefri K, Al Sharif O, Abdallah M, Ahmed HM, et al. Review on Alopecia Areata in the Middle East and Africa: Landscape and Unmet Needs. Dermatology and Therapy. 2023;13(7):1435-64.https://doi.org/10.1007/s13555-023-00946-8 | Excluded | Lack of relation between the title and the abstract of the article with the subject under study. |
|  | Al Qasem A, Smith F, Clifford S. Adherence to medication among chronic patients in Middle Eastern countries: Review of studies. Eastern Mediterranean Health Journal. 2011;17(4):356-63.https://doi.org/10.26719/2011.17.4.356 | Excluded | Lack of relation between the title and the abstract of the article with the subject under study. |
|  | Al-Alwan A, Kaminsky D. Vocal cord dysfunction in athletes: Clinical presentation and review of the literature. Physician and Sportsmedicine. 2012;40(2).https://doi.org/10.3810/psm.2012.05.1961 | Excluded | Lack of relation between the title and the abstract of the article with the subject under study. |
|  | Alanazi MS, Degenhardt B, Franklin G, Jacobson E, Fritz S, Kettner N, et al. Autonomic nervous system and viscera-related responses to manual therapy: A narrative overview. International Journal of Osteopathic Medicine. 2024;54.https://doi.org/10.1016/j.ijosm.2024.100735 | Excluded | Lack of relation between the title and the abstract of the article with the subject under study. |
|  | Al-Aqeel S. Nonadherence to antiseizure medications: what have we learned and what can be done next? Expert Review of Pharmacoeconomics and Outcomes Research. 2024;24(7):791-8.https://doi.org/10.1080/14737167.2024.2349191 | Excluded | Lack of relation between the title and the abstract of the article with the subject under study. |
|  | Albers FC, Bratton DJ, Gunsoy NB, Cockle SM, Alfonso-Cristancho R, Braunstahl GJ. Mepolizumab improves work productivity, activity limitation, symptoms, and rescue medication use in severe eosinophilic asthma. Clinical Respiratory Journal. 2022;16(3):252-8.https://doi.org/10.1111/crj.13474 | Excluded | Lack of relation between the title and the abstract of the article with the subject under study. |
|  | Albuquerque Baltar J, Socorro Brasileiro Santos MD, da Silva HJ, de Pontes Filho NT. Does asthma promote changes in static posture? – Systematic review. Revista Portuguesa de Pneumologia (English Edition). 2010;16(3):471-6.https://doi.org/10.1016/S2173-5115(10)70051-8 | Excluded | Lack of relation between the title and the abstract of the article with the subject under study. |
|  | Alcantara J, Alcantara JD. The chiropractic care of patients with asthma: a systematic review of the literature to inform clinical practice. Clinical Chiropractic 2012 Mar;15(1):23-30. 2012 | Excluded | Lack of relation between the title and the abstract of the article with the subject under study. |
|  | Alexander DD, Bailey WH, Perez V, Mitchell ME, Su S. Air ions and respiratory function outcomes: A comprehensive review. Journal of Negative Results in BioMedicine. 2013;12(1).https://doi.org/10.1186/1477-5751-12-14 | Excluded | Lack of relation between the title and the abstract of the article with the subject under study. |
|  | Alfraji N, Upadhyaya VD, Bekampis C, Kuzyshyn H. Mixed Cryoglobulinemia Syndrome (MCS) due to untreated hepatitis B with uncommon presentation: case report and literature review. BMC Rheumatology. 2020;4(1).https://doi.org/10.1186/s41927-020-00159-y | Excluded | Lack of relation between the title and the abstract of the article with the subject under study. |
|  | Alhamed MS, Alharbi F, Al Joher A, Dhahry S, Fallatah AA, Alanazi OH, et al. Vitamin D Deficiency in Children and Adolescents in Saudi Arabia: A Systematic Review. Cureus. 2024;16(1):e52040.https://doi.org/10.7759/cureus.52040 | Excluded | Lack of relation between the title and the abstract of the article with the subject under study. |
|  | Alharbi AS, Yousef AA, Alharbi SA, Almaghamsi TM, Al Qwaiee MM, Al-Somali FM, et al. Severe asthma in children An official statement from Saudi Pediatric Pulmonology Association. Saudi Medical Journal. 2022;43(4):329-40.https://doi.org/10.15537/SMJ.2022.4.43.20210756 | Excluded | Lack of relation between the title and the abstract of the article with the subject under study. |
|  | Alhosain D, Kouba L. Concurrent cerebral arterial and venous sinus thrombosis revealing celiac disease-a case report and literature review. BMC Gastroenterology. 2020;20(1).https://doi.org/10.1186/s12876-020-01483-w | Excluded | Lack of relation between the title and the abstract of the article with the subject under study. |
|  | Alhucema P, Jindal L, Subiramanian S, Turley J, King A, Brown I. Mindfulness for resilience in chronic atopic conditions-A matter of mind over body. Internal Medicine Journal. 2018;48:10-1.https://doi.org/10.1111/imj.14077 | Excluded | Lack of relation between the title and the abstract of the article with the subject under study. |
|  | Ali H, Panettieri Jr RA. Anaphylatoxin C3a receptors in asthma. Respiratory Research. 2005;6.https://doi.org/10.1186/1465-9921-6-19 | Excluded | Lack of relation between the title and the abstract of the article with the subject under study. |
|  | Ali MU, Liu G, Yousaf B, Ullah H, Abbas Q, Munir MAM. A systematic review on global pollution status of particulate matter-associated potential toxic elements and health perspectives in urban environment. Environmental Geochemistry and Health. 2019;41(3):1131-62.https://doi.org/10.1007/s10653-018-0203-z | Excluded | Lack of relation between the title and the abstract of the article with the subject under study. |
|  | Ali N, Rosenbloom C. MOVING MEDICINE: PROMOTING INCREASED PHYSICAL ACTIVITY IN CHILDREN AND ADOLESCENTS WITH ASTHMA THROUGH EVIDENCE BASED INFORMATION LEAFLETS. Archives of Disease in Childhood. 2022;107:A63.https://doi.org/10.1136/archdischild-2022-rcpch.104 | Excluded | Lack of relation between the title and the abstract of the article with the subject under study. |
|  | Ali RAR, Hassan J, Egan LJ. Review of recent evidence on the management of heartburn in pregnant and breastfeeding women. BMC Gastroenterology. 2022;22(1).https://doi.org/10.1186/s12876-022-02287-w | Excluded | Lack of relation between the title and the abstract of the article with the subject under study. |
|  | Ali Z, Ulrik CS. Obesity and asthma: A coincidence or a causal relationship? A systematic review. Respiratory Medicine. 2013;107(9):1287-300.https://doi.org/10.1016/j.rmed.2013.03.019 | Excluded | Lack of relation between the title and the abstract of the article with the subject under study. |
|  | Ali Z, Ulrik CS, Agner T, Thomsen SF. Is atopic dermatitis associated with obesity? A systematic review of observational studies. Journal of the European Academy of Dermatology and Venereology. 2018;32(8):1246-55.https://doi.org/10.1111/jdv.14879 | Excluded | Lack of relation between the title and the abstract of the article with the subject under study. |
|  | Alinaghi SAS, Karimi A, Pashaei Z, Afzalian A, Ghorbanzadeh K, Ghasemzadeh A, et al. Safety and Adverse Events Related to COVID-19 mRNA Vaccines; a Systematic Review. Archives of Academic Emergency Medicine. 2022;10(1).https://doi.org/10.22037/aaem.v10i1.1597 | Excluded | Lack of relation between the title and the abstract of the article with the subject under study. |
|  | Al-Jahdali H, Alshimemeri A, Mobeireek A, Albanna A, Al Shirawi N, Wali S, et al. The Saudi Thoracic Society guidelines for diagnosis and management of noncystic fibrosis bronchiectasis. Annals of Thoracic Medicine. 2017;12(3):135-61.https://doi.org/10.4103/atm.ATM_171_17 | Excluded | Lack of relation between the title and the abstract of the article with the subject under study. |
|  | Aljubran SA, Whelan GJ, Glaum MC, Lockey RF. Osteoporosis in the at-risk asthmatic. Allergy: European Journal of Allergy and Clinical Immunology. 2014;69(11):1429-39.https://doi.org/10.1111/all.12438 | Excluded | Lack of relation between the title and the abstract of the article with the subject under study. |
|  | Allado E, Poussel M, Hily O, Chenuel B. The interest of rehabilitation of respiratory disorders in athletes: Myth or reality? Annals of Physical and Rehabilitation Medicine. 2022;65(4).https://doi.org/10.1016/j.rehab.2020.101461 | Excluded | Lack of relation between the title and the abstract of the article with the subject under study. |
|  | Allen SC, Siddique N. Inhaler therapy in old age: The critical role of cognitive function. CME Journal Geriatric Medicine. 2006;8(2):66-71 | Excluded | Lack of relation between the title and the abstract of the article with the subject under study. |
|  | Al-Moamary M, Alhaider S, Idrees M, Al Ghobain M, Zeitouni M, Al-Harbi A, et al. The Saudi Initiative for Asthma-2016 update: Guidelines for the diagnosis and management of asthma in adults and children. Annals of Thoracic Medicine. 2016;11(1):3-42.https://doi.org/10.4103/1817-1737.173196 | Excluded | Lack of relation between the title and the abstract of the article with the subject under study. |
|  | Al-Moamary M, Al-Hajjaj M, Idrees M, Zeitouni M, Alanezi M, Al-Jahdal H, et al. The Saudi initiative for asthma. Annals of Thoracic Medicine. 2009;4(4):216-33.https://doi.org/10.4103/1817-1737.56001 | Excluded | Lack of relation between the title and the abstract of the article with the subject under study. |
|  | Alotaibi S, House R. Diagnosis of occupational asthma: Review. Bahrain Medical Bulletin. 2000;22(1):30-4 | Excluded | Lack of relation between the title and the abstract of the article with the subject under study. |
|  | Al-Qudimat AR, Al Darwish MB, Elaarag M, Al-Zoubi RM, Rejeb MA, Ojha LK, et al. COVID-19 effect on patients with noncommunicable diseases: A narrative review. Health Science Reports. 2023;6(1).https://doi.org/10.1002/hsr2.995 | Excluded | Lack of relation between the title and the abstract of the article with the subject under study. |
|  | Alreshidi N, Al-Kalaldeh M. The impact of asthma education programs on children's life aspects: A systematic review. Current Respiratory Medicine Reviews. 2021;17(1):20-8.https://doi.org/10.2174/1573398X17666210129130547 | Excluded | Lack of relation between the title and the abstract of the article with the subject under study. |
|  | Alshabanat A, Zafari Z, Albanyan O, Dairi M, FitzGerald JM. Asthma and COPD overlap syndrome (ACOS): A systematic review and meta analysis. PLoS ONE. 2015;10(9).https://doi.org/10.1371/journal.pone.0136065 | Excluded | Lack of relation between the title and the abstract of the article with the subject under study. |
|  | Althobiani MA, Evans RA, Alqahtani JS, Aldhahir AM, Russell A-M, Hurst JR, et al. Home monitoring of physiology and symptoms to detect interstitial lung disease exacerbations and progression: a systematic review. Erj Open Research. 2021;7(4).https://doi.org/10.1183/23120541.00441-2021 | Excluded | Lack of relation between the title and the abstract of the article with the subject under study. |
|  | Althoff M, Holguin F. Contemporary management techniques of asthma in obese patients. Expert Review of Respiratory Medicine. 2020;14(3):249-57.https://doi.org/10.1080/17476348.2020.1706486 | Excluded | Lack of relation between the title and the abstract of the article with the subject under study. |
|  | Altieri S. Variant form of angina pectoris. (Review of literature; case reports; nosologic classification; physiopathogenetic aspects). Minerva Cardioangiologica. 1972;20(5):239-57 | Excluded | Lack of relation between the title and the abstract of the article with the subject under study. |
|  | Amalakuhan B, Maselli DJ, Martinez-Garcia MA. Update in bronchiectasis 2014. American Journal of Respiratory and Critical Care Medicine. 2015;192(10):1155-61.https://doi.org/10.1164/rccm.201505-0926UP | Excluded | Lack of relation between the title and the abstract of the article with the subject under study. |
|  | Amati F, Leonardi G, Contarini M, Morlacchi LC, Stainer A, Pizzamiglio G, et al. Immunodeficiencies and CFTR dysfunction: results from a systematic screening in a cohort of adults with cystic fibrosis and CFTR-related disorders. Therapeutic Advances in Respiratory Disease. 2024;18.https://doi.org/10.1177/17534666241253945 | Excluded | Lack of relation between the title and the abstract of the article with the subject under study. |
|  | Ambrosino P, Accardo M, Mosella M, Papa A, Fuschillo S, Spedicato GA, et al. Performance of fractional exhaled nitric oxide in predicting response to inhaled corticosteroids in chronic cough: a meta-analysis. Annals of Medicine. 2021;53(1):1659-72.https://doi.org/10.1080/07853890.2021.1979242 | Excluded | Lack of relation between the title and the abstract of the article with the subject under study. |
|  | Ambrosino P, Marcuccio G, Raffio G, Formisano R, Candia C, Manzo F, et al. Endotyping Chronic Respiratory Diseases: T2 Inflammation in the United Airways Model. Life. 2024;14(7).https://doi.org/10.3390/life14070899 | Excluded | Lack of relation between the title and the abstract of the article with the subject under study. |
|  | Amico A, Mammino L, Palmucci S, Latino R, Milone P, Li Destri G, et al. Giant hepatic hemangioma case report: When is it time for surgery? Annals of Medicine and Surgery. 2020;58:4-7.https://doi.org/10.1016/j.amsu.2020.08.003 | Excluded | Lack of relation between the title and the abstract of the article with the subject under study. |
|  | Amoah AS, Forson AG, Boakye DA. A review of epidemiological studies of asthma in Ghana. Ghana medical journal. 2012;46(2 Suppl):23-8 | Excluded | Lack of relation between the title and the abstract of the article with the subject under study. |
|  | An F, Liu J, Lu W, Jareemit D. A review of the effect of traffic-related air pollution around schools on student health and its mitigation. Journal of Transport and Health. 2021;23.https://doi.org/10.1016/j.jth.2021.101249 | Excluded | Lack of relation between the title and the abstract of the article with the subject under study. |
|  | An R, Ji M, Yan H, Guan C. Impact of ambient air pollution on obesity: a systematic review. International Journal of Obesity. 2018;42(6):1112-26.https://doi.org/10.1038/s41366-018-0089-y | Excluded | Lack of relation between the title and the abstract of the article with the subject under study. |
|  | An W, Li T, Tian X, Fu X, Li C, Wang Z, et al. Allergies to Allergens from Cats and Dogs: A Review and Update on Sources, Pathogenesis, and Strategies. International journal of molecular sciences. 2024;25(19).https://doi.org/10.3390/ijms251910520 | Excluded | Lack of relation between the title and the abstract of the article with the subject under study. |
|  | Anagnostou K. Anaphylaxis in children: Epidemiology, risk factors and management. Current Pediatric Reviews. 2018;14(3):180-6.https://doi.org/10.2174/1573396314666180507115115 | Excluded | Lack of relation between the title and the abstract of the article with the subject under study. |
|  | Anand A, Castiglia E, Zamora ML. The Association Between Personal Air Pollution Exposures and Fractional Exhaled Nitric Oxide (FeNO): A Systematic Review. Current Environmental Health Reports. 2024;11(2):210-24.https://doi.org/10.1007/s40572-024-00430-1 | Excluded | Lack of relation between the title and the abstract of the article with the subject under study. |
|  | Anandavelu R, Turner AM. The Role of Gefapixant in the Management of Chronic Cough. European Respiratory and Pulmonary Diseases. 2022;7(1):15-20 | Excluded | Lack of relation between the title and the abstract of the article with the subject under study. |
|  | Anderson K, Burford O, Emmerton L. App Chronic Disease Checklist: Protocol to Evaluate Mobile Apps for Chronic Disease Self-Management. JMIR research protocols. 2016;5(4):e204-e | Excluded | Lack of relation between the title and the abstract of the article with the subject under study. |
|  | Anderson WC, Baptist AP, Eakin MN, Federman A, Murphy VE. Adherence Challenges and Strategies in Specific Groups With Asthma: Adolescents, Pregnancy, and Older Adults. Journal of Allergy and Clinical Immunology: In Practice. 2024;12(12):3216-22.https://doi.org/10.1016/j.jaip.2024.07.031 | Excluded | Lack of relation between the title and the abstract of the article with the subject under study. |
|  | Angelow A, Black N. The use and impact of national confidential enquiries in high-income countries. Bmj Quality & Safety. 2011;20(1):38-45.https://doi.org/10.1136/bmjqs.2010.040477 | Excluded | Lack of relation between the title and the abstract of the article with the subject under study. |
|  | Ansar S, Coveliers A, De Bruyn S, Janssen T, Oostermeyer R, Wille F, et al. Peer support in paediatrics: A literature review. Journal of Paediatrics and Child Health. 2024;60(12):783-8.https://doi.org/10.1111/jpc.16703 | Excluded | Lack of relation between the title and the abstract of the article with the subject under study. |
|  | Anselmo-Lima WT, Sakano E, Araripe Nunes AA, Fernandes AM, Tamashiro E, Pereira EA, et al. Rhinosinusitis: Evidence and experience. October 18 and 19, 2013-São Paulo. Brazilian Journal of Otorhinolaryngology. 2015;81:S1-S49.https://doi.org/10.1016/j.bjorl.2015.01.003 | Excluded | Lack of relation between the title and the abstract of the article with the subject under study. |
|  | Antó JM. Recent advances in the epidemiologic investigation of risk factors for Asthma: A review of the 2011 literature. Current Allergy and Asthma Reports. 2012;12(3):192-200.https://doi.org/10.1007/s11882-012-0254-7 | Excluded | Lack of relation between the title and the abstract of the article with the subject under study. |
|  | Appleton S, Jones T, Poole P, Pilotto L, Adams R, Lasserson TJ, et al. Ipratropium bromide versus long-acting beta-2 agonists for stable chronic obstructive pulmonary disease. The Cochrane database of systematic reviews. 2006;2006(3):Cd006101.https://doi.org/10.1002/14651858.Cd006101 | Excluded | Lack of relation between the title and the abstract of the article with the subject under study. |
|  | Arikan-Ayyildiz Z, Işik S, Çaǧlayan-Sözmen Ş, Karaman Ö, Uzuner N. Cold, cholinergic and aquagenic urticaria in children: Presentation of three cases and review of the literature. Turkish Journal of Pediatrics. 2013;55(1):94-8 | Excluded | Lack of relation between the title and the abstract of the article with the subject under study. |
|  | Asai N, Ohkuni Y, Kato H, Hagihara M, Mikamo H, Kaneko N. COPD Pathogenesis and Alterations in the Oral, Lung, and Gut Microbiomes. Microbiology Research. 2024;15(3):1605-15.https://doi.org/10.3390/microbiolres15030106 | Excluded | Lack of relation between the title and the abstract of the article with the subject under study. |
|  | Atag E, Krivec U, Ersu R. Non-invasive Ventilation for Children With Chronic Lung Disease. Frontiers in Pediatrics. 2020;8.https://doi.org/10.3389/fped.2020.561639 | Excluded | Lack of relation between the title and the abstract of the article with the subject under study. |
|  | Atay Z, Bereket A. Current status on obesity in childhood and adolescence: Prevalence, etiology, co-morbidities and management. Obesity Medicine. 2016;3:1-9.https://doi.org/10.1016/j.obmed.2016.05.005 | Excluded | Lack of relation between the title and the abstract of the article with the subject under study. |
|  | Augusti KT, Jose R, Sajitha GR, Augustine P. A rethinking on the benefits and drawbacks of common antioxidants and a proposal to look for the antioxidants in Allium products as ideal agents: A review. Indian Journal of Clinical Biochemistry. 2012;27(1):6-20.https://doi.org/10.1007/s12291-011-0146-y | Excluded | Lack of relation between the title and the abstract of the article with the subject under study. |
|  | Ayre S, Walters G. Are therapeutic decisions made on the medical admissions unit any more evidence-based than they used to be? Journal of Evaluation in Clinical Practice. 2009;15(6):1180-6.https://doi.org/10.1111/j.1365-2753.2009.01345.x | Excluded | Lack of relation between the title and the abstract of the article with the subject under study. |
|  | Azizpour Y, Delpisheh A, Montazeri Z, Sayehmiri K, Darabi B. Effect of childhood BMI on asthma: a systematic review and meta-analysis of case-control studies. Bmc Pediatrics. 2018;18.https://doi.org/10.1186/s12887-018-1093-z | Excluded | Lack of relation between the title and the abstract of the article with the subject under study. |
|  | Bain E, Pierides KL, Clifton VL, Hodyl NA, Stark MJ, Crowther CA, et al. Interventions for managing asthma in pregnancy. Cochrane Database of Systematic Reviews. 2014;2014(10).https://doi.org/10.1002/14651858.CD010660.pub2 | Excluded | Lack of relation between the title and the abstract of the article with the subject under study. |
|  | Bain E, Pierides KL, Middleton P, Clifton VL, Hodyl NA, Stark MJ, et al. Interventions for managing asthma in pregnancy. Cochrane Database of Systematic Reviews. 2013;2013(7).https://doi.org/10.1002/14651858.CD010660 | Excluded | Lack of relation between the title and the abstract of the article with the subject under study. |
|  | Bakhtiari E, Moazzen N. Pulmonary function in children post -SARS-CoV-2 infection: a systematic review and meta-analysis. BMC Pediatrics. 2024;24(1).https://doi.org/10.1186/s12887-024-04560-1 | Excluded | Lack of relation between the title and the abstract of the article with the subject under study. |
|  | Baldin Tiguman GM, Ferreira Rocha de Alencar RR, Penha AdP, Galvao TF, Silva MT. Prevalence of self-reported asthma in adults in the Brazilian Amazon: a population-based cross-sectional study. Jornal Brasileiro De Pneumologia. 2020;46(4).https://doi.org/10.36416/1806-3756/e20200086 | Excluded | Lack of relation between the title and the abstract of the article with the subject under study. |
|  | Balli F. Game jams to co-create respiratory health games prototypes as participatory research methodology. Forum Qualitative Sozialforschung. 2018;19(3).https://doi.org/10.17169/fqs-19.3.2734 | Excluded | Lack of relation between the title and the abstract of the article with the subject under study. |
|  | Balon JW, Mior SA. Chiropractic care in asthma and allergy. Annals of Allergy, Asthma and Immunology. 2004;93(2 SUPPL. 1):S55-S60.https://doi.org/10.1016/S1081-1206(10)61487-1 | Excluded | Lack of relation between the title and the abstract of the article with the subject under study. |
|  | Baltar JA, Brasileiro Santos MdS, da Silva HJ. Does asthma promote changes in static posture? - Systematic review. Revista Portuguesa De Pneumologia. 2010;16(3):471-6.https://doi.org/10.1016/s0873-2159(15)30043-x | Excluded | Lack of relation between the title and the abstract of the article with the subject under study. |
|  | Banh HL. Unconventional treatment options in severe asthma: An overview. Journal of Pharmacy and Pharmaceutical Sciences. 2011;14(3):387-99.https://doi.org/10.18433/j37s36 | Excluded | Lack of relation between the title and the abstract of the article with the subject under study. |
|  | Bannon JP, Fater M, Solit R. Intestinal ileus secondary to strongyloides stercoralis infection: Case report and review of the literature. American Surgeon. 1995;61(4):377-80 | Excluded | Lack of relation between the title and the abstract of the article with the subject under study. |
|  | Bar C, Cheuret E, Bessou P, Pedespan JM. Childhood idiopathic spinal cord infarction: Description of 7 cases and review of the literature. Brain and Development. 2017;39(10):818-27.https://doi.org/10.1016/j.braindev.2017.05.009 | Excluded | Lack of relation between the title and the abstract of the article with the subject under study. |
|  | Barani M, Sangiovanni E, Angarano M, Rajizadeh MA, Mehrabani M, Piazza S, et al. Phytosomes as innovative delivery systems for phytochemicals: A comprehensive review of literature. International Journal of Nanomedicine. 2021;16:6983-7022.https://doi.org/10.2147/IJN.S318416 | Excluded | Lack of relation between the title and the abstract of the article with the subject under study. |
|  | Barbagelata E, Nicolini A, Ambrosino I, Politi C. Gender differences and Chronic obstructive pulmonary disease: An update on the literature. Italian Journal of Medicine. 2018;12(3):171-9.https://doi.org/10.4081/itjm.2018.987 | Excluded | Lack of relation between the title and the abstract of the article with the subject under study. |
|  | Barber AT, Loughlin CE. Pediatric Pulmonology 2020 year in review: Asthma. Pediatric Pulmonology. 2021;56(8):2455-9.https://doi.org/10.1002/ppul.25510 | Excluded | Lack of relation between the title and the abstract of the article with the subject under study. |
|  | Bardin PG, Rangaswamy J, Yo SW. Managing comorbid conditions in severe asthma. Medical Journal of Australia. 2018;209(2):S11-S7.e3.https://doi.org/10.5694/mja18.00196 | Excluded | Lack of relation between the title and the abstract of the article with the subject under study. |
|  | Barie PS, Brindle ME, Khadaroo RG, Klassen TL, Huston JM. Omicron, Long-COVID, and the Safety of Elective Surgery for Adults and Children: Joint Guidance from the Therapeutics and Guidelines Committee of the Surgical Infection Society and the Surgery Strategic Clinical Network, Alberta Health Services. Surgical Infections. 2023;24(1):6-18.https://doi.org/10.1089/sur.2022.274 | Excluded | Lack of relation between the title and the abstract of the article with the subject under study. |
|  | Barker N, Thevasagayam R, Ugonna K, Kirkby J. Pediatric Dysfunctional Breathing: Proposed Components, Mechanisms, Diagnosis, and Management. Frontiers in Pediatrics. 2020;8.https://doi.org/10.3389/fped.2020.00379 | Excluded | Lack of relation between the title and the abstract of the article with the subject under study. |
|  | Barker NJ, Jones M, O'Connell NE, Everard ML. Breathing exercises for dysfunctional breathing/hyperventilation syndrome in children. Cochrane Database of Systematic Reviews. 2013;2013(12).https://doi.org/10.1002/14651858.CD010376.pub2 | Excluded | Lack of relation between the title and the abstract of the article with the subject under study. |
|  | Barman A, Sinha MK, Sahoo J, Jena D, Patel V. Respiratory rehabilitation in patients recovering from severe acute respiratory syndrome: A systematic review and meta-analysis. Heart & Lung. 2022;53:11-24.https://doi.org/10.1016/j.hrtlng.2022.01.005 | Excluded | Lack of relation between the title and the abstract of the article with the subject under study. |
|  | Barnabé V, Saraiva B, Stelmach R, Martins MA, Nunes MPT. Chest physiotherapy does not induce bronchospasm in stable asthma. Physiotherapy. 2003;89(12):714-9 | Excluded | Lack of relation between the title and the abstract of the article with the subject under study. |
|  | Barnthouse M, Jones BL. The Impact of Environmental Chronic and Toxic Stress on Asthma. Clinical Reviews in Allergy and Immunology. 2019;57(3):427-38.https://doi.org/10.1007/s12016-019-08736-x | Excluded | Lack of relation between the title and the abstract of the article with the subject under study. |
|  | Barone E, Corrado A, Gemignani F, Landi S. Environmental risk factors for pancreatic cancer: an update. Archives of Toxicology. 2016;90(11):2617-42.https://doi.org/10.1007/s00204-016-1821-9 | Excluded | Lack of relation between the title and the abstract of the article with the subject under study. |
|  | Barradell AC, Gerlis C, Houchen-Wolloff L, Bekker HL, Robertson N, Singh SJ. Systematic review of shared decision-making interventions for people living with chronic respiratory diseases. BMJ Open. 2023;13(5).https://doi.org/10.1136/bmjopen-2022-069461 | Excluded | Lack of relation between the title and the abstract of the article with the subject under study. |
|  | Barrett NA, Arm JP. Is a comorbidity the real problem? Difficult-to-control asthma: Diagnostic dilemmas. Journal of Respiratory Diseases. 2007;28(8):319-24 | Excluded | Lack of relation between the title and the abstract of the article with the subject under study. |
|  | Barua P, O'Mahony MS. Overcoming gaps in the management of asthma in older patients: New insights. Drugs and Aging. 2005;22(12):1029-59.https://doi.org/10.2165/00002512-200522120-00004 | Excluded | Lack of relation between the title and the abstract of the article with the subject under study. |
|  | Basaca DG, Jugănaru I, Belei O, Nicoară DM, Asproniu R, Stoicescu ER, et al. Long COVID in Children and Adolescents: Mechanisms, Symptoms, and Long-Term Impact on Health—A Comprehensive Review. Journal of Clinical Medicine. 2025;14(2).https://doi.org/10.3390/jcm14020378 | Excluded | Lack of relation between the title and the abstract of the article with the subject under study. |
|  | Basch CE. Asthma and the Achievement Gap Among Urban Minority Youth. Journal of School Health. 2011;81(10):606-13.https://doi.org/10.1111/j.1746-1561.2011.00634.x | Excluded | Lack of relation between the title and the abstract of the article with the subject under study. |
|  | Bascom R, Dhingra R, Francomano CA. Respiratory manifestations in the Ehlers–Danlos syndromes. American Journal of Medical Genetics, Part C: Seminars in Medical Genetics. 2021;187(4):533-48.https://doi.org/10.1002/ajmg.c.31953 | Excluded | Lack of relation between the title and the abstract of the article with the subject under study. |
|  | Bastas D, Brandão LR, Vincelli J, Schneiderman JE, Cunningham J, Avila ML. Physical activity for children with deep vein thrombosis and pulmonary embolism on anticoagulation: a scoping review. Research and Practice in Thrombosis and Haemostasis. 2023;7(2).https://doi.org/10.1016/j.rpth.2023.100094 | Excluded | Lack of relation between the title and the abstract of the article with the subject under study. |
|  | Batozhargalova B, Mizernitskiy Y. Meta-analysis of the prevalence of asthma and asthma symptoms in Russia. European Respiratory Journal. 2020;56.https://doi.org/10.1183/13993003.congress-2020.3499 | Excluded | Lack of relation between the title and the abstract of the article with the subject under study. |
|  | Batra V, Niazi S, Peters SP. Persistent asthma: What approach is best? Journal of Respiratory Diseases. 2002;23(6):330-40 | Excluded | Lack of relation between the title and the abstract of the article with the subject under study. |
|  | Bauer A, Dickel H, Jakob T, Kleinheinz A, Lippert U, Metz M, et al. Expert consensus on practical aspects in the treatment of chronic urticaria. Allergo Journal International. 2021;30(2):64-75.https://doi.org/10.1007/s40629-021-00162-w | Excluded | Lack of relation between the title and the abstract of the article with the subject under study. |
|  | Bauman AE, Fardy HJ, Harris PG. Getting it right: why bother with patient-centred care? Medical Journal of Australia. 2003;179(5):253-6.https://doi.org/10.5694/j.1326-5377.2003.tb05532.x | Excluded | Lack of relation between the title and the abstract of the article with the subject under study. |
|  | Baumeister H, Hutter N, Bengel J, Haerter M. Quality of Life in Medically Ill Persons with Comorbid Mental Disorders: A Systematic Review and Meta-Analysis. Psychotherapy and Psychosomatics. 2011;80(5):275-86.https://doi.org/10.1159/000323404 | Excluded | Lack of relation between the title and the abstract of the article with the subject under study. |
|  | Baumeister SE, Finger JD, Gläser S, Dörr M, Markus MR, Ewert R, et al. Alcohol consumption, smoking and cardiorespiratory fitness: Findings from four population-based studies. European Journal of Epidemiology. 2016;31:S21.https://doi.org/10.1007/s10654-016-0183-1 | Excluded | Lack of relation between the title and the abstract of the article with the subject under study. |
|  | Bazi T, Takahashi S, Ismail S, Bø K, Ruiz-Zapata AM, Duckett J, et al. Prevention of pelvic floor disorders: international urogynecological association research and development committee opinion. International Urogynecology Journal. 2016;27(12):1785-95.https://doi.org/10.1007/s00192-016-2993-9 | Excluded | Lack of relation between the title and the abstract of the article with the subject under study. |
|  | Beamon SP, Falkenbach A, Fainburg G, Linde K. Speleotherapy for asthma. Cochrane Database of Systematic Reviews. 2001(2).https://doi.org/10.1002/14651858.CD001741 | Excluded | Lack of relation between the title and the abstract of the article with the subject under study. |
|  | Becker TK, Tafoya CA, Osei-Ampofo M, Tafoya MJ, Kessler RA, Theyyunni N, et al. Cardiopulmonary ultrasound for critically ill adults improves diagnostic accuracy in a resource-limited setting: the AFRICA trial. Tropical Medicine and International Health. 2017;22(12):1599-608.https://doi.org/10.1111/tmi.12992 | Excluded | Lack of relation between the title and the abstract of the article with the subject under study. |
|  | Behbehani N, FitzGerald JM. The assessment and management of patients with acute asthma. International Journal of Tuberculosis and Lung Disease. 2006;10(4):356-64 | Excluded | Lack of relation between the title and the abstract of the article with the subject under study. |
|  | Beigoli S, Amin F, Kazemi Rad H, Rezaee R, Boskabady MH. Occupational respiratory disorders in Iran: a review of prevalence and inducers. Frontiers in Medicine. 2024;11.https://doi.org/10.3389/fmed.2024.1310040 | Excluded | Lack of relation between the title and the abstract of the article with the subject under study. |
|  | Bel E, Ten Brinke A. A rational approach to the management of severe refractory asthma. Treatments in Respiratory Medicine. 2005;4(6):365-79.https://doi.org/10.2165/00151829-200504060-00002 | Excluded | Lack of relation between the title and the abstract of the article with the subject under study. |
|  | Belkin A, Swigris JJ. Patient expectations and experiences in idiopathic pulmonary fibrosis: Implications of patient surveys for improved care. Expert Review of Respiratory Medicine. 2014;8(2):173-8.https://doi.org/10.1586/17476348.2014.880056 | Excluded | Lack of relation between the title and the abstract of the article with the subject under study. |
|  | Bellanti JA. Literature review: The best new articles in the specialty of allergy, asthma, and immunology, 2004-2005. Allergy and Asthma Proceedings. 2006;27(3):186-96.https://doi.org/10.2500/aap.2006.27.2859 | Excluded | Lack of relation between the title and the abstract of the article with the subject under study. |
|  | Belyh NA, Piznyur IV. Obesity-associated asthma phenotype in children. Profilakticheskaya Meditsina. 2024;27(1):106-14.https://doi.org/10.17116/profmed202427011106 | Excluded | Lack of relation between the title and the abstract of the article with the subject under study. |
|  | Bennett S, Shafran R, Coughtrey A, Walker S, Heyman I. Psychological interventions for mental health disorders in children with chronic physical illness: A systematic review. Archives of Disease in Childhood. 2015;100(4):308-16.https://doi.org/10.1136/archdischild-2014-307474 | Excluded | Lack of relation between the title and the abstract of the article with the subject under study. |
|  | Bentley S, Morgan L, Exall E, Arbuckle R, Rossom RC, Roche N, et al. Qualitative Interviews to Support Development and Cognitive Debriefing of the Adelphi Adherence Questionnaire (ADAQ©): A Patient-Reported Measure of Medication Adherence Developed for Use in a Range of Diseases, Treatment Modalities, and Countries. Patient Preference and Adherence. 2022;16:2579-92.https://doi.org/10.2147/PPA.S358046 | Excluded | Lack of relation between the title and the abstract of the article with the subject under study. |
|  | Bentley TGK, D’Andrea-Penna G, Rakic M, Arce N, LaFaille M, Berman R, et al. Breathing Practices for Stress and Anxiety Reduction: Conceptual Framework of Implementation Guidelines Based on a Systematic Review of the Published Literature. Brain Sciences. 2023;13(12).https://doi.org/10.3390/brainsci13121612 | Excluded | Lack of relation between the title and the abstract of the article with the subject under study. |
|  | Bernard A. Swimming attendance during childhood and development of asthma: Meta-analysis. Pediatrics International. 2017;59(7):846-7.https://doi.org/10.1111/ped.13276 | Excluded | Lack of relation between the title and the abstract of the article with the subject under study. |
|  | Bertels X, Edris A, Garcia-Aymerich J, Faner R, Meteran H, Sigsgaard T, et al. Phenotyping asthma with airflow obstruction in middle-aged and older adults: a CADSET clinical research collaboration. Bmj Open Respiratory Research. 2023;10(1).https://doi.org/10.1136/bmjresp-2023-001760 | Excluded | Lack of relation between the title and the abstract of the article with the subject under study. |
|  | Bertlich M, Freytag S, Dombrowski T, Jurmeister P, Spiegel JL, Bertlich I, et al. Subgroups in the treatment of nasal polyposis with dupilumab: A retrospective study. Medicine (United States). 2022;101(45):E31031.https://doi.org/10.1097/MD.0000000000031031 | Excluded | Lack of relation between the title and the abstract of the article with the subject under study. |
|  | Bertrand C, Tschirhart E. Epithelial factors: modulation of the airway smooth muscle tone. Fundamental & Clinical Pharmacology. 1993;7(6):261-73.https://doi.org/10.1111/j.1472-8206.1993.tb00240.x | Excluded | Lack of relation between the title and the abstract of the article with the subject under study. |
|  | Besag FMC, Vasey MJ, Lao KSJ, Wong ICK. Adverse Events Associated with Melatonin for the Treatment of Primary or Secondary Sleep Disorders: A Systematic Review. CNS Drugs. 2019;33(12):1167-86.https://doi.org/10.1007/s40263-019-00680-w | Excluded | Lack of relation between the title and the abstract of the article with the subject under study. |
|  | Beyens A, Van Meensel K, Pottie L, De Rycke R, De Bruyne M, Baeke F, et al. Defining the clinical, molecular and ultrastructural characteristics in occipital horn syndrome: Two new cases and review of the literature. Genes. 2019;10(7).https://doi.org/10.3390/genes10070528 | Excluded | Lack of relation between the title and the abstract of the article with the subject under study. |
|  | Beynon AM, Hebert JJ, Lebouef-Yde C, Walker BF. Potential risk factors and triggers for back pain in children and young adults. A scoping review, part II: Unclear or mixed types of back pain. Chiropractic and Manual Therapies. 2019;27(1).https://doi.org/10.1186/s12998-019-0281-8 | Excluded | Lack of relation between the title and the abstract of the article with the subject under study. |
|  | Bhagra A, Tierney DM, Sekiguchi H, Soni NJ. Point-of-Care Ultrasonography for Primary Care Physicians and General Internists. Mayo Clinic Proceedings. 2016;91(12):1811-27.https://doi.org/10.1016/j.mayocp.2016.08.023 | Excluded | Lack of relation between the title and the abstract of the article with the subject under study. |
|  | Bhalla A, Bhalla R, Ganta S. Working remotely: a perspective on telemedicine in delivery of obstetrics and gynaecology health care. Obstetrician and Gynaecologist. 2021;23(4):237-42.https://doi.org/10.1111/tog.12763 | Excluded | Lack of relation between the title and the abstract of the article with the subject under study. |
|  | Bhattacharya U, Dutta A. Efficacy of Advanced Allied Interventions for Dyspnoea, Exercise Capacity, and Quality of Life among the Geriatric Population: A Literature Review. Journal of Clinical and Diagnostic Research. 2024;18(4):YE05-YE8.https://doi.org/10.7860/JCDR/2024/68354.19276 | Excluded | Lack of relation between the title and the abstract of the article with the subject under study. |
|  | Bhogal S, Zemek R, Ducharme FM. Written action plans for asthma in children (Cochrane review) [with consumer summary]. Cochrane Database of Systematic Reviews 2006;Issue 3. 2006 | Excluded | Lack of relation between the title and the abstract of the article with the subject under study. |
|  | Bhutani M, Hernandez P, Bourbeau J, Dechman G, Penz E, Aceron R, et al. Key Highlights of the Canadian Thoracic Society's Position Statement on the Optimization of COPD Management During the Coronavirus Disease 2019 Pandemic. Chest. 2020;158(3):869-72.https://doi.org/10.1016/j.chest.2020.05.530 | Excluded | Lack of relation between the title and the abstract of the article with the subject under study. |
|  | Bickton F, Fombe C, Chisati E, Rylance J. Evidence for pulmonary rehabilitation in chronic respiratory diseases in sub-Saharan Africa: a systematic review. European Respiratory Journal. 2020;56.https://doi.org/10.1183/13993003.congress-2020.845 | Excluded | Lack of relation between the title and the abstract of the article with the subject under study. |
|  | Bidell MR, Lodise TP. Fluoroquinolone-Associated Tendinopathy: Does Levofloxacin Pose the Greatest Risk? Pharmacotherapy. 2016;36(6):679-93.https://doi.org/10.1002/phar.1761 | Excluded | Lack of relation between the title and the abstract of the article with the subject under study. |
|  | Biedrzycki G, Wolszczak-Biedrzycka B, Dorf J, Maciejczyk M. The antioxidant barrier, oxidative/nitrosative stress, and protein glycation in allergy: from basic research to clinical practice. Frontiers in Immunology. 2024;15.https://doi.org/10.3389/fimmu.2024.1440313 | Excluded | Lack of relation between the title and the abstract of the article with the subject under study. |
|  | Bielory L, Russin J, Zuckerman GB. Clinical efficacy, mechanisms of action, and adverse effects of complementary and alternative medicine therapies for asthma. Allergy and Asthma Proceedings. 2004;25(5):283-91 | Excluded | Lack of relation between the title and the abstract of the article with the subject under study. |
|  | Billich N, Maugeri I, Calligaro L, Davidson Z. The impact of interventions including a dietary component in overweight and obese children and adolescents with chronic diseases: a systematic review and meta-analysis. Obesity Research and Clinical Practice. 2019;13(3):315.https://doi.org/10.1016/j.orcp.2018.11.219 | Excluded | Lack of relation between the title and the abstract of the article with the subject under study. |
|  | Binetti M, Lauro A, Golfieri R, Vaccari S, D’Andrea V, Marino IR, et al. False in Name Only—Gastroduodenal Artery Pseudoaneurysm in a Recurrently Bleeding Patient: Case Report and Literature Review. Digestive Diseases and Sciences. 2019;64(11):3086-91.https://doi.org/10.1007/s10620-019-05853-7 | Excluded | Lack of relation between the title and the abstract of the article with the subject under study. |
|  | Bisgaard H. Long-acting beta(2)-agonists in management of childhood asthma: A critical review of the literature. Pediatr Pulmonol. 2000;29(3):221-34.https://doi.org/10.1002/(sici)1099-0496(200003)29:3<221::aid-ppul11>3.0.co;2-p | Excluded | Lack of relation between the title and the abstract of the article with the subject under study. |
|  | Blackhall K, Appleton S, Cates CJ. Ionisers for chronic asthma. Cochrane database of systematic reviews (Online). 2003(3):CD002986 | Excluded | Lack of relation between the title and the abstract of the article with the subject under study. |
|  | Blackhall K, Appleton S, Cates CJ. Ionisers for chronic asthma. Cochrane Database of Systematic Reviews. 2012;2017(9).https://doi.org/10.1002/14651858.CD002986.pub2 | Excluded | Lack of relation between the title and the abstract of the article with the subject under study. |
|  | Blaiss M. Current concepts and therapeutic strategies forallergic rhinitis in school-age children. Clinical Therapeutics. 2004;26(11):1876-89.https://doi.org/10.1016/j.clinthera.2004.11.003 | Excluded | Lack of relation between the title and the abstract of the article with the subject under study. |
|  | Blase K, Vermetten E, Lehrer P, Gevirtz R. Neurophysiological Approach by Self-Control of Your Stress-Related Autonomic Nervous System with Depression, Stress and Anxiety Patients. International Journal of Environmental Research and Public Health. 2021;18(7).https://doi.org/10.3390/ijerph18073329 | Excluded | Lack of relation between the title and the abstract of the article with the subject under study. |
|  | Boland MRS, Tsiachristas A, Kruis A, Chavannes N, Rutten-Van Mölken MPMH. Are disease management programs for COPD cost-saving? Value in Health. 2012;15(7):A566.https://doi.org/10.1016/j.jval.2012.08.2039 | Excluded | Lack of relation between the title and the abstract of the article with the subject under study. |
|  | Boling PA. Preface. Clinics in Geriatric Medicine. 2009;25(1):xi-xiii.https://doi.org/10.1016/j.cger.2008.12.001 | Excluded | Lack of relation between the title and the abstract of the article with the subject under study. |
|  | Bolton CE, Bevan-Smith EF, Blakey JD, Crowe P, Elkin SL, Garrod R, et al. British Thoracic Society guideline on pulmonary rehabilitation in adults. Thorax. 2013;68(SUPPL. 2):ii1-ii30.https://doi.org/10.1136/thoraxjnl-2013-203808 | Excluded | Lack of relation between the title and the abstract of the article with the subject under study. |
|  | Bonikowski M, Sławek J. Safety and efficacy of botulinum toxin type-A preparations in cerebral palsy — An evidence-based review. Neurologia i Neurochirurgia Polska. 2021;55(2):158-64.https://doi.org/10.5603/PJNNS.A2021.0032 | Excluded | Lack of relation between the title and the abstract of the article with the subject under study. |
|  | Bonini M, Di Paolo M, Bagnasco D, Baiardini I, Braido F, Caminati M, et al. Minimal clinically important difference for asthma endpoints: An expert consensus report. European Respiratory Review. 2020;29(156):1-14.https://doi.org/10.1183/16000617.0137-2019 | Excluded | Lack of relation between the title and the abstract of the article with the subject under study. |
|  | Booms CZC, Barbee MGA. Spontaneous pneumomediastinum in a pediatric patient after a 1600-m run: Case report and literature review. Journal of the American Osteopathic Association. 2015;115(5):338-41.https://doi.org/10.7556/jaoa.2015.065 | Excluded | Lack of relation between the title and the abstract of the article with the subject under study. |
|  | Boonpiyathad S, Sangasapaviliya A. Refractory asthma treatment is complicated by tracheobronchomalacia: Case reports and review of the literature. Case Reports in Medicine. 2013;2013.https://doi.org/10.1155/2013/735058 | Excluded | Lack of relation between the title and the abstract of the article with the subject under study. |
|  | Borak J, Lefkowitz RY. Bronchial hyperresponsiveness. Occupational Medicine-Oxford. 2016;66(2):95-105.https://doi.org/10.1093/occmed/kqv158 | Excluded | Lack of relation between the title and the abstract of the article with the subject under study. |
|  | Borke ME, Nwagu MU, Obaseki D, Bazuaye NO. Churg Strauss syndrome: a review. Nigerian journal of medicine : journal of the National Association of Resident Doctors of Nigeria. 2010;19(2):136-9.https://doi.org/10.4314/njm.v19i2.56499 | Excluded | Lack of relation between the title and the abstract of the article with the subject under study. |
|  | Born CDC, Bhadra R, D'Souza G, Kremers SPJ, Sambashivaiah S, Schols AMWJ, et al. Combined Lifestyle Interventions in the Prevention and Management of Asthma and COPD: A Systematic Review. Nutrients. 2024;16(10).https://doi.org/10.3390/nu16101515 | Excluded | Lack of relation between the title and the abstract of the article with the subject under study. |
|  | Bouaziz H, Charfî N, Kaffel N, Mnif M, Abid M. Rare complication of diabetic acidoketosis: The pneumomediastinum. Revue de Pneumologie Clinique. 2007;63(5):327-30.https://doi.org/10.1016/S0761-8417(07)74211-6 | Excluded | Lack of relation between the title and the abstract of the article with the subject under study. |
|  | Bougault V, Adami PE, Sewry N, Fitch K, Carlsten C, Villiger B, et al. Environmental factors associated with non-infective acute respiratory illness in athletes: A systematic review by a subgroup of the IOC consensus group on "acute respiratory illness in the athlete". Journal of Science and Medicine in Sport. 2022;25(6):466-73.https://doi.org/10.1016/j.jsams.2022.03.003 | Excluded | Lack of relation between the title and the abstract of the article with the subject under study. |
|  | Bougault V, Turmel J, Levesque B, Boulet L. The respiratory health of swimmers. Sports Medicine. 2009;39(4):295-312.https://doi.org/10.2165/00007256-200939040-00003 | Excluded | Lack of relation between the title and the abstract of the article with the subject under study. |
|  | Boulet LP, Turmel J. Cough in exercise and athletes. Pulmonary Pharmacology and Therapeutics. 2019;55:67-74.https://doi.org/10.1016/j.pupt.2019.02.003 | Excluded | Lack of relation between the title and the abstract of the article with the subject under study. |
|  | Boulet L-P, Turmel J, Irwin RS, Panel CEC. Cough in the Athlete: CHEST Guideline and Expert Panel Report. CHEST. 2017;151(2):441-54.https://doi.org/10.1016/j.chest.2016.10.054 | Excluded | Lack of relation between the title and the abstract of the article with the subject under study. |
|  | Bourbeau J, Bhutani M, Hernandez P, Marciniuk DD, Aaron SD, Balter M, et al. CTS position statement: Pharmacotherapy in patients with COPD—An update. Canadian Journal of Respiratory, Critical Care, and Sleep Medicine. 2017;1(4):222-41.https://doi.org/10.1080/24745332.2017.1395588 | Excluded | Lack of relation between the title and the abstract of the article with the subject under study. |
|  | Bourbeau J, Marciniuk J. Non-Pharmacological Treatments of Asthma Chronic Obstructive Pulmonary Disease Overlap and Rehabilitation Programs. Immunology and Allergy Clinics of North America. 2022;42(3):e1-e12.https://doi.org/10.1016/j.iac.2023.05.002 | Excluded | Lack of relation between the title and the abstract of the article with the subject under study. |
|  | Bousquet J, Burney PG, Zuberbier T, v. Cauwenberge P, Akdis CA, Bindslev-Jensen C, et al. GA<SUP>2</SUP>LEN (Global Allergy and Asthma European Network) addresses the allergy and asthma 'epidemic'. Allergy. 2009;64(7):969-77.https://doi.org/10.1111/j.1398-9995.2009.02059.x | Excluded | Lack of relation between the title and the abstract of the article with the subject under study. |
|  | Bousquet J, Gern JE, Martinez FD, Anto JM, Johnson CC, Holt PG, et al. Birth cohorts in asthma and allergic diseases: Report of a NIAID/NHLBI/MeDALL joint workshop. Journal of Allergy and Clinical Immunology. 2014;133(6):1535-46.https://doi.org/10.1016/j.jaci.2014.01.018 | Excluded | Lack of relation between the title and the abstract of the article with the subject under study. |
|  | Boutayeb A, Boutayeb S, Boutayeb W. Multi-morbidity of non communicable diseases and equity in WHO Eastern Mediterranean countries. International Journal for Equity in Health. 2013;12(1).https://doi.org/10.1186/1475-9276-12-60 | Excluded | Lack of relation between the title and the abstract of the article with the subject under study. |
|  | Boutayeb A, Boutayeb S, Boutayeb W. Multi-morbidity of non communicable diseases and equity in WHO Eastern Mediterranean countries. International Journal for Equity in Health. 2015;12(1).https://doi.org/10.1186/1475-9276-12-60 | Excluded | Lack of relation between the title and the abstract of the article with the subject under study. |
|  | Boyce JA, Assa'ad A, Burks AW, Jones SM, Sampson HA, Wood RA, et al. Guidelines for the Diagnosis and Management of Food Allergy in the United States: Summary of the NIAID-Sponsored Expert Panel Report. Nutrition Research. 2011;31(1):61-75.https://doi.org/10.1016/j.nutres.2011.01.001 | Excluded | Lack of relation between the title and the abstract of the article with the subject under study. |
|  | Bradfield JP, Taal HR, Timpson NJ, Scherag A, Lecoeur C, Warrington NM, et al. A genome-wide association meta-analysis identifies new childhood obesity loci. Nature Genetics. 2012;44(5):526-31.https://doi.org/10.1038/ng.2247 | Excluded | Lack of relation between the title and the abstract of the article with the subject under study. |
|  | Bradfield JP, Vogelezang S, Felix JF, Chesi A, Helgeland Ø, Horikoshi M, et al. A trans-ancestral meta-analysis of genome-wide association studies reveals loci associated with childhood obesity. Human Molecular Genetics. 2019;28(19):3327-38.https://doi.org/10.1093/hmg/ddz161 | Excluded | Lack of relation between the title and the abstract of the article with the subject under study. |
|  | Bradford C, Martin D, Loughran K, Robertson N, Carne A, Skidmore N, et al. The impact of sport on the physical, psychological and social wellbeing of people with chronic breathlessness: A mixed-methods systematic review. Clinical Rehabilitation. 2023;37(12):1611-36.https://doi.org/10.1177/02692155231190770 | Excluded | Lack of relation between the title and the abstract of the article with the subject under study. |
|  | Bradicich M, Schuurmans MM. Smoking status and second-hand smoke biomarkers in COPD, asthma and healthy controls. ERJ Open Research. 2020;6(2):1-7.https://doi.org/10.1183/23120541.00192-2019 | Excluded | Lack of relation between the title and the abstract of the article with the subject under study. |
|  | Braegger C, Campoy C, Colomb V, Decsi T, Domellof M, Fewtrell M, et al. Vitamin d in the healthy European paediatric population. Journal of Pediatric Gastroenterology and Nutrition. 2013;56(6):692-701.https://doi.org/10.1097/MPG.0b013e31828f3c05 | Excluded | Lack of relation between the title and the abstract of the article with the subject under study. |
|  | Brett S, Reay H. The James lind alliance intensive care research priority setting partnership: Why another research prioritisation exercise?! Journal of the Intensive Care Society. 2013;14(3):204.https://doi.org/10.1177/175114371301400305 | Excluded | Lack of relation between the title and the abstract of the article with the subject under study. |
|  | Brigden A, Parslow RM, Linney C, Higson-Sweeney N, Read R, Loades M, et al. How are behavioural interventions delivered to children (5-11 years old): a systematic mapping review. Bmj Paediatrics Open. 2019;3(1).https://doi.org/10.1136/bmjpo-2019-000543 | Excluded | Lack of relation between the title and the abstract of the article with the subject under study. |
|  | Brigham EP, West NE. Diagnosis of asthma: Diagnostic testing. International Forum of Allergy and Rhinology. 2015;5:S27-S30.https://doi.org/10.1002/alr.21597 | Excluded | Lack of relation between the title and the abstract of the article with the subject under study. |
|  | Brissot R, Gonzalez-Bermejo J, Lassalle A, Desrues B, Doutrellot PL. Fatigue and respiratory disorders. Annales de Readaptation et de Medecine Physique. 2006;49(6):403-12.https://doi.org/10.1016/j.annrmp.2006.04.008 | Excluded | Lack of relation between the title and the abstract of the article with the subject under study. |
|  | Bronfort G, Haas M, Evans R, Leininger B, Triano J. Effectiveness of manual therapies: the UK evidence report. Chiropractic & Osteopathy 2010 Feb 25;18(3):Epub. 2010 | Excluded | Lack of relation between the title and the abstract of the article with the subject under study. |
|  | Brown ES, Varghese FP, McEwen BS. Association of depression with medical illness: Does cortisol play a role? Biological Psychiatry. 2004;55(1):1-9.https://doi.org/10.1016/S0006-3223(03)00473-6 | Excluded | Lack of relation between the title and the abstract of the article with the subject under study. |
|  | Brown JS. Nitrogen dioxide exposure and airway responsiveness in individuals with asthma. Inhalation Toxicology. 2015;27(1):1-14.https://doi.org/10.3109/08958378.2014.979960 | Excluded | Lack of relation between the title and the abstract of the article with the subject under study. |
|  | Brown SD, Calvert HH, Fitzpatrick AM. Vitamin D and asthma. Dermato-Endocrinology. 2012;4(2):137-45.https://doi.org/10.4161/derm.20434 | Excluded | Lack of relation between the title and the abstract of the article with the subject under study. |
|  | Brown SGA. Anaphylaxis: Clinical concepts and research priorities. EMA - Emergency Medicine Australasia. 2006;18(2):155-69.https://doi.org/10.1111/j.1742-6723.2006.00831.x | Excluded | Lack of relation between the title and the abstract of the article with the subject under study. |
|  | Brown WM. Cilomilast GlaxoSmithKline. Current Opinion in Investigational Drugs. 2005;6(5):545-58 | Excluded | Lack of relation between the title and the abstract of the article with the subject under study. |
|  | Brusselle GG, Gaga M. ERS guidelines, statements and technical standards published in the ERJ in 2014: A year in review. European Respiratory Journal. 2015;45(4):863-6.https://doi.org/10.1183/09031936.00238514 | Excluded | Lack of relation between the title and the abstract of the article with the subject under study. |
|  | Buhi ER, Trudnak TE, Martinasek MP, Oberne AB, Fuhrmann HJ, McDermott RJ. Mobile phone-based behavioural interventions for health: A systematic review. Health Education Journal. 2013;72(5):564-83.https://doi.org/10.1177/0017896912452071 | Excluded | Lack of relation between the title and the abstract of the article with the subject under study. |
|  | Buist M. Micromassage: The round-headed needle. Journal of Chinese Medicine. 2017;2017-June(114):69-79 | Excluded | Lack of relation between the title and the abstract of the article with the subject under study. |
|  | Bukowski JA, Lewis RJ, Gamble JF, Wojcik NC, Laumbach RJ. Range-finding study of risk factors for childhood asthma development and national asthma prevalence. Human and Ecological Risk Assessment. 2002;8(4):735-65.https://doi.org/10.1080/10807030290879925 | Excluded | Lack of relation between the title and the abstract of the article with the subject under study. |
|  | Burge AT, Gadowski AM, Jones A, Romero L, Smallwood NE, Ekström M, et al. Breathing techniques to reduce symptoms in people with serious respiratory illness: a systematic review. European Respiratory Review. 2024;33(174).https://doi.org/10.1183/16000617.0012-2024 | Excluded | Lack of relation between the title and the abstract of the article with the subject under study. |
|  | Burns J, Boogaard H, Polus S, Pfadenhauer LM, Rohwer AC, van Erp AM, et al. Interventions to reduce ambient particulate matter air pollution and their effect on health. Cochrane Database of Systematic Reviews. 2019(5).https://doi.org/10.1002/14651858.CD010919.pub2 | Excluded | Lack of relation between the title and the abstract of the article with the subject under study. |
|  | Burtscher M, Mairer K, Wille M, Gatterer H, Ruedl G, Faulhaber M, et al. Short-term exposure to hypoxia for work and leisure activities in health and disease: Which level of hypoxia is safe? Sleep and Breathing. 2012;16(2):435-42.https://doi.org/10.1007/s11325-011-0521-1 | Excluded | Lack of relation between the title and the abstract of the article with the subject under study. |
|  | Bush A. Asthma inflammometry assessment improves asthma care-Con. 2011. p. S13-S5 | Excluded | Lack of relation between the title and the abstract of the article with the subject under study. |
|  | Bush A. How should we manage asthma in preschoolers-from guidelines to consensus. Paediatric Respiratory Reviews. 2012;13:S6-S8 | Excluded | Lack of relation between the title and the abstract of the article with the subject under study. |
|  | Busquets RM, Antó JM, Sunyer J, Sancho N, Vall O. Prevalence of asthma-related symptoms and bronchial responsiveness to exercise in children aged 13-14 yrs in Barcelona, Spain. European Respiratory Journal. 1996;9(10):2094-8.https://doi.org/10.1183/09031936.96.09102094 | Excluded | Lack of relation between the title and the abstract of the article with the subject under study. |
|  | Butler SJ, Lee AL, Goldstein RS, Brooks D. Active video games as a training tool for individuals with chronic respiratory disease: A systematic review. Canadian Journal of Respiratory Critical Care and Sleep Medicine. 2017;1(2):113.https://doi.org/10.1080/24745332.2017.1332400 | Excluded | Lack of relation between the title and the abstract of the article with the subject under study. |
|  | Butler SJ, Lee AL, Goldstein RS, Brooks D. Active Video Games as a Training Tool for Individuals With Chronic Respiratory Diseases: A SYSTEMATIC REVIEW. Journal of Cardiopulmonary Rehabilitation and Prevention. 2019;39(2):85-90.https://doi.org/10.1097/HCR.0000000000000320 | Excluded | Lack of relation between the title and the abstract of the article with the subject under study. |
|  | Buttery SC, Lewis A, Kemp SV, Banya W, Quint JK, Steiner MC, et al. Lung volume reduction eligibility in patients with COPD completing pulmonary rehabilitation: Results from the UK National Asthma and COPD Audit Programme. BMJ Open. 2020;10(11).https://doi.org/10.1136/bmjopen-2020-040942 | Excluded | Lack of relation between the title and the abstract of the article with the subject under study. |
|  | Byeman CJ, Harshman LA, Engen RM. Adult and late adolescent complications of pediatric solid organ transplantation. Pediatric Transplantation. 2024;28(4).https://doi.org/10.1111/petr.14766 | Excluded | Lack of relation between the title and the abstract of the article with the subject under study. |
|  | Cabra HA, Zanela OO, Anaya P, Rodriguez S. Economic evaluation of bariatric surgery as treatment for obesity and associated comorbidities-estimation by discrete event simulation. Value in Health. 2009;12(7):A491 | Excluded | Lack of relation between the title and the abstract of the article with the subject under study. |
|  | Cabrera Martimbianco AL, Pacheco RL, Bagattini ÂM, Riera R. Frequency, signs and symptoms, and criteria adopted for long COVID-19: A systematic review. International Journal of Clinical Practice. 2021;75(10).https://doi.org/10.1111/ijcp.14357 | Excluded | Lack of relation between the title and the abstract of the article with the subject under study. |
|  | Cai H, Li G, Hua S, Liu Y, Chen L. Effect of exercise on cognitive function in chronic disease patients: A meta-analysis and systematic review of randomized controlled trials. Clinical Interventions in Aging. 2017;12:773-83.https://doi.org/10.2147/CIA.S135700 | Excluded | Lack of relation between the title and the abstract of the article with the subject under study. |
|  | Cain H. Bronchoprovocation testing. Clinics in Chest Medicine. 2001;22(4):651-9.https://doi.org/10.1016/S0272-5231(05)70058-7 | Excluded | Lack of relation between the title and the abstract of the article with the subject under study. |
|  | Caldirola D, Perna G. Toward a personalized therapy for panic disorder: Preliminary considerations from a work in progress. Neuropsychiatric Disease and Treatment. 2019;15:1957-70.https://doi.org/10.2147/NDT.S174433 | Excluded | Lack of relation between the title and the abstract of the article with the subject under study. |
|  | Caldwell C, Victoria HK. Breathwork in body psychotherapy: Towards a more unified theory and practice. Body, Movement and Dance in Psychotherapy. 2011;6(2):89-101.https://doi.org/10.1080/17432979.2011.574505 | Excluded | Lack of relation between the title and the abstract of the article with the subject under study. |
|  | Calhoun WJ. Heterogeneity of response to therapy. Advances in Experimental Medicine and Biology. 2014;795:117-22.https://doi.org/10.1007/978-1-4614-8603-9_8 | Excluded | Lack of relation between the title and the abstract of the article with the subject under study. |
|  | Calzetta L, Di Daniele N, Chetta A, Vitale M, Gholamalishahi S, Cazzola M, et al. The Impact of Thermal Water in Asthma and COPD: A Systematic Review According to the PRISMA Statement. Journal of Clinical Medicine. 2024;13(4).https://doi.org/10.3390/jcm13041071 | Excluded | Lack of relation between the title and the abstract of the article with the subject under study. |
|  | Cambach W, Wagenaar RC, Koelman TW, Van Keimpema ARJT, Kemper HCG. The long-term effects of pulmonary rehabilitation in patients with asthma and chronic obstructive pulmonary disease: A research synthesis. Archives of Physical Medicine and Rehabilitation. 1999;80(1):103-11.https://doi.org/10.1016/S0003-9993(99)90316-7 | Excluded | Lack of relation between the title and the abstract of the article with the subject under study. |
|  | Campbell SM, Roland MO, Middleton E, Reeves D. Improvements in quality of clinical care in English general practice 1998-2003: Longitudinal observational study. British Medical Journal. 2005;331(7525):1121-3.https://doi.org/10.1136/bmj.38632.611123.AE | Excluded | Lack of relation between the title and the abstract of the article with the subject under study. |
|  | Cancer-Perez S, Alfayate-García J, Vicente-Jiménez S, Ruiz-Muñoz M, Dhimes-Tejada FP, Gutiérrez-Baz M, et al. Symptomatic Common Carotid Free-Floating Thrombus in a COVID-19 Patient, Case Report and Literature Review. Annals of Vascular Surgery. 2021;73:122-8.https://doi.org/10.1016/j.avsg.2021.02.008 | Excluded | Lack of relation between the title and the abstract of the article with the subject under study. |
|  | Canepa M, Franssen FME, Olschewski H, Lainscak M, Böhm M, Tavazzi L, et al. Diagnostic and Therapeutic Gaps in Patients With Heart Failure and Chronic Obstructive Pulmonary Disease. JACC: Heart Failure. 2019;7(10):823-33.https://doi.org/10.1016/j.jchf.2019.05.009 | Excluded | Lack of relation between the title and the abstract of the article with the subject under study. |
|  | Cano-De La Cuerda R, Useros-Olmo AI, Muñoz-Hellín E. Effectiveness of therapeutic education and respiratory rehabilitation programs for the patient with asthma. Archivos de Bronconeumologia. 2010;46(11):600-6.https://doi.org/10.1016/j.arbres.2010.07.003 | Excluded | Lack of relation between the title and the abstract of the article with the subject under study. |
|  | Cao H, Han M, Li X, Dong S, Shang Y, Wang Q, et al. Clinical research evidence of cupping therapy in China: a systematic literature review. BMC Complementary and Alternative Medicine 2010 Nov 16;10(70):Epub. 2010 | Excluded | Lack of relation between the title and the abstract of the article with the subject under study. |
|  | Carlisle AJ, Sharp NCC. Exercise and outdoor ambient air pollution. British Journal of Sports Medicine. 2001;35(4):214-22.https://doi.org/10.1136/bjsm.35.4.214 | Excluded | Lack of relation between the title and the abstract of the article with the subject under study. |
|  | Carlos E, Rodriguez M, Sossa-Briceno MP, Castro-Rodriguez JA. Factors predicting persistence of early wheezing through childhood and adolescence: a systematic review of the literature. Journal of Asthma and Allergy. 2017;10:83-98.https://doi.org/10.2147/jaa.S128319 | Excluded | Lack of relation between the title and the abstract of the article with the subject under study. |
|  | Carlsen KH. Physical activity and airways diseases, asthma and allergy. Tidsskrift for den Norske Laegeforening. 2000;120(27):3305-9 | Excluded | Lack of relation between the title and the abstract of the article with the subject under study. |
|  | Carregã M, Sousa P, Rocha G, Ferreira-Magalhães M, Azevedo I. Respiratory and non-respiratory outcomes of bronchopulmonary dysplasia in adolescents: A systematic review. Early Human Development. 2023;180.https://doi.org/10.1016/j.earlhumdev.2023.105756 | Excluded | Lack of relation between the title and the abstract of the article with the subject under study. |
|  | Carroll CL, Sala KA. Pediatric Status Asthmaticus. Critical Care Clinics. 2013;29(2):153-66.https://doi.org/10.1016/j.ccc.2012.12.001 | Excluded | Lack of relation between the title and the abstract of the article with the subject under study. |
|  | Carsin AE, Fuertes E, Schaffner E, Jarvis D, Antó JM, Heinrich J, et al. Restrictive spirometry pattern is associated with low physical activity levels. A population based international study. Respiratory Medicine. 2019;146:116-23.https://doi.org/10.1016/j.rmed.2018.11.017 | Excluded | Lack of relation between the title and the abstract of the article with the subject under study. |
|  | Carter N, Nalbant G, Chahal P, Chattopadhyay K. Effectiveness and safety of self-management interventions for improving glycemic control and health-related quality of life among adults with type 2 diabetes mellitus in sub-Saharan Africa: A systematic review and meta-Analysis. JBI Evidence Synthesis. 2024;22(9):1715-88.https://doi.org/10.11124/JBIES-23-00273 | Excluded | Lack of relation between the title and the abstract of the article with the subject under study. |
|  | Carter T, Schoenaker D, Adams J, Steel A. Paternal preconception modifiable risk factors for adverse pregnancy and offspring outcomes: a review of contemporary evidence from observational studies. BMC public health. 2023;23(1):509.https://doi.org/10.1186/s12889-023-15335-1 | Excluded | Lack of relation between the title and the abstract of the article with the subject under study. |
|  | Caruana M, West LM, Cordina M. Current Asthma Management Practices by Primary School Teaching Staff: A Systematic Review. The Journal of school health. 2021;91(3):227-38.https://doi.org/10.1111/josh.12992 | Excluded | Lack of relation between the title and the abstract of the article with the subject under study. |
|  | Cassileth B. Integrative oncology - Yoga. ONCOLOGY. 2010;24(9) | Excluded | Lack of relation between the title and the abstract of the article with the subject under study. |
|  | Cassim R, Koplin JJ, Dharmage SC, Senaratna BC, Lodge CJ, Lowe AJ, et al. The difference in amount of physical activity performed by children with and without asthma: A systematic review and meta-analysis. The Journal of asthma : official journal of the Association for the Care of Asthma. 2016;53(9):882-92.https://doi.org/10.1080/02770903.2016.1175474 | Excluded | Lack of relation between the title and the abstract of the article with the subject under study. |
|  | Castricum A, Inge P, Goulding L, Perera N, Orchard J. Exercise as medicine – Evidence for prescribing exercise for Australia's nine national health priority areas: An umbrella review. Journal of Science and Medicine in Sport. 2018;21:S5-S6.https://doi.org/10.1016/j.jsams.2018.09.016 | Excluded | Lack of relation between the title and the abstract of the article with the subject under study. |
|  | Cates CJ, Welsh EJ, Rowe BH. Holding chambers (spacers) versus nebulisers for beta-agonist treatment of acute asthma (Cochrane review) [with consumer summary]. Cochrane Database of Systematic Reviews 2013;Issue 9. 2013 | Excluded | Lack of relation between the title and the abstract of the article with the subject under study. |
|  | Cavalcante Marcelino AMF, da Silva HJ. Role of maximal inspiratory presure in the evaluetion of respiratory muscle strength in asthmatics - Systematic review. Revista Portuguesa De Pneumologia. 2010;16(3):463-70.https://doi.org/10.1016/s2173-5115(10)70050-6 | Excluded | Lack of relation between the title and the abstract of the article with the subject under study. |
|  | Cavalcanti AC, Melo ICAR, Medeiros AFD, Neves MVM, Pereira AN, Oliveira EJ. Studies with Cissampelos sympodialis: The search towards the scientific validation of a traditional Brazilian medicine used for the treatment of asthma. Revista Brasileira de Farmacognosia. 2013;23(3):527-41.https://doi.org/10.1590/S0102-695X2013005000029 | Excluded | Lack of relation between the title and the abstract of the article with the subject under study. |
|  | Cavalcanti-Ribeiro P, Andrade-Nascimento M, Morais-De-Jesus M, De Medeiros GM, Daltro-Oliveira R, Conceição JO, et al. Post-traumatic stress disorder as a comorbidity: Impact on disease outcomes. Expert Review of Neurotherapeutics. 2012;12(8):1023-37.https://doi.org/10.1586/ern.12.77 | Excluded | Lack of relation between the title and the abstract of the article with the subject under study. |
|  | Cave L, Cooper MN, Zubrick SR, Shepherd CCJ. Racial discrimination and child and adolescent health in longitudinal studies: A systematic review. Social Science and Medicine. 2020;250.https://doi.org/10.1016/j.socscimed.2020.112864 | Excluded | Lack of relation between the title and the abstract of the article with the subject under study. |
|  | Cegla UH. Is respiratory physiotherapy evidence-based? Basic principles and results of the oscillating PEP techniques. Atemwegs- und Lungenkrankheiten. 2010;36(5):205-16.https://doi.org/10.5414/atp36205 | Excluded | Lack of relation between the title and the abstract of the article with the subject under study. |
|  | Celedon JC. Chronic stress, omics, and asthma. Faseb Journal. 2022;36.https://doi.org/10.1096/fasebj.2022.36.S1.0I203 | Excluded | Lack of relation between the title and the abstract of the article with the subject under study. |
|  | Celli BR, Decramer M, Wedzicha JA, Wilson KC, Agustí A, Criner GJ, et al. An official American Thoracic Society/European Respiratory Society statement: Research questions in COPD. European Respiratory Journal. 2015;45(4):879-905.https://doi.org/10.1183/09031936.00009015 | Excluded | Lack of relation between the title and the abstract of the article with the subject under study. |
|  | Chalmers I, Atkinson P, Fenton M, Firkins L, Crowe S, Cowan K. Tackling treatment uncertainties together: The evolution of the James Lind Initiative, 2003-2013. Journal of the Royal Society of Medicine. 2013;106(12):482-91.https://doi.org/10.1177/0141076813493063 | Excluded | Lack of relation between the title and the abstract of the article with the subject under study. |
|  | Chamberlain S, Birring SS, Garrod R. Nonpharmacological interventions for refractory chronic cough patients: Systematic review. Lung. 2014;192(1):75-85.https://doi.org/10.1007/s00408-013-9508-y | Excluded | Lack of relation between the title and the abstract of the article with the subject under study. |
|  | Chan BKY, Kudsk-Iversen S, Balaguruswamy S, Purewal TS. A complicated simple fall - An atypical case of serogroup Y meningococcal pneumonia with secondary septicaemia and literature review. BMJ Case Reports. 2012.https://doi.org/10.1136/bcr.11.2011.5095 | Excluded | Lack of relation between the title and the abstract of the article with the subject under study. |
|  | Chan JFW, Choy BY, Lai KN. Nephrotic syndrome secondary to strongyloidiasis: A common infection with an uncommon presentation. Hong Kong Journal of Nephrology. 2008;10(1):37-41.https://doi.org/10.1016/S1561-5413(08)60017-4 | Excluded | Lack of relation between the title and the abstract of the article with the subject under study. |
|  | Chang AB, Anderson-James S, Marchant JM. Chronic cough in children. Clinical Pulmonary Medicine. 2014;21(3):138-44.https://doi.org/10.1097/CPM.0000000000000037 | Excluded | Lack of relation between the title and the abstract of the article with the subject under study. |
|  | Chang AB, Bell SC, Byrnes CA, Dawkins P, Holland AE, Kennedy E, et al. Thoracic Society of Australia and New Zealand (TSANZ) position statement on chronic suppurative lung disease and bronchiectasis in children, adolescents and adults in Australia and New Zealand. Respirology. 2023;28(4):339-49.https://doi.org/10.1111/resp.14479 | Excluded | Lack of relation between the title and the abstract of the article with the subject under study. |
|  | Chang AB, Fortescue R, Grimwood K, Alexopoulou E, Bell L, Boyd J, et al. European Respiratory Society guidelines for the management of children and adolescents with bronchiectasis. European Respiratory Journal. 2021;58(2).https://doi.org/10.1183/13993003.02990-2020 | Excluded | Lack of relation between the title and the abstract of the article with the subject under study. |
|  | Chang AB, Grimwood K, Maguire G, King PT, Morris PS, Torzillo PJ. Management of bronchiectasis and chronic suppurative lung disease in Indigenous children and adults from rural and remote Australian communities. Medical Journal of Australia. 2008;189(7):386-93.https://doi.org/10.5694/j.1326-5377.2008.tb02085.x | Excluded | Lack of relation between the title and the abstract of the article with the subject under study. |
|  | Chang AB, Zacharasiewicz A, Goyal V, Boyd J, Alexopoulou E, Aliberti S, et al. Task Force report: European Respiratory Society statement for defining respiratory exacerbations in children and adolescents with bronchiectasis for clinical trials. European Respiratory Journal. 2022;60(5).https://doi.org/10.1183/13993003.00300-2022 | Excluded | Lack of relation between the title and the abstract of the article with the subject under study. |
|  | Chang YL, Chang ST. The effects of intravascular photobiomodulation on sleep disturbance caused by Guillain-Barré syndrome after Astrazeneca vaccine inoculation: Case report and literature review. Medicine (United States). 2022;101(6).https://doi.org/10.1097/MD.0000000000028758 | Excluded | Lack of relation between the title and the abstract of the article with the subject under study. |
|  | Chaudhari V, Clark LA, Iqbal U, Mulligan K. PMU81 WHAT IS THE VALUE OF DIGITAL THERAPEUTICS? A REVIEW OF HEALTH ECONOMIC EVIDENCE. Value in Health. 2020;23:S247-S8.https://doi.org/10.1016/j.jval.2020.04.848 | Excluded | Lack of relation between the title and the abstract of the article with the subject under study. |
|  | Chehab MS, Bafagih HA, Al-Dabbagh MM. Overview of bronchiolitis. Saudi Medical Journal. 2005;26(2):177-90 | Excluded | Lack of relation between the title and the abstract of the article with the subject under study. |
|  | Chen J, Spracklen CN, Marenne G, Varshney A, Corbin LJ, Luan J, et al. The trans-ancestral genomic architecture of glycemic traits. Nature Genetics. 2021;53(6):840-60.https://doi.org/10.1038/s41588-021-00852-9 | Excluded | Lack of relation between the title and the abstract of the article with the subject under study. |
|  | Chen YA, Hsu HM, Wang H, Lan HH, Huang SH, Hung CC, et al. Epidemiology, clinical features, and outcomes of strongyloidiasis in Taiwan from 1988 to 2020: A case series and literature review. Journal of Microbiology, Immunology and Infection. 2023;56(1):172-81.https://doi.org/10.1016/j.jmii.2022.06.007 | Excluded | Lack of relation between the title and the abstract of the article with the subject under study. |
|  | Cheng MJ, Huang PH, Liao PW, Chen JT, Chiang TR. Multiple cerebral and cerebellar infarcts as the first clinical manifestation in a patient with churg-strauss syndrome: Case report and literature review. Acta Neurologica Taiwanica. 2012;21(4):169-75 | Excluded | Lack of relation between the title and the abstract of the article with the subject under study. |
|  | Chetry D, Telles S, Balkrishna A. A PubMed-Based Exploration of the Course of Yoga Research from 1948 to 2020. International journal of yoga therapy. 2021;31(1).https://doi.org/10.17761/2021-d-21-00017 | Excluded | Lack of relation between the title and the abstract of the article with the subject under study. |
|  | Cheung W, Gershon A, Ryerson C, Dechman G, Hernandez P, Bhutani M, et al. Creating quality indicators for Canadian pulmonary rehabilitation programs: A rand appropriateness method study. Canadian Journal of Respiratory Critical Care and Sleep Medicine. 2017;1(2):113-4.https://doi.org/10.1080/24745332.2017.1332400 | Excluded | Lack of relation between the title and the abstract of the article with the subject under study. |
|  | Chiappini E, Principi N, Longhi R, Tovo PA, Becherucci P, Bonsignori F, et al. Management of fever in children: Summary of the Italian pediatric society guidelines. Clinical Therapeutics. 2009;31(8):1826-43.https://doi.org/10.1016/j.clinthera.2009.08.006 | Excluded | Lack of relation between the title and the abstract of the article with the subject under study. |
|  | Chinese Society of Tuberculosis CMA, Chinese Throacic Society CMA. Chinese expert consensus on diagnosis and treatment of non-tuberculous mycobacterial pulmonary disease complicated with bronchiectasis. Zhonghua jie he he hu xi za zhi = Zhonghua jiehe he huxi zazhi = Chinese journal of tuberculosis and respiratory diseases. 2025;48(2):101-15.https://doi.org/10.3760/cma.j.cn112147-20240808-00471 | Excluded | Lack of relation between the title and the abstract of the article with the subject under study. |
|  | Chiou CF, Weaver MR, Bell MA, Lee TA, Krieger JW. Development of the multi-attribute pediatric asthma health outcome measure (PAHOM). International Journal for Quality in Health Care. 2005;17(1):23-30.https://doi.org/10.1093/intqhc/mzh086 | Excluded | Lack of relation between the title and the abstract of the article with the subject under study. |
|  | Chipps BE, Spahn JD. What are the determinates of asthma control? Journal of Asthma. 2006;43(8):567-72.https://doi.org/10.1080/02770900600619782 | Excluded | Lack of relation between the title and the abstract of the article with the subject under study. |
|  | Chiu KY, Li JG, Lin Y. Calcium channel blockers for lung function improvement in asthma: A systematic review and meta-analysis. Annals of Allergy, Asthma and Immunology. 2017;119(6):518-23.e3.https://doi.org/10.1016/j.anai.2017.08.013 | Excluded | Lack of relation between the title and the abstract of the article with the subject under study. |
|  | Chohan K, Mittal N, McGillis L, Lopez-Hernandez L, Camacho E, Rachinsky M, et al. A review of respiratory manifestations and their management in Ehlers-Danlos syndromes and hypermobility spectrum disorders. Chron Respir Dis. 2021;18:14799731211025313.https://doi.org/10.1177/14799731211025313 | Excluded | Lack of relation between the title and the abstract of the article with the subject under study. |
|  | Chong-Neto HJ, D'Amato G, Rosario Filho NA. Impact of the environment on the microbiome. Jornal De Pediatria. 2022;98:S32-S7.https://doi.org/10.1016/j.jped.2021.10.001 | Excluded | Lack of relation between the title and the abstract of the article with the subject under study. |
|  | Choubey J, Patel A, Verma MK. Phytotherapy in the treatment of arthritis: A review. International Journal of Pharmaceutical Sciences and Research. 2013;4(8):2853-65.https://doi.org/10.13040/IJPSR.0975-8232 | Excluded | Lack of relation between the title and the abstract of the article with the subject under study. |
|  | Christenson SA. COPD Phenotyping. Respiratory Care. 2023;68(7):871-80.https://doi.org/10.4187/respcare.11035 | Excluded | Lack of relation between the title and the abstract of the article with the subject under study. |
|  | Chua LBA, Andaya PAG, Gutierrez KMF. Effect of weight loss on lung function and asthma control in obese asthmatics: A systematic review. Allergy: European Journal of Allergy and Clinical Immunology. 2021;76(SUPPL 110):103-4.https://doi.org/10.1111/all.15095 | Excluded | Lack of relation between the title and the abstract of the article with the subject under study. |
|  | Chung KF, Barnes PJ. Zafirlukast (Accolate(TM)). Drugs of Today. 1998;34(4):375-88.https://doi.org/10.1358/dot.1998.34.4.472184 | Excluded | Lack of relation between the title and the abstract of the article with the subject under study. |
|  | Ciccone MM, Scicchitano P, Gesualdo M, Zito A, Carbonara S, Ricci G, et al. The role of omega-3 polyunsaturated fatty acids supplementation in childhood: A review. Recent Patents on Cardiovascular Drug Discovery. 2013;8(1):42-55.https://doi.org/10.2174/1574890111308010006 | Excluded | Lack of relation between the title and the abstract of the article with the subject under study. |
|  | Cindy Ng LW, MacKney J, Jenkins S, Hill K. Does exercise training change physical activity in people with COPD? A systematic review and meta-analysis. Chronic Respiratory Disease. 2012;9(1):17-26.https://doi.org/10.1177/1479972311430335 | Excluded | Lack of relation between the title and the abstract of the article with the subject under study. |
|  | Clar C, Tsertsvadze A, Court R, Hundt GL, Clarke A, Sutcliffe P. Clinical effectiveness of manual therapy for the management of musculoskeletal and non-musculoskeletal conditions: Systematic review and update of UK evidence report. Chiropractic and Manual Therapies. 2014;22(1).https://doi.org/10.1186/2045-709X-22-12 | Excluded | Lack of relation between the title and the abstract of the article with the subject under study. |
|  | Clark NM, Dodge JA, Partridge MR, Martinez FJ. Focusing on outcomes: making the most of COPD interventions. International journal of chronic obstructive pulmonary disease. 2009;4:61-77 | Excluded | Lack of relation between the title and the abstract of the article with the subject under study. |
|  | Clarke DM, Currie KC. Depression, anxiety and their relationship with chronic diseases: A review of the epidemiology, risk and treatment evidence. Medical Journal of Australia. 2009;190(7 SUPPL.):S54-S60.https://doi.org/10.5694/j.1326-5377.2009.tb02471.x | Excluded | Lack of relation between the title and the abstract of the article with the subject under study. |
|  | Clements WT, Lee SR, Bloomer RJ. Nitrate ingestion: A review of the health and physical performance effects. Nutrients. 2014;6(11):5224-64.https://doi.org/10.3390/nu6115224 | Excluded | Lack of relation between the title and the abstract of the article with the subject under study. |
|  | Cline EC, Davis R, Burkard JF. Vocal cord dysfunction: A case report. AANA Journal. 2006;74(5):375-8 | Excluded | Lack of relation between the title and the abstract of the article with the subject under study. |
|  | Cmorej PC, Nesvadba M, Mamova A, Babela R, Peran D, Pekara J, et al. Anaphylaxis in public health. Neuroendocrinology Letters. 2019;40:3-10 | Excluded | Lack of relation between the title and the abstract of the article with the subject under study. |
|  | Coffman JM, Cabana MD, Yelin EH. Do school-based asthma education programs improve self-management and health outcomes? Pediatrics 2009 Aug;124(2):729-742. 2009 | Excluded | Lack of relation between the title and the abstract of the article with the subject under study. |
|  | Confalonieri M, Kodric M, Della Porta R, Demsar M. Management of patients with exacerbation of bronchial asthma. Minerva Pneumologica. 2006;45(1):1-15 | Excluded | Lack of relation between the title and the abstract of the article with the subject under study. |
|  | Connolly B, Macbean V, Crowley C, Lunt A, Moxham J, Rafferty GF, et al. Ultrasound for the assessment of peripheral skeletal muscle architecture in critical illness: A systematic review. Critical Care Medicine. 2015;43(4):897-905.https://doi.org/10.1097/CCM.0000000000000821 | Excluded | Lack of relation between the title and the abstract of the article with the subject under study. |
|  | Connolly B, Salisbury L, O'Neill B, Geneen L, Douiri A, Grocott MP, et al. Exercise rehabilitation following intensive care unit discharge for recovery from critical illness: executive summary of a Cochrane Collaboration systematic review. Journal of cachexia, sarcopenia and muscle. 2016;7(5):520-6 | Excluded | Lack of relation between the title and the abstract of the article with the subject under study. |
|  | Connolly B, Salisbury L, O'Neill B, Geneen L, Douiri A, Grocott MP, et al. Exercise rehabilitation following intensive care unit discharge for recovery from critical illness. Cochrane Database of Systematic Reviews. 2015;2015(6).https://doi.org/10.1002/14651858.CD008632.pub2 | Excluded | Lack of relation between the title and the abstract of the article with the subject under study. |
|  | Connolly MJ, Yohannes AM. The impact of depression in older patients with chronic obstructive pulmonary disease and asthma. Maturitas. 2016;92:9-14.https://doi.org/10.1016/j.maturitas.2016.07.005 | Excluded | Lack of relation between the title and the abstract of the article with the subject under study. |
|  | Constant CA, Boyd J, Bush A, Hill AT, Powell Z, Zacharasiewicz A, et al. International consensus statement on core outcomes for clinical trials in children and adolescents with bronchiectasis - Child-BEARnet ERS CRC. European Respiratory Journal. 2023;62:PA4460.https://doi.org/10.1183/13993003.congress-2023.PA4460 | Excluded | Lack of relation between the title and the abstract of the article with the subject under study. |
|  | Coogle J, Coogle B, Quezada J. Hypnosis in the Treatment of Pediatric Functional Neurological Disorder: The Magic Glove Technique. Pediatric Neurology. 2021;125:20-5.https://doi.org/10.1016/j.pediatrneurol.2021.08.011 | Excluded | Lack of relation between the title and the abstract of the article with the subject under study. |
|  | Cooke MW, Higgins J, Kidd P. Use of emergency observation and assessment wards: a systematic literature review. Emergency Medicine Journal. 2003;20(2):138-42.https://doi.org/10.1136/emj.20.2.138 | Excluded | Lack of relation between the title and the abstract of the article with the subject under study. |
|  | Cooley C, Park Y, Ajilore O, Leow A, Nyenhuis SM. Impact of interventions targeting anxiety and depression in adults with asthma. Journal of Asthma. 2022;59(2):273-87.https://doi.org/10.1080/02770903.2020.1847927 | Excluded | Lack of relation between the title and the abstract of the article with the subject under study. |
|  | Coop CA, Adams KE, Webb CN. SCUBA Diving and Asthma: Clinical Recommendations and Safety. Clinical Reviews in Allergy and Immunology. 2016;50(1):18-22.https://doi.org/10.1007/s12016-015-8474-y | Excluded | Lack of relation between the title and the abstract of the article with the subject under study. |
|  | Corcione A, Borrelli M, Radice L, Sacco O, Torre M, Santoro F, et al. Chronic respiratory disorders due to aberrant innominate artery: a case series and critical review of the literature. Italian Journal of Pediatrics. 2023;49(1).https://doi.org/10.1186/s13052-023-01473-0 | Excluded | Lack of relation between the title and the abstract of the article with the subject under study. |
|  | Corrao WM. Chronic cough: A manifestation of bronchial asthma. Comprehensive Therapy. 1982;8(3):22-6 | Excluded | Lack of relation between the title and the abstract of the article with the subject under study. |
|  | Corren J. The relationship between allergic rhinitis and bronchial asthma. Current Opinion in Pulmonary Medicine. 1999;5(1):35-7.https://doi.org/10.1097/00063198-199901000-00006 | Excluded | Lack of relation between the title and the abstract of the article with the subject under study. |
|  | Costa E, Giardini A, Savin M, Menditto E, Lehane E, Laosa O, et al. Interventional tools to improve medication adherence: Review of literature. Patient Preference and Adherence. 2015;9:1303-14.https://doi.org/10.2147/PPA.S87551 | Excluded | Lack of relation between the title and the abstract of the article with the subject under study. |
|  | Cote P, Hartvigsen J, Axen I, Leboeuf-Yde C, Corso M, Shearer H, et al. The global summit on the efficacy and effectiveness of spinal manipulative therapy for the prevention and treatment of non-musculoskeletal disorders: a systematic review of the literature. Chiropractic & Manual Therapies 2021 Feb 17;29(8):Epub. 2021 | Excluded | Lack of relation between the title and the abstract of the article with the subject under study. |
|  | Coulter A, Entwistle VA, Eccles A, Ryan S, Shepperd S, Perera R. Personalised care planning for adults with chronic or long‐term health conditions. Cochrane Database of Systematic Reviews. 2015(3).https://doi.org/10.1002/14651858.CD010523.pub2 | Excluded | Lack of relation between the title and the abstract of the article with the subject under study. |
|  | Couto Alves A, De Silva NMG, Karhunen V, Sovio U, Das S, Taal HR, et al. GWAS on longitudinal growth traits reveals different genetic factors influencing infant, child, and adult BMI. Science advances. 2019;5(9):eaaw3095.https://doi.org/10.1126/sciadv.aaw3095 | Excluded | Lack of relation between the title and the abstract of the article with the subject under study. |
|  | Cramer H, Krucoff C, Dobos G. Adverse Events Associated with Yoga: A Systematic Review of Published Case Reports and Case Series. PLoS ONE. 2013;8(10).https://doi.org/10.1371/journal.pone.0075515 | Excluded | Lack of relation between the title and the abstract of the article with the subject under study. |
|  | Crane-Godreau MA, Payne P. A history of second hand smoke exposure: are we asking the right questions? Frontiers in Physiology. 2013;4.https://doi.org/10.3389/fphys.2013.00025 | Excluded | Lack of relation between the title and the abstract of the article with the subject under study. |
|  | Criner G, Duffy S. Reducing and managing chronic obstructive pulmonary disease exacerbations with tiotropium + olodaterol. Current Medical Research and Opinion. 2021;37(2):275-84.https://doi.org/10.1080/03007995.2020.1841615 | Excluded | Lack of relation between the title and the abstract of the article with the subject under study. |
|  | Cristea AI, Baker CD, Allen J, Amin R, Austin ED, Cataletto ME, et al. Outpatient Respiratory Management of Infants, Children, and Adolescents with Post-Prematurity Respiratory Disease An Official American Thoracic Society Clinical Practice Guideline. American Journal of Respiratory and Critical Care Medicine. 2021;204(12):E115-E33.https://doi.org/10.1164/rccm.202110-2269ST | Excluded | Lack of relation between the title and the abstract of the article with the subject under study. |
|  | Croitoru A, Bogdan MA. Evidences related to pulmonary rehabilitation in the respiratory pathology. Pneumologia. 2014;63(2):88-95 | Excluded | Lack of relation between the title and the abstract of the article with the subject under study. |
|  | Crowe S, Regan S, Daly A. Public engagement in outcomes development-three degrees of separation. Trials. 2015;16 | Excluded | Lack of relation between the title and the abstract of the article with the subject under study. |
|  | Cubała WJ, Gabrielsson A. Sleep related amnestic behaviors due to zolpidem. Klinik Psikofarmakoloji Bulteni. 2014;24(2):188-94.https://doi.org/10.5455/bcp.20130527020102 | Excluded | Lack of relation between the title and the abstract of the article with the subject under study. |
|  | Cubo E, Gallego-Nieto C, Elizari-Roncal M, Barroso-Pérez T, Collazo C, Calvo S, et al. Is restless legs syndrome associated with an increased risk of mortality? A meta-analysis of cohort studies. Tremor and Other Hyperkinetic Movements. 2019;9:1-11.https://doi.org/10.5334/tohm.508 | Excluded | Lack of relation between the title and the abstract of the article with the subject under study. |
|  | Cui N, Dai T, Liu Y, Wang YY, Lin JY, Zheng QF, et al. Laryngopharyngeal reflux disease: Updated examination of mechanisms, pathophysiology, treatment, and association with gastroesophageal reflux disease. World Journal of Gastroenterology. 2024;30(16):2209-19.https://doi.org/10.3748/wjg.v30.i16.2209 | Excluded | Lack of relation between the title and the abstract of the article with the subject under study. |
|  | Cullinane A, Garvey M, Walsh C, Gibbons J, Creighton A. A Scoping Review of Non-Structural Airway Disease as a Cause of Poor Performance in Racehorses. Animals. 2023;13(3).https://doi.org/10.3390/ani13030429 | Excluded | Lack of relation between the title and the abstract of the article with the subject under study. |
|  | Cummins CO, Prochaska JO, Driskell MM, Evers KE, Wright JA, Prochaska JM, et al. Development of review criteria to evaluate health behavior change websites. Journal of Health Psychology. 2003;8(1):55-62.https://doi.org/10.1177/1359105303008001434 | Excluded | Lack of relation between the title and the abstract of the article with the subject under study. |
|  | Currie GP, Devereux GS, Lee DKC, Ayres JG. Recent developments in asthma management. BMJ: British Medical Journal (International Edition). 2005;330(7491):585-9.https://doi.org/10.1136/bmj.330.7491.585 | Excluded | Lack of relation between the title and the abstract of the article with the subject under study. |
|  | Currie SS, Strong KA, Ware E. Bringing Gratitude to Aphasia Intervention: A Scoping Review of Gratitude Interventions for Adults with Chronic Health Conditions. Seminars in Speech and Language. 2024;45(4):283-99.https://doi.org/10.1055/s-0044-1791647 | Excluded | Lack of relation between the title and the abstract of the article with the subject under study. |
|  | Cushing CC, Steele RG. A Meta-Analytic Review of eHealth Interventions for Pediatric Health Promoting and Maintaining Behaviors. Journal of Pediatric Psychology. 2010;35(9):937-49.https://doi.org/10.1093/jpepsy/jsq023 | Excluded | Lack of relation between the title and the abstract of the article with the subject under study. |
|  | Custovic A. Allergic rhinitis. Pediatric Pulmonology. 2017;52:S38-S9.https://doi.org/10.1002/ppul.23729 | Excluded | Lack of relation between the title and the abstract of the article with the subject under study. |
|  | Cutrufello PT, Smoliga JM, Rundell KW. Small Things Make a Big Difference: Particulate Matter and Exercise. Sports Medicine. 2012;42(12):1041-58 | Excluded | Lack of relation between the title and the abstract of the article with the subject under study. |
|  | Dahm KT, Dalsbø TK, Kirkehei I, Reinar LM. NIPH Systematic Reviews: Executive Summaries. Effect of Respiratory Muscle Training for Acute Traumatic High Spinal Cord Injury: A Systematic Review. Oslo, Norway: Knowledge Centre for the Health Services at The Norwegian Institute of Public Health (NIPH) | Excluded | Lack of relation between the title and the abstract of the article with the subject under study. |
|  | Daines L, McLean S, Buelo A, Lewis S, Sheikh A, Pinnock H. Clinical prediction models to support the diagnosis of asthma in primary care: A systematic review. Thorax. 2018;73:A171-A2.https://doi.org/10.1136/thorax-2018-212555.286 | Excluded | Lack of relation between the title and the abstract of the article with the subject under study. |
|  | Damianaki A, Vagiakis E, Sigala I, Pataka A, Rovina N, Vlachou A, et al. The co-existence of obstructive sleep apnea and bronchial asthma: Revelation of a new asthma phenotype? Journal of Clinical Medicine. 2019;8(9).https://doi.org/10.3390/jcm8091476 | Excluded | Lack of relation between the title and the abstract of the article with the subject under study. |
|  | D'Andrea G. Quercetin: A flavonol with multifaceted therapeutic applications? Fitoterapia. 2015;106:256-71.https://doi.org/10.1016/j.fitote.2015.09.018 | Excluded | Lack of relation between the title and the abstract of the article with the subject under study. |
|  | Darchini-Maragheh E, Balali-Mood M. Delayed complications and long-term management of sulfur mustard poisoning: Recent advances by iranian researchers (part I of II). Iranian Journal of Medical Sciences. 2018;43(2):103-24 | Excluded | Lack of relation between the title and the abstract of the article with the subject under study. |
|  | Das M, Thajuddin N, Patra S, Pundir M. Ancient Indian Diet - A Balanced Diet For the Healthy Diversity of Gut Microbiota and Management of Asthma. Current Research in Nutrition &amp; Food Science. 2024;12(1):349-73.https://doi.org/10.12944/CRNFSJ.12.1.29 | Excluded | Lack of relation between the title and the abstract of the article with the subject under study. |
|  | Dauletbaev N, Oftring ZS, Akik W, Michaelis-Braun L, Korel J, Lands LC, et al. A scoping review of mHealth monitoring of pediatric bronchial asthma before and during COVID-19 pandemic. Paediatric Respiratory Reviews. 2022;43:67-77.https://doi.org/10.1016/j.prrv.2022.01.002 | Excluded | Lack of relation between the title and the abstract of the article with the subject under study. |
|  | Davidson LM, Berkelhamer SK. Bronchopulmonary dysplasia: Chronic lung disease of infancy and long-term pulmonary outcomes. Journal of Clinical Medicine. 2017;6(1).https://doi.org/10.3390/jcm6010004 | Excluded | Lack of relation between the title and the abstract of the article with the subject under study. |
|  | Davies E, Rogers NK, Lloyd-Lavery A, Grindlay DJC, Thomas KS. What's new in atopic eczema? An analysis of systematic reviews published in 2015. Part 1: epidemiology and methodology. Clinical and Experimental Dermatology. 2018;43(4):375-9.https://doi.org/10.1111/ced.13377 | Excluded | Lack of relation between the title and the abstract of the article with the subject under study. |
|  | Dawson S, Johnson H, Huntley AL, Turner KM, McCahon D. Understanding non-recreational prescription medication-sharing behaviours: a systematic review. British Journal of General Practice. 2024;74(740):e183-e8.https://doi.org/10.3399/BJGP.2023.0189 | Excluded | Lack of relation between the title and the abstract of the article with the subject under study. |
|  | De A, Rastogi D. Association of pediatric obesity and asthma, pulmonary physiology, metabolic dysregulation, and atopy; and the role of weight management. Expert Review of Endocrinology and Metabolism. 2019;14(5):335-49.https://doi.org/10.1080/17446651.2019.1635007 | Excluded | Lack of relation between the title and the abstract of the article with the subject under study. |
|  | De Gennaro R, Gastaldo E, Tamborino C, Baraldo M, Casula N, Pedrali M, et al. Selective cranial multineuritis in severe COVID-19 pneumonia: two cases and literature review. Neurological Sciences. 2021;42(5):1643-8.https://doi.org/10.1007/s10072-021-05087-4 | Excluded | Lack of relation between the title and the abstract of the article with the subject under study. |
|  | de Godoy V, Zanetti NM, Johnston C. Manual hyperinflation in airway clearance in pediatric patients: a systematic review. Revista Brasileira de Terapia Intensiva 2013 Jul-Sep;25(3):258-262. 2013 | Excluded | Lack of relation between the title and the abstract of the article with the subject under study. |
|  | De Jongste JC, Jongejan RC, Kerrebijn KF. Airway smooth muscle in astma. Progress in asthma and COPD: proceedings of the Symposium 'Progress in asthma and COPD' ICS849. 1989:15-33 | Excluded | Lack of relation between the title and the abstract of the article with the subject under study. |
|  | de Medeiros ER, da Cruz Reboucas DG, de Sousa Paiva AC, Abdias do Nascimento CP, Bezerra e Silva SY, Galvao Pinto ES. Studies evaluating of health interventions at schools: an integrative literature review. Revista Latino-Americana De Enfermagem. 2018;26.https://doi.org/10.1590/1518-8345.2463.3008 | Excluded | Lack of relation between the title and the abstract of the article with the subject under study. |
|  | de Rezende DRB, Andrade Neto I, Iunes DH, Carvalho LC. Analysis of the effectiveness of remote intervention of patients affected by chronic diseases: A systematic review and meta-analysis. Journal of Medicine Access. 2023;7.https://doi.org/10.1177/27550834231197316 | Excluded | Lack of relation between the title and the abstract of the article with the subject under study. |
|  | Debon R, Coleone JD, Bellei EA, De Marchi ACB. Mobile health applications for chronic diseases: A systematic review of features for lifestyle improvement. Diabetes and Metabolic Syndrome: Clinical Research and Reviews. 2019;13(4):2507-12.https://doi.org/10.1016/j.dsx.2019.07.016 | Excluded | Lack of relation between the title and the abstract of the article with the subject under study. |
|  | Declerck S, Testelmans D, Nafteux PH, Coosemans W, Belge C, Decramer M, et al. Diaphragm plication for unilateral diaphragm paralysis: A case report and review of the literature. Acta Clinica Belgica. 2013;68(4):311-5.https://doi.org/10.2143/ACB.3307 | Excluded | Lack of relation between the title and the abstract of the article with the subject under study. |
|  | Deenstra D, Wolvetang N, Kock S, Wills S, Cobben N, Wijnen P, et al. PREVALENCE OF INTERSTITIAL AND OTHER LUNG DISEASES ON ARUBA. Sarcoidosis Vasculitis and Diffuse Lung Diseases. 2017;34(3):217-25 | Excluded | Lack of relation between the title and the abstract of the article with the subject under study. |
|  | del Río Navarro BE, Sol Monterrey EG, Paredes Novelo C, Sienra Monge JJ. Anaphylaxis and urticaria caused by exercise. Review of the literature and report of 2 cases. Revista alergia : organo oficial de la Sociedad Mexicana de Alergia e Inmunlogía. 1993;40(3):63-6 | Excluded | Lack of relation between the title and the abstract of the article with the subject under study. |
|  | Delgado L, Moreira A, Capão-Filipe M. Rhinitis and its impact on sports. Allergy and Clinical Immunology International. 2006;18(3):98-105.https://doi.org/10.1027/0838-1925.18.3.98 | Excluded | Lack of relation between the title and the abstract of the article with the subject under study. |
|  | Delgoshaei B, Mobinizadeh M, Mojdekar R, Afzal E, Arabloo J, Mohamadi E. Telemedicine: A systematic review of economic evaluations. Medical journal of the Islamic Republic of Iran. 2017;31:113.https://doi.org/10.14196/mjiri.31.113 | Excluded | Lack of relation between the title and the abstract of the article with the subject under study. |
|  | Demarin V, Vuković V, Lovrenčić-Huzjan A, Lušić I, Jančuljak D, Wilheim K, et al. Evidence based guidelines for treatment of primary headaghes. Acta Clinica Croatica. 2005;44(2):139-83 | Excluded | Lack of relation between the title and the abstract of the article with the subject under study. |
|  | Demoly P, Calderon MA, Casale T, Scadding G, Annesi-Maesano I, Braun JJ, et al. Assessment of disease control in allergic rhinitis. Clinical and Translational Allergy. 2013;3(1):1-7.https://doi.org/10.1186/2045-7022-3-7 | Excluded | Lack of relation between the title and the abstract of the article with the subject under study. |
|  | Denjean A, Vorger P. Physiology and respiratory function tests. Revue des Maladies Respiratoires. 2003;20(SPEC.):5S20-5S5 | Excluded | Lack of relation between the title and the abstract of the article with the subject under study. |
|  | Dennett EJ, Janjua S, Stovold E, Harrison SL, McDonnell MJ, Holland AE. Tailored or adapted interventions for adults with chronic obstructive pulmonary disease and at least one other long‐term condition: a mixed methods review. Cochrane Database of Systematic Reviews. 2021(7).https://doi.org/10.1002/14651858.CD013384.pub2 | Excluded | Lack of relation between the title and the abstract of the article with the subject under study. |
|  | Denning DW, O'Driscoll BR, Hogaboam CM, Bowyer P, Niven RM. The link between fungi and severe asthma: A summary of the evidence. European Respiratory Journal. 2006;27(3):615-26.https://doi.org/10.1183/09031936.06.00074705 | Excluded | Lack of relation between the title and the abstract of the article with the subject under study. |
|  | Detaille SI, Heerkens YF, Engels JA, van der Gulden JW, van Dijk FJ. Common prognostic factors of work disability among employees with a chronic somatic disease: a systematic review of cohort studies. Scandinavian journal of work, environment & health. 2009;35(4):261-81.https://doi.org/10.5271/sjweh.1337 | Excluded | Lack of relation between the title and the abstract of the article with the subject under study. |
|  | Detailleur S, Vos R, Goeminne P. The Deteriorating Patient: Therapies including Lung Transplantation. Seminars in Respiratory and Critical Care Medicine. 2021;42(4):623-38.https://doi.org/10.1055/s-0041-1730946 | Excluded | Lack of relation between the title and the abstract of the article with the subject under study. |
|  | Dhanraj P, Pitere R, Pepper MS. The impact of obesity on the cellular and molecular pathophysiology of COVID-19. South African Medical Journal. 2021;111(3):211-4.https://doi.org/10.7196/SAMJ.2021V111I2.15398 | Excluded | Lack of relation between the title and the abstract of the article with the subject under study. |
|  | Di Bona D, Stefania M, Leto-Barone MS, La Piana S, Di Lorenzo G. Sublingual immunotherapy with natural grass pollen extracts: An appraisal of the evidence. Therapy. 2011;8(4):443-54.https://doi.org/10.2217/thy.11.31 | Excluded | Lack of relation between the title and the abstract of the article with the subject under study. |
|  | Di Genova L, Penta L, Biscarini A, Di Cara G, Esposito S. Children with obesity and asthma: Which are the best options for their management? Nutrients. 2018;10(11).https://doi.org/10.3390/nu10111634 | Excluded | Lack of relation between the title and the abstract of the article with the subject under study. |
|  | Di Maria G, Spicuzza L, Mazzarella G. Future treatment of chronic obstructive pulmonary disease. Monaldi Archives for Chest Disease - Pulmonary Series. 2002;57(3-4):200-5 | Excluded | Lack of relation between the title and the abstract of the article with the subject under study. |
|  | Dicpinigaitis PV. Chronic cough due to asthma: ACCP evidende-based clinical practice guidelines. Chest. 2006;129(1 SUPPL.):75S-9S.https://doi.org/10.1378/chest.129.1_suppl.75S | Excluded | Lack of relation between the title and the abstract of the article with the subject under study. |
|  | DiDario AG, Becker JM, editors. Asthma, sports, and death. Allergy and Asthma Proceedings; 2005. | Excluded | Lack of relation between the title and the abstract of the article with the subject under study. |
|  | Dimidjian S, Segal ZV. Prospects for a Clinical Science of Mindfulness-Based Intervention. American Psychologist. 2015;70(7):593-620.https://doi.org/10.1037/a0039589 | Excluded | Lack of relation between the title and the abstract of the article with the subject under study. |
|  | Dinakar C, Oppenheimer J, Portnoy J, Bacharier LB, Li J, Kercsmar CM, et al. Management of acute loss of asthma control in the yellow zone: A practice parameter. Annals of Allergy, Asthma and Immunology. 2014;113(2):143-59.https://doi.org/10.1016/j.anai.2014.05.017 | Excluded | Lack of relation between the title and the abstract of the article with the subject under study. |
|  | Ding B, Lu Y. Omalizumab in combination with subcutaneous immunotherapy for the treatment of multiple allergies associated with attention-deficit/hyperactivity disorder: a case report and a literature review. Frontiers in Pharmacology. 2024;15.https://doi.org/10.3389/fphar.2024.1367551 | Excluded | Lack of relation between the title and the abstract of the article with the subject under study. |
|  | Ding D, Elbarbary M. Addressing the syndemics of physical inactivity and air pollution. CMAJ. 2021;193(32):E1255-E6.https://doi.org/10.1503/cmaj.211282 | Excluded | Lack of relation between the title and the abstract of the article with the subject under study. |
|  | Dirweesh A, Alvarez C, Khan M, Shah N. A unilateral hyperlucent lung - Swyer-James syndrome: A case report and literature review. Respiratory Medicine Case Reports. 2017;20:104-6.https://doi.org/10.1016/j.rmcr.2017.01.004 | Excluded | Lack of relation between the title and the abstract of the article with the subject under study. |
|  | Dixon EG, Rugg-Gunn CEM, Sellick V, Sinha IP, Hawcutt DB. Adverse drug reactions of leukotriene receptor antagonists in children with asthma: A systematic review. BMJ Paediatrics Open. 2021;5(1).https://doi.org/10.1136/bmjpo-2021-001206 | Excluded | Lack of relation between the title and the abstract of the article with the subject under study. |
|  | Do Amaral YNV, Marano D, Filha MMT, Moreira MEL. Pre-gestational overweight and polyunsaturated fatty acids in human milk: theoretical causality model. Ciencia e Saude Coletiva. 2024;29(2).https://doi.org/10.1590/1413-81232024292.10752022EN | Excluded | Lack of relation between the title and the abstract of the article with the subject under study. |
|  | Doan D, Luks AM. Wilderness and adventure travel with underlying asthma. Wilderness and Environmental Medicine. 2014;25(2):231-40.https://doi.org/10.1016/j.wem.2013.08.009 | Excluded | Lack of relation between the title and the abstract of the article with the subject under study. |
|  | Dodd S, Widnall E, Russell AE, Curtin EL, Simmonds R, Limmer M, et al. School-based peer education interventions to improve health: a global systematic review of effectiveness. Bmc Public Health. 2022;22(1).https://doi.org/10.1186/s12889-022-14688-3 | Excluded | Lack of relation between the title and the abstract of the article with the subject under study. |
|  | Dogné JM, De Leval X, Delarge J, Masereel B. Recent developments of thromboxane modulators. Expert Opinion on Therapeutic Patents. 2001;11(11):1663-75.https://doi.org/10.1517/13543776.11.11.1663 | Excluded | Lack of relation between the title and the abstract of the article with the subject under study. |
|  | Dolovich MB, Ahrens RC, Hess DR, Anderson P, Dhand R, Rau JL, et al. Device selection and outcomes of aerosol therapy: Evidence-based guidelines. Chest. 2005;127(1):335-71.https://doi.org/10.1378/chest.127.1.335 | Excluded | Lack of relation between the title and the abstract of the article with the subject under study. |
|  | Domhardt M, Schröder A, Geirhos A, Steubl L, Baumeister H. Efficacy of digital health interventions in youth with chronic medical conditions: A meta-analysis. Internet Interventions. 2021;24.https://doi.org/10.1016/j.invent.2021.100373 | Excluded | Lack of relation between the title and the abstract of the article with the subject under study. |
|  | Domingues G, de Moraes-Filho JPP. Gastroesophageal reflux disease: a practical approach. Arquivos de Gastroenterologia. 2021;58(4):525-33.https://doi.org/10.1590/S0004-2803.202100000-94 | Excluded | Lack of relation between the title and the abstract of the article with the subject under study. |
|  | Dominski FH, Branco JHL, Buonanno G, Stabile L, da Silva MG, Andrade A. Effects of air pollution on health: A mapping review of systematic reviews and meta-analyses. Environmental Research. 2021;201.https://doi.org/10.1016/j.envres.2021.111487 | Excluded | Lack of relation between the title and the abstract of the article with the subject under study. |
|  | Dong AC, Stephenson MD, Stagnaro-Green AS. The need for dynamic clinical guidelines: A systematic review of new research published after release of the 2017 ATA guidelines on thyroid disease during pregnancy and the postpartum. Frontiers in Endocrinology. 2020;11.https://doi.org/10.3389/fendo.2020.00193 | Excluded | Lack of relation between the title and the abstract of the article with the subject under study. |
|  | Dong N. (PO-115) Newly Diagnosed Psychogenic Non-epileptic Seizures in the Context of Chronic Pain: Addressing Challenges in Management and Review of Literature. Journal of the Academy of Consultation-Liaison Psychiatry. 2022;63:S55-S6.https://doi.org/10.1016/j.jaclp.2022.03.116 | Excluded | Lack of relation between the title and the abstract of the article with the subject under study. |
|  | Donlan M, Fontela PS, Puligandla PS. Use of continuous positive airway pressure (CPAP) in acute viral bronchiolitis: a systematic review. Pediatric Pulmonology 2011 Aug;46(8):736-746. 2011 | Excluded | Lack of relation between the title and the abstract of the article with the subject under study. |
|  | dos Santos NC, Miravitlles M, Camelier AA, de Almeida VDC, Tosta Maciel RRB, Rosa Camelier FW. Prevalence and Impact of Comorbidities in Individuals with Chronic Obstructive Pulmonary Disease: A Systematic Review. Tuberculosis and Respiratory Diseases. 2022;85(3):205-20.https://doi.org/10.4046/trd.2021.0179 | Excluded | Lack of relation between the title and the abstract of the article with the subject under study. |
|  | Dossett ML, Cohen EM, Cohen J. Integrative Medicine for Gastrointestinal Disease. Primary Care - Clinics in Office Practice. 2017;44(2):265-80.https://doi.org/10.1016/j.pop.2017.02.002 | Excluded | Lack of relation between the title and the abstract of the article with the subject under study. |
|  | Dougherty RH, Fahy JV. Acute exacerbations of asthma: Epidemiology, biology and the exacerbation-prone phenotype. Clinical and Experimental Allergy. 2009;39(2):193-202.https://doi.org/10.1111/j.1365-2222.2008.03157.x | Excluded | Lack of relation between the title and the abstract of the article with the subject under study. |
|  | Douglas JE, Bosso JV. What's New in the Diagnosis and Treatment of Aspirin-Exacerbated Respiratory Disease: A Brief Review. American Journal of Rhinology and Allergy. 2023;37(2):198-206.https://doi.org/10.1177/19458924221145254 | Excluded | Lack of relation between the title and the abstract of the article with the subject under study. |
|  | Doward LC, Balp MM, Twiss J, Slota C, Cryer D, Brass CA, et al. Development of a Patient-Reported Outcome Measure for Non-Alcoholic Steatohepatitis (NASH-CHECK): Results of a Qualitative Study. Patient. 2021;14(5):533-43.https://doi.org/10.1007/s40271-020-00485-w | Excluded | Lack of relation between the title and the abstract of the article with the subject under study. |
|  | Downs CA. Functional assessment of chronic obstructive pulmonary disease. Journal of the American Academy of Nurse Practitioners. 2011;23(4):161-7.https://doi.org/10.1111/j.1745-7599.2011.00602.x | Excluded | Lack of relation between the title and the abstract of the article with the subject under study. |
|  | Dreischulte T, Grant AM, McCowan C, McAnaw JJ, Guthrie B. Quality and safety of medication use in primary care: Consensus validation of a new set of explicit medication assessment criteria and prioritisation of topics for improvement. BMC Clinical Pharmacology. 2012;12.https://doi.org/10.1186/1472-6904-12-5 | Excluded | Lack of relation between the title and the abstract of the article with the subject under study. |
|  | Driehuis F, Hoogeboom TJ, Nijhuis-van der Sanden MWG, de Bie RA, Staal JB. Spinal manual therapy in infants, children and adolescents: a systematic review and meta-analysis on treatment indication, technique and outcomes. PLoS ONE 2019 Jun;14(6):e0218940. 2019 | Excluded | Lack of relation between the title and the abstract of the article with the subject under study. |
|  | Drysdale SB, Green CA, Sande CJ. Best practice in the prevention and management of paediatric respiratory syncytial virus infection. Therapeutic Advances in Infectious Disease. 2015;3(2):63-71.https://doi.org/10.1177/2049936116630243 | Excluded | Lack of relation between the title and the abstract of the article with the subject under study. |
|  | Duarte J, Castelo Branco J, Rodrigues F, Vaz M, Santos Baptista J. Occupational Exposure to Mineral Dust in Mining and Earthmoving Works: A Scoping Review. Safety. 2022;8(1).https://doi.org/10.3390/safety8010009 | Excluded | Lack of relation between the title and the abstract of the article with the subject under study. |
|  | Dubovyi A, Chelimo C, Berry S, Bisyuk Y, Grant CC. Asthma definitions in population-based birth cohorts: A review. Allergy: European Journal of Allergy and Clinical Immunology. 2018;73:685-6.https://doi.org/10.1111/all.13539 | Excluded | Lack of relation between the title and the abstract of the article with the subject under study. |
|  | Duke T. Randomised controlled trials in child and adolescent health in 2023. Archives of Disease in Childhood. 2023;108(9):709-14.https://doi.org/10.1136/archdischild-2023-326046 | Excluded | Lack of relation between the title and the abstract of the article with the subject under study. |
|  | Dulaney D, Dave P, Walsh S, Mehandru S, Colombel JF, Agrawal M. Noninfectious Pulmonary Complications Associated with Anti-Integrin Therapy: A Case Report and Systematic Review of the Literature. Inflammatory Bowel Diseases. 2022;28(3):479-83.https://doi.org/10.1093/ibd/izab212 | Excluded | Lack of relation between the title and the abstract of the article with the subject under study. |
|  | Dupre AE, Slama MCC. Mononeuropathy Multiplex after Severe SARS-CoV-2 Infection: A Case Series and Literature Review. Journal of Clinical Neuromuscular Disease. 2023;25(1):27-35.https://doi.org/10.1097/CND.0000000000000450 | Excluded | Lack of relation between the title and the abstract of the article with the subject under study. |
|  | Dwamena F, Holmes-Rovner M, Gaulden CM, Jorgenson S, Sadigh G, Sikorskii A, et al. Interventions for providers to promote a patient-centred approach in clinical consultations. Cochrane Database of Systematic Reviews. 2012;2012(12).https://doi.org/10.1002/14651858.CD003267.pub2 | Excluded | Lack of relation between the title and the abstract of the article with the subject under study. |
|  | Dwornik M, Puszczałowska-Lizis E, Wójcik M, Szajkowski S, Graczykowski M, Szymański D, et al. Efficacy of osteopathic manipulative treatment (93.6, ICD-9) - systematic review. Medical Studies/Studia Medyczne. 2024;40(3):289-307.https://doi.org/10.5114/ms.2024.138016 | Excluded | Lack of relation between the title and the abstract of the article with the subject under study. |
|  | Eberle C, Jünger K, Debatin KM, Wabitsch M. Spontaneously occurring pneumomediastinum related to a pneumopericardium, a pneumothorax and a skin emphysema in a 12-year old boy. Klinische Padiatrie. 2010;222(1):40-4.https://doi.org/10.1055/s-0029-1220942 | Excluded | Lack of relation between the title and the abstract of the article with the subject under study. |
|  | Echouffo-Tcheugui JB, Kengne AP. Chronic non-communicable diseases in Cameroon - burden, determinants and current policies. Globalization and Health. 2011;7.https://doi.org/10.1186/1744-8603-7-44 | Excluded | Lack of relation between the title and the abstract of the article with the subject under study. |
|  | Eckenwiler L. Displacement and solidarity: An ethic of place-making. Bioethics. 2018;32(9):562-8.https://doi.org/10.1111/bioe.12538 | Excluded | Lack of relation between the title and the abstract of the article with the subject under study. |
|  | Edenborough FP, Borgo G, Knoop C, Lannefors L, Mackenzie WE, Madge S, et al. Guidelines for the management of pregnancy in women with cystic fibrosis. Journal of Cystic Fibrosis. 2008;7(SUPPL. 1):S2-S32.https://doi.org/10.1016/j.jcf.2007.10.001 | Excluded | Lack of relation between the title and the abstract of the article with the subject under study. |
|  | Effing TW. Developments in respiratory self-management interventions over the last two decades. Chronic Respiratory Disease. 2023;20.https://doi.org/10.1177/14799731231221819 | Excluded | Lack of relation between the title and the abstract of the article with the subject under study. |
|  | Egbuta C, Mason KP. Recognizing risks and optimizing perioperative care to reduce respiratory complications in the pediatric patient. Journal of Clinical Medicine. 2020;9(6):1-29.https://doi.org/10.3390/jcm9061942 | Excluded | Lack of relation between the title and the abstract of the article with the subject under study. |
|  | Ehling D. Oriental medicine: An introduction. Alternative Therapies in Health and Medicine. 2001;7(4):71-82 | Excluded | Lack of relation between the title and the abstract of the article with the subject under study. |
|  | Eid N, Buchheit J, Neuling M, Phelps H. Chest physiotherapy in review. Respiratory Care. 1991;36(4):270-82 | Excluded | Lack of relation between the title and the abstract of the article with the subject under study. |
|  | Elenius V, Chawes B, Malmberg PL, Adamiec A, Ruszczyński M, Feleszko W, et al. Lung function testing and inflammation markers for wheezing preschool children: A systematic review for the EAACI Clinical Practice Recommendations on Diagnostics of Preschool Wheeze. Pediatric Allergy and Immunology. 2021;32(3):501-13.https://doi.org/10.1111/pai.13418 | Excluded | Lack of relation between the title and the abstract of the article with the subject under study. |
|  | Elia D, Marinou A, Chetta A. Life-threatening asthma after heroin inhalation. A case report and a review of the literature. Acta Biomedica de l'Ateneo Parmense. 2010;81(1):63-7 | Excluded | Lack of relation between the title and the abstract of the article with the subject under study. |
|  | Elshof J, Duiverman ML. Clinical Evidence of Nasal High-Flow Therapy in Chronic Obstructive Pulmonary Disease Patients. Respiration. 2020;99(2):140-53.https://doi.org/10.1159/000505583 | Excluded | Lack of relation between the title and the abstract of the article with the subject under study. |
|  | Elzawy G, Petrasek P, Fatehi Hassanabad A. The Unique Case of Acute Limb Ischemia in a Patient With a Patent Foramen Ovale. Vascular and Endovascular Surgery. 2024;58(8):894-9.https://doi.org/10.1177/15385744241276615 | Excluded | Lack of relation between the title and the abstract of the article with the subject under study. |
|  | Eneli IU, Skybo T, Camargo CA, Jr. Weight loss and asthma: a systematic review. Thorax. 2008;63(8):671-6.https://doi.org/10.1136/thx.2007.086470 | Excluded | Lack of relation between the title and the abstract of the article with the subject under study. |
|  | Engler D, Malan L. Antihistamines and allergies - An update. SA Pharmaceutical Journal. 2017;84(5):24-34 | Excluded | Lack of relation between the title and the abstract of the article with the subject under study. |
|  | Erdogan M, Esatoglu SN, Hatemi G, Hamuryudan V. Aortic involvement in relapsing polychondritis: case-based review. Rheumatology International. 2021;41(4):827-37.https://doi.org/10.1007/s00296-019-04468-5 | Excluded | Lack of relation between the title and the abstract of the article with the subject under study. |
|  | Ernst E. Spinal manipulation for asthma: a systematic review of randomised clinical trials. Respiratory Medicine 2009 Dec;103(12):1791-1795. 2009 | Excluded | Lack of relation between the title and the abstract of the article with the subject under study. |
|  | Ernst E. Is reflexology an effective intervention? A systematic review of randomised controlled trials. The Medical Journal of Australia 2009 Sep;191(5):263-266. 2009 | Excluded | Lack of relation between the title and the abstract of the article with the subject under study. |
|  | Ernst E. Chiropractic spinal manipulation: What does the 'best' evidence show? Focus on Alternative and Complementary Therapies. 2012;17(4):202-6.https://doi.org/10.1111/j.2042-7166.2012.01175.x | Excluded | Lack of relation between the title and the abstract of the article with the subject under study. |
|  | Ernst E, Canter PH. A systematic review of systematic reviews of spinal manipulation. Journal of the Royal Society of Medicine 2006 Apr;99(4):192-196. 2006 | Excluded | Lack of relation between the title and the abstract of the article with the subject under study. |
|  | Ernst E, Harkness E. Spinal manipulation: a systematic review of sham-controlled, double-blind, randomized clinical trials. Journal of Pain and Symptom Management 2001 Oct;22(4):879-889. 2001 | Excluded | Lack of relation between the title and the abstract of the article with the subject under study. |
|  | Ernst E, Lee MS. How effective is yoga? A concise overview of systematic reviews. Focus on Alternative and Complementary Therapies. 2010;15(4):274-9.https://doi.org/10.1111/j.2042-7166.2010.01049.x | Excluded | Lack of relation between the title and the abstract of the article with the subject under study. |
|  | Eslick GD. Epidemiology and Risk Factors of Pediatric Chest Pain: A Systematic Review. Pediatric Clinics of North America. 2010;57(6):1211-+.https://doi.org/10.1016/j.pcl.2010.09.013 | Excluded | Lack of relation between the title and the abstract of the article with the subject under study. |
|  | Essa A, Macaraeg J, Jagan N, Kwon D, Randhawa S, Kruse M, et al. Review of Cases of E-Cigarette or Vaping Product Use-Associated Lung Injury (EVALI) and Brief Review of the Literature. Case Reports in Pulmonology. 2020;2020.https://doi.org/10.1155/2020/1090629 | Excluded | Lack of relation between the title and the abstract of the article with the subject under study. |
|  | Evangelista JA, Parsons M, Renneburg AK. Chest pain in children: diagnosis through history and physical examination. Journal of pediatric health care : official publication of National Association of Pediatric Nurse Associates & Practitioners. 2000;14(1):3-8.https://doi.org/10.1016/s0891-5245(00)70037-x | Excluded | Lack of relation between the title and the abstract of the article with the subject under study. |
|  | Evers KE, Cummins CO, Prochaska JO, Prochaska JM. Online health behavior and disease management programs: Are we ready for them? Are they ready for us? Journal of Medical Internet Research. 2005;7(3).https://doi.org/10.2196/jmir.7.3.e27 | Excluded | Lack of relation between the title and the abstract of the article with the subject under study. |
|  | Eves ND, Davidson WJ. Evidence-based risk assessment and recommendations for physical activity clearance: Respiratory disease. Applied Physiology, Nutrition and Metabolism. 2011;36(SUPPL.1):80-100.https://doi.org/10.1139/H10-087 | Excluded | Lack of relation between the title and the abstract of the article with the subject under study. |
|  | Eyigör S, Umay E. Dysphagia management during covid-19 pandemic: A review of the literature and international guidelines. Turkish Journal of Physical Medicine and Rehabilitation. 2021;67(3):267-74.https://doi.org/10.5606/tftrd.2021.8427 | Excluded | Lack of relation between the title and the abstract of the article with the subject under study. |
|  | Fahy AS, Chiu PPL. Airway Clearance in Tracheomalacia. Seminars in Pediatric Surgery. 2021;30(3).https://doi.org/10.1016/j.sempedsurg.2021.151061 | Excluded | Lack of relation between the title and the abstract of the article with the subject under study. |
|  | Fair FJ, Ford GL, Soltani H. Interventions for supporting the initiation and continuation of breastfeeding among women who are overweight or obese. Cochrane Database of Systematic Reviews. 2019(9).https://doi.org/10.1002/14651858.CD012099.pub2 | Excluded | Lack of relation between the title and the abstract of the article with the subject under study. |
|  | Farah CS, Salome CM. Asthma and obesity: A known association but unknown mechanism. Respirology. 2012;17(3):412-21.https://doi.org/10.1111/j.1440-1843.2011.02080.x | Excluded | Lack of relation between the title and the abstract of the article with the subject under study. |
|  | Farhadi F, Baradaran Rahimi V, Mohamadi N, Askari VR. Effects of rosmarinic acid, carnosic acid, rosmanol, carnosol, and ursolic acid on the pathogenesis of respiratory diseases. BioFactors. 2023;49(3):478-501.https://doi.org/10.1002/biof.1929 | Excluded | Lack of relation between the title and the abstract of the article with the subject under study. |
|  | Fathi H, Morice AH. Cough. Medicine. 2008;36(3):129-31.https://doi.org/10.1016/j.mpmed.2007.12.001 | Excluded | Lack of relation between the title and the abstract of the article with the subject under study. |
|  | Feig EH, Madva EN, Millstein RA, Zambrano J, Amonoo HL, Longley RM, et al. Can positive psychological interventions improve health behaviors? A systematic review of the literature. Preventive Medicine. 2022;163.https://doi.org/10.1016/j.ypmed.2022.107214 | Excluded | Lack of relation between the title and the abstract of the article with the subject under study. |
|  | Feleszko W, Dziekiewicz M, Wąsowicz A. Immunostimulation using bacterial antigens - Mechanism of action and clinical practice in viral respiratory tract infections. Pediatria i Medycyna Rodzinna. 2015;11(4):358-64.https://doi.org/10.15557/PiMR.2015.0033 | Excluded | Lack of relation between the title and the abstract of the article with the subject under study. |
|  | Felix JF, Bradfield JP, Monnereau C, Van Der Valk RJP, Stergiakouli E, Chesi A, et al. Genome-wide association analysis identifies three new susceptibility loci for childhood body mass index. Human Molecular Genetics. 2016;25(2):389-403.https://doi.org/10.1093/hmg/ddv472 | Excluded | Lack of relation between the title and the abstract of the article with the subject under study. |
|  | Feng S, Shao Z, Ju L, Zhang Y. Atopy, asthma, and risk of bladder cancer: Systematic review and meta-analysis of cohort studies. European Journal of Inflammation. 2021;19.https://doi.org/10.1177/20587392211016117 | Excluded | Lack of relation between the title and the abstract of the article with the subject under study. |
|  | Feng YS, Kohlmann T, Janssen MF, Buchholz I. Psychometric properties of the EQ-5D-5L: a systematic review of the literature. Quality of Life Research. 2021;30(3):647-73.https://doi.org/10.1007/s11136-020-02688-y | Excluded | Lack of relation between the title and the abstract of the article with the subject under study. |
|  | Feng Z, Zhang L, Wang Y, Guo H, Liu J. Efficacy and Safety of Bisoprolol in Patients with Chronic Obstructive Pulmonary Disease: A Systematic Review and Meta-Analysis. International journal of chronic obstructive pulmonary disease. 2023;18:3067-83.https://doi.org/10.2147/copd.S438930 | Excluded | Lack of relation between the title and the abstract of the article with the subject under study. |
|  | Fergeson JE, Patel SS, Lockey RF. Acute asthma, prognosis, and treatment. Journal of Allergy and Clinical Immunology. 2017;139(2):438-47.https://doi.org/10.1016/j.jaci.2016.06.054 | Excluded | Lack of relation between the title and the abstract of the article with the subject under study. |
|  | Fernández-López I, Peña-Otero D, Atín-Arratibel MDLÁ, Eguillor-Mutiloa M. Effects of Manual Therapy on the Diaphragm in the Musculoskeletal System: A Systematic Review. Archives of Physical Medicine and Rehabilitation. 2021;102(12):2402-15.https://doi.org/10.1016/j.apmr.2021.03.031 | Excluded | Lack of relation between the title and the abstract of the article with the subject under study. |
|  | Ferraro VA, Zanconato S, Carraro S. Impact of COVID-19 in Children with Chronic Lung Diseases. International Journal of Environmental Research and Public Health. 2022;19(18).https://doi.org/10.3390/ijerph191811483 | Excluded | Lack of relation between the title and the abstract of the article with the subject under study. |
|  | Ferreira IM, Brooks D, White J, Goldstein R. Nutritional supplementation for stable chronic obstructive pulmonary disease. Cochrane database of systematic reviews (Online). 2012;12:CD000998 | Excluded | Lack of relation between the title and the abstract of the article with the subject under study. |
|  | Ferreira P, Ferreira M, Maher C, Hopper J, Huxley R, Alcantara C, et al. What is the research involving twins and low back pain telling US? A systematic review. Twin Research and Human Genetics. 2010;13(3):257 | Excluded | Lack of relation between the title and the abstract of the article with the subject under study. |
|  | Ferreira PH, Ferreira M, Maher C, Hopper J, Huxley R, Alcantara C, et al. What is the research involving twins and low back pain telling us? a systematic review. Physiotherapy (United Kingdom). 2011;97:eS340.https://doi.org/10.1016/j.physio.2011.04.002 | Excluded | Lack of relation between the title and the abstract of the article with the subject under study. |
|  | Fibbiani M, Di Rorà LGL, Novelli T, Peroni DG. The impact of human milk oligosaccharides on health from infancy to childhood. Minerva Pediatrics. 2022;74(6):724-32.https://doi.org/10.23736/s2724-5276.22.07037-9 | Excluded | Lack of relation between the title and the abstract of the article with the subject under study. |
|  | Fieten KB, Drijver-Messelink MT, Cogo A, Charpin D, Sokolowska M, Agache I, et al. Alpine altitude climate treatment for severe and uncontrolled asthma: An EAACI position paper. Allergy: European Journal of Allergy and Clinical Immunology. 2022;77(7):1991-2024.https://doi.org/10.1111/all.15242 | Excluded | Lack of relation between the title and the abstract of the article with the subject under study. |
|  | Filip-Ciubotaru F, Pandele GI, Foia L. Complementary therapies in the treatment of bronchial asthma. Revista medico-chirurgicala a Societaţii de Medici ş̧i Naturaliş̧ti din Iaş̧i. 2005;109(3):478-82 | Excluded | Lack of relation between the title and the abstract of the article with the subject under study. |
|  | Fishwick D. Work aggravated asthma; A review of the recent evidence. British Medical Bulletin. 2014;110(1):77-88.https://doi.org/10.1093/bmb/ldu004 | Excluded | Lack of relation between the title and the abstract of the article with the subject under study. |
|  | Fisk MZ, Steigerwald MD, Smoliga JM, Rundell KW. Asthma in swimmers: A review of the current literature. Physician and Sportsmedicine. 2010;38(4):28-34.https://doi.org/10.3810/psm.2010.12.1822 | Excluded | Lack of relation between the title and the abstract of the article with the subject under study. |
|  | Fitzgerald DA. Mini-symposium: Childhood obesity and its impact on respiratory wellbeing. Editorial title: Childhood obesity is the global warming of healthcare. Paediatric Respiratory Reviews. 2014;15(3):209-10.https://doi.org/10.1016/j.prrv.2014.08.001 | Excluded | Lack of relation between the title and the abstract of the article with the subject under study. |
|  | Folinsbee LJ. Does nitrogen dioxide exposure increase airways responsiveness? Toxicology and Industrial Health. 1992;8(5):273-83.https://doi.org/10.1177/074823379200800505 | Excluded | Lack of relation between the title and the abstract of the article with the subject under study. |
|  | Folletti I, Paolocci G, Murgia N, Abraha I, Dell'Omo M, Gambelunghe A, et al. Indoor occupational risk-factor in nonindustrial settings and work-related asthma. A systematic review. Allergy: European Journal of Allergy and Clinical Immunology. 2015;70:93-4.https://doi.org/10.1111/all.12715 | Excluded | Lack of relation between the title and the abstract of the article with the subject under study. |
|  | Fong KC, Hart JE, James P. A Review of Epidemiologic Studies on Greenness and Health: Updated Literature Through 2017. Current environmental health reports. 2018;5(1):77-87.https://doi.org/10.1007/s40572-018-0179-y | Excluded | Lack of relation between the title and the abstract of the article with the subject under study. |
|  | Forbes A, While A, Ullman R, Murgatroyd B. The contribution of nurses to child health and child health services: Findings of a scoping exercise. Journal of Child Health Care. 2007;11(3):231-47.https://doi.org/10.1177/1367493507079570 | Excluded | Lack of relation between the title and the abstract of the article with the subject under study. |
|  | Forte GC, Richter da Silva DT, Hennemann ML, Sarmento RA, Almeida JC, Roth Dalcin PdT. Diet effects in the asthma treatment: A systematic review. Critical Reviews in Food Science and Nutrition. 2018;58(11):1878-87.https://doi.org/10.1080/10408398.2017.1289893 | Excluded | Lack of relation between the title and the abstract of the article with the subject under study. |
|  | Fowler SJ, Pantin CT. A systematic approach to assessing complex breathlessness. ERS Monograph. 2022;2022(97):61-74.https://doi.org/10.1183/2312508X.10012921 | Excluded | Lack of relation between the title and the abstract of the article with the subject under study. |
|  | Francis DO, Sharda R, Patel D, Hovis KL, Penson D, Feurer I, et al. Developmental characteristics of extraesophageal reflux-related patient-reported outcome measures: A systematic review. Gastroenterology. 2016;150(4):S267-S8 | Excluded | Lack of relation between the title and the abstract of the article with the subject under study. |
|  | Frazier MD, Cheifetz IM. The Role of Heliox in Paediatric Respiratory Disease. Paediatric Respiratory Reviews. 2010;11(1):46-53.https://doi.org/10.1016/j.prrv.2009.10.008 | Excluded | Lack of relation between the title and the abstract of the article with the subject under study. |
|  | Freeman RJ, States LJ, Lewandowski SA, Singer DE, Patankar SN, Niebuhr DW. ACPM Position Statement: Air Pollution and Environmental Justice. American Journal of Preventive Medicine. 2024;67(5):792-800.https://doi.org/10.1016/j.amepre.2024.07.003 | Excluded | Lack of relation between the title and the abstract of the article with the subject under study. |
|  | Frent S, Calarasu C, Suska K, Gashynova K, Keir H. Ers international congress 2020: Highlights from the respiratory infections assembly. ERJ Open Research. 2021;7(2).https://doi.org/10.1183/23120541.00091-2021 | Excluded | Lack of relation between the title and the abstract of the article with the subject under study. |
|  | Freund KM, Dolan NC, Nelson HD. Update in women's health. Annals of Internal Medicine. 2003;138(2):119-27.https://doi.org/10.7326/0003-4819-138-2-200301210-00013 | Excluded | Lack of relation between the title and the abstract of the article with the subject under study. |
|  | Fronczek J, Gilbert JD, Byard RW. Forensic issues arising in the assessment of chlorine-related deaths in a domestic setting. Medicine, Science and the Law. 2021;61(3):232-5.https://doi.org/10.1177/00258024211002737 | Excluded | Lack of relation between the title and the abstract of the article with the subject under study. |
|  | Fu XL, Qian Y, Jin XH, Yu HR, Du L, Wu H, et al. COVID-19 in patients with systemic lupus erythematosus: A systematic review. Lupus. 2022;31(6):684-96.https://doi.org/10.1177/09612033221093502 | Excluded | Lack of relation between the title and the abstract of the article with the subject under study. |
|  | Fuentes-Aspe R, Gutierrez-Arias R, González-Seguel F, Marzuca-Nassr GN, Torres-Castro R, Najum-Flores J, et al. Which factors are associated with acquired weakness in the ICU? An overview of systematic reviews and meta-analyses. Journal of Intensive Care. 2024;12(1).https://doi.org/10.1186/s40560-024-00744-0 | Excluded | Lack of relation between the title and the abstract of the article with the subject under study. |
|  | Fujino N, Sugiura H. ACO (Asthma-COPD Overlap) Is Independent from COPD, a Case in Favor: A Systematic Review. Diagnostics. 2021;11(5).https://doi.org/10.3390/diagnostics11050859 | Excluded | Lack of relation between the title and the abstract of the article with the subject under study. |
|  | Gaillard EA, Kuehni CE, Turner S, Goutaki M, Holden KA, de Jong CCM, et al. European respiratory society clinical practice guidelines for the diagnosis of asthma in children aged. European Respiratory Journal. 2021;58(5).https://doi.org/10.1183/13993003.04173-2020 | Excluded | Lack of relation between the title and the abstract of the article with the subject under study. |
|  | Galant SP, Komarow HD, Shin HW, Siddiqui S, Lipworth BJ. The case for impulse oscillometry in the management of asthma in children and adults. Annals of Allergy, Asthma and Immunology. 2017;118(6):664-71.https://doi.org/10.1016/j.anai.2017.04.009 | Excluded | Lack of relation between the title and the abstract of the article with the subject under study. |
|  | Galantino ML, Boothroyd C, Lucci S. Complementary and alternative medicine interventions for the orthopedic patient: A review of the literature. Seminars in Integrative Medicine. 2003;1(2):65-79.https://doi.org/10.1016/S1543-1150(03)00009-7 | Excluded | Lack of relation between the title and the abstract of the article with the subject under study. |
|  | Galipeau J, Pussegoda K, Stevens A, Brehaut JC, Curran J, Forster AJ, et al. Effectiveness and Safety of Short-stay Units in the Emergency Department: A Systematic Review. Academic Emergency Medicine. 2015;22(8):893-907.https://doi.org/10.1111/acem.12730 | Excluded | Lack of relation between the title and the abstract of the article with the subject under study. |
|  | Gallagher E, Alvarez E, Jin L, Guenter D, Hatcher L, Furlan A. Patient contracts for chronic medical conditions Scoping review. Canadian Family Physician. 2022;68(5):E169-E77.https://doi.org/10.46747/cfp.6805e169 | Excluded | Lack of relation between the title and the abstract of the article with the subject under study. |
|  | Gandam H, Patel A, Rico Mora D, Walton L. Persistent intracranial hypotension after epidural for labor analgesia. Journal of Neurosurgical Anesthesiology. 2017;29(4):560.https://doi.org/10.1097/ANA.0000000000000452 | Excluded | Lack of relation between the title and the abstract of the article with the subject under study. |
|  | Gangat N, Szuber N, Pardanani A, Tefferi A. JAK2 unmutated erythrocytosis: current diagnostic approach and therapeutic views. Leukemia. 2021;35(8):2166-81.https://doi.org/10.1038/s41375-021-01290-6 | Excluded | Lack of relation between the title and the abstract of the article with the subject under study. |
|  | Garcia-Marcos L, Edwards J, Kennington E, Aurora P, Baraldi E, Carraro S, et al. Priorities for future research into asthma diagnostic tools: A PAN-EU consensus exercise from the European asthma research innovation partnership (EARIP). Clinical and Experimental Allergy. 2018;48(2):104-20.https://doi.org/10.1111/cea.13080 | Excluded | Lack of relation between the title and the abstract of the article with the subject under study. |
|  | Garcia-Rio F, Alvarez-Puebla MJ, Esteban-Gorgojo I, Barranco P, Olaguibel JM. Obesity and asthma: Key clinical questions. Journal of Investigational Allergology and Clinical Immunology. 2019;29(4):262-71.https://doi.org/10.18176/jiaci.0316 | Excluded | Lack of relation between the title and the abstract of the article with the subject under study. |
|  | Garlipp DC, Guimaraes RB, Savaris SL, Junior CF, Dutra O, Leiria TLL. Physical Activity and Incidence of Atrial Fibrillation-Systematic Review and Meta-Analysis. International Journal of Cardiovascular Sciences. 2019;32(4):384-90.https://doi.org/10.5935/2359-4802.20190055 | Excluded | Lack of relation between the title and the abstract of the article with the subject under study. |
|  | Garrod R, Lasserson T. Role of physiotherapy in the management of chronic lung diseases: an overview of systematic reviews. Respir Med. 2007;101(12):2429-36.https://doi.org/10.1016/j.rmed.2007.06.007 | Excluded | Lack of relation between the title and the abstract of the article with the subject under study. |
|  | Garvey WT, Mechanick JI, Brett EM, Garber AJ, Hurley DL, Jastreboff AM, et al. American association of clinical endocrinologists and American college of endocrinology comprehensive clinical practice guidelines for medical care of patients with obesity: Executive summary. Endocrine Practice. 2016;22(7):842-84.https://doi.org/10.4158/EP161356.ESGL | Excluded | Lack of relation between the title and the abstract of the article with the subject under study. |
|  | Gasser CRB, Pellaton R, Rochat CP. Pediatric spontaneous pneumomediastinum: Narrative literature review. Pediatric Emergency Care. 2017;33(5):370-4.https://doi.org/10.1097/PEC.0000000000000625 | Excluded | Lack of relation between the title and the abstract of the article with the subject under study. |
|  | Gaw R, Yap C, Newhouse SM, Aiyappan V. Clinical conundrums: How safe is exercise in patients with asthma and is high-flow nasal oxygen useful in respiratory failure? American Journal of Respiratory and Critical Care Medicine. 2016;194(5):631-3.https://doi.org/10.1164/rccm.201511-2214RR | Excluded | Lack of relation between the title and the abstract of the article with the subject under study. |
|  | Gayes LA, Steele RG. A meta-analysis of motivational interviewing interventions for pediatric health behavior change. Journal of Consulting and Clinical Psychology. 2014;82(3):521-35.https://doi.org/10.1037/a0035917 | Excluded | Lack of relation between the title and the abstract of the article with the subject under study. |
|  | Gebresillassie BM, Attia JR, Mersha AG, Harris ML. Prognostic models and factors identifying end-of-life in non-cancer chronic diseases: a systematic review. BMJ Supportive and Palliative Care. 2024;14(e3):e2316-e29.https://doi.org/10.1136/spcare-2023-004656 | Excluded | Lack of relation between the title and the abstract of the article with the subject under study. |
|  | Geoghegan L, Scarborough A, Wormald JCR, Harrison CJ, Collins D, Gardiner M, et al. Automated conversational agents for post-intervention follow-up: A systematic review. BJS Open. 2021;5(4).https://doi.org/10.1093/bjsopen/zrab070 | Excluded | Lack of relation between the title and the abstract of the article with the subject under study. |
|  | George M. Health beliefs, treatment preferences and complementary and alternative medicine for asthma, smoking and lung cancer self-management in diverse Black communities. Patient Education and Counseling. 2012;89(3):489-500.https://doi.org/10.1016/j.pec.2012.05.003 | Excluded | Lack of relation between the title and the abstract of the article with the subject under study. |
|  | Geramas I, Terzakis D, Hatzimanolis E, Georgalas C. Social Factors in the Development of Chronic Rhinosinusitis: a Systematic Review. Current Allergy and Asthma Reports. 2018;18(2).https://doi.org/10.1007/s11882-018-0763-0 | Excluded | Lack of relation between the title and the abstract of the article with the subject under study. |
|  | Ghiani H. Obesity and asthma: Physiopathological implications. Salud(i)Ciencia. 2010;17(8):764-6 | Excluded | Lack of relation between the title and the abstract of the article with the subject under study. |
|  | Gholami SK, Santiago C, Bhojaraja VS, Michael LYS, Radhakrishnan AK, Mehta DH. MIND-BODY THERAPIES AND ITS EFFECT ON THE IMMUNE SYSTEM IN CHRONIC DISEASES: A LITERATURE REVIEW. Journal of Health and Translational Medicine. 2022;25(1):97-107.https://doi.org/10.22452/jummec.vol25no1.16 | Excluded | Lack of relation between the title and the abstract of the article with the subject under study. |
|  | Ghoshouni H, Rafiei N, Panah MY, Firouzabadi DD, Mahmoudi F, Asghariahmadabad M, et al. Asthma and chronic obstructive pulmonary disease (COPD) in people with multiple sclerosis: A systematic review and meta-analysis. Multiple Sclerosis and Related Disorders. 2024;85.https://doi.org/10.1016/j.msard.2024.105546 | Excluded | Lack of relation between the title and the abstract of the article with the subject under study. |
|  | Giangregorio F, Mosconi E, Debellis MG, Provini S, Esposito C, Garolfi M, et al. A Systematic Review of Metabolic Syndrome: Key Correlated Pathologies and Non-Invasive Diagnostic Approaches. Journal of Clinical Medicine. 2024;13(19).https://doi.org/10.3390/jcm13195880 | Excluded | Lack of relation between the title and the abstract of the article with the subject under study. |
|  | Gibbaoui H, Abouchacra S, Yaman M. A case of primary diffuse tracheobronchial amyloidosis. Annals of Thoracic Surgery. 2004;77(5):1832-4.https://doi.org/10.1016/S0003-4975(03)00999-8 | Excluded | Lack of relation between the title and the abstract of the article with the subject under study. |
|  | Gibbons MC, Wilson RF, Samal L, Lehman CU, Dickersin K, Lehmann HP, et al. Impact of consumer health informatics applications. Evidence report/technology assessment. 2009(188):1-546 | Excluded | Lack of relation between the title and the abstract of the article with the subject under study. |
|  | Gibson PG, McDonald VM. Asthma-COPD overlap 2015: <i>now we are six</i>. Thorax. 2015;70(7):683-91.https://doi.org/10.1136/thoraxjnl-2014-206740 | Excluded | Lack of relation between the title and the abstract of the article with the subject under study. |
|  | Gibson PG, Powell H, Coughlan J, Wilson AJ, Abramson M, Haywood P, et al. Self-management education and regular practitioner review for adults with asthma (Cochrane review) [with consumer summary]. Cochrane Database of Systematic Reviews 2002;Issue 3. 2002 | Excluded | Lack of relation between the title and the abstract of the article with the subject under study. |
|  | Gibson PG, Powell H, Coughlan J, Wilson AJ, Abramson M, Haywood P, et al. Self-management education and regular practitioner review for adults with asthma. Cochrane database of systematic reviews (Online). 2003(1):CD001117 | Excluded | Lack of relation between the title and the abstract of the article with the subject under study. |
|  | Gibson PG, Powell H, Coughlan J, Wilson AJ, Hensley MJ, Abramson M, et al. Limited (information only) patient education programs for adults with asthma (Cochrane review) [with consumer summary]. Cochrane Database of Systematic Reviews 2002;Issue 1. 2002 | Excluded | Lack of relation between the title and the abstract of the article with the subject under study. |
|  | Gibson PG, Powell H, Wilson A, Abramson MJ, Haywood P, Bauman A, et al. Self‐management education and regular practitioner review for adults with asthma. Cochrane Database of Systematic Reviews. 2002(3).https://doi.org/10.1002/14651858.CD001117 | Excluded | Lack of relation between the title and the abstract of the article with the subject under study. |
|  | Giggins OM, Persson UM, Caulfield B. Biofeedback in rehabilitation. Journal of neuroengineering and rehabilitation. 2013;10:60 | Excluded | Lack of relation between the title and the abstract of the article with the subject under study. |
|  | Gilad J, Pirogovsky A, Bartal C. Unmasking of carnitine palmitoyltransferase deficiency during an acute exacerbation of asthma complicated by rhabdomyolysis in a soldier. Military Medicine. 2004;169(10):821-3.https://doi.org/10.7205/MILMED.169.10.821 | Excluded | Lack of relation between the title and the abstract of the article with the subject under study. |
|  | Gill P, Dowell AC, Neal RD, Smith N, Heywood P, Wilson AE. Evidence based general practice: A retrospective study of interventions in one training practice. British Medical Journal. 1996;312(7034):819-21.https://doi.org/10.1136/bmj.312.7034.819 | Excluded | Lack of relation between the title and the abstract of the article with the subject under study. |
|  | Gimenez LM, Zafra H. Vocal cord dysfunction: An update. Annals of Allergy, Asthma and Immunology. 2011;106(4):267-74.https://doi.org/10.1016/j.anai.2010.09.004 | Excluded | Lack of relation between the title and the abstract of the article with the subject under study. |
|  | Gipsman AI, Feld L, Johnson B, Needleman JP, Boas H, Lin N, et al. Eosinophilic plastic bronchitis: Case series and review of the literature. Pediatric Pulmonology. 2023;58(11):3023-31.https://doi.org/10.1002/ppul.26650 | Excluded | Lack of relation between the title and the abstract of the article with the subject under study. |
|  | Gladstone JP, Dodick DW. Current and emerging treatment options for migraine and other primary headache disorders. Expert Review of Neurotherapeutics. 2003;3(6):845-72 | Excluded | Lack of relation between the title and the abstract of the article with the subject under study. |
|  | Gleberzon BJ, Arts J, Mei A, McManus EL. The use of spinal manipulative therapy for pediatric health conditions: a systematic review of the literature. Journal of the Canadian Chiropractic Association 2012 Jun;56(2):128-141. 2012 | Excluded | Lack of relation between the title and the abstract of the article with the subject under study. |
|  | Glushko T, Seifert R, Brown F, Vigilance D, Iriarte B, Teytelboym OM. Transseptal course of anomalous left main coronary artery originating from single right coronary orifice presenting as unstable angina. Radiology Case Reports. 2018;13(3):549-54.https://doi.org/10.1016/j.radcr.2018.02.009 | Excluded | Lack of relation between the title and the abstract of the article with the subject under study. |
|  | Goeman DP, Douglass JA. Optimal management of asthma in elderly patients: Strategies to improve adherence to recommended interventions. Drugs and Aging. 2007;24(5):381-94.https://doi.org/10.2165/00002512-200724050-00003 | Excluded | Lack of relation between the title and the abstract of the article with the subject under study. |
|  | Göhl AO, Pleyer K, Biberger C, Taube K, Müller C, Worth H. Recommendations for planning and realisation of exercise training in outpatient lung sports groups. Pneumologie. 2006;60(11):716-23.https://doi.org/10.1055/s-2006-944318 | Excluded | Lack of relation between the title and the abstract of the article with the subject under study. |
|  | Göhl O, Walker DJ, Walterspacher S, Langer D, Spengler CM, Wanke T, et al. Respiratory Muscle Training: State of the Art. Pneumologie. 2016;70(1):37-48.https://doi.org/10.1055/s-0041-109312 | Excluded | Lack of relation between the title and the abstract of the article with the subject under study. |
|  | Goldstein S, Weinberg JM. Recurrent and persistent urticaria: Is it chronic idiopathic urticaria?: Narrative review on diagnosis and management. Journal of the Dermatology Nurses' Association. 2018;10(6):279-89.https://doi.org/10.1097/JDN.0000000000000439 | Excluded | Lack of relation between the title and the abstract of the article with the subject under study. |
|  | Golzar Y, Doukky R. Regadenoson use in patients with chronic obstructive pulmonary disease: The state of current knowledge. International Journal of COPD. 2014;9:129-37.https://doi.org/10.2147/COPD.S56879 | Excluded | Lack of relation between the title and the abstract of the article with the subject under study. |
|  | Gonzalez ME, Burk CJ, Barbouth DS, Connelly EA. Macrocephaly-capillary malformation: A report of three cases and review of the literature. Pediatric Dermatology. 2009;26(3):342-6.https://doi.org/10.1111/j.1525-1470.2009.00924.x | Excluded | Lack of relation between the title and the abstract of the article with the subject under study. |
|  | Goodman JE, Chandalia JK, Thakali S, Seeley M. Meta-analysis of nitrogen dioxide exposure and airway hyper-responsiveness in asthmatics. Critical Reviews in Toxicology. 2009;39(9):719-42.https://doi.org/10.3109/10408440903283641 | Excluded | Lack of relation between the title and the abstract of the article with the subject under study. |
|  | Goodman JE, Kennedy EM, Seeley M. Do individuals with asthma experience airway hyper-responsiveness after exposure to nitrogen dioxide? Regulatory Toxicology and Pharmacology. 2017;89:279-87.https://doi.org/10.1016/j.yrtph.2017.07.021 | Excluded | Lack of relation between the title and the abstract of the article with the subject under study. |
|  | Goodman N, Campbell S, Tong M, Cameron D, Brain M, Borchers Arriagada N, et al. Interventions for reducing exposure to air pollution from landscape fires in a changing environment: A systematic review. Science of the Total Environment. 2025;966.https://doi.org/10.1016/j.scitotenv.2025.178621 | Excluded | Lack of relation between the title and the abstract of the article with the subject under study. |
|  | Gopinath H, Shivashankar M. Herbo-metallic Indian nano-medicine Abhrak Bhasma (MICA): A periodical review. Research Journal of Pharmaceutical, Biological and Chemical Sciences. 2016;7(6):2373-81 | Excluded | Lack of relation between the title and the abstract of the article with the subject under study. |
|  | Gordon JB. The importance of child abuse and neglect in adult medicine. Pharmacology Biochemistry and Behavior. 2021;211.https://doi.org/10.1016/j.pbb.2021.173268 | Excluded | Lack of relation between the title and the abstract of the article with the subject under study. |
|  | Gorji N, Moeini R. Singing as a rehabilitation method from the viewpoint of Avicenna (980-1037 AD). npj Primary Care Respiratory Medicine. 2017;27(1).https://doi.org/10.1038/s41533-017-0021-2 | Excluded | Lack of relation between the title and the abstract of the article with the subject under study. |
|  | Gosselink HAAM, Wagenaar RC. Efficacy of breathing exercises in chronic obstructive pulmonary disease and asthma. A meta-analysis of the literature. Journal of Rehabilitation Sciences. 1993;6(3):66-79 | Excluded | Lack of relation between the title and the abstract of the article with the subject under study. |
|  | Gotshall RW. Airway response during exercise and hyperpnoea in non-asthmatic and asthmatic individuals. Sports Medicine. 2006;36(6):513-27.https://doi.org/10.2165/00007256-200636060-00005 | Excluded | Lack of relation between the title and the abstract of the article with the subject under study. |
|  | Gotua M, Lomidze N, Dolidze N, Gotua T. IgE-mediated food hypersensitivity disorders. Georgian medical news. 2008(157):39-44 | Excluded | Lack of relation between the title and the abstract of the article with the subject under study. |
|  | Gouws A. Severe atopic dermatitis/eczema - a review of the literature. Current Allergy and Clinical Immunology. 2012;25(3):140-4 | Excluded | Lack of relation between the title and the abstract of the article with the subject under study. |
|  | Gozal D, Rubin BK. Introduction to CIPP V: A paediatric forum for respiratory disease. Paediatric Respiratory Reviews. 2004;5(SUPPL. A):S1.https://doi.org/10.1016/S1526-0542(04)90000-0 | Excluded | Lack of relation between the title and the abstract of the article with the subject under study. |
|  | Granger CL, Connolly B, Denehy L, Hart N, Antippa P, Lin KY, et al. Understanding factors influencing physical activity and exercise in lung cancer: a systematic review. Supportive Care in Cancer. 2017;25(3):983-99.https://doi.org/10.1007/s00520-016-3484-8 | Excluded | Lack of relation between the title and the abstract of the article with the subject under study. |
|  | Grant WB, Wimalawansa SJ, Holick MF, Cannell JJ, Pludowski P, Lappe JM, et al. Emphasizing the health benefits of vitamin D for those with neurodevelopmental disorders and intellectual disabilities. Nutrients. 2015;7(3):1538-64.https://doi.org/10.3390/nu7031538 | Excluded | Lack of relation between the title and the abstract of the article with the subject under study. |
|  | Green RH, Brightling CE, Pavord ID, Wardlaw AJ. Management of asthma in adults: Current therapy and future directions. Postgraduate Medical Journal. 2003;79(931):259-67.https://doi.org/10.1136/pmj.79.931.259 | Excluded | Lack of relation between the title and the abstract of the article with the subject under study. |
|  | Griffith RJ, Alsweiler J, Moore AE, Brown S, Middleton P, Shepherd E, et al. Interventions to prevent women from developing gestational diabetes mellitus: an overview of Cochrane reviews (Cochrane review) [with consumer summary]. Cochrane Database of Systematic Reviews 2020;Issue 6. 2020 | Excluded | Lack of relation between the title and the abstract of the article with the subject under study. |
|  | Grigg J. Outdoor air pollution and children's health. Pediatric Pulmonology. 2018;53:S52-S3.https://doi.org/10.1002/ppul.24031 | Excluded | Lack of relation between the title and the abstract of the article with the subject under study. |
|  | Grillo LJF, Housley GM, Gangadharan S, Majid A, Hull JH. Physiotherapy for large airway collapse: an ABC approach. ERJ Open Research. 2022;8(1).https://doi.org/10.1183/23120541.00510-2021 | Excluded | Lack of relation between the title and the abstract of the article with the subject under study. |
|  | Grinevica A, Udre A, Balodis A, Strumfa I. Tic Cough in an Adolescent with Organic Brain Pathology—A Case Report and Literature Review. Brain Sciences. 2024;14(1).https://doi.org/10.3390/brainsci14010079 | Excluded | Lack of relation between the title and the abstract of the article with the subject under study. |
|  | Gross AR, Olson KA, Pool J, Basson A, Clewley D, Dice JL, et al. Spinal manipulation and mobilisation in paediatrics–an international evidence-based position statement for physiotherapists. Journal of Manual and Manipulative Therapy. 2024;32(3):211-33.https://doi.org/10.1080/10669817.2024.2332026 | Excluded | Lack of relation between the title and the abstract of the article with the subject under study. |
|  | Gross N, Levin D. Primary Care of the Patient with Chronic Obstructive Pulmonary Disease-Part 2: Pharmacologic Treatment Across All Stages of Disease. American Journal of Medicine. 2008;121(7 SUPPL. 1):S13-S24.https://doi.org/10.1016/j.amjmed.2008.04.003 | Excluded | Lack of relation between the title and the abstract of the article with the subject under study. |
|  | Gruber W, Eber E, Zach M. Alternative medicine and bronchial asthma - A review from a paediatric perspective. Monatsschrift fur Kinderheilkunde. 1997;145(8):786-96.https://doi.org/10.1007/s001120050178 | Excluded | Lack of relation between the title and the abstract of the article with the subject under study. |
|  | Grundy Q. A Review of the Quality and Impact of Mobile Health Apps. Annual Review of Public Health. 2022;43:117-34.https://doi.org/10.1146/annurev-publhealth-052020-103738 | Excluded | Lack of relation between the title and the abstract of the article with the subject under study. |
|  | Gu C, Yu Y, Chen Y, Duan S, Xu R, Liu S, et al. Effect of acupoint catgut embedding combined with western medicine on patients with stable COPD: Acupoint catgut embedding treating stable COPD meta-analysis. Medicine (United States). 2023;102(41):E35281.https://doi.org/10.1097/MD.0000000000035281 | Excluded | Lack of relation between the title and the abstract of the article with the subject under study. |
|  | Gu Y, Fujitomo Y, Ohmagari N. Outcomes and future prospect of Japan’s national action plan on antimicrobial resistance (2016–2020). Antibiotics. 2021;10(11).https://doi.org/10.3390/antibiotics10111293 | Excluded | Lack of relation between the title and the abstract of the article with the subject under study. |
|  | Gudbjartsson T, Gudmundsson G. Middle lobe syndrome: A review of clinicopathological features, diagnosis and treatment. Respiration. 2012;84(1):80-6.https://doi.org/10.1159/000336238 | Excluded | Lack of relation between the title and the abstract of the article with the subject under study. |
|  | Gulen T, Akin C. Idiopathic Anaphylaxis: a Perplexing Diagnostic Challenge for Allergists. Current Allergy and Asthma Reports. 2021;21(2).https://doi.org/10.1007/s11882-021-00988-y | Excluded | Lack of relation between the title and the abstract of the article with the subject under study. |
|  | Gupta S, Goodridge D, Pakhalé S, McIntyre K, Pendharkar SR. Choosing wisely: The Canadian Thoracic Society's list of six things that physicians and patients should question. Canadian Journal of Respiratory, Critical Care, and Sleep Medicine. 2017;1(2):54-61.https://doi.org/10.1080/24745332.2017.1331666 | Excluded | Lack of relation between the title and the abstract of the article with the subject under study. |
|  | Gupta VK, Shobha P, Maria AK, Narang VK, Arora S, Gupta V, et al. To study the prescription pattern of inhaler devices and medication in management of chronic obstructive pulmonary disease in primary care practice. Respirology. 2013;18:22-3.https://doi.org/10.1111/resp.12042 | Excluded | Lack of relation between the title and the abstract of the article with the subject under study. |
|  | Gyorik SA, Brutsche MH. Complementary and alternative medicine for bronchial asthma: is there new evidence? Current Opinion in Pulmonary Medicine 2004 Jan;10(1):37-43. 2004 | Excluded | Lack of relation between the title and the abstract of the article with the subject under study. |
|  | Haahtela T, Bousquet J, Antó JM. From biodiversity to nature deficiency in human health and disease. Porto Biomedical Journal. 2024;9(1).https://doi.org/10.1097/j.pbj.0000000000000245 | Excluded | Lack of relation between the title and the abstract of the article with the subject under study. |
|  | Haahtela T, Holgate S, Pawankar R, Akdis CA, Benjaponpitak S, Caraballo L, et al. The biodiversity hypothesis and allergic disease: World allergy organization position statement. World Allergy Organization Journal. 2013;6(1).https://doi.org/10.1186/1939-4551-6-3 | Excluded | Lack of relation between the title and the abstract of the article with the subject under study. |
|  | Haines AJ, Mackenzie L, Honey A, Middleton PG. Occupations and balance during the transition to motherhood with a lifetime chronic illness: A scoping review examining cystic fibrosis, asthma, and Type-1 diabetes. Australian Occupational Therapy Journal. 2023;70(6):730-44.https://doi.org/10.1111/1440-1630.12899 | Excluded | Lack of relation between the title and the abstract of the article with the subject under study. |
|  | Haines AJ, Middleton PG. Pulmonary disorders in pregnancy: Bronchiectasis, cystic fibrosis, sarcoidosis and interstitial diseases. Best Practice and Research: Clinical Obstetrics and Gynaecology. 2022;85:114-26.https://doi.org/10.1016/j.bpobgyn.2022.09.001 | Excluded | Lack of relation between the title and the abstract of the article with the subject under study. |
|  | Haines J, Hull JH, Fowler SJ. Clinical presentation, assessment, and management of inducible laryngeal obstruction. Current Opinion in Otolaryngology and Head and Neck Surgery. 2018;26(3):174-9.https://doi.org/10.1097/MOO.0000000000000452 | Excluded | Lack of relation between the title and the abstract of the article with the subject under study. |
|  | Haines J, Smith JA, Wingfield-Digby J, King J, Yorke J, Fowler SJ. Systematic review of the effectiveness of non-pharmacological interventions used to treat adults with inducible laryngeal obstruction. BMJ Open Respiratory Research. 2022;9(1).https://doi.org/10.1136/bmjresp-2022-001199 | Excluded | Lack of relation between the title and the abstract of the article with the subject under study. |
|  | Hall KK, Petsky HL, Chang AB, O'Grady KF. Caseworker‐assigned discharge plans to prevent hospital readmission for acute exacerbations in children with chronic respiratory illness. Cochrane Database of Systematic Reviews. 2018(11).https://doi.org/10.1002/14651858.CD012315.pub2 | Excluded | Lack of relation between the title and the abstract of the article with the subject under study. |
|  | Hallal PC, Victora CG, Azevedo MR, Wells JCK. Adolescent physical activity and health - A systematic review. Sports Medicine. 2006;36(12):1019-30.https://doi.org/10.2165/00007256-200636120-00003 | Excluded | Lack of relation between the title and the abstract of the article with the subject under study. |
|  | Halpin DM, Kaplan AG, Russell RK. Why choose tiotropium for my patient? A comprehensive review of actions and outcomes versus other bronchodilators. Respiratory Medicine. 2017;128:28-41.https://doi.org/10.1016/j.rmed.2017.04.008 | Excluded | Lack of relation between the title and the abstract of the article with the subject under study. |
|  | Hamadneh M, Alquran A, Manna R. Impact of the COVID-19 on asthma control among children: A systematic review. Journal of Public Health Research. 2023;12(3).https://doi.org/10.1177/22799036231197186 | Excluded | Lack of relation between the title and the abstract of the article with the subject under study. |
|  | Hamasaki H. Effects of Diaphragmatic Breathing on Health: A Narrative Review. Medicines (Basel, Switzerland). 2020;7(10).https://doi.org/10.3390/medicines7100065 | Excluded | Lack of relation between the title and the abstract of the article with the subject under study. |
|  | Hambrook DW, Fink JN. Airbag asthma: A case report and review of the literature. Annals of Allergy, Asthma and Immunology. 2006;96(2):369-72.https://doi.org/10.1016/S1081-1206(10)61250-1 | Excluded | Lack of relation between the title and the abstract of the article with the subject under study. |
|  | Hammoudeh S, Gadelhak W, Janahi I. Asthma and obesity in the Middle East region: An overview. Annals of Thoracic Medicine. 2019;14(2):116-21.https://doi.org/10.4103/atm.ATM_115_18 | Excluded | Lack of relation between the title and the abstract of the article with the subject under study. |
|  | Hamre HJ, Glockmann A, Tröger W, Kienle GS, Kiene H. Assessing the order of magnitude of outcomes in single-arm cohorts through systematic comparison with corresponding cohorts: An example from the AMOS study. BMC Medical Research Methodology. 2008;8.https://doi.org/10.1186/1471-2288-8-11 | Excluded | Lack of relation between the title and the abstract of the article with the subject under study. |
|  | Hamre HJ, Kiene H, Ziegler R, Troger W, Meinecke C, Schnurer C, et al. Overview of the Publications From the Anthroposophic Medicine Outcomes Study (AMOS): A Whole System Evaluation Study. Global advances in health and medicine. 2014;3(1):54-70.https://doi.org/10.7453/gahmj.2013.010 | Excluded | Lack of relation between the title and the abstract of the article with the subject under study. |
|  | Han F, Wang Y, Wang Y, Dong J, Nie C, Chen M, et al. Intraoperative cardiac arrest. Medicine (United States). 2017;96(17).https://doi.org/10.1097/MD.0000000000006794 | Excluded | Lack of relation between the title and the abstract of the article with the subject under study. |
|  | Hanania NA. Evaluating the safety of COPD medications: An evidence-based review. Chest. 2013;144(4):1357-67.https://doi.org/10.1378/chest.12-2438 | Excluded | Lack of relation between the title and the abstract of the article with the subject under study. |
|  | Hancox RJ, Jones S, Baggott C, Chen D, Corna N, Davies C, et al. New Zealand COPD Guidelines: Quick Reference Guide. New Zealand Medical Journal. 2021;134(1530):76-110 | Excluded | Lack of relation between the title and the abstract of the article with the subject under study. |
|  | Hanlon P, Butterly EW, Shah ASV, Hannigan LJ, Lewsey J, Mair FS, et al. Treatment effect modification due to comorbidity: Individual participant data meta-analyses of 120 randomised controlled trials. PLoS Medicine. 2023;20(6).https://doi.org/10.1371/journal.pmed.1004176 | Excluded | Lack of relation between the title and the abstract of the article with the subject under study. |
|  | Hanlon P, Daines L, Campbell C, McKinstry B, Weller D, Pinnock H. Telehealth interventions to support self-management of long-term conditions: a systematic metareview of diabetes, heart failure, asthma, chronic obstructive pulmonary disease, and cancer. Journal of Medical Internet Research 2017 May;19(5):e172. 2017 | Excluded | Lack of relation between the title and the abstract of the article with the subject under study. |
|  | Hansen KE, Johnson MG. An update on Vitamin D for clinicians. Current Opinion in Endocrinology, Diabetes and Obesity. 2016;23(6):440-4.https://doi.org/10.1097/MED.0000000000000288 | Excluded | Lack of relation between the title and the abstract of the article with the subject under study. |
|  | Hansen LB, Vondracek SF. Prevention and treatment of nonpostmenopausal osteoporosis. American Journal of Health-System Pharmacy. 2004;61(24):2637-56.https://doi.org/10.1093/ajhp/61.24.2637 | Excluded | Lack of relation between the title and the abstract of the article with the subject under study. |
|  | Hansen TS, Poulsen I, Nørholm V, Loft MI, Jensen PS. Nutritional Support and Physical Activity Intervention Programs with a Person-Centred Approach in People with Chronic Obstructive Pulmonary Disease: a Scoping Review. International Journal of COPD. 2024;19:2193-216.https://doi.org/10.2147/COPD.S458289 | Excluded | Lack of relation between the title and the abstract of the article with the subject under study. |
|  | Haran M, Schattner A, Mate A, Starobin D, Haran G, Shtalrid M. Can a rare form of myasthenia gravis shed additional light on disease mechanisms? Clinical Neurology and Neurosurgery. 2013;115(5):562-6.https://doi.org/10.1016/j.clineuro.2012.06.038 | Excluded | Lack of relation between the title and the abstract of the article with the subject under study. |
|  | Harrison SL, Lee A, Janaudis-Ferreira T, Goldstein RS, Brooks D. Mindfulness in people with a respiratory diagnosis: A systematic review. Patient Education and Counseling. 2016;99(3):348-55.https://doi.org/10.1016/j.pec.2015.10.013 | Excluded | Lack of relation between the title and the abstract of the article with the subject under study. |
|  | Hartman JE, Garner JL, Shah PL, Slebos DJ. New bronchoscopic treatment modalities for patients with chronic bronchitis. European Respiratory Review. 2021;30(159):1-11.https://doi.org/10.1183/16000617.0281-2020 | Excluded | Lack of relation between the title and the abstract of the article with the subject under study. |
|  | Hartwig FP, Davies NM, Horta BL, Ahluwalia TS, Bisgaard H, Bønnelykke K, et al. Effect modification of FADS2 polymorphisms on the association between breastfeeding and intelligence: Results from a collaborative meta-analysis. International Journal of Epidemiology. 2019;48(1):45-57.https://doi.org/10.1093/ije/dyy273 | Excluded | Lack of relation between the title and the abstract of the article with the subject under study. |
|  | Hawk C, Adams J, Hartvigsen J. The role of CAM in public health, disease prevention, and health promotion. Evidence-based Complementary and Alternative Medicine. 2015;2015.https://doi.org/10.1155/2015/528487 | Excluded | Lack of relation between the title and the abstract of the article with the subject under study. |
|  | Hawk C, Khorsan R, Lisi AJ, Ferrance RJ, Evans MW. Chiropractic care for nonmusculoskeletal conditions: a systematic review with implications for whole systems research. Journal of Alternative & Complementary Medicine 2007 Jun;13(5):491-512. 2007 | Excluded | Lack of relation between the title and the abstract of the article with the subject under study. |
|  | Haworth S, Shungin D, Van Der Tas JT, Vucic S, Medina-Gomez C, Yakimov V, et al. Consortium-based genome-wide meta-analysis for childhood dental caries traits. Human Molecular Genetics. 2018;27(17):3113-27.https://doi.org/10.1093/hmg/ddy237 | Excluded | Lack of relation between the title and the abstract of the article with the subject under study. |
|  | Hay AD, Wilson A, Fahey T, Peters TJ. The duration of acute cough in pre-school children presenting to primary care: A prospective cohort study. Family Practice. 2003;20(6):696-705.https://doi.org/10.1093/fampra/cmg613 | Excluded | Lack of relation between the title and the abstract of the article with the subject under study. |
|  | Hay JA, Cairney J. Development of the habitual activity estimation scale for clinical research: A systematic approach. Pediatric Exercise Science. 2006;18(2):193-202.https://doi.org/10.1123/pes.18.2.193 | Excluded | Lack of relation between the title and the abstract of the article with the subject under study. |
|  | Hayati AA, Wan-Hitam WH, Cheong MT, Yunus R, Shatriah I. Optic neuritis in a child with biotinidase deficiency: Case report and literature review. Clinical Ophthalmology. 2012;6(1):389-95.https://doi.org/10.2147/OPTH.S29048 | Excluded | Lack of relation between the title and the abstract of the article with the subject under study. |
|  | Heaney LG, Lindsay JT, McGarvey LPA. Inflammation in chronic obstructive pulmonary disease: Implications for new treatment strategies. Current Medicinal Chemistry. 2007;14(7):787-96.https://doi.org/10.2174/092986707780090936 | Excluded | Lack of relation between the title and the abstract of the article with the subject under study. |
|  | Helenius IJ, Tikkanen HO, Haahtela T. Association between type of training and risk of asthma in elite athletes. Thorax. 1997;52(2):157-60.https://doi.org/10.1136/thx.52.2.157 | Excluded | Lack of relation between the title and the abstract of the article with the subject under study. |
|  | Henneberger PK. Work-exacerbated asthma. Current Opinion in Allergy and Clinical Immunology. 2007;7(2):146-51.https://doi.org/10.1097/ACI.0b013e328054c640 | Excluded | Lack of relation between the title and the abstract of the article with the subject under study. |
|  | Heraghty JL, Henderson AJ. Highlights in asthma 2005. Archives of Disease in Childhood. 2006;91(5):422-5.https://doi.org/10.1136/adc.2006.094094 | Excluded | Lack of relation between the title and the abstract of the article with the subject under study. |
|  | Herbert RD, Maher CG, Moseley AM, Sherrington C. Regular review - Effective physiotherapy. Bmj-British Medical Journal. 2001;323(7316):788-90.https://doi.org/10.1136/bmj.323.7316.788 | Excluded | Lack of relation between the title and the abstract of the article with the subject under study. |
|  | Herbison P, Robertson MC, McKenzie JE. Do alternative methods for analysing count data produce similar estimates? Implications for meta-analyses. Systematic Reviews. 2015;4(1).https://doi.org/10.1186/s13643-015-0144-x | Excluded | Lack of relation between the title and the abstract of the article with the subject under study. |
|  | Hess DR. Evidence-based respiratory care. Respiratory Care. 2021;66(7):1105-19.https://doi.org/10.4187/RESPCARE.08950 | Excluded | Lack of relation between the title and the abstract of the article with the subject under study. |
|  | Hieftje K, Edelman EJ, Camenga DR, Fiellin LE. Electronic media-based health interventions promoting behavior change in youth: A systematic review. JAMA Pediatrics. 2013;167(6):574-80.https://doi.org/10.1001/jamapediatrics.2013.1095 | Excluded | Lack of relation between the title and the abstract of the article with the subject under study. |
|  | Hiles S, Gibson P, McDonald V. Treatable traits predict health status and treatment response in airways disease. Respirology. 2019;24:61.https://doi.org/10.1111/resp.13491 | Excluded | Lack of relation between the title and the abstract of the article with the subject under study. |
|  | Hiles SA, Gibson PG, Agusti A, McDonald VM. Treatable Traits That Predict Health Status and Treatment Response in Airway Disease. The journal of allergy and clinical immunology In practice. 2021;9(3):1255-64.e2.https://doi.org/10.1016/j.jaip.2020.09.046 | Excluded | Lack of relation between the title and the abstract of the article with the subject under study. |
|  | Hill AR, Silverberg NB, Mayorga D, Baldwin HE. Medical hazards of the tear gas CS: A case of persistent, multisystem, hypersensitivity reaction and review of the literature. Medicine. 2000;79(4):234-40.https://doi.org/10.1097/00005792-200007000-00004 | Excluded | Lack of relation between the title and the abstract of the article with the subject under study. |
|  | Hill K, Gardiner PA, Cavalheri V, Jenkins SC, Healy GN. Physical activity and sedentary behaviour: Applying lessons to chronic obstructive pulmonary disease. Internal Medicine Journal. 2015;45(5):474-82.https://doi.org/10.1111/imj.12570 | Excluded | Lack of relation between the title and the abstract of the article with the subject under study. |
|  | Holland AE, Lewis A. Evidence-based management of symptoms in serious respiratory illness: what is in our toolbox? European Respiratory Review. 2024;33(174).https://doi.org/10.1183/16000617.0205-2024 | Excluded | Lack of relation between the title and the abstract of the article with the subject under study. |
|  | Holland R, Houten JK, Elsamragy S, Kim J, Leyvi G, Kinon MD. Intraoperative Thrombolysis of Massive Pulmonary Embolus During Spine Surgery: Case Report of Survival Complicated by Massive Bleeding and Review of the Literature. World Neurosurgery. 2021;146:59-63.https://doi.org/10.1016/j.wneu.2020.10.018 | Excluded | Lack of relation between the title and the abstract of the article with the subject under study. |
|  | Holmes RL, Fadden CT. Evaluation of the Patient with Chronic Cough. American Family Physician. 2004;69(9):2159-66+69 | Excluded | Lack of relation between the title and the abstract of the article with the subject under study. |
|  | Holthof K, Bridevaux PO, Frésard I. Underlying lung disease and exposure to terrestrial moderate and high altitude: personalised risk assessment. BMC Pulmonary Medicine. 2022;22(1).https://doi.org/10.1186/s12890-022-01979-z | Excluded | Lack of relation between the title and the abstract of the article with the subject under study. |
|  | Hon KL, Leung AKC, Wong AHC, Dudi A, Leung KKY. Respiratory Syncytial Virus is the Most Common Causative Agent of Viral Bronchiolitis in Young Children: An Updated Review. Current Pediatric Reviews. 2023;19(2):139-49.https://doi.org/10.2174/1573396318666220810161945 | Excluded | Lack of relation between the title and the abstract of the article with the subject under study. |
|  | Hon KLE, Fung CK, Leung AKC, Leung TNH, Ng DKK. Complementary and alternative medicine for childhood asthma: An overview of evidence and patents. Recent Patents on Inflammation and Allergy Drug Discovery. 2015;9(1):66-79.https://doi.org/10.2174/1872213X09666150302105225 | Excluded | Lack of relation between the title and the abstract of the article with the subject under study. |
|  | Hon KLE, Leung AKC. Medications and recent patents for status asthmaticus in children. Recent Patents on Inflammation and Allergy Drug Discovery. 2017;11(1):12-21.https://doi.org/10.2174/1872213X11666170130143524 | Excluded | Lack of relation between the title and the abstract of the article with the subject under study. |
|  | Honkoop P, Usmani O, Bonini M. The Current and Future Role of Technology in Respiratory Care. Pulmonary Therapy. 2022;8(2):167-79.https://doi.org/10.1007/s41030-022-00191-y | Excluded | Lack of relation between the title and the abstract of the article with the subject under study. |
|  | Hoppe UC, Böhm M, Dietz R, Hanrath P, Kroemer HK, Osterspey A, et al. Guidelines for the treatment of chronic heart failure. Zeitschrift fur Kardiologie. 2005;94(8):488-509.https://doi.org/10.1007/s00392-005-0268-4 | Excluded | Lack of relation between the title and the abstract of the article with the subject under study. |
|  | Horikoshi M, Beaumont RN, Day FR, Warrington NM, Kooijman MN, Fernandez-Tajes J, et al. Genome-wide associations for birth weight and correlations with adult disease. Nature. 2016;538(7624):248-52.https://doi.org/10.1038/nature19806 | Excluded | Lack of relation between the title and the abstract of the article with the subject under study. |
|  | Horikoshi M, Yaghootkar H, Mook-Kanamori DO, Sovio U, Taal HR, Hennig BJ, et al. New loci associated with birth weight identify genetic links between intrauterine growth and adult height and metabolism. Nature Genetics. 2013;45(1):76-82.https://doi.org/10.1038/ng.2477 | Excluded | Lack of relation between the title and the abstract of the article with the subject under study. |
|  | Hosford K, Firth C, Brauer M, Winters M. The effects of road pricing on transportation and health equity: a scoping review. Transport Reviews. 2021;41(6):766-87.https://doi.org/10.1080/01441647.2021.1898488 | Excluded | Lack of relation between the title and the abstract of the article with the subject under study. |
|  | Hosoki K, Chakraborty A, Sur S. Molecular mechanisms and epidemiology of COVID-19 from an allergist's perspective. Journal of Allergy and Clinical Immunology. 2020;146(2):285-99.https://doi.org/10.1016/j.jaci.2020.05.033 | Excluded | Lack of relation between the title and the abstract of the article with the subject under study. |
|  | Houle MC, Sjulin TJ, McInnis IC, Walter RJ, Morris MJ. Clinical Evaluation of Exertional Dyspnea in Adult Pectus Excavatum Patients. Current Respiratory Medicine Reviews. 2022;18(1):12-9.https://doi.org/10.2174/1573398X18666220128110107 | Excluded | Lack of relation between the title and the abstract of the article with the subject under study. |
|  | Hoy R. Work-related laryngeal syndromes. Current Opinion in Allergy and Clinical Immunology. 2012;12(2):95-101.https://doi.org/10.1097/ACI.0b013e328350fdaa | Excluded | Lack of relation between the title and the abstract of the article with the subject under study. |
|  | Hsu UH, Chiang BL. γδ T Cells and Allergic Diseases. Clinical Reviews in Allergy and Immunology. 2023;65(2):172-82.https://doi.org/10.1007/s12016-023-08966-0 | Excluded | Lack of relation between the title and the abstract of the article with the subject under study. |
|  | Hu ZW, Wang ZG, Zhang Y, Tian SR, Wu JM, Zhu GC, et al. Gastroesophageal reflux in chronic cough and cough syncope and the effect of antireflux treatment: Case report and literature review. Annals of Otology, Rhinology and Laryngology. 2014;123(10):719-25.https://doi.org/10.1177/0003489414534011 | Excluded | Lack of relation between the title and the abstract of the article with the subject under study. |
|  | Hua DT, Shah F, Perez-Corral C. A case of spontaneous pneumomediastinum in a patient with severe SARS-CoV-2 and a review of the literature. SAGE Open Medical Case Reports. 2021;9.https://doi.org/10.1177/2050313X211010021 | Excluded | Lack of relation between the title and the abstract of the article with the subject under study. |
|  | Huang E, Albrecht L, O’Hearn K, Nicolas N, Armstrong J, Weinberg M, et al. Reporting of social determinants of health in randomized controlled trials conducted in the pediatric intensive care unit. Frontiers in Pediatrics. 2024;12.https://doi.org/10.3389/fped.2024.1329648 | Excluded | Lack of relation between the title and the abstract of the article with the subject under study. |
|  | Huang J, Ding M, Zhu X, Chen P, Han S. Pulmonary epithelioid hemangioendothelioma complicated with chronic obstructive pulmonary disease :A case report and literature review. Journal of Jilin University Medicine Edition. 2021;47(1):196-202.https://doi.org/10.13481/j.1671-587x.20210127 | Excluded | Lack of relation between the title and the abstract of the article with the subject under study. |
|  | Huang P, Zhao Y, Wei H, Wu W, Guo Z, Ma S, et al. Causal Relationships Between Blood Lipid Levels and Chronic Obstructive Pulmonary Disease: A Mendelian Randomization Analysis. International Journal of COPD. 2025;20:83-93.https://doi.org/10.2147/COPD.S476833 | Excluded | Lack of relation between the title and the abstract of the article with the subject under study. |
|  | Huang QP, Xie ZF, Huang J. Assessment of the Association between Genetic Polymorphisms in the CHI3L1 Gene and Asthma Risk. International Archives of Allergy and Immunology. 2022;183(8):907-18.https://doi.org/10.1159/000522393 | Excluded | Lack of relation between the title and the abstract of the article with the subject under study. |
|  | Huang T, Wang T, Zheng Y, Ellervik C, Li X, Gao M, et al. Association of Birth Weight with Type 2 Diabetes and Glycemic Traits: A Mendelian Randomization Study. JAMA Network Open. 2019.https://doi.org/10.1001/jamanetworkopen.2019.10915 | Excluded | Lack of relation between the title and the abstract of the article with the subject under study. |
|  | Huckvale K, Car M, Morrison C, Car J. Apps for asthma self-management: A systematic assessment of content and tools. BMC Medicine. 2012;10.https://doi.org/10.1186/1741-7015-10-144 | Excluded | Lack of relation between the title and the abstract of the article with the subject under study. |
|  | Hudler A, Holguin F, Althoff M, Fuhlbrigge A, Sharma S. Pathophysiology and clinical evaluation of the patient with unexplained persistent dyspnea. Expert Review of Respiratory Medicine. 2022;16(5):511-8.https://doi.org/10.1080/17476348.2022.2030222 | Excluded | Lack of relation between the title and the abstract of the article with the subject under study. |
|  | Hudson-Colby JJ, Lewis A, Varkonyi-Sepp J, Ainsworth B, Freeman A, Day A, et al. Understanding the impact of breathing pattern disorders in difficult-to-treat asthma. Expert Review of Respiratory Medicine. 2024;18(10):777-88.https://doi.org/10.1080/17476348.2024.2404673 | Excluded | Lack of relation between the title and the abstract of the article with the subject under study. |
|  | Hughes AR, Reilly JJ. Disease management programs targeting obesity in children: Setting the scene for wellness in the future. Disease Management and Health Outcomes. 2008;16(4):255-66.https://doi.org/10.2165/00115677-200816040-00006 | Excluded | Lack of relation between the title and the abstract of the article with the subject under study. |
|  | Hulkower S, Pagan B, Watts J, Ketterman E. Do preparticipation clinical exams reduce morbidity and mortality for athletes? Journal of Family Practice. 2005;54(7):628-9+32 | Excluded | Lack of relation between the title and the abstract of the article with the subject under study. |
|  | Hull JH, Jackson AR, Ranson C, Brown F, Wootten M, Loosemore M. The benefits of a systematic assessment of respiratory health in illnesssusceptible athletes. European Respiratory Journal. 2021;57(6).https://doi.org/10.1183/13993003.03722-2020 | Excluded | Lack of relation between the title and the abstract of the article with the subject under study. |
|  | Human A, Mostert-Wentzel K. Best current evidence on chest physiotherapy in non-ventilated paediatric patients (0-24 months) with bronchiolitis: A systematic review. Physiotherapy (United Kingdom). 2011;97:eS514-eS5.https://doi.org/10.1016/j.physio.2011.04.002 | Excluded | Lack of relation between the title and the abstract of the article with the subject under study. |
|  | Humbert M. Update on the European Respiratory Review. European Respiratory Journal. 2010;36(5):993-4.https://doi.org/10.1183/09031936.00134210 | Excluded | Lack of relation between the title and the abstract of the article with the subject under study. |
|  | Humphreys J, Martin H, Roberts B, Ferretti C. Strengthening an academic nursing center through partnership. Nursing Outlook. 2004;52(4):197-202.https://doi.org/10.1016/S0029-6554(04)00067-3 | Excluded | Lack of relation between the title and the abstract of the article with the subject under study. |
|  | Hunt K, Ernst E. The evidence-base for complementary medicine in children: A critical overview of systematic reviews. Archives of Disease in Childhood. 2011;96(8):769-76.https://doi.org/10.1136/adc.2009.179036 | Excluded | Lack of relation between the title and the abstract of the article with the subject under study. |
|  | Hussein MS, Ismail NE. A review of health outcome instruments for asthmatic children & their caregivers. International Journal of Pharmacy and Pharmaceutical Sciences. 2015;7(8):6-20 | Excluded | Lack of relation between the title and the abstract of the article with the subject under study. |
|  | Hutchinson JM, Raffoul A, Pepetone A, Andrade L, Williams TE, McNaughton SA, et al. Advances in methods for characterizing dietary patterns: A scoping review. 2024.10.1101/2024.06.20.24309251 | Excluded | Lack of relation between the title and the abstract of the article with the subject under study. |
|  | Hütter BO, Würtemberger G. Functional capacity (dyspnoea) and quality of life in patients with chronic obstructive pulmonary disease (COPD): Assessment and methodological aspects. Pneumologie. 1999;53(3):133-42 | Excluded | Lack of relation between the title and the abstract of the article with the subject under study. |
|  | Idrees M, Fitzgerald JM. Vocal cord dysfunction in bronchial asthma. A review article. Journal of Asthma. 2015;52(4):327-35.https://doi.org/10.3109/02770903.2014.982288 | Excluded | Lack of relation between the title and the abstract of the article with the subject under study. |
|  | Iessa N, Berard A. Update on Prepregnancy Maternal Obesity: Birth Defects and Childhood Outcomes. Journal of pediatric genetics. 2015;4(2):71-83.https://doi.org/10.1055/s-0035-1556739 | Excluded | Lack of relation between the title and the abstract of the article with the subject under study. |
|  | Illidi CR, Romer LM, Johnson MA, Williams NC, Rossiter HB, Casaburi R, et al. Distinguishing science from pseudoscience in commercial respiratory interventions: an evidence-based guide for health and exercise professionals. European Journal of Applied Physiology. 2023;123(8):1599-625.https://doi.org/10.1007/s00421-023-05166-8 | Excluded | Lack of relation between the title and the abstract of the article with the subject under study. |
|  | Inchauspe RM, Maróstica PJC, Barreto SSM, da Silva MM, Rabaioli CT. The effectiveness of the use of video games and software-based programs for asthma education and self-management for children and teenagers. Fisioterapia em Movimento. 2021;34:1-7.https://doi.org/10.1590/fm.2021.34202 | Excluded | Lack of relation between the title and the abstract of the article with the subject under study. |
|  | Inchingolo R, Ielo S, Barone R, Whalen MB, Carriera L, Smargiassi A, et al. Ultrasound and Intrapleural Enzymatic Therapy for Complicated Pleural Effusion: A Case Series with a Literature Review. Journal of Clinical Medicine. 2024;13(15).https://doi.org/10.3390/jcm13154346 | Excluded | Lack of relation between the title and the abstract of the article with the subject under study. |
|  | Ionescu MD, Popescu NA, Stănescu D, Enculescu A, Bălgrădean M, Căpitănescu GM, et al. The Challenging Diagnosis of Interstitial Lung Disease in Children—One Case Report and Literature Review. Journal of Clinical Medicine. 2022;11(22).https://doi.org/10.3390/jcm11226736 | Excluded | Lack of relation between the title and the abstract of the article with the subject under study. |
|  | Islam MZ, Johnston J, Sly PD. Green space and early childhood development: a systematic review. Reviews on environmental health. 2020;35(2):189-200.https://doi.org/10.1515/reveh-2019-0046 | Excluded | Lack of relation between the title and the abstract of the article with the subject under study. |
|  | Ismail AB, Ergören MÇ. Mediterranean exposotype: Genomic architecture and plant-based dietary metabolites. Clinical Nutrition ESPEN. 2023;55:1-9.https://doi.org/10.1016/j.clnesp.2023.02.017 | Excluded | Lack of relation between the title and the abstract of the article with the subject under study. |
|  | Ismail K, Stahl D, Bayley A, Twist K, Stewart K, Ridge K, et al. Enhanced motivational interviewing for reducing weight and increasing physical activity in adults with high cardiovascular risk: The MOVE IT three-arm RCT. Health Technology Assessment. 2019;23(69):1-144.https://doi.org/10.3310/hta23690 | Excluded | Lack of relation between the title and the abstract of the article with the subject under study. |
|  | Iyer VN, Lim KG. Bronchial Thermoplasty: Reappraising the Evidence (or Lack Thereof ). Chest. 2014;146(1):17-21.https://doi.org/10.1378/chest.14-0536 | Excluded | Lack of relation between the title and the abstract of the article with the subject under study. |
|  | Jacquemin B, Schikowski T, Carsin A, Hansell A, Krämer U, Sunyer J, et al. The role of air pollution in adult-onset asthma: A review of the current evidence. Seminars in Respiratory and Critical Care Medicine. 2012;33(6):606-19.https://doi.org/10.1055/s-0032-1325191 | Excluded | Lack of relation between the title and the abstract of the article with the subject under study. |
|  | Jafri S, Janzen J, Kim R, Abrams EM, Gruber J, Protudjer JLP. Burden of Allergic Disease in Racial and Ethnic Structurally Oppressed Communities Within Canada and the United States: A Scoping Review. Journal of Allergy and Clinical Immunology: In Practice. 2022;10(11):2995-3001.https://doi.org/10.1016/j.jaip.2022.08.018 | Excluded | Lack of relation between the title and the abstract of the article with the subject under study. |
|  | Jain A, Lolak S. Psychiatric aspects of chronic lung disease. Current Psychiatry Reports. 2009;11(3):219-25.https://doi.org/10.1007/s11920-009-0034-9 | Excluded | Lack of relation between the title and the abstract of the article with the subject under study. |
|  | Jain K, Wainwright CE, Smyth AR. Bronchoscopy-guided antimicrobial therapy for cystic fibrosis. Cochrane Database of Systematic Reviews. 2024;2024(5).https://doi.org/10.1002/14651858.CD009530.pub5 | Excluded | Lack of relation between the title and the abstract of the article with the subject under study. |
|  | Jaleel G, Khan RA, Khan A, Malhotra D. Effect of electromagnetic field therapy in chronic respiratory disease: systematic review. Physiotherapy Quarterly. 2024;32(2):19-24.https://doi.org/10.5114/pq/166496 | Excluded | Lack of relation between the title and the abstract of the article with the subject under study. |
|  | Janjua S, Carter D, Threapleton CJD, Prigmore S, T DR. Telehealth interventions: remote monitoring and consultations for people with chronic obstructive pulmonary disease (COPD) (Cochrane review) [with consumer summary]. Cochrane Database of Systematic Reviews 2021;Issue 7. 2021 | Excluded | Lack of relation between the title and the abstract of the article with the subject under study. |
|  | Janjua S, Powell P, Atkinson R, Stovold E, Fortescue R. Individual‐level interventions to reduce personal exposure to outdoor air pollution and their effects on people with long‐term respiratory conditions. Cochrane Database of Systematic Reviews. 2021(8).https://doi.org/10.1002/14651858.CD013441.pub2 | Excluded | Lack of relation between the title and the abstract of the article with the subject under study. |
|  | Janson P, Willeke K, Zaibert L, Budnick A, Berghöfer A, Kittel-Schneider S, et al. Mortality, Morbidity and Health-Related Outcomes in Informal Caregivers Compared to Non-Caregivers: A Systematic Review. International Journal of Environmental Research and Public Health. 2022;19(10).https://doi.org/10.3390/ijerph19105864 | Excluded | Lack of relation between the title and the abstract of the article with the subject under study. |
|  | Jayawardena R, Ranasinghe P, Ranawaka H, Gamage N, Dissanayake D, Misra A. Exploring the therapeutic benefits of Pranayama (yogic breathing): A systematic review. International Journal of Yoga. 2020;13(2):99-110.https://doi.org/10.4103/ijoy.IJOY_37_19 | Excluded | Lack of relation between the title and the abstract of the article with the subject under study. |
|  | Jefferson T, Jones MA, Doshi P, Del Mar CB, Hama R, Thompson MJ, et al. Neuraminidase inhibitors for preventing and treating influenza in adults and children. The Cochrane database of systematic reviews. 2014;2014(4):Cd008965.https://doi.org/10.1002/14651858.CD008965.pub4 | Excluded | Lack of relation between the title and the abstract of the article with the subject under study. |
|  | Jefferson T, Jones MA, Doshi P, Del Mar CB, Hama R, Thompson MJ, et al. Neuraminidase inhibitors for preventing and treating influenza in adults and children. Cochrane Database of Systematic Reviews. 2014(4).https://doi.org/10.1002/14651858.CD008965.pub4 | Excluded | Lack of relation between the title and the abstract of the article with the subject under study. |
|  | Jenkins AR, Gaynor-Sodeifi K, Lewthwaite H, Triandafilou J, Belo LF, de Oliveira MF, et al. Efficacy of interventions to alter measures of fat-free mass in people with COPD: a systematic review and meta-analysis. ERJ Open Research. 2023;9(4).https://doi.org/10.1183/23120541.00102-2023 | Excluded | Lack of relation between the title and the abstract of the article with the subject under study. |
|  | Jenkins CR, Chapman KR, Donohue JF, Roche N, Tsiligianni I, Han MK. Improving the Management of COPD in Women. Chest. 2017;151(3):686-96.https://doi.org/10.1016/j.chest.2016.10.031 | Excluded | Lack of relation between the title and the abstract of the article with the subject under study. |
|  | Jensen ME, Barrett HL, Peek MJ, Gibson PG, Murphy VE. Maternal asthma and gestational diabetes mellitus: Exploration of potential associations. Obstetric Medicine. 2021;14(1):12-8.https://doi.org/10.1177/1753495X20926799 | Excluded | Lack of relation between the title and the abstract of the article with the subject under study. |
|  | Jerrett M, Connolly R, Garcia-Gonzales DA, Bekker C, Nguyen JT, Su J, et al. Climate change and public health in California: A structured review of exposures, vulnerable populations, and adaptation measures. Proceedings of the National Academy of Sciences of the United States of America. 2024;121(32).https://doi.org/10.1073/pnas.2310081121 | Excluded | Lack of relation between the title and the abstract of the article with the subject under study. |
|  | Jiao Y, Gong C, Wang S, Duan Y, Zhang Y. The Influence of Air Pollution on Pulmonary Disease Incidence Analyzed Based on Grey Correlation Analysis. Contrast Media and Molecular Imaging. 2022;2022.https://doi.org/10.1155/2022/4764720 | Excluded | Lack of relation between the title and the abstract of the article with the subject under study. |
|  | Jimenez MP, Deville NV, Elliott EG, Schiff JE, Wilt GE, Hart JE, et al. Associations between nature exposure and health: A review of the evidence. International Journal of Environmental Research and Public Health. 2021;18(9).https://doi.org/10.3390/ijerph18094790 | Excluded | Lack of relation between the title and the abstract of the article with the subject under study. |
|  | Johannessen A, Xu S, Abbah AP, Janson C. Greenness exposure: beneficial but multidimensional. A review. European Respiratory Journal. 2023;62:PA1602.https://doi.org/10.1183/13993003.congress-2023.PA1602 | Excluded | Lack of relation between the title and the abstract of the article with the subject under study. |
|  | Johansson MKV, Johanson G, Oberg M. Evaluation of the experimental basis for assessment factors to protect individuals with asthma from health effects during short-term exposure to airborne chemicals. Critical Reviews in Toxicology. 2016;46(3):241-60.https://doi.org/10.3109/10408444.2015.1092498 | Excluded | Lack of relation between the title and the abstract of the article with the subject under study. |
|  | Jolfaei AG, Karim H, Salehian R, Kashaninasab F. Sleep disturbance in hospitalized medical patients: A review article. Archivos Venezolanos de Farmacologia y Terapeutica. 2021;40(8):828-38.https://doi.org/10.5281/zenodo.5791353 | Excluded | Lack of relation between the title and the abstract of the article with the subject under study. |
|  | Jolliffe DA, Camargo CA, Sluyter JD, Aglipay M, Aloia JF, Bergman P, et al. Vitamin D supplementation to prevent acute respiratory infections: systematic review and meta-analysis of stratified aggregate data. 2024.10.1101/2024.09.18.24313866 | Excluded | Lack of relation between the title and the abstract of the article with the subject under study. |
|  | Jolliffe DA, Camargo CA, Sluyter JD, Aglipay M, Aloia JF, Ganmaa D, et al. Vitamin D supplementation to prevent acute respiratory infections: a systematic review and meta-analysis of aggregate data from randomised controlled trials. The Lancet Diabetes and Endocrinology. 2021;9(5):276-92.https://doi.org/10.1016/S2213-8587(21)00051-6 | Excluded | Lack of relation between the title and the abstract of the article with the subject under study. |
|  | Jones JRA, Karahalios A, Puthucheary ZA, Berry MJ, Files DC, Griffith DM, et al. Responsiveness of Critically Ill Adults with Multimorbidity to Rehabilitation Interventions: A Patient-Level Meta-Analysis Using Individual Pooled Data from Four Randomized Trials. Critical Care Medicine. 2023;51(10):1373-85.https://doi.org/10.1097/CCM.0000000000005936 | Excluded | Lack of relation between the title and the abstract of the article with the subject under study. |
|  | Jones M, Harvey A, Marston L, O'Connell NE. Breathing exercises for dysfunctional breathing/hyperventilation syndrome in adults. Cochrane Database of Systematic Reviews. 2013(5).https://doi.org/10.1002/14651858.CD009041.pub2 | Excluded | Lack of relation between the title and the abstract of the article with the subject under study. |
|  | Jones MH, Stein RT. Long-term respiratory outcomes of premature infants. Pediatric Pulmonology. 2019;54:S51-S2.https://doi.org/10.1002/ppul.24371 | Excluded | Lack of relation between the title and the abstract of the article with the subject under study. |
|  | Jones TM, Alderson D, Sheard JDH, Switf AC. Tracheal paraganglioma: A diagnostic dilemma culminating in a complex airway management problem. Journal of Laryngology and Otology. 2001;115(9):747-9.https://doi.org/10.1258/0022215011908838 | Excluded | Lack of relation between the title and the abstract of the article with the subject under study. |
|  | Joo HH, Huang EY, Schoo D, Ward B, Chen JX. Association Between Hearing Difficulty and Mobility in Adults of All Ages: National Health Interview Survey. Otolaryngology - Head and Neck Surgery (United States). 2024;170(4):1059-65.https://doi.org/10.1002/ohn.593 | Excluded | Lack of relation between the title and the abstract of the article with the subject under study. |
|  | Jubber I, Ong S, Bukavina L, Black PC, Compérat E, Kamat AM, et al. Epidemiology of Bladder Cancer in 2023: A Systematic Review of Risk Factors. European Urology. 2023;84(2):176-90.https://doi.org/10.1016/j.eururo.2023.03.029 | Excluded | Lack of relation between the title and the abstract of the article with the subject under study. |
|  | Jude JA, Panettieri RA. Bronchomotor tone imbalance evokes airway hyperresponsiveness. Expert Review of Respiratory Medicine. 2024;18(11):835-41.https://doi.org/10.1080/17476348.2024.2419543 | Excluded | Lack of relation between the title and the abstract of the article with the subject under study. |
|  | Juel CT, Ali Z, Nilas L, Ulrik CS. Asthma and obesity: does weight loss improve asthma control? a systematic review. J Asthma Allergy. 2012;5:21-6.https://doi.org/10.2147/jaa.S32232 | Excluded | Lack of relation between the title and the abstract of the article with the subject under study. |
|  | Juniper EF. The value of quality of life in asthma. European Respiratory Review. 1997;7(49):333-7 | Excluded | Lack of relation between the title and the abstract of the article with the subject under study. |
|  | Jurov I, Demšar J. Factors affecting maximal oxygen uptake in prepubertal children: a systematic review and meta-analysis. BMC Pediatrics. 2024;24(1).https://doi.org/10.1186/s12887-024-05013-5 | Excluded | Lack of relation between the title and the abstract of the article with the subject under study. |
|  | Justicia-Grande AJ, Martinón-Torres F. The ReSVinet Score for Bronchiolitis: A Scale for All Seasons. American Journal of Perinatology. 2019;36:S48-S53.https://doi.org/10.1055/s-0039-1691800 | Excluded | Lack of relation between the title and the abstract of the article with the subject under study. |
|  | Kabisch N, van den Bosch M, Lafortezza R. The health benefits of nature-based solutions to urbanization challenges for children and the elderly - A systematic review. Environmental Research. 2017;159:362-73.https://doi.org/10.1016/j.envres.2017.08.004 | Excluded | Lack of relation between the title and the abstract of the article with the subject under study. |
|  | Kabra SK. Management and prevention of chronic suppurative lung diseases and bronchiolitis obliterans. Pediatric Pulmonology. 2018;53:S43-S5.https://doi.org/10.1002/ppul.24031 | Excluded | Lack of relation between the title and the abstract of the article with the subject under study. |
|  | Kabra SK, Lodha R, editors. Long-term management of asthma. Indian Journal of Pediatrics; 2003. | Excluded | Lack of relation between the title and the abstract of the article with the subject under study. |
|  | Kahl CG, Deas C. Exercise-induced anaphylaxis in an air force aviator taking a HMG-CoA reductase inhibitor: A case report and review of the presentation, diagnoses, and treatment. Military Medicine. 2017;182(5):e1816-e9.https://doi.org/10.7205/MILMED-D-16-00247 | Excluded | Lack of relation between the title and the abstract of the article with the subject under study. |
|  | Kahn N, Mekov E, Fregonese L, Andrianopoulos V, Franssen FME, Grgic A, et al. European respiratory society international congress 2017: Highlights from the clinical assembly. ERJ Open Research. 2018;4(1).https://doi.org/10.1183/23120541.00134-2017 | Excluded | Lack of relation between the title and the abstract of the article with the subject under study. |
|  | Kajantie E, Strang-Karlsson S, Evensen KAI, Haaramo P. Adult outcomes of being born late preterm or early term – What do we know? Seminars in Fetal and Neonatal Medicine. 2019;24(1):66-83.https://doi.org/10.1016/j.siny.2018.11.001 | Excluded | Lack of relation between the title and the abstract of the article with the subject under study. |
|  | Kalhoff H. Mild dehydration: A risk factor of broncho-pulmonary disorders? European Journal of Clinical Nutrition. 2003;57(SUPPL.2):S81-S7.https://doi.org/10.1038/sj.ejcn.1601906 | Excluded | Lack of relation between the title and the abstract of the article with the subject under study. |
|  | Kamal AH, Maguire JM, Wheeler JL, Currow DC, Abernethy AP. Dyspnea review for the palliative care professional: Treatment goals and therapeutic options. Journal of Palliative Medicine. 2012;15(1):106-14.https://doi.org/10.1089/jpm.2011.0110 | Excluded | Lack of relation between the title and the abstract of the article with the subject under study. |
|  | Kamalanathan E, Sharma S. Inhaled Corticosteroids Versus Placebo for Stable Chronic Obstructive Pulmonary Disease. Clinical and Experimental Allergy. 2024;54(7):455-8.https://doi.org/10.1111/cea.14521 | Excluded | Lack of relation between the title and the abstract of the article with the subject under study. |
|  | Kamani P, Sinha R, Vachhani B, Myint EI, Iyer I, Kukkar JR, et al. CASTING LIGHT ON THE UNFORESEEN: NEBULIZED IPRATROPIUM'S RARE TWIST--A CASE REPORT OF URINARY RETENTION IN A PATIENT WITHOUT BENIGN PROSTATIC HYPERPLASIA. 2024. p. A2059-A60.10.1016/j.chest.2024.06.1275 | Excluded | Lack of relation between the title and the abstract of the article with the subject under study. |
|  | Kamath A, Aravind Kudva R, Mallya L, Shenoy R. Update on Medical emergency in dental operatory - A Review. NeuroQuantology. 2023;21(1):361-7.https://doi.org/10.48047/nq.2023.21.01.NQ20027 | Excluded | Lack of relation between the title and the abstract of the article with the subject under study. |
|  | Kaminskyj A, Frazier M, Johnstone K, Gleberzon BJ. Chiropractic care for patients with asthma: a systematic review of the literature. Journal of the Canadian Chiropractic Association 2010 Mar;54(1):24-32. 2010 | Excluded | Lack of relation between the title and the abstract of the article with the subject under study. |
|  | Kamp Mvd, Hengeveld VS, Brusse-Keizer MGJ, Thio BJ, Tabak M. eHealth Technologies for Monitoring Pediatric Asthma at Home: Scoping Review. Journal of Medical Internet Research. 2023;25.https://doi.org/10.2196/45896 | Excluded | Lack of relation between the title and the abstract of the article with the subject under study. |
|  | Kanda M, Kamekura R, Sugawara M, Nagahata K, Suzuki C, Takano K, et al. IgG4-related disease administered dupilumab: Case series and review of the literature. RMD Open. 2023;9(1).https://doi.org/10.1136/rmdopen-2023-003026 | Excluded | Lack of relation between the title and the abstract of the article with the subject under study. |
|  | Kanoje R, Pargaonkar A. Home remedies for the elderly in the direction of protection against COVID-19: An ayurveda perspective. International Journal of Research in Pharmaceutical Sciences. 2020;11(Special Issue 1):1167-70.https://doi.org/10.26452/ijrps.v11iSPL1.3576 | Excluded | Lack of relation between the title and the abstract of the article with the subject under study. |
|  | Karakatsani A, Analitis A, Perifanou D, Ayres JG, Harrison RM, Kotronarou A, et al. Particulate matter air pollution and respiratory symptoms in individuals having either asthma or chronic obstructive pulmonary disease: a European multicentre panel study. Environmental health : a global access science source. 2012;11:75.https://doi.org/10.1186/1476-069x-11-75 | Excluded | Lack of relation between the title and the abstract of the article with the subject under study. |
|  | Karakousis ND, Kotsiou OS, Gourgoulianis KI. Bronchial Asthma and Sarcopenia: An Upcoming Potential Interaction. Journal of Personalized Medicine. 2022;12(10).https://doi.org/10.3390/jpm12101556 | Excluded | Lack of relation between the title and the abstract of the article with the subject under study. |
|  | Karim HMR, Esquinas AM, Ziatabar S, Insalaco G, Skoczyński S, Šarc I, et al. Continuous positive airway pressure (CPAP) in nonapneic asthma: A clinical review of current evidence. Turkish Thoracic Journal. 2020;21(4):274-9.https://doi.org/10.5152/TurkThoracJ.2019.19049 | Excluded | Lack of relation between the title and the abstract of the article with the subject under study. |
|  | Karkouli G, Douros K, Moriki D, Moutsatsou P, Giannopoulou I, Maratou E, et al. Dysfunctional Breathing in Children: A Literature Review. Children. 2024;11(5).https://doi.org/10.3390/children11050556 | Excluded | Lack of relation between the title and the abstract of the article with the subject under study. |
|  | Kasparian AM, Badawy SM. Utility of Fitbit devices among children and adolescents with chronic health conditions: a scoping review. mHealth. 2022;8.https://doi.org/10.21037/mhealth-21-28 | Excluded | Lack of relation between the title and the abstract of the article with the subject under study. |
|  | Katelaris CH, Linneberg A, Magnan A, Thomas WR, Wardlaw AJ, Wark P. Developments in the field of allergy in 2010 through the eyes of Clinical and Experimental Allergy. Clinical and Experimental Allergy. 2011;41(12):1690-710.https://doi.org/10.1111/j.1365-2222.2011.03892.x | Excluded | Lack of relation between the title and the abstract of the article with the subject under study. |
|  | Katja B, Thomas O, Arndt B. Effects of yoga interventions on fatigue: A meta-analysis. European Journal of Integrative Medicine. 2012;4:18.https://doi.org/10.1016/j.eujim.2012.07.515 | Excluded | Lack of relation between the title and the abstract of the article with the subject under study. |
|  | Keenan SP, Mehta S. Noninvasive ventilation for patients presenting with acute respiratory failure: the randomized controlled trials. Respiratory Care 2009 Jan;54(1):116-126. 2009 | Excluded | Lack of relation between the title and the abstract of the article with the subject under study. |
|  | Kelly C, Grundy S, Lynes D, Evans DJ, Gudur S, Milan SJ, et al. Self-management for bronchiectasis. The Cochrane database of systematic reviews. 2018;2(2):Cd012528.https://doi.org/10.1002/14651858.CD012528.pub2 | Excluded | Lack of relation between the title and the abstract of the article with the subject under study. |
|  | Kelly MM, Griffith PB. Umbrella Review of School Age Health Outcomes of Preterm Birth Survivors. Journal of Pediatric Health Care. 2020;34(5):e59-e76.https://doi.org/10.1016/j.pedhc.2020.05.007 | Excluded | Lack of relation between the title and the abstract of the article with the subject under study. |
|  | Kelly W, Massoumi A, Lazarus A. Asthma in pregnancy: Physiology, diagnosis, and management. Postgraduate Medicine. 2015;127(4):349-58.https://doi.org/10.1080/00325481.2015.1016386 | Excluded | Lack of relation between the title and the abstract of the article with the subject under study. |
|  | Kemble H, Foster M, Blamires J, Mowat R. Children and young people's self-reported experiences of asthma and self-management nursing strategies: An integrative review. Journal of Pediatric Nursing. 2024;77:212-35.https://doi.org/10.1016/j.pedn.2024.03.029 | Excluded | Lack of relation between the title and the abstract of the article with the subject under study. |
|  | Kemény KK, Ducza E. Physiological Cooperation between Aquaporin 5 and TRPV4. International journal of molecular sciences. 2022;23(19).https://doi.org/10.3390/ijms231911634 | Excluded | Lack of relation between the title and the abstract of the article with the subject under study. |
|  | Kemp J, Chesi A, Kreiner-Møller E, Ahluwalia T, Mook D, Liu Y, et al. GWAS meta-analysis for total body BMD unveils 14 new BMD loci and variants exerting age-specific effects. Journal of Bone and Mineral Research. 2015;30.https://doi.org/10.1002/jbmr.2763 | Excluded | Lack of relation between the title and the abstract of the article with the subject under study. |
|  | Kenn K, Hess MM. Vocal cord dysfunction: An important differential diagnosis of bronchial asthma. Deutsches Arzteblatt. 2008;105(41):699-704.https://doi.org/10.3238/arztebl.2008.0699 | Excluded | Lack of relation between the title and the abstract of the article with the subject under study. |
|  | Kennedy TM, Malia L, Dessie A, Kessler DO, Ng L, Chiang EL, et al. Lung point-of-care ultrasound in pediatric COVID-19: A case series. Pediatric Emergency Care. 2020;36(11):544-8.https://doi.org/10.1097/PEC.0000000000002254 | Excluded | Lack of relation between the title and the abstract of the article with the subject under study. |
|  | Kerr CP. Eight underused prescriptions. American Family Physician. 1994;50(7):1497-504 | Excluded | Lack of relation between the title and the abstract of the article with the subject under study. |
|  | Kesireddy N, Khokher W, Mudiyansselage P, Vukanti S. Efficacy of Exercise Program on Asthma Control Questionnaire: A Systematic Review and Meta-Analysis. American Journal of Respiratory and Critical Care Medicine. 2021;203(9).https://doi.org/10.1164/ajrccm-conference.2021.TP102 | Excluded | Lack of relation between the title and the abstract of the article with the subject under study. |
|  | Kew KM, Carr R, Donovan T, Gordon M. Asthma education for school staff. Cochrane Database of Systematic Reviews. 2017;2017(4).https://doi.org/10.1002/14651858.CD012255.pub2 | Excluded | Lack of relation between the title and the abstract of the article with the subject under study. |
|  | Khaddour K, Shayuk M, Ludhwani D, Gowda S, Ward WL. Pregnancy unmasking symptoms of undiagnosed lymphangioleiomyomatosis: Case report and review of literature. Respiratory Medicine Case Reports. 2019;26:63-7.https://doi.org/10.1016/j.rmcr.2018.11.010 | Excluded | Lack of relation between the title and the abstract of the article with the subject under study. |
|  | Khair AM, Kabrt J, Falchek S. Drug-Resistant Epilepsy in Children with Juvenile Huntington’s Disease: A Challenging Case and Brief Review. Qatar Medical Journal. 2020;2020(1).https://doi.org/10.5339/QMJ.2020.18 | Excluded | Lack of relation between the title and the abstract of the article with the subject under study. |
|  | Khandelwal V, Sharma T, Choudhary P, Gupta S, Sohal JS, Jain M, et al. MicroRNAs: An Important Signature Molecule to Improve Health and Welfare in Livestock: A Review. Indian Journal of Animal Research. 2024;58(2):179-89.https://doi.org/10.18805/IJAR.B-5231 | Excluded | Lack of relation between the title and the abstract of the article with the subject under study. |
|  | Khatib I, Young PM. Technegas, A Universal Technique for Lung Imaging in Nuclear Medicine: Technology, Physicochemical Properties, and Clinical Applications. Pharmaceutics. 2023;15(4).https://doi.org/10.3390/pharmaceutics15041108 | Excluded | Lack of relation between the title and the abstract of the article with the subject under study. |
|  | Khoiry QA, Alfian SD, van Boven JFM, Abdulah R. Self-reported medication adherence instruments and their applicability in low-middle income countries: a scoping review. Frontiers in public health. 2023;11:1104510.https://doi.org/10.3389/fpubh.2023.1104510 | Excluded | Lack of relation between the title and the abstract of the article with the subject under study. |
|  | Khoiry QAA, Alfian SDD, van Boven JFM, Abdulah R. Self-reported medication adherence instruments and their applicability in low-middle income countries: a scoping review. Frontiers in Public Health. 2023;11.https://doi.org/10.3389/fpubh.2023.1104510 | Excluded | Lack of relation between the title and the abstract of the article with the subject under study. |
|  | Khoo JK, Venning V, Wong C, Jayaram L. Bronchiectasis in the last five years: New developments. Journal of Clinical Medicine. 2016;5(12).https://doi.org/10.3390/jcm5120115 | Excluded | Lack of relation between the title and the abstract of the article with the subject under study. |
|  | Khrapov KN, Kovalev MG, Sedov SS. Preparation for anesthesia of patients with concomitant lung pathology and a high risk of developing postoperative pulmonary complications. Messenger of Anesthesiology and Resuscitation. 2020;17(2):20-8.https://doi.org/10.21292/2078-5658-2020-17-2-20-28 | Excluded | Lack of relation between the title and the abstract of the article with the subject under study. |
|  | Khurana S, Jarjour NN. Systematic Approach to Asthma of Varying Severity. Clinics in Chest Medicine. 2019;40(1):59-70.https://doi.org/10.1016/j.ccm.2018.10.004 | Excluded | Lack of relation between the title and the abstract of the article with the subject under study. |
|  | Kiani S, Abasi S, Yazdani A. Evaluation of m‐Health‐rehabilitation for respiratory disorders: A systematic review. Health Science Reports. 2022;5(3).https://doi.org/10.1002/hsr2.575 | Excluded | Lack of relation between the title and the abstract of the article with the subject under study. |
|  | Kilpeläinen TO, Carli JFM, Skowronski AA, Sun Q, Kriebel J, Feitosa MF, et al. Genome-wide meta-analysis uncovers novel loci influencing circulating leptin levels. Nature Communications. 2016;7.https://doi.org/10.1038/ncomms10494 | Excluded | Lack of relation between the title and the abstract of the article with the subject under study. |
|  | Kim A, Silverberg JI. A systematic review of vigorous physical activity in eczema. British Journal of Dermatology. 2016;174(3):660-2.https://doi.org/10.1111/bjd.14179 | Excluded | Lack of relation between the title and the abstract of the article with the subject under study. |
|  | Kim DK, Park YB, Oh YM, Jung KS, Yoo JH, Yoo KH, et al. Korean Asthma Guideline 2014: Summary of Major Updates to the Korean Asthma Guideline 2014. Tuberc Respir Dis (Seoul). 2016;79(3):111-20.https://doi.org/10.4046/trd.2016.79.3.111 | Excluded | Lack of relation between the title and the abstract of the article with the subject under study. |
|  | Kim JA, Song SY, Jeong W, Jun JK. Non-cancer health risks in firefighters: a systematic review. Epidemiology and Health. 2022;44.https://doi.org/10.4178/epih.e2022109 | Excluded | Lack of relation between the title and the abstract of the article with the subject under study. |
|  | Kim JH, Kim JY, Lee J, Jeong GH, Lee E, Lee S, et al. Environmental risk factors, protective factors, and peripheral biomarkers for ADHD: an umbrella review. The Lancet Psychiatry. 2020;7(11):955-70.https://doi.org/10.1016/S2215-0366(20)30312-6 | Excluded | Lack of relation between the title and the abstract of the article with the subject under study. |
|  | King KM, Humen DP, Teo KK. Cardiac rehabilitation: The forgotten intervention. Canadian Journal of Cardiology. 1999;15(9):979-85 | Excluded | Lack of relation between the title and the abstract of the article with the subject under study. |
|  | King NJ. The behavioral management of asthma and asthma-related problems in children: A critical review of the literature. Journal of Behavioral Medicine. 1980;3(2):169-89.https://doi.org/10.1007/BF00844989 | Excluded | Lack of relation between the title and the abstract of the article with the subject under study. |
|  | Kippelen P, Friemel F, Godard P. Asthma in athletes. Revue des Maladies Respiratoires. 2003;20(3):385-97 | Excluded | Lack of relation between the title and the abstract of the article with the subject under study. |
|  | Kiyohara M, Shirai T, Nishiyama S, Sato H, Fujii H, Ishii T, et al. Hypertrophic Pachymeningitis Development in Eosinophilic Granulomatosis with Polyangiitis at Relapse of Disease: A Case-Based Review. Tohoku Journal of Experimental Medicine. 2022;256(3):241-7.https://doi.org/10.1620/tjem.256.241 | Excluded | Lack of relation between the title and the abstract of the article with the subject under study. |
|  | Klain A, Dinardo G, Salvatori A, Indolfi C, Contieri M, Brindisi G, et al. An Overview on the Primary Factors That Contribute to Non-Allergic Asthma in Children. Journal of Clinical Medicine. 2022;11(21).https://doi.org/10.3390/jcm11216567 | Excluded | Lack of relation between the title and the abstract of the article with the subject under study. |
|  | Klain A, Senatore AA, Licari A, Galletta F, Bettini I, Tomei L, et al. The Prevention of House Dust Mite Allergies in Pediatric Asthma. Children. 2024;11(4).https://doi.org/10.3390/children11040469 | Excluded | Lack of relation between the title and the abstract of the article with the subject under study. |
|  | Kleniewska P, Pawliczak R. The participation of oxidative stress in the pathogenesis of bronchial asthma. Biomedicine and Pharmacotherapy. 2017;94:100-8.https://doi.org/10.1016/j.biopha.2017.07.066 | Excluded | Lack of relation between the title and the abstract of the article with the subject under study. |
|  | Kneale D, Harris K, McDonald VM, Thomas J, Grigg J. Effectiveness of school-based self-management interventions for asthma among children and adolescents: findings from a Cochrane systematic review and meta-analysis [with consumer summary]. Thorax 2019 May;74(5):432-438. 2019 | Excluded | Lack of relation between the title and the abstract of the article with the subject under study. |
|  | Knight KM, McGowan L, Dickens C, Bundy C. A systematic review of motivational interviewing in physical health care settings. British Journal of Health Psychology. 2006;11(2):319-32.https://doi.org/10.1348/135910705X52516 | Excluded | Lack of relation between the title and the abstract of the article with the subject under study. |
|  | Ko E, Tupper MW. Tapentadol and Sleepwalking: A Case Report. Journal of Pharmacy Practice. 2022;35(4):647-9.https://doi.org/10.1177/0897190021996975 | Excluded | Lack of relation between the title and the abstract of the article with the subject under study. |
|  | Koehle M, Lloyd-Smith R, McKenzie D, Taunton J. Asthma and recreational SCUBA diving: a systematic review. Sports Med. 2003;33(2):109-16.https://doi.org/10.2165/00007256-200333020-00003 | Excluded | Lack of relation between the title and the abstract of the article with the subject under study. |
|  | Koh J, Phyland D, Baxter M, Leong P, Bardin PG. Vocal cord dysfunction/inducible laryngeal obstruction: novel diagnostics and therapeutics. Expert Review of Respiratory Medicine. 2023;17(6):429-45.https://doi.org/10.1080/17476348.2023.2215434 | Excluded | Lack of relation between the title and the abstract of the article with the subject under study. |
|  | Köhler R, Kaistha BP, Wulff H. Vascular KCa-channels as therapeutic targets in hypertension and restenosis disease. Expert Opinion on Therapeutic Targets. 2010;14(2):143-55.https://doi.org/10.1517/14728220903540257 | Excluded | Lack of relation between the title and the abstract of the article with the subject under study. |
|  | Kokubu H, Maeda Y, Kato M, Asada H, Kobayashi Y, Takahashi T, et al. Eosinophilic granulomatosis with polyangiitis presenting with eosinophilic myositis: A case report and review of literature. Dermatologica Sinica. 2024;42(3):250-1.https://doi.org/10.4103/ds.DS-D-24-00026 | Excluded | Lack of relation between the title and the abstract of the article with the subject under study. |
|  | Konstantaki E, Priftis KN, Antonogeorgos G, Papoutsakis C, Drakouli M, Matziou V. The association of sedentary lifestyle with childhood asthma. The role of nurse as educator. Allergologia et Immunopathologia. 2014;42(6):609-15.https://doi.org/10.1016/j.aller.2013.05.008 | Excluded | Lack of relation between the title and the abstract of the article with the subject under study. |
|  | Kopp MV, Muche-Borowski C, Abou-Dakn M, Ahrens B, Beyer K, Bluemchen K, et al. S3 Guideline Allergy Prevention. Allergologie Select. 2022;6:61-97.https://doi.org/10.5414/alx02303e | Excluded | Lack of relation between the title and the abstract of the article with the subject under study. |
|  | Korang SK, Baker M, Feinberg J, Newth CJL, Khemani RG, Jakobsen JC. Non-invasive positive pressure ventilation for acute asthma in children (Cochrane review) [with consumer summary]. Cochrane Database of Systematic Reviews 2024;Issue 10. 2024 | Excluded | Lack of relation between the title and the abstract of the article with the subject under study. |
|  | Korkmazov AM, Lengina MA, Korkmazov MY, Kornova NV. The effect of targeted therapy on the quality of life of patients with polypous rhinosinusitis and comorbid bronchial asthma. Meditsinskiy Sovet. 2024;18(9):38-46.https://doi.org/10.21518/ms2024-208 | Excluded | Lack of relation between the title and the abstract of the article with the subject under study. |
|  | Köse N, Yildirim T. Acute coronary syndrome because of a scorpion sting in a patient with chronic coronary syndrome: A case report and review of the literature. Turk Kardiyoloji Dernegi Arsivi. 2021;49(4):328-33.https://doi.org/10.5543/TKDA.2021.08834 | Excluded | Lack of relation between the title and the abstract of the article with the subject under study. |
|  | Kosinski M, Nelson LM, Stanford RH, Flom JD, Schatz M. Patient-Reported Outcome Measure Development and Validation: A Primer for Clinicians. Journal of Allergy and Clinical Immunology: In Practice. 2024;12(10):2554-61.https://doi.org/10.1016/j.jaip.2024.08.030 | Excluded | Lack of relation between the title and the abstract of the article with the subject under study. |
|  | Koskela HO. Cold air-provoked respiratory symptoms: the mechanisms and management. International journal of circumpolar health. 2007;66(2):91-100.https://doi.org/10.3402/ijch.v66i2.18237 | Excluded | Lack of relation between the title and the abstract of the article with the subject under study. |
|  | Kotecha S, Clemm H, Halvorsen T, Kotecha SJ. Bronchial hyper-responsiveness in preterm-born subjects: A systematic review and meta-analysis. Pediatric Allergy and Immunology. 2018;29(7):715-25.https://doi.org/10.1111/pai.12957 | Excluded | Lack of relation between the title and the abstract of the article with the subject under study. |
|  | Koulouris NG, Koutsoukou A, D'Angelo E. Clinical uses of heliox mixtures in chronic obstructive pulmonary disease. Current Respiratory Medicine Reviews. 2009;5(3):168-73.https://doi.org/10.2174/157339809788922379 | Excluded | Lack of relation between the title and the abstract of the article with the subject under study. |
|  | Kouri A, Wong EKC, Sale JEM, Straus SE, Gupta S. Are older adults considered in asthma and chronic obstructive pulmonary disease mobile health research? A scoping review. Age and Ageing. 2023;52(9).https://doi.org/10.1093/ageing/afad144 | Excluded | Lack of relation between the title and the abstract of the article with the subject under study. |
|  | Kraushaar C, Kornblum-Hautkappe A. Chest physiotherapy in paediatrics. Atemwegs- und Lungenkrankheiten. 2023;49(8):392-400.https://doi.org/10.5414/ATX02715 | Excluded | Lack of relation between the title and the abstract of the article with the subject under study. |
|  | Kravchenko EN, Lautenschleger EV, Lautenschleger LV. Prolongation of the pelvic organs. Literature review. Russian Journal of Human Reproduction. 2024;30(2):122-9.https://doi.org/10.17116/repro202430021122 | Excluded | Lack of relation between the title and the abstract of the article with the subject under study. |
|  | Krishna S, Boren SA, Balas EA. Healthcare via cell phones: A systematic review. Telemedicine and e-Health. 2009;15(3):231-40.https://doi.org/10.1089/tmj.2008.0099 | Excluded | Lack of relation between the title and the abstract of the article with the subject under study. |
|  | Kroll JL, Ritz T. Asthma, the central nervous system, and neurocognition: Current findings, potential mechanisms, and treatment implications. Neuroscience and Biobehavioral Reviews. 2023;146.https://doi.org/10.1016/j.neubiorev.2023.105063 | Excluded | Lack of relation between the title and the abstract of the article with the subject under study. |
|  | Krüger K, Heintze C, Gehrke-Beck S, Holzinger F. Acute Cough in Adult Patients. Zeitschrift fur Allgemeinmedizin. 2022;98(5):169-77.https://doi.org/10.53180/zfa.2022.0169-0177 | Excluded | Lack of relation between the title and the abstract of the article with the subject under study. |
|  | Krüger K, Holzinger F, Trauth J, Koch M, Heintze C, Gehrke-Beck S. Chronic Cough. Deutsches Arzteblatt International. 2022;119(5):59-65.https://doi.org/10.3238/arztebl.m2021.0396 | Excluded | Lack of relation between the title and the abstract of the article with the subject under study. |
|  | Krupp NL, Fiscus C, Webb R, Webber EC, Stanley T, Pettit R, et al. Multifaceted quality improvement initiative to decrease pediatric asthma readmissions. Journal of Asthma. 2017;54(9):911-8.https://doi.org/10.1080/02770903.2017.1281294 | Excluded | Lack of relation between the title and the abstract of the article with the subject under study. |
|  | Kuder MM, Nyenhuis SM. Optimizing lifestyle interventions in adult patients with comorbid asthma and obesity. Therapeutic Advances in Respiratory Disease. 2020;14.https://doi.org/10.1177/1753466620906323 | Excluded | Lack of relation between the title and the abstract of the article with the subject under study. |
|  | Kuepper T, Morrison A, Gieseler U, Schoeffl V. Sport climbing with pre-existing cardio-pulmonary medical conditions. International Journal of Sports Medicine. 2009;30(6):395-402.https://doi.org/10.1055/s-0028-1112143 | Excluded | Lack of relation between the title and the abstract of the article with the subject under study. |
|  | Kujala UM. Evidence for exercise therapy in the treatment of chronic disease based on at least three randomized controlled trials - Summary of published systematic reviews. Scandinavian Journal of Medicine and Science in Sports. 2004;14(6):339-45.https://doi.org/10.1111/j.1600-0838.2004.00413.x | Excluded | Lack of relation between the title and the abstract of the article with the subject under study. |
|  | Kurihara K, Tsugawa J, Ouma S, Ogata T, Aoki M, Omoto M, et al. Eosinophilic Granulomatosis with Polyangiitis Presenting with Myocarditis as an Initial Symptom: A Case Report and Review of the Literature. Case Reports in Neurology. 2021;13(2):329-33.https://doi.org/10.1159/000516255 | Excluded | Lack of relation between the title and the abstract of the article with the subject under study. |
|  | Kurin M, Wiesen J, Mehta AC. Yellow nail syndrome: a case report and review of treatment options. Clinical Respiratory Journal. 2017;11(4):405-10.https://doi.org/10.1111/crj.12354 | Excluded | Lack of relation between the title and the abstract of the article with the subject under study. |
|  | Kwa EK, Cheong SK, Ong LK, Lee PF. Comparing Guided and Non-guided Deep Breathing Impact on Disability Well-Being: A Systematic Review. Journal of Medical and Biological Engineering. 2024;44(5):635-54.https://doi.org/10.1007/s40846-024-00901-8 | Excluded | Lack of relation between the title and the abstract of the article with the subject under study. |
|  | Kytikova OY, Denisenko YK, Novgorodtseva TP, Kovalenko IS, Antonyuk MV. Polyunsaturated fatty acids and lipid mediators controlling chronic inflammation in asthma. Russian Open Medical Journal. 2023;12(2).https://doi.org/10.15275/rusomj.2023.0201 | Excluded | Lack of relation between the title and the abstract of the article with the subject under study. |
|  | Labre MP, Herman EJ, Dumitru GG, Valenzuela KA, Cechman CL. Public health interventions for asthma: an umbrella review, 1990 to 2010. American Journal of Preventive Medicine 2012 Apr;42(4):403-410. 2012 | Excluded | Lack of relation between the title and the abstract of the article with the subject under study. |
|  | Lachat C, Otchere S, Roberfroid D, Abdulai A, Maria F, Seret A, et al. Diet and Physical Activity for the Prevention of Noncommunicable Diseases in Low- and Middle-Income Countries: A Systematic Policy Review. Plos Medicine. 2013;10(6).https://doi.org/10.1371/journal.pmed.1001465 | Excluded | Lack of relation between the title and the abstract of the article with the subject under study. |
|  | Lachowicz JI, Milia S, Jaremko M, Oddone E, Cannizzaro E, Cirrincione L, et al. Cooking Particulate Matter: A Systematic Review on Nanoparticle Exposure in the Indoor Cooking Environment. Atmosphere. 2023;14(1).https://doi.org/10.3390/atmos14010012 | Excluded | Lack of relation between the title and the abstract of the article with the subject under study. |
|  | Laçinel Gürlevik S, Günbey C, Ozsurekci Y, Oygar PD, Kesici S, Gocmen R, et al. Neurologic manifestations in children with COVID-19 from a tertiary center in Turkey and literature review. European Journal of Paediatric Neurology. 2022;37:139-54.https://doi.org/10.1016/j.ejpn.2022.02.003 | Excluded | Lack of relation between the title and the abstract of the article with the subject under study. |
|  | Lahart IM, Metsios GS. Chronic physiological effects of swim training interventions in non-elite swimmers: a systematic review and meta-analysis [with consumer summary]. Sports Medicine 2018 Feb;48(2):337-359. 2018 | Excluded | Lack of relation between the title and the abstract of the article with the subject under study. |
|  | Lai ZH, Ding KQ, Tu XQ, Song YY, Zeng LL. Idiopathic hypereosinophilic syndrome presenting as capsular warning syndrome: A case report and literature review. Medicine (United States). 2023;102(36):E34682.https://doi.org/10.1097/MD.0000000000034682 | Excluded | Lack of relation between the title and the abstract of the article with the subject under study. |
|  | Lalwani L, Mishra G, Gaidhane A, Quazi N, Taksande A. Chest physiotherapy in patients admitted to the intensive care unit with covid-19: A review. Open Public Health Journal. 2021;14:145-8.https://doi.org/10.2174/1874944502114010145 | Excluded | Lack of relation between the title and the abstract of the article with the subject under study. |
|  | Lama PJ. Systemic adverse effects of beta-adrenergic blockers: An evidence-based assessment. American Journal of Ophthalmology. 2002;134(5):749-60.https://doi.org/10.1016/S0002-9394(02)01699-9 | Excluded | Lack of relation between the title and the abstract of the article with the subject under study. |
|  | Lamberton CE, Mosher CL. Review of the Evidence for Pulmonary Rehabilitation in COPD: Clinical Benefits and Cost-Effectiveness. Respiratory Care. 2024;69(6):686-96.https://doi.org/10.4187/respcare.11541 | Excluded | Lack of relation between the title and the abstract of the article with the subject under study. |
|  | Lammi V, Ollila HM. Tackling Long COVID using international host genetics research collaboration. Sleep Medicine. 2022;100:S64-S5.https://doi.org/10.1016/j.sleep.2022.05.184 | Excluded | Lack of relation between the title and the abstract of the article with the subject under study. |
|  | Lamy O, Burnand B. Clinical practice guidelines: Do they help improving medical practice? Medecine et Hygiene. 2004;62(2506):2363-6 | Excluded | Lack of relation between the title and the abstract of the article with the subject under study. |
|  | Lang JE. Obesity, nutrition, and asthma in children. Pediatric, Allergy, Immunology, and Pulmonology. 2012;25(2):64-75.https://doi.org/10.1089/ped.2011.0137 | Excluded | Lack of relation between the title and the abstract of the article with the subject under study. |
|  | Langan SM, Thomas KS, Williams HC. What is meant by a "flare" in atopic dermatitis? A systematic review and proposal. Archives of Dermatology. 2006;142(9):1190-6.https://doi.org/10.1001/archderm.142.9.1190 | Excluded | Lack of relation between the title and the abstract of the article with the subject under study. |
|  | Lapiz-Bluhm MD, Nguyen TK. Systematic Review of Filipino-American (FA) Health Issues: Informing Clinical and Research Focus. Journal of Nursing Practice Applications &amp; Reviews of Research. 2020;10(1):14-39.https://doi.org/10.13178/jnparr.2020.10.01.1004 | Excluded | Lack of relation between the title and the abstract of the article with the subject under study. |
|  | Larsson J, Anderson SD, Dahlén SE, Dahlén B. Refractoriness to Exercise Challenge. A Review of the Mechanisms Old and New. Immunology and Allergy Clinics of North America. 2013;33(3):329-45.https://doi.org/10.1016/j.iac.2013.02.004 | Excluded | Lack of relation between the title and the abstract of the article with the subject under study. |
|  | LaRusso L. On the Edge. Nursing for Women's Health. 2013;17(6):471-7.https://doi.org/10.1111/1751-486X.12075 | Excluded | Lack of relation between the title and the abstract of the article with the subject under study. |
|  | LaRusso L. New Systematic Review of Midwifery Care. Nursing for Women's Health. 2013;17(6):471-7.https://doi.org/10.1111/1751-486X.12075 | Excluded | Lack of relation between the title and the abstract of the article with the subject under study. |
|  | Laveneziana P, Albuquerque A, Aliverti A, Babb T, Barreiro E, Dres M, et al. ERS statement on respiratory muscle testing at rest and during exercise. European Respiratory Journal. 2019;53(6).https://doi.org/10.1183/13993003.01214-2018 | Excluded | Lack of relation between the title and the abstract of the article with the subject under study. |
|  | Law E, Fisher E, Eccleston C, Palermo TM. Psychological interventions for parents of children and adolescents with chronic illness. Cochrane Database of Systematic Reviews. 2019(3).https://doi.org/10.1002/14651858.CD009660.pub4 | Excluded | Lack of relation between the title and the abstract of the article with the subject under study. |
|  | Leal RCAC, Braile DM, Souza DRS, Batigália F. Assistance model for patients with asthma in primary care. Revista da Associacao Medica Brasileira. 2011;57(6):683-7.https://doi.org/10.1016/s0104-4230(11)70137-3 | Excluded | Lack of relation between the title and the abstract of the article with the subject under study. |
|  | Learman LA. Chronic pelvic pain - Part 2: An integrated management approach. Advanced Studies in Medicine. 2005;5(7):360-6 | Excluded | Lack of relation between the title and the abstract of the article with the subject under study. |
|  | Ledford DK. Self-contained underwater breathing apparatus diving and asthma: Where are we in 2023? Annals of Allergy, Asthma and Immunology. 2023;130(4):463-9.https://doi.org/10.1016/j.anai.2023.01.023 | Excluded | Lack of relation between the title and the abstract of the article with the subject under study. |
|  | Lee C, Alexander E, Lee R, Okorocha N, Manikam L, Lakhanpaul M. Behavioral interventions for asthma self-management in South Asian populations: a systematic review. Journal of Asthma. 2021;58(1):112-20.https://doi.org/10.1080/02770903.2019.1658209 | Excluded | Lack of relation between the title and the abstract of the article with the subject under study. |
|  | Lee CS, Westland H, Faulkner KM, Iovino P, Thompson JH, Sexton J, et al. The effectiveness of self-care interventions in chronic illness: A meta-analysis of randomized controlled trials. International Journal of Nursing Studies. 2022;134.https://doi.org/10.1016/j.ijnurstu.2022.104322 | Excluded | Lack of relation between the title and the abstract of the article with the subject under study. |
|  | Lee JH, An J, Won HK, Kang Y, Kwon HS, Kim TB, et al. Prevalence and impact of comorbid laryngeal dysfunction in asthma: A systematic review and meta-analysis. Journal of Allergy and Clinical Immunology. 2020;145(4):1165-73.https://doi.org/10.1016/j.jaci.2019.12.906 | Excluded | Lack of relation between the title and the abstract of the article with the subject under study. |
|  | Lee S, Rhim N. Systematic review on health effects of climate change and air pollution among people with disability. Environmental Epidemiology. 2024;8:69 | Excluded | Lack of relation between the title and the abstract of the article with the subject under study. |
|  | Lee SK, Lee SH, Park BM, Yang BS, Kim JH, Lee HM. Ogilvie’s Syndrome after Lumbar Spinal Surgery. Journal of Korean Society of Spine Surgery. 2019;26(2):63-7.https://doi.org/10.4184/jkss.2019.26.2.63 | Excluded | Lack of relation between the title and the abstract of the article with the subject under study. |
|  | Lehrer PM, Kaur K. Training in slow (6/min) breathing improves general resilience. Psychosomatic Medicine. 2019;81(4):A188.https://doi.org/10.1097/PSY.0000000000000699 | Excluded | Lack of relation between the title and the abstract of the article with the subject under study. |
|  | Lehrer PM, Sargunaraj D, Hochron S. Psychological Approaches to the Treatment of Asthma. Journal of Consulting and Clinical Psychology. 1992;60(4):639-43.https://doi.org/10.1037/0022-006X.60.4.639 | Excluded | Lack of relation between the title and the abstract of the article with the subject under study. |
|  | Leinaar E, Alamian A, Wang L. A systematic review of the relationship between asthma, overweight, and the effects of physical activity in youth. Annals of Epidemiology. 2016;26(7):504-10.e6.https://doi.org/10.1016/j.annepidem.2016.06.002 | Excluded | Lack of relation between the title and the abstract of the article with the subject under study. |
|  | Leiser A, Wade A, Kulick-Soper C, Catalano LM. NSAID-EXACERBATED RESPIRATORY DISEASE REQUIRING INTUBATION. Chest. 2023;164(4):A5090-A1.https://doi.org/10.1016/j.chest.2023.07.3296 | Excluded | Lack of relation between the title and the abstract of the article with the subject under study. |
|  | Leitl D, Jarosch I, Gloeckl R, Schneeberger T, Rembert Koczulla A. Rehabilitation in pneumology. Pneumologe. 2021;18(4):241-50.https://doi.org/10.1007/s10405-021-00395-0 | Excluded | Lack of relation between the title and the abstract of the article with the subject under study. |
|  | Lemieux SÈ, Fournier-Ross E, Celis-Preciado CA. Redefining asthma management: Remodeling, rehabilitation and remission. Canadian Journal of Respiratory, Critical Care, and Sleep Medicine. 2024;8(4):179-82.https://doi.org/10.1080/24745332.2024.2359990 | Excluded | Lack of relation between the title and the abstract of the article with the subject under study. |
|  | Lendl L, Barton AK. Equine Asthma Diagnostics: Review of Influencing Factors and Difficulties in Diagnosing Subclinical Disease. Animals. 2024;14(23).https://doi.org/10.3390/ani14233504 | Excluded | Lack of relation between the title and the abstract of the article with the subject under study. |
|  | Leung TNH, Cheng JWCH, Chan AKC. Paediatrics: How to manage obstructive sleep apnoea syndrome. Drugs in Context. 2021;10.https://doi.org/10.7573/DIC.2020-12-5 | Excluded | Lack of relation between the title and the abstract of the article with the subject under study. |
|  | Levenson JL. Psychiatric issues in pulmonary disease. Primary Psychiatry. 2007;14(3):25-8 | Excluded | Lack of relation between the title and the abstract of the article with the subject under study. |
|  | Leving MT, Kocks J, Bosnic-Anticevich S, Dekhuijzen R, Usmani OS. Relationship between Peak Inspiratory Flow and Patient and Disease Characteristics in Individuals with COPD-A Systematic Scoping Review. Biomedicines. 2022;10(2).https://doi.org/10.3390/biomedicines10020458 | Excluded | Lack of relation between the title and the abstract of the article with the subject under study. |
|  | Lewis MJ, Short AL, Lewis KE. Autonomic nervous system control of the cardiovascular and respiratory systems in asthma. Respiratory Medicine. 2006;100(10):1688-705.https://doi.org/10.1016/j.rmed.2006.01.019 | Excluded | Lack of relation between the title and the abstract of the article with the subject under study. |
|  | Lewthwaite H, Byrne A, Brew B, Gibson PG. Treatable traits for long COVID. Respirology. 2023;28(11):1005-22.https://doi.org/10.1111/resp.14596 | Excluded | Lack of relation between the title and the abstract of the article with the subject under study. |
|  | Li P, Chen Z, Zhu W. Dose-response Effects Of Exercises In Children With Asthma: A Review: 1899 Board #160 May 31 2:00 PM - 3:30 PM. Medicine &amp; Science in Sports &amp; Exercise. 2018;50:456- | Excluded | Lack of relation between the title and the abstract of the article with the subject under study. |
|  | Li T, Chen J. Research Trends on Pulmonary Rehabilitation: A Bibliometric Analysis From 2011 to 2020. Frontiers in Medicine. 2022;9.https://doi.org/10.3389/fmed.2022.887793 | Excluded | Lack of relation between the title and the abstract of the article with the subject under study. |
|  | Li T, Wang Y, Zhu Y, Zhao Q. Study on early intervention strategies of children's snoring: a meta-analysis based on network. Minerva Pediatrics. 2024;76(3):457-9.https://doi.org/10.23736/S2724-5276.24.07533-5 | Excluded | Lack of relation between the title and the abstract of the article with the subject under study. |
|  | Li W, Wang X, Diao H, Yang Y, Ding L, Huan W, et al. Systemic immune inflammation index with all-cause and cause-specific mortality: a meta-analysis. Inflammation Research. 2024;73(12):2199-216.https://doi.org/10.1007/s00011-024-01959-5 | Excluded | Lack of relation between the title and the abstract of the article with the subject under study. |
|  | Liacouras CA, Furuta GT, Hirano I, Atkins D, Attwood SE, Bonis PA, et al. Eosinophilic esophagitis: Updated consensus recommendations for children and adults. Journal of Allergy and Clinical Immunology. 2011;128(1):3-20.e6.https://doi.org/10.1016/j.jaci.2011.02.040 | Excluded | Lack of relation between the title and the abstract of the article with the subject under study. |
|  | Liang CW, Cheng HY, Lee YH, Liou TH, Liao CD, Huang SW. Effects of conjugated linoleic acid and exercise on body composition and obesity: a systematic review and meta-analysis. Nutrition Reviews. 2023;81(4):397-415.https://doi.org/10.1093/nutrit/nuac060 | Excluded | Lack of relation between the title and the abstract of the article with the subject under study. |
|  | Liao QN, Fang ZK, Chen SB, Fan HZ, Chen LC, Wu XP, et al. Pleomorphic adenoma of the trachea: A case report and review of the literature. World Journal of Clinical Cases. 2020;8(23):6026-35.https://doi.org/10.12998/wjcc.v8.i23.6026 | Excluded | Lack of relation between the title and the abstract of the article with the subject under study. |
|  | Lim JJ, Lim YYE, Ng JY, Malipeddi P, Ng YT, Teo WY, et al. An update on the prevalence, chronicity, and severity of atopic dermatitis and the associated epidemiological risk factors in the Singapore/Malaysia Chinese young adult population: A detailed description of the Singapore/Malaysia Cross-Sectional Genetics Epidemiology Study (SMCGES) cohort. The World Allergy Organization journal. 2022;15(12):100722.https://doi.org/10.1016/j.waojou.2022.100722 | Excluded | Lack of relation between the title and the abstract of the article with the subject under study. |
|  | Lim WJ, Mohammed Akram R, Carson KV, Mysore S, Labiszewski NA, Wedzicha JA, et al. Non-invasive positive pressure ventilation for treatment of respiratory failure due to severe acute exacerbations of asthma (Cochrane review) [with consumer summary]. Cochrane Database of Systematic Reviews 2012;Issue 12. 2012 | Excluded | Lack of relation between the title and the abstract of the article with the subject under study. |
|  | Lima LBBM, editor Accessible tourism supply services in spain. Proceedings of the International Conference on Tourism Research; 2019. | Excluded | Lack of relation between the title and the abstract of the article with the subject under study. |
|  | Lin W, Tay SH, Mak A. Takotsubo syndrome and rheumatic diseases - A critical systematic review. Rheumatology (United Kingdom). 2021;60(1):11-22.https://doi.org/10.1093/rheumatology/keaa504 | Excluded | Lack of relation between the title and the abstract of the article with the subject under study. |
|  | Lindblad V, Kragholm KH, Gaardsted PS, Hansen LEM, Lauritzen FF, Melgaard D. From illness to inactivity: Exploring the influence of physical diseases on youth Not in Education, Employment, or Training status in Europe: A systematic literature review. Journal of Adolescence. 2024;96(8):1695-712.https://doi.org/10.1002/jad.12386 | Excluded | Lack of relation between the title and the abstract of the article with the subject under study. |
|  | Ling MYJ, Ahmad N, Aizuddin AN. Risk perception of non-communicable diseases: A systematic review on its assessment and associated factors. PLoS ONE. 2023;18(6 JUNE).https://doi.org/10.1371/journal.pone.0286518 | Excluded | Lack of relation between the title and the abstract of the article with the subject under study. |
|  | Ling T, Li JJ, Xu RJ, Wang B, Ge WH. Topical diclofenac solution for osteoarthritis of the knee: An updated meta-analysis of randomized controlled trials. BioMed Research International. 2020;2020.https://doi.org/10.1155/2020/1758071 | Excluded | Lack of relation between the title and the abstract of the article with the subject under study. |
|  | Liu F, Liu YR, Liu L. Effect of exercise rehabilitation on exercise capacity and quality of life in children with bronchial asthma: A systematic review. Chinese Journal of Contemporary Pediatrics. 2021;23(10):1050-7.https://doi.org/10.7499/j.issn.1008-8830.2104124 | Excluded | Lack of relation between the title and the abstract of the article with the subject under study. |
|  | Liu P-C, Kieckhefer GM, Gau B-S. A systematic review of the association between obesity and asthma in children. Journal of Advanced Nursing. 2013;69(7):1446-65.https://doi.org/10.1111/jan.12129 | Excluded | Lack of relation between the title and the abstract of the article with the subject under study. |
|  | Liu X, Helenius D, Skotte L, Beaumont RN, Wielscher M, Geller F, et al. Variants in the fetal genome near pro-inflammatory cytokine genes on 2q13 associate with gestational duration. Nature Communications. 2019;10(1).https://doi.org/10.1038/s41467-019-11881-8 | Excluded | Lack of relation between the title and the abstract of the article with the subject under study. |
|  | Liu X, Liu J, Nai T, Yang Y, Hu Y. Primary ectopic meningioma in the thoracic cavity: A rare case report and review of the literature. Frontiers in Oncology. 2023;13.https://doi.org/10.3389/fonc.2023.1149627 | Excluded | Lack of relation between the title and the abstract of the article with the subject under study. |
|  | Liu X, Wang L, Zhou K, Hua Y, Shi X, Wang C. A delayed diagnosis of eosinophilic granulomatosis with polyangiitis complicated with extensive artery occlusion of lower extremities in children: Case report and literature review. Pediatric Rheumatology. 2019;17(1).https://doi.org/10.1186/s12969-019-0331-8 | Excluded | Lack of relation between the title and the abstract of the article with the subject under study. |
|  | Liu X-C, Pan L, Hu Q, Dong W-P, Yan J-H, Dong L. Effects of yoga training in patients with chronic obstructive pulmonary disease: a systematic review and meta-analysis. Journal of Thoracic Disease. 2014;6(6):795-802.https://doi.org/10.3978/j.issn.2072-1439.2014.06.05 | Excluded | Lack of relation between the title and the abstract of the article with the subject under study. |
|  | Livingston R, Bellas H, Sahota J, Bidder T, Robinson D. Physical activity levels in a severe asthma population. European Respiratory Journal. 2017;50.https://doi.org/10.1183/1393003.congress-2017.PA4701 | Excluded | Lack of relation between the title and the abstract of the article with the subject under study. |
|  | Lochte L, Granell R, C Sterne JA, Henderson AJ. Childhood incident asthma and physical activity: A systematic literature review and meta-analysis with heterogeneity assessments. Thorax. 2010;65:A39-A40.https://doi.org/10.1136/thx.2010.150938.35 | Excluded | Lack of relation between the title and the abstract of the article with the subject under study. |
|  | Lochte L, Nielsen KG, Petersen PE, Platts-Mills TA. Childhood asthma and physical activity: a systematic review with meta-analysis and Graphic Appraisal Tool for Epidemiology assessment. BMC Pediatr. 2016;16:50.https://doi.org/10.1186/s12887-016-0571-4 | Excluded | Lack of relation between the title and the abstract of the article with the subject under study. |
|  | Lorenc AB, Wang Y, Madge SL, Hu X, Mian AM, Robinson N. Meditative movement for respiratory function: A systematic review. Respiratory Care. 2014;59(3):427-40.https://doi.org/10.4187/respcare.02570 | Excluded | Lack of relation between the title and the abstract of the article with the subject under study. |
|  | Loth DW, Artigas MS, Gharib SA, Wain LV, Franceschini N, Koch B, et al. Genome-wide association analysis identifies six new loci associated with forced vital capacity. Nature Genetics. 2014;46(7):669-77.https://doi.org/10.1038/ng.3011 | Excluded | Lack of relation between the title and the abstract of the article with the subject under study. |
|  | Loxham M, Nieuwenhuijsen MJ. Health effects of particulate matter air pollution in underground railway systems- A critical review of the evidence. Particle and Fibre Toxicology. 2019;16(1).https://doi.org/10.1186/s12989-019-0296-2 | Excluded | Lack of relation between the title and the abstract of the article with the subject under study. |
|  | Lucas SR, Platts-Mills TAE. Paediatric asthma and obesity. Paediatric Respiratory Reviews. 2006;7(4):233-8.https://doi.org/10.1016/j.prrv.2006.08.001 | Excluded | Lack of relation between the title and the abstract of the article with the subject under study. |
|  | Luks V, Burkett A, Turner L, Pakhale S. Effect of physical training on airway inflammation in animal models of asthma: A systematic review. BMC Pulmonary Medicine. 2013;13(1).https://doi.org/10.1186/1471-2466-13-24 | Excluded | Lack of relation between the title and the abstract of the article with the subject under study. |
|  | Luo Q, Zhang S, Yang Q, Deng Y, Yi H, Li X. Causal factors for osteoarthritis risk revealed by mendelian randomization analysis. Aging Clinical and Experimental Research. 2024;36(1).https://doi.org/10.1007/s40520-024-02812-9 | Excluded | Lack of relation between the title and the abstract of the article with the subject under study. |
|  | Lupu VV, Lupu A, Jechel E, Starcea IM, Stoleriu G, Ioniuc I, et al. The role of vitamin D in pediatric systemic lupus erythematosus - a double pawn in the immune and microbial balance. Frontiers in Immunology. 2024;15.https://doi.org/10.3389/fimmu.2024.1373904 | Excluded | Lack of relation between the title and the abstract of the article with the subject under study. |
|  | Luz MI, Aguiar R, Morais-Almeida M. The reality of LAMAs for adult asthmatic patients. Expert Review of Respiratory Medicine. 2020:1087-94.https://doi.org/10.1080/17476348.2020.1794828 | Excluded | Lack of relation between the title and the abstract of the article with the subject under study. |
|  | Lv N, Xiao L, Ma J. Weight Management Interventions in Adult and Pediatric Asthma Populations: A Systematic Review. Journal of pulmonary & respiratory medicine. 2015;5(232).https://doi.org/10.4172/2161-105x.1000232 | Excluded | Lack of relation between the title and the abstract of the article with the subject under study. |
|  | Ma X, Longley I, Gao J, Salmond J. Assessing schoolchildren's exposure to air pollution during the daily commute - A systematic review. Science of the Total Environment. 2020;737.https://doi.org/10.1016/j.scitotenv.2020.140389 | Excluded | Lack of relation between the title and the abstract of the article with the subject under study. |
|  | Maan AA, Nazir A, Khan MKI, Ahmad T, Zia R, Murid M, et al. The therapeutic properties and applications of Aloe vera: A review. Journal of Herbal Medicine. 2018;12:1-10.https://doi.org/10.1016/j.hermed.2018.01.002 | Excluded | Lack of relation between the title and the abstract of the article with the subject under study. |
|  | Mac TT, Castinetti F, Bar C, Julia S, Pasquet M, Romanet P, et al. Deficient anterior pituitary with common variable immune deficiency (DAVID syndrome): a new case and literature reports. Journal of Neuroendocrinology. 2023;35(6).https://doi.org/10.1111/jne.13287 | Excluded | Lack of relation between the title and the abstract of the article with the subject under study. |
|  | Macdonald M, Martin-Misener R, Helwig M, Smith LJ, Godfrey CM. The experiences of adults with cystic fibrosis in adhering to medication regimens: A systematic review protocol. JBI Database of Systematic Reviews and Implementation Reports. 2014;12(12):91-105.https://doi.org/10.11124/jbisrir-2014-1525 | Excluded | Lack of relation between the title and the abstract of the article with the subject under study. |
|  | Machado A, Quadflieg K, Oliveira A, Keytsman C, Marques A, Hansen D, et al. Exercise Training in Patients with Chronic Respiratory Diseases: Are Cardiovascular Comorbidities and Outcomes Taken into Account?-A Systematic Review. J Clin Med. 2019;8(9).https://doi.org/10.3390/jcm8091458 | Excluded | Lack of relation between the title and the abstract of the article with the subject under study. |
|  | Machado B, Quimbaya P, Bustos RH, Jaimes D, Cortes K, Vargas D, et al. Assessment of Medication Adherence Using Mobile Applications in Chronic Obstructive Pulmonary Disease: A Scoping Review. International Journal of Environmental Research and Public Health. 2024;21(10).https://doi.org/10.3390/ijerph21101265 | Excluded | Lack of relation between the title and the abstract of the article with the subject under study. |
|  | MacIntyre NR. Chronic obstructive pulmonary disease management: the evidence base. Respiratory care. 2001;46(11):1294-303 | Excluded | Lack of relation between the title and the abstract of the article with the subject under study. |
|  | Mackintosh KA, McNarry MA, Berntsen S, Steele J, Sejersted E, Westergren T. Physical activity and sedentary time in children and adolescents with asthma: A systematic review and meta‐analysis. Scandinavian Journal of Medicine &amp; Science in Sports. 2021;31(6):1183-95 | Excluded | Lack of relation between the title and the abstract of the article with the subject under study. |
|  | Madmani ME, Yusuf Solaiman A, Tamr Agha K, Madmani Y, Shahrour Y, Essali A, et al. Coenzyme Q10 for heart failure. Cochrane Database of Systematic Reviews. 2014;2014(6).https://doi.org/10.1002/14651858.CD008684.pub2 | Excluded | Lack of relation between the title and the abstract of the article with the subject under study. |
|  | Madsen TE, Samaei M, Pikula A, Yu AYX, Carcel C, Millsaps E, et al. Sex Differences in Physical Activity and Incident Stroke: A Systematic Review. Clinical Therapeutics. 2022;44(4):586-611.https://doi.org/10.1016/j.clinthera.2022.02.006 | Excluded | Lack of relation between the title and the abstract of the article with the subject under study. |
|  | Magalhães PA, Lanza F, Figueiredo BB. Clinical features and physiotherapy management for COVID-19 in children. Minerva Pediatrics. 2023;75(2):260-9.https://doi.org/10.23736/S2724-5276.20.06100-9 | Excluded | Lack of relation between the title and the abstract of the article with the subject under study. |
|  | Magis-Escurra C, Reijers MH. Bronchiectasis. BMJ clinical evidence. 2015;2015 | Excluded | Lack of relation between the title and the abstract of the article with the subject under study. |
|  | Magnani L, Aguglia A, Alexander J, Maiorano A, Richard-Lepouriel H, Iancau SP, et al. Evening Chronotype and Suicide: Exploring Neuroinflammation and Psychopathological Dimensions as Possible Bridging Factors—A Narrative Review. Brain Sciences. 2024;14(1).https://doi.org/10.3390/brainsci14010030 | Excluded | Lack of relation between the title and the abstract of the article with the subject under study. |
|  | Maguire A, Gopalakaje S, Eastham K. All that wheezes is not asthma: A 6-year-old with foreign body aspiration and no suggestive history. BMJ Case Reports. 2012.https://doi.org/10.1136/bcr-2012-006640 | Excluded | Lack of relation between the title and the abstract of the article with the subject under study. |
|  | Mahalleh M, Behnoush AH, Khalaji A, Gouravani M, Assempoor R, Jamshidi A, et al. Serum Galectin-3 Level in Patients with Rheumatoid Arthritis: A Systematic Review and Meta-analysis. Iranian Journal of Allergy, Asthma and Immunology. 2024;23(5):476-88.https://doi.org/10.18502/ijaai.v23i5.16777 | Excluded | Lack of relation between the title and the abstract of the article with the subject under study. |
|  | Mahoney J, Hew M, Vertigan A, Oates J. Treatment effectiveness for Vocal Cord Dysfunction in adults and adolescents: A systematic review. Clinical and Experimental Allergy. 2022;52(3):387-404.https://doi.org/10.1111/cea.14036 | Excluded | Lack of relation between the title and the abstract of the article with the subject under study. |
|  | Mahoney J, Oates J, Vertigan A, Hew M. Intervention Effectiveness for Vocal Cord Dysfunction: A Systematic Review. Respirology. 2020;25:121.https://doi.org/10.1111/resp.13778 | Excluded | Lack of relation between the title and the abstract of the article with the subject under study. |
|  | Maidment DW, Clarkson K, Shiel EV, Nielsen K, Yarker J, Munir F. A Rapid Systematic Review Assessing the Effectiveness of Interventions to Promote Self-Management in Workers with Long-Term Health Conditions and Disabilities. International Journal of Environmental Research and Public Health. 2024;21(12).https://doi.org/10.3390/ijerph21121714 | Excluded | Lack of relation between the title and the abstract of the article with the subject under study. |
|  | Mair JL, Salamanca-Sanabria A, Augsburger M, Frese BF, Abend S, Jakob R, et al. Effective Behavior Change Techniques in Digital Health Interventions for the Prevention or Management of Noncommunicable Diseases: An Umbrella Review. Annals of behavioral medicine : a publication of the Society of Behavioral Medicine. 2023;57(10):817-35.https://doi.org/10.1093/abm/kaad041 | Excluded | Lack of relation between the title and the abstract of the article with the subject under study. |
|  | Maison N. Asthma in childhood and adulthood. Pneumologe. 2019;16(2):98-103.https://doi.org/10.1007/s10405-019-0230-1 | Excluded | Lack of relation between the title and the abstract of the article with the subject under study. |
|  | Majeed M, Lewis G, Olang C, Lake S, Gorantla V. The Paradoxical Relationship Between Nicotine and SARS-CoV-2 Infection: A Systematic Review. International Journal of Research in Pharmaceutical Sciences. 2022;13(1):92-101.https://doi.org/10.26452/ijrps.v13i1.26 | Excluded | Lack of relation between the title and the abstract of the article with the subject under study. |
|  | Maki-Heikkila R, Karjalainen J, Parkkari J, Valtonen M, Lehtimaki L. Asthma in Competitive Cross-Country Skiers: A Systematic Review and Meta-analysis. Sports Medicine. 2020;50(11):1963-81.https://doi.org/10.1007/s40279-020-01334-4 | Excluded | Lack of relation between the title and the abstract of the article with the subject under study. |
|  | Malara G, Verduci C, Altomonte M, Cuzzola M, Trifiro C, Politi C, et al. Thalidomide and discoid lupus erythematosus: case series and review of literature. Drugs in Context. 2022;11.https://doi.org/10.7573/dic.2021-9-8 | Excluded | Lack of relation between the title and the abstract of the article with the subject under study. |
|  | Malden S, Gillespie J, Hughes A, Gibson A, Martin A, Summerbell C, et al. The relationship between obesity in early childhood and physical morbidity in childhood and adolescence: A systematic review and meta-analysis. Obesity Facts. 2019;12:217.https://doi.org/10.1159/000489691 | Excluded | Lack of relation between the title and the abstract of the article with the subject under study. |
|  | Malden S, Gillespie J, Hughes A, Gibson A-M, Farooq A, Martin A, et al. Obesity in young children and its relationship with diagnosis of asthma, vitamin D deficiency, iron deficiency, specific allergies and flat-footedness: A systematic review and meta-analysis. Obesity Reviews. 2021;22(3).https://doi.org/10.1111/obr.13129 | Excluded | Lack of relation between the title and the abstract of the article with the subject under study. |
|  | Malhotra S, Deshmukh SS, Dastidar SG. COX inhibitors for airway inflammation. Expert Opinion on Therapeutic Targets. 2012;16(2):195-207.https://doi.org/10.1517/14728222.2012.661416 | Excluded | Lack of relation between the title and the abstract of the article with the subject under study. |
|  | Malizia RW, Baumann BM, Chansky ME, Kirchhoff MA. Ambulatory Dysfunction Due to Unrecognized Pernicious Anemia. Journal of Emergency Medicine. 2010;38(3):302-7.https://doi.org/10.1016/j.jemermed.2007.05.044 | Excluded | Lack of relation between the title and the abstract of the article with the subject under study. |
|  | Maloney AE. Pediatric obesity: A review for the child psychiatrist. Child and Adolescent Psychiatric Clinics of North America. 2010;19(2):353-70.https://doi.org/10.1016/j.chc.2010.01.005 | Excluded | Lack of relation between the title and the abstract of the article with the subject under study. |
|  | Maloney AE. Pediatric obesity: A review for the child psychiatrist. Pediatric Clinics of North America. 2011;58(4):955-72.https://doi.org/10.1016/j.pcl.2011.06.005 | Excluded | Lack of relation between the title and the abstract of the article with the subject under study. |
|  | Maltseva OB, Stryapko YI, Samoilenko SM, Lyakhovets LO. THE PECULIARITY OF THE USE OF A COMPLEX OF NON-TRADITIONAL HEALING METHODS FOR PATIENTS WITH A PULMONOLOGICAL PROFILE. Rehabilitation and Recreation. 2022;2022(12):52-9.https://doi.org/10.32782/2522-1795.2022.12.7 | Excluded | Lack of relation between the title and the abstract of the article with the subject under study. |
|  | Man LX. Complementary and alternative medicine for allergic rhinitis. Current Opinion in Otolaryngology and Head and Neck Surgery. 2009;17(3):226-31.https://doi.org/10.1097/MOO.0b013e3283295791 | Excluded | Lack of relation between the title and the abstract of the article with the subject under study. |
|  | Mangla A, Agarwal N, Saei Hamedani F, Liu J, Gupta S, Mullane MR. Metastasis of cervical cancer to breast: A case report and review of literature. Gynecologic Oncology Reports. 2017;21:48-52.https://doi.org/10.1016/j.gore.2017.06.009 | Excluded | Lack of relation between the title and the abstract of the article with the subject under study. |
|  | Mangova M, Lipek T, vom Hove M, Körner A, Kiess W, Treudler R, et al. Obesity-associated asthma in childhood. Allergologie. 2021;44(1):31-40.https://doi.org/10.5414/ALX02178 | Excluded | Lack of relation between the title and the abstract of the article with the subject under study. |
|  | Manheimer E, Wieland S, Kimbrough E, Cheng K, Berman BM. Evidence from the cochrane collaboration for traditional chinese medicine therapies. Journal of Alternative and Complementary Medicine. 2009;15(9):1001-14.https://doi.org/10.1089/acm.2008.0414 | Excluded | Lack of relation between the title and the abstract of the article with the subject under study. |
|  | Mansur AH, Prasad N. Management of difficult-to-treat asthma in adolescence and young adults. Breathe. 2023;19(1).https://doi.org/10.1183/20734735.0025-2022 | Excluded | Lack of relation between the title and the abstract of the article with the subject under study. |
|  | Mao Y, Hu G, Meng Q, Li X, Sun X, Zhou J, et al. Efficacy of Shenling Baizhu San on stable chronic obstructive pulmonary disease patients: A systematic review and meta-analysis. Journal of Ethnopharmacology. 2021;272.https://doi.org/10.1016/j.jep.2021.113927 | Excluded | Lack of relation between the title and the abstract of the article with the subject under study. |
|  | Marain N, Claus R, Ronsmans S, Colemont M, Janssen L, Vanoirbeek J, et al. Diagnostic Accuracy of Cold Air Challenge Test in Airway Hyperresponsiveness: Systematic Review and Meta-analysis. American Journal of Respiratory and Critical Care Medicine. 2024;209 | Excluded | Lack of relation between the title and the abstract of the article with the subject under study. |
|  | Marcano Belisario JS, Huckvale K, Greenfield G, Car J, Gunn LH. Smartphone and tablet self management apps for asthma. Cochrane Database of Systematic Reviews. 2013(11).https://doi.org/10.1002/14651858.CD010013.pub2 | Excluded | Lack of relation between the title and the abstract of the article with the subject under study. |
|  | Marguet C, Moirez P, Cohen L, Mallet E, editors. Preliminary study of the place of breathing control in the education of adolescent asthmatics. Revue Francaise d'Allergologie et d'Immunologie Clinique; 1997. | Excluded | Lack of relation between the title and the abstract of the article with the subject under study. |
|  | Mariana M, Gaio R, Albuquerque J, Gonçalves M, Lobo L. Swyer-James-Macleod Syndrome presentating as pneumothorax. Journal of Pediatric Surgery Case Reports. 2018;37:57-9.https://doi.org/10.1016/j.epsc.2018.07.015 | Excluded | Lack of relation between the title and the abstract of the article with the subject under study. |
|  | Maricoto T, Monteiro L, Gama JMR, Correia-de-Sousa J, Taborda-Barata L. Inhaler Technique Education and Exacerbation Risk in Older Adults with Asthma or Chronic Obstructive Pulmonary Disease: A Meta-Analysis. Journal of the American Geriatrics Society. 2019;67(1):57-66.https://doi.org/10.1111/jgs.15602 | Excluded | Lack of relation between the title and the abstract of the article with the subject under study. |
|  | Marín-Gámez N, Kessel-Sardiñas H, Cervantes-Bonet B, López-Palmero S, Antón-Molina F, Martínez-García L. Biomedical research, the market, clinicians, safety and corporate social responsibility post-phase III: Maintaining confidence. Revista de Calidad Asistencial. 2010;25(3):169-72.https://doi.org/10.1016/j.cali.2010.01.003 | Excluded | Lack of relation between the title and the abstract of the article with the subject under study. |
|  | Markham AW, Wilkinson JM. Complementary and alternative medicines (CAM) in the management of asthma: an examination of the evidence. The Journal of Asthma 2004;41(2):131-139. 2004 | Excluded | Lack of relation between the title and the abstract of the article with the subject under study. |
|  | Martineau AR, Jolliffe DA, Greenberg L, Aloia JF, Bergman P, Dubnov-Raz G, et al. Vitamin D supplementation to prevent acute respiratory infections: Individual participant data meta-analysis. Health Technology Assessment. 2019;23(2):1-44.https://doi.org/10.3310/hta23020 | Excluded | Lack of relation between the title and the abstract of the article with the subject under study. |
|  | Martineau AR, Jolliffe DA, Hooper RL, Greenberg L, Aloia JF, Bergman P, et al. Vitamin D supplementation to prevent acute respiratory tract infections: Systematic review and meta-analysis of individual participant data. BMJ (Online). 2017;356.https://doi.org/10.1136/bmj.i6583 | Excluded | Lack of relation between the title and the abstract of the article with the subject under study. |
|  | Martinez CH, Han MK. Contribution of the environment and comorbidities to chronic obstructive pulmonary disease phenotypes. Medical Clinics of North America. 2012;96(4):713-27.https://doi.org/10.1016/j.mcna.2012.02.007 | Excluded | Lack of relation between the title and the abstract of the article with the subject under study. |
|  | Martorell A, Alonso E, Echeverría L, Escudero C, García-Rodríguez R, Blasco C, et al. Oral immunotherapy for food allergy: A spanish guideline. immunotherapy egg and milk spanish guide (ITEMS guide). part i: Cow milk and egg oral immunotherapy: Introduction, methodology, rationale, current state, indications, contraindications, and oral immunotherapy build-up phase. Journal of Investigational Allergology and Clinical Immunology. 2017;27(4):225-37.https://doi.org/10.18176/jiaci.0177 | Excluded | Lack of relation between the title and the abstract of the article with the subject under study. |
|  | Marye S. Health insurance, pediatric asthma, and emergency department usage. Public health nursing (Boston, Mass). 2021;38(5):931-40.https://doi.org/10.1111/phn.12926 | Excluded | Lack of relation between the title and the abstract of the article with the subject under study. |
|  | Maselli DJ, Hanania NA. Management of asthma COPD overlap. Annals of Allergy, Asthma and Immunology. 2019;123(4):335-44.https://doi.org/10.1016/j.anai.2019.07.021 | Excluded | Lack of relation between the title and the abstract of the article with the subject under study. |
|  | Massé G, Al Khaldi M, Schwenter F, Boudier-Revéret M, Sebajang H. Abdominal pseudohernia caused by thoracic disk herniation: case series and review of the literature. Journal of Surgical Case Reports. 2025;2025(1).https://doi.org/10.1093/jscr/rjae822 | Excluded | Lack of relation between the title and the abstract of the article with the subject under study. |
|  | Mattsson M, Olofsson T, Nair G, editors. Exploring the health and energy savings dilemma in swimming facilities and the potential influence of Positive Energy Districts. Journal of Physics: Conference Series; 2023. | Excluded | Lack of relation between the title and the abstract of the article with the subject under study. |
|  | Mazzoleni E, Donelli D, Zabini F, Meneguzzo F, Antonelli M. Forest Therapy Research in Europe: A Scoping Review of the Scientific Literature. Forests. 2024;15(5).https://doi.org/10.3390/f15050848 | Excluded | Lack of relation between the title and the abstract of the article with the subject under study. |
|  | McCallum GB, Morris PS, Brown N, Chang AB. Culture-specific programs for children and adults from minority groups who have asthma (Cochrane review) [with consumer summary]. Cochrane Database of Systematic Reviews 2017;Issue 8. 2017 | Excluded | Lack of relation between the title and the abstract of the article with the subject under study. |
|  | McCartney G, Thomas S, Thomson H, Scott J, Hamilton V, Hanlon P, et al. The health and socioeconomic impacts of major multi-sport events: systematic review (1978-2008). BMJ (Clinical research ed). 2010;340:c2369.https://doi.org/10.1136/bmj.c2369 | Excluded | Lack of relation between the title and the abstract of the article with the subject under study. |
|  | McDonald VM, Osadnik CR, Gibson PG. Treatable traits in acute exacerbations of chronic airway diseases. Chronic Respiratory Disease. 2019;16.https://doi.org/10.1177/1479973119867954 | Excluded | Lack of relation between the title and the abstract of the article with the subject under study. |
|  | McDonnell CJ, White KS. Assessment and treatment of psychological factors in pediatric chest pain. Pediatric Clinics of North America. 2010;57(6):1235-60.https://doi.org/10.1016/j.pcl.2010.09.010 | Excluded | Lack of relation between the title and the abstract of the article with the subject under study. |
|  | McGee DL, Wald DA, Hinchliffe S. Helium-oxygen therapy in the emergency department. Journal of Emergency Medicine. 1997;15(3):291-6.https://doi.org/10.1016/S0736-4679(97)00008-5 | Excluded | Lack of relation between the title and the abstract of the article with the subject under study. |
|  | McKay FH, Cheng C, Wright A, Shill J, Stephens H, Uccellini M. Evaluating mobile phone applications for health behaviour change: A systematic review. Journal of telemedicine and telecare. 2018;24(1):22-30.https://doi.org/10.1177/1357633X16673538 | Excluded | Lack of relation between the title and the abstract of the article with the subject under study. |
|  | McKeage K, Keam SJ. Salmeterol fluticasone propionate: A review of its use in asthma. Drugs. 2009;69(13):1799-828.https://doi.org/10.2165/11202210-000000000-00000 | Excluded | Lack of relation between the title and the abstract of the article with the subject under study. |
|  | McMurray J, Widger K, Stephenson AL, Stremler R. Actigraphic and patient and family reported sleep outcomes in children and youth with cystic fibrosis: A systematic review. Journal of Cystic Fibrosis. 2022;21(2):e49-e82.https://doi.org/10.1016/j.jcf.2021.05.005 | Excluded | Lack of relation between the title and the abstract of the article with the subject under study. |
|  | McWilliams E, Yablon D, Kesim R, Ge R, Donkoh A, Abdelnour M, et al. A systematic review of behavioral change techniques in mobile health interventions for adherence or self-management: application to people with cystic fibrosis. Journal of Cystic Fibrosis. 2022;21:S179.https://doi.org/10.1016/S1569-1993(22)00993-6 | Excluded | Lack of relation between the title and the abstract of the article with the subject under study. |
|  | Mease P, Goffe BS. Diagnosis and treatment of psoriatic arthritis. Journal of the American Academy of Dermatology. 2005;52(1):1-19.https://doi.org/10.1016/j.jaad.2004.06.013 | Excluded | Lack of relation between the title and the abstract of the article with the subject under study. |
|  | Mebrahtu TF, Feltbower RG, Greenwood DC, Parslow RC. Childhood body mass index and wheezing disorders: a systematic review and meta-analysis. Pediatric Allergy and Immunology. 2015;26(1):62-72.https://doi.org/10.1111/pai.12321 | Excluded | Lack of relation between the title and the abstract of the article with the subject under study. |
|  | Medar SS, Derespina KR, Jakobleff WA, Ushay MH, Peek GJ. A winter to remember! Extracorporeal membrane oxygenation for life-threatening asthma in children: A case series and review of literature. Pediatric Pulmonology. 2020;55(2):E1-E4.https://doi.org/10.1002/ppul.24616 | Excluded | Lack of relation between the title and the abstract of the article with the subject under study. |
|  | Medina-Gomez C, Kemp JP, Trajanoska K, Luan J, Chesi A, Ahluwalia TS, et al. Life-Course Genome-wide Association Study Meta-analysis of Total Body BMD and Assessment of Age-Specific Effects. American Journal of Human Genetics. 2018;102(1):88-102.https://doi.org/10.1016/j.ajhg.2017.12.005 | Excluded | Lack of relation between the title and the abstract of the article with the subject under study. |
|  | Medina-Gomez C, Mullin BH, Chesi A, Prijatelj V, Kemp JP, Shochat-Carvalho C, et al. Bone mineral density loci specific to the skull portray potential pleiotropic effects on craniosynostosis. Communications biology. 2023;6(1):691.https://doi.org/10.1038/s42003-023-04869-0 | Excluded | Lack of relation between the title and the abstract of the article with the subject under study. |
|  | Mehta GR, Mohammed R, Sarfraz S, Khan T, Ahmed K, Villareal M, et al. Chronic obstructive pulmonary disease: A guide for the primary care physician. Disease-a-Month. 2016;62(6):164-87.https://doi.org/10.1016/j.disamonth.2016.03.002 | Excluded | Lack of relation between the title and the abstract of the article with the subject under study. |
|  | Mengzhu P, Xiangang Y, Lili X, Guojun L. Exhaled breath condensate specimens for testing airway health during exercise. Chinese Journal of Tissue Engineering Research. 2023;27(23):3755-62.https://doi.org/10.12307/2023.582 | Excluded | Lack of relation between the title and the abstract of the article with the subject under study. |
|  | Mens JMA. The use of medication in low back pain. Best Practice and Research: Clinical Rheumatology. 2005;19(4):609-21.https://doi.org/10.1016/j.berh.2005.03.011 | Excluded | Lack of relation between the title and the abstract of the article with the subject under study. |
|  | Menson KE, Dowman L. Pulmonary Rehabilitation for Diseases Other Than COPD. Journal of Cardiopulmonary Rehabilitation and Prevention. 2024;44(6):425-31.https://doi.org/10.1097/HCR.0000000000000915 | Excluded | Lack of relation between the title and the abstract of the article with the subject under study. |
|  | Menzies-Gow A, Moore WC, Wechsler ME. Difficult-to-Control Asthma Management in Adults. Journal of Allergy and Clinical Immunology: In Practice. 2022;10(2):378-84.https://doi.org/10.1016/j.jaip.2021.12.007 | Excluded | Lack of relation between the title and the abstract of the article with the subject under study. |
|  | Merison K, Jacobs H. Diagnosis and Treatment of Childhood Migraine. Current Treatment Options in Neurology. 2016;18(11).https://doi.org/10.1007/s11940-016-0431-4 | Excluded | Lack of relation between the title and the abstract of the article with the subject under study. |
|  | Mesa MD, Loureiro B, Iglesia I, Gonzalez SF, Olivé EL, Algar OG, et al. The evolving microbiome from pregnancy to early infancy: A comprehensive review. Nutrients. 2020;12(1).https://doi.org/10.3390/nu12010133 | Excluded | Lack of relation between the title and the abstract of the article with the subject under study. |
|  | Miceli Sopo S, Battista A, Greco M, Monaco S. Grass pollen sublingual immunotherapy and paediatric allergic rhinitis: A patient-oriented decision. Allergologia et Immunopathologia. 2016;44(4):382-6.https://doi.org/10.1016/j.aller.2015.05.004 | Excluded | Lack of relation between the title and the abstract of the article with the subject under study. |
|  | Michaelchuk W, Quach S, Benoit A, Maybank A, Olivera A, Goldstein R, et al. Systematic review and meta-analysis of interactive digital self-management interventions for chronic respiratory disease. Canadian Journal of Respiratory, Critical Care, and Sleep Medicine. 2023;7:35-6.https://doi.org/10.1080/24745332.2023.2214070 | Excluded | Lack of relation between the title and the abstract of the article with the subject under study. |
|  | Mickleborough TD, Gotshall RW. Dietary salt intake as a potential modifier of airway responsiveness in bronchial asthma. Journal of Alternative and Complementary Medicine. 2004;10(4):633-42.https://doi.org/10.1089/1075553041829398 | Excluded | Lack of relation between the title and the abstract of the article with the subject under study. |
|  | Mickleborough TD, Ionescu AA, Rundell KW. Omega-3 fatty acids and airway hyperresponsiveness in asthma. Journal of Alternative and Complementary Medicine. 2004;10(6):1067-75.https://doi.org/10.1089/acm.2004.10.1067 | Excluded | Lack of relation between the title and the abstract of the article with the subject under study. |
|  | Midulla F, Lombardi E, Rottier B, Lindblad A, Grigg J, Bohlin K, et al. Paediatrics in Barcelona. European Respiratory Journal. 2014;44(2):457-74.https://doi.org/10.1183/09031936.00046414 | Excluded | Lack of relation between the title and the abstract of the article with the subject under study. |
|  | Miethe S, Karsonova A, Karaulov A, Renz H. Obesity and asthma. Journal of Allergy and Clinical Immunology. 2020;146(4):685-93.https://doi.org/10.1016/j.jaci.2020.08.011 | Excluded | Lack of relation between the title and the abstract of the article with the subject under study. |
|  | Miller PSJ, Hill H, Andersson FL. Nocturia Work Productivity and Activity Impairment Compared with Other Common Chronic Diseases. PharmacoEconomics. 2016;34(12):1277-97.https://doi.org/10.1007/s40273-016-0441-9 | Excluded | Lack of relation between the title and the abstract of the article with the subject under study. |
|  | Mills JF, Monaghan NP, Nguyen SA, O'Rourke AK, Halstead LA, Meyer TA. Adult Laryngomalacia: A Scoping Review. Otolaryngology - Head and Neck Surgery (United States). 2024;170(4):1020-31.https://doi.org/10.1002/ohn.639 | Excluded | Lack of relation between the title and the abstract of the article with the subject under study. |
|  | Mirra V, Montella S, Santamaria F. Pediatric severe asthma: A case series report and perspectives on anti-IgE treatment. BMC Pediatrics. 2018;18(1).https://doi.org/10.1186/s12887-018-1019-9 | Excluded | Lack of relation between the title and the abstract of the article with the subject under study. |
|  | Mitchinson L, Chu C, Bruun A, Sisk AR, Armstrong M, Vindrola-Padros C, et al. How best to capture the impact of complementary therapies in palliative care: A systematic review to identify and assess the appropriateness and validity of multi-domain tools. Palliative Medicine. 2022;36(9):1320-35.https://doi.org/10.1177/02692163221122955 | Excluded | Lack of relation between the title and the abstract of the article with the subject under study. |
|  | Mleczko M, Gerkowicz A, Krasowska D. Chronic Inflammation as the Underlying Mechanism of the Development of Lung Diseases in Psoriasis: A Systematic Review. International journal of molecular sciences. 2022;23(3).https://doi.org/10.3390/ijms23031767 | Excluded | Lack of relation between the title and the abstract of the article with the subject under study. |
|  | Moen MD, Wagstaff AJ. Nebivolol: A review of its use in the management of hypertension and chronic heart failure. Drugs. 2006;66(10):1389-409.https://doi.org/10.2165/00003495-200666100-00007 | Excluded | Lack of relation between the title and the abstract of the article with the subject under study. |
|  | Mohan V, Rathinam C, Yates D, Paungmali A, Boos C. Validity and reliability of outcome measures to assess dysfunctional breathing: a systematic review. BMJ Open Respiratory Research. 2024;11(1).https://doi.org/10.1136/bmjresp-2023-001884 | Excluded | Lack of relation between the title and the abstract of the article with the subject under study. |
|  | Molis WE, Bagniewski S, Weaver AL, Jacobson RM, Juhn YJ. Timeliness of diagnosis of asthma in children and its predictors. Allergy: European Journal of Allergy and Clinical Immunology. 2008;63(11):1529-35.https://doi.org/10.1111/j.1398-9995.2008.01749.x | Excluded | Lack of relation between the title and the abstract of the article with the subject under study. |
|  | Monninkhof EM, van der Valk PD, van der Palen J, van Herwaarden CL, Partidge MR, Walters EH, et al. Self-management education for chronic obstructive pulmonary disease. Cochrane database of systematic reviews (Online). 2003(1):CD002990 | Excluded | Lack of relation between the title and the abstract of the article with the subject under study. |
|  | Moran B, Bryan S, Farrar T, Salud C, Visser G, Decuba R, et al. Diagnostic evaluation of nontraumatic chest pain in athletes. Current Sports Medicine Reports. 2017;16(2):84-94.https://doi.org/10.1249/JSR.0000000000000342 | Excluded | Lack of relation between the title and the abstract of the article with the subject under study. |
|  | Moreno-Macias H, Romieu I. Reply. Journal of Allergy and Clinical Immunology. 2014;134(5):1216.https://doi.org/10.1016/j.jaci.2014.08.033 | Excluded | Lack of relation between the title and the abstract of the article with the subject under study. |
|  | Morgan CT, Maloney JD, Decamp MM, McCarthy DP. A narrative review of primary spontaneous pneumomediastinum: A poorly understood and resource-intensive problem. Journal of Thoracic Disease. 2021;13(6):3721-30.https://doi.org/10.21037/jtd-21-193 | Excluded | Lack of relation between the title and the abstract of the article with the subject under study. |
|  | Morgan IG, Wu PC, Ostrin LA, Tideman JWL, Yam JC, Lan W, et al. IMI risk factors for myopia. Investigative Ophthalmology and Visual Science. 2021;62(5).https://doi.org/10.1167/iovs.62.5.3 | Excluded | Lack of relation between the title and the abstract of the article with the subject under study. |
|  | Morris MJ, Christopher KL. Diagnostic criteria for the classification of vocal cord dysfunction. Chest. 2010;138(5):1213-23.https://doi.org/10.1378/chest.09-2944 | Excluded | Lack of relation between the title and the abstract of the article with the subject under study. |
|  | Morrison D, Mair FS, Chaudhuri R, McGee-Lennon M, Thomas M, Thomson NC, et al. Details of development of the resource for adults with asthma in the RAISIN (randomized trial of an asthma internet self-management intervention) study. BMC Medical Informatics and Decision Making. 2015;15(1).https://doi.org/10.1186/s12911-015-0177-z | Excluded | Lack of relation between the title and the abstract of the article with the subject under study. |
|  | Morton AR, Fitch KD. Australian Association for Exercise and Sports Science position statement on exercise and asthma. Journal of Science and Medicine in Sport. 2011;14(4):312-6.https://doi.org/10.1016/j.jsams.2011.02.009 | Excluded | Lack of relation between the title and the abstract of the article with the subject under study. |
|  | Mphahlele R, Masekela R. ADOLESCENT ASTHMA IN SOUTH AFRICA: A CURRENT OVERVIEW OF RISK FACTORS, BARRIERS TO CONTROL AND RESEARCH PRIORITIES. Current Allergy and Clinical Immunology. 2024;37(3):122-5.https://doi.org/10.10520/ejc-caci-v37-n3-a2 | Excluded | Lack of relation between the title and the abstract of the article with the subject under study. |
|  | Mueller W, Milner J, Loh M, Vardoulakis S, Wilkinson P. Exposure to urban greenspace and pathways to respiratory health: An exploratory systematic review. Science of the Total Environment. 2022;829.https://doi.org/10.1016/j.scitotenv.2022.154447 | Excluded | Lack of relation between the title and the abstract of the article with the subject under study. |
|  | Mulholland Y, Nicokavoura E, Broom J, Rolland C. Very-low-energy diets and morbidity: A systematic review of longer-term evidence. British Journal of Nutrition. 2012;108(5):832-51.https://doi.org/10.1017/S0007114512001924 | Excluded | Lack of relation between the title and the abstract of the article with the subject under study. |
|  | Muller A, Rochoy M. Diving and asthma: Literature review. Revue de Pneumologie Clinique. 2018;74(6):416-26.https://doi.org/10.1016/j.pneumo.2018.10.002 | Excluded | Lack of relation between the title and the abstract of the article with the subject under study. |
|  | Munblit D, Greenhawt M, Brough HA, Pushkareva A, Karimova D, Demidova A, et al. Allergic diseases and immunodeficiencies in children, lessons learnt from COVID-19 pandemic by 2022: A statement from the EAACI-section on pediatrics. Pediatric Allergy and Immunology. 2022;33(10).https://doi.org/10.1111/pai.13851 | Excluded | Lack of relation between the title and the abstract of the article with the subject under study. |
|  | Murata T, Fujiyama Y, Yamaga T, Miyazaki H. Breath malodor in an asthmatic patient caused by side-effects of medication: A case report and review of the literature. Oral Diseases. 2003;9(5):273-6.https://doi.org/10.1034/j.1601-0825.2003.02874.x | Excluded | Lack of relation between the title and the abstract of the article with the subject under study. |
|  | Murphy LA, Harrington P, Taylor SJC, Teljeur C, Smith SM, Pinnock H, et al. Clinical-effectiveness of self-management interventions in chronic obstructive pulmonary disease: An overview of reviews. Chronic Respiratory Disease. 2017;14(3):276-88.https://doi.org/10.1177/1479972316687208 | Excluded | Lack of relation between the title and the abstract of the article with the subject under study. |
|  | Murray CJL, Abraham J, Ali MK, Alvarado M, Atkinson C, Baddour LM, et al. The State of US health, 1990-2010: Burden of diseases, injuries, and risk factors. JAMA. 2013;310(6):591-608.https://doi.org/10.1001/jama.2013.13805 | Excluded | Lack of relation between the title and the abstract of the article with the subject under study. |
|  | Murray NG, Low BJ, Hollis C, Cross AW, Davis SM. Coordinated school health programs and academic achievement: A systematic review of the literature. Journal of School Health. 2007;77(9):589-600.https://doi.org/10.1111/j.1746-1561.2007.00238.x | Excluded | Lack of relation between the title and the abstract of the article with the subject under study. |
|  | Murtas R, Tunesi S, Russo AG. Personal protection strategies for mitigating the effects of air pollution: A narrative literature review. Epidemiologia e Prevenzione. 2024;48(6):419-28.https://doi.org/10.19191/EP24.6.A756.126 | Excluded | Lack of relation between the title and the abstract of the article with the subject under study. |
|  | Musich SA, Burton WN, Edington DW. Costs and benefits of prevention and disease management. Disease Management and Health Outcomes. 1999;5(3):153-66.https://doi.org/10.2165/00115677-199905030-00004 | Excluded | Lack of relation between the title and the abstract of the article with the subject under study. |
|  | Myer AB, File TM, Kellermier HC, Myers JP. Cryptococcal Laryngitis Report of a Case and Comprehensive Review of the Literature. Infectious Diseases in Clinical Practice. 2023;31(1).https://doi.org/10.1097/IPC.0000000000001183 | Excluded | Lack of relation between the title and the abstract of the article with the subject under study. |
|  | Mygind L, Kurtzhals M, Nowell C, Melby PS, Stevenson MP, Nieuwenhuijsen M, et al. Landscapes of becoming social: A systematic review of evidence for associations and pathways between interactions with nature and socioemotional development in children. Environment International. 2021;146.https://doi.org/10.1016/j.envint.2020.106238 | Excluded | Lack of relation between the title and the abstract of the article with the subject under study. |
|  | Nabavi SF, Habtemariam S, Daglia M, Sureda A, Sobarzo-Sánchez E, Selamoglu Z, et al. Melatonin and respiratory diseases: A review. Current Topics in Medicinal Chemistry. 2017;17(7).https://doi.org/10.2174/1568026616666160824120338 | Excluded | Lack of relation between the title and the abstract of the article with the subject under study. |
|  | Nadeem A, Siddiqui N, Alharbi NO, Alharbi MM. Airway and systemic oxidant-antioxidant dysregulation in asthma: A possible scenario of oxidants spill over from lung into blood. Pulmonary Pharmacology and Therapeutics. 2014;29(1):31-40.https://doi.org/10.1016/j.pupt.2014.06.001 | Excluded | Lack of relation between the title and the abstract of the article with the subject under study. |
|  | Naim A, Hajjij A, Abbad F, Rami A, Essaadi M. Rare location of head and neck adenoid cystic carcinoma. Pan African Medical Journal. 2019;34.https://doi.org/10.11604/pamj.2019.34.33.19245 | Excluded | Lack of relation between the title and the abstract of the article with the subject under study. |
|  | Nair JJ, van Staden J. Anti-inflammatory Principles of the Plant Family Amaryllidaceae. Planta medica. 2024;90(12):900-37.https://doi.org/10.1055/a-2369-8104 | Excluded | Lack of relation between the title and the abstract of the article with the subject under study. |
|  | Nanda A. Physician wellness in allergy and immunology: Personal resiliency. Annals of Allergy, Asthma and Immunology. 2021;126(3):228-34.https://doi.org/10.1016/j.anai.2020.10.018 | Excluded | Lack of relation between the title and the abstract of the article with the subject under study. |
|  | Narayan P, Yunus A, Morgan JA, Ascione R. Severe tracheal compression as a late complication of plombage. Asian Cardiovascular and Thoracic Annals. 2005;13(1):74-6.https://doi.org/10.1177/021849230501300118 | Excluded | Lack of relation between the title and the abstract of the article with the subject under study. |
|  | Narayanan D, Adebiyi A, Jaggar JH. Inositol trisphosphate receptors in smooth muscle cells. American Journal of Physiology - Heart and Circulatory Physiology. 2012;302(11):H2190-H210.https://doi.org/10.1152/ajpheart.01146.2011 | Excluded | Lack of relation between the title and the abstract of the article with the subject under study. |
|  | Natale C, D'Journo XB, Duconseil P, Thomas PA. Recurrent spontaneous pneumomediastinum in an adult. European Journal of Cardio-thoracic Surgery. 2012;41(5):1199-201.https://doi.org/10.1093/ejcts/ezr135 | Excluded | Lack of relation between the title and the abstract of the article with the subject under study. |
|  | Navalpakam A, Thanaputkaiporn N, Poowuttikul P. Anaphylaxis: Long-term management and resources. Allergy and Asthma Proceedings. 2023;44(1):35-44.https://doi.org/10.2500/aap.2023.44.220089 | Excluded | Lack of relation between the title and the abstract of the article with the subject under study. |
|  | Nayak AP, Penn RB. The proton-sensing receptor ovarian cancer G-protein coupled receptor 1 (OGR1) in airway physiology and disease. Current Opinion in Pharmacology. 2020;51:1-10.https://doi.org/10.1016/j.coph.2020.03.004 | Excluded | Lack of relation between the title and the abstract of the article with the subject under study. |
|  | Ndongo Sonfack DJ, Bojanowski MW, Tarabay B, Gennari A, Shédid D, Yuh SJ. Vertebral artery stenosis from osteophyte: A systematic review and case series. Neurochirurgie. 2024;70(3).https://doi.org/10.1016/j.neuchi.2023.101525 | Excluded | Lack of relation between the title and the abstract of the article with the subject under study. |
|  | Negewo NA, McDonald VM, Gibson PG. Comorbidity in chronic obstructive pulmonary disease. Respiratory Investigation. 2015;53(6):249-58.https://doi.org/10.1016/j.resinv.2015.02.004 | Excluded | Lack of relation between the title and the abstract of the article with the subject under study. |
|  | Negro JM, Miralles JC, Ortiz JL, Funes E, García A. Biosynthesis inhibitors for leukotrienes in bronchial asthma. Allergologia et Immunopathologia. 1997;25(4):209-16 | Excluded | Lack of relation between the title and the abstract of the article with the subject under study. |
|  | Nellessen A, Hernandes NA, Pitta F. Physiotherapy and rehabilitative interventions in patients with chronic respiratory diseases: exercise and non-exercise treatment. Panminerva Medica 2013 Jun;55(2):197-209. 2013 | Excluded | Lack of relation between the title and the abstract of the article with the subject under study. |
|  | Neville RG, Bryce FP, Robertson FM, Crombie IK, Clark RA. Diagnosis and treatment of asthma in children: Usefulness of a review of medical records. British Journal of General Practice. 1992;42(365):501-3 | Excluded | Lack of relation between the title and the abstract of the article with the subject under study. |
|  | Nevitt SJ, Tudur Smith C, Weston J, Marson AG. Lamotrigine versus carbamazepine monotherapy for epilepsy: An individual participant data review. Cochrane Database of Systematic Reviews. 2018;2018(6).https://doi.org/10.1002/14651858.CD001031.pub4 | Excluded | Lack of relation between the title and the abstract of the article with the subject under study. |
|  | Neyra AL, Santiago VS, Asensi JRV. Practical issues in the treatment of childhood asthma. An evidence based approach. Acta Pediatrica Espanola. 2014;72(8):134-41 | Excluded | Lack of relation between the title and the abstract of the article with the subject under study. |
|  | Niazi SK. Advice to the FDA to Improve Its Proposed Guidelines to Rationalize Clinical Trials by Restricting Placebo Control, Preventing Low-Powered Studies, and Disallowing Studies Where Bioavailability Is Not Proven. Pharmaceuticals. 2024;17(11).https://doi.org/10.3390/ph17111424 | Excluded | Lack of relation between the title and the abstract of the article with the subject under study. |
|  | Nichols DE, Grob CS. Is LSD toxic? Forensic Science International. 2018;284:141-5.https://doi.org/10.1016/j.forsciint.2018.01.006 | Excluded | Lack of relation between the title and the abstract of the article with the subject under study. |
|  | Nici L, Aaron SD, Alexander PE, Au DH, Boyd CM, Charbek E, et al. Pharmacologic Management of Chronic Obstructive Pulmonary Disease An Official American Thoracic Society Clinical Practice Guideline. American Journal of Respiratory and Critical Care Medicine. 2020;201(9):E56-E69.https://doi.org/10.1164/RCCM.202003-0625ST | Excluded | Lack of relation between the title and the abstract of the article with the subject under study. |
|  | Nicolie B, Bernier B, Drouet M. Maize allergy. Revue Francaise d'Allergologie. 2009;49(7):547-53.https://doi.org/10.1016/j.reval.2009.07.001 | Excluded | Lack of relation between the title and the abstract of the article with the subject under study. |
|  | Nielsen M, Bårnes CB, Ulrik CS. Clinical characteristics of the asthma–COPD overlap syndrome – A systematic review. International Journal of COPD. 2015;10(1):1443-54.https://doi.org/10.2147/COPD.S85363 | Excluded | Lack of relation between the title and the abstract of the article with the subject under study. |
|  | Nielsen MB, Nordestgaard BG, Benn M, colak Y. Plasma adiponectin and risk of asthma: observational analysis, genetic Mendelian randomisation and meta-analysis. Thorax. 2022;77(11):1070-7.https://doi.org/10.1136/thoraxjnl-2021-217675 | Excluded | Lack of relation between the title and the abstract of the article with the subject under study. |
|  | Niu J, Li B, Zhang Q, Chen G, Papadaki A. Exploring the traditional Chinese diet and its association with health status—a systematic review. Nutrition Reviews. 2025;83(2):e237-e56.https://doi.org/10.1093/nutrit/nuae013 | Excluded | Lack of relation between the title and the abstract of the article with the subject under study. |
|  | Njoku CM, Alqahtani JS, Wimmer BC, Peterson GM, Kinsman L, Hurst JR, et al. Risk factors and associated outcomes of hospital readmission in COPD: A systematic review. Respiratory Medicine. 2020;173.https://doi.org/10.1016/j.rmed.2020.105988 | Excluded | Lack of relation between the title and the abstract of the article with the subject under study. |
|  | Njoku CM, Hurst JR, Kinsman L, Balogun S, Obamiro K. COPD in Africa: Risk factors, hospitalisation, readmission and associated outcomes - A systematic review and meta-analysis. Thorax. 2023;78(6):596-605.https://doi.org/10.1136/thorax-2022-218675 | Excluded | Lack of relation between the title and the abstract of the article with the subject under study. |
|  | Noal RB, Menezes AMB, Macedo SEC, Dumith SC. Childhood body mass index and risk of asthma in adolescence: A systematic review. Obesity Reviews. 2011;12(2):93-104.https://doi.org/10.1111/j.1467-789X.2010.00741.x | Excluded | Lack of relation between the title and the abstract of the article with the subject under study. |
|  | Noori F, Abduljawad S, Suffin DM, Riar S, Pi J, Bennett-Venner A, et al. Mounier-Kuhn syndrome: A case report. Lung. 2010;188(4):353-4.https://doi.org/10.1007/s00408-009-9220-0 | Excluded | Lack of relation between the title and the abstract of the article with the subject under study. |
|  | Noormal AS. Prevalence of major non-communicable diseases and their associated risk factors in Afghanistan: a systematic review and meta-analysis. Therapeutic Advances in Chronic Disease. 2024;15.https://doi.org/10.1177/20406223241229850 | Excluded | Lack of relation between the title and the abstract of the article with the subject under study. |
|  | Norouzi Kamareh MH. The relationship between exercise and immune system. Iranian Journal of Allergy, Asthma and Immunology. 2013;12(1):S67 | Excluded | Lack of relation between the title and the abstract of the article with the subject under study. |
|  | Norouzi Kamareh MH. The relationship between exercise and asthma. Iranian Journal of Allergy, Asthma and Immunology. 2013;12(1):S98 | Excluded | Lack of relation between the title and the abstract of the article with the subject under study. |
|  | Nowińska B, Piotrowski J, Dorobisz K. Samter’s Triad: pathogenesis, clinical picture, diagnosis, comparison of biological and surgical treatment and the role of aspirin desensitisation. Family Medicine and Primary Care Review. 2022;24(4):370-4.https://doi.org/10.5114/fmpcr.2022.120862 | Excluded | Lack of relation between the title and the abstract of the article with the subject under study. |
|  | Nowobilski R, Plaszewski M, Wloch T, Mika P, Gajewski P, Brozek JL. Physiotherapy in asthma - Seeking consensus. Journal of Asthma. 2013;50(6):681-6.https://doi.org/10.3109/02770903.2013.790421 | Excluded | Lack of relation between the title and the abstract of the article with the subject under study. |
|  | Nowobilski R, Plaszewski M, Wloch T, Mika P, Gajewski P, Brożek JL. Physiotherapy in asthma--seeking consensus. The Journal of asthma : official journal of the Association for the Care of Asthma. 2013;50(6):681-6.https://doi.org/10.3109/02770903.2013.790421 | Excluded | Lack of relation between the title and the abstract of the article with the subject under study. |
|  | Nur ABSS, Chua JYX, Shorey S. Effectiveness of community-based family-focused interventions on family functioning among families of children with chronic health conditions: A systematic review and meta-analysis. Family Process. 2023;62(4):1408-22.https://doi.org/10.1111/famp.12930 | Excluded | Lack of relation between the title and the abstract of the article with the subject under study. |
|  | Nyberg ST, Batty GD, Pentti J, Virtanen M, Alfredsson L, Fransson EI, et al. Obesity and loss of disease-free years owing to major non-communicable diseases: a multicohort study. The Lancet Public health. 2018;3(10):E490-E7.https://doi.org/10.1016/s2468-2667(18)30139-7 | Excluded | Lack of relation between the title and the abstract of the article with the subject under study. |
|  | Nyberg ST, Singh-Manoux A, Pentti J, Madsen IEH, Sabia S, Alfredsson L, et al. Association of Healthy Lifestyle With Years Lived Without Major Chronic Diseases. Jama Internal Medicine. 2020;180(5):760-8.https://doi.org/10.1001/jamainternmed.2020.0618 | Excluded | Lack of relation between the title and the abstract of the article with the subject under study. |
|  | Nyssen OP, Taylor SJC, Wong G, Steed E, Bourke L, Lord J, et al. Does therapeutic writing help people with long-term conditions? Systematic review, realist synthesis and economic considerations. Health Technology Assessment. 2016;20(27):1-367.https://doi.org/10.3310/hta20270 | Excluded | Lack of relation between the title and the abstract of the article with the subject under study. |
|  | O'Brien DT, Farrell C, Welsh BC. Looking Through Broken Windows: The Impact of Neighborhood Disorder on Aggression and Fear of Crime Is an Artifact of Research Design. In: Petersilia J, Sampson RJ, editors. Annual Review of Criminology, Vol 2. Annual Review of Criminology. 22019. p. 53-71. | Excluded | Lack of relation between the title and the abstract of the article with the subject under study. |
|  | O'Byrne PM, Barnes NC. Summary: The future promise of mediator inhibitors. European Respiratory Review. 1997;7(46):274-7 | Excluded | Lack of relation between the title and the abstract of the article with the subject under study. |
|  | O'Byrne PM, Barnes NC, editors. Summary: The future promise of mediator inhibitors. European Respiratory Review; 1997. | Excluded | Lack of relation between the title and the abstract of the article with the subject under study. |
|  | Oikonomidi T, Vivot A, Tran VT, Riveros C, Robin E, Ravaud P. A Methodologic Systematic Review of Mobile Health Behavior Change Randomized Trials. American Journal of Preventive Medicine. 2019;57(6):836-43.https://doi.org/10.1016/j.amepre.2019.07.008 | Excluded | Lack of relation between the title and the abstract of the article with the subject under study. |
|  | Okada M. Big data and real-world data-based medicine in the management of hypertension. Hypertension Research. 2021;44(2):147-53.https://doi.org/10.1038/s41440-020-00580-3 | Excluded | Lack of relation between the title and the abstract of the article with the subject under study. |
|  | Okoniewski W, Lu KD, Forno E. Weight loss for children and adults with obesity and asthma a systematic review of randomized controlled trials. Annals of the American Thoracic Society. 2019;16(5):613-25.https://doi.org/10.1513/AnnalsATS.201810-651SR | Excluded | Lack of relation between the title and the abstract of the article with the subject under study. |
|  | Olin JT, Hull JH. The Future of Exertional Respiratory Problems: What Do We Know About the Total Airway Approach and What Do We Need to Know? Immunology and Allergy Clinics of North America. 2018;38(2):333-9.https://doi.org/10.1016/j.iac.2018.01.013 | Excluded | Lack of relation between the title and the abstract of the article with the subject under study. |
|  | Olympia RP, Brady J. Emergency preparedness in high school-based athletics: A review of the literature and recommendations for sport health professionals. Physician and Sportsmedicine. 2013;41(2):15-25.https://doi.org/10.3810/psm.2013.05.2008 | Excluded | Lack of relation between the title and the abstract of the article with the subject under study. |
|  | Ombada M, Belay NAOD, Alamin AMAL, Mohamed ALAA, Hassan R, Mohamed DUAA, et al. UNRAVELING THE UNKNOWN: EXPLORING THE EFFECTS OF E-CIGARETTES. 2024. p. A6240-A1.10.1016/j.chest.2024.06.3696 | Excluded | Lack of relation between the title and the abstract of the article with the subject under study. |
|  | Omole OB, Pretorius D, von Pressentin KB. An approach to persons who are not willing to engage in behavioural change. South African Family Practice. 2024;66.https://doi.org/10.4102/safp.v66i1.5874 | Excluded | Lack of relation between the title and the abstract of the article with the subject under study. |
|  | O'Neill K, Parrott H, Mc Grath R, Neilly C, Bradley JM. A systematic review to assess the components of clinical decision support systems (CDSS)in chronic respiratory disease (CRD) and their relevance to physiotherapy management. European Respiratory Journal. 2021;58(SUPPL 65).https://doi.org/10.1183/13993003.congress-2021.PA3449 | Excluded | Lack of relation between the title and the abstract of the article with the subject under study. |
|  | Opolski M, Wilson I. Asthma and depression: A pragmatic review of the literature and recommendations for future research. Clinical Practice and Epidemiology in Mental Health. 2005;1.https://doi.org/10.1186/1745-0179-1-18 | Excluded | Lack of relation between the title and the abstract of the article with the subject under study. |
|  | Orlowski A, Ettinger J, Bottle A, Snow S, Ashton R, Quint JK. Modifiable risk factors that may be addressed in routine care to prevent progression to and extension of multimorbidity in people with COPD: a systematic literature review. BMJ Open Respir Res. 2024;11(1).https://doi.org/10.1136/bmjresp-2023-002272 | Excluded | Lack of relation between the title and the abstract of the article with the subject under study. |
|  | Osadnik CR, Singh S. Pulmonary rehabilitation for obstructive lung disease. Respirology. 2019;24(9):871-8.https://doi.org/10.1111/resp.13569 | Excluded | Lack of relation between the title and the abstract of the article with the subject under study. |
|  | Ostrom NK. Tolerability of Short-Term, High-Dose Formoterol in Healthy Volunteers and Patients with Asthma. Clinical Therapeutics. 2003;25(11):2635-46.https://doi.org/10.1016/S0149-2918(03)80325-9 | Excluded | Lack of relation between the title and the abstract of the article with the subject under study. |
|  | Oudjedi A, Said Aissa K. Associations between obesity, asthma and physical activity in children and adolescents. Apunts Sports Medicine. 2020;55(205):39-48.https://doi.org/10.1016/j.apunsm.2020.02.003 | Excluded | Lack of relation between the title and the abstract of the article with the subject under study. |
|  | Owess MM, Owda AY, Owda M, Massad S. Supervised Machine Learning-Based Models for Predicting Raised Blood Sugar. International Journal of Environmental Research and Public Health. 2024;21(7).https://doi.org/10.3390/ijerph21070840 | Excluded | Lack of relation between the title and the abstract of the article with the subject under study. |
|  | Özgüney I. An alternative topical treatment of osteoarthritis of the knee with cutaneous diclofenac solution. Expert Opinion on Pharmacotherapy. 2008;9(10):1805-16.https://doi.org/10.1517/14656566.9.10.1805 | Excluded | Lack of relation between the title and the abstract of the article with the subject under study. |
|  | Pacilio RM, Livingston RK, Gordon MR. The Use of Electroconvulsive Therapy in Eating Disorders: A Systematic Literature Review and Case Report. Journal of ECT. 2019;35(4):272-8.https://doi.org/10.1097/YCT.0000000000000599 | Excluded | Lack of relation between the title and the abstract of the article with the subject under study. |
|  | Packard KA, Wurdeman RL, Arouni AJ. ACE inhibitor-induced bronchial reactivity in patients with respiratory dysfunction. The Annals of pharmacotherapy. 2002;36(6):1058-67.https://doi.org/10.1345/aph.1A332 | Excluded | Lack of relation between the title and the abstract of the article with the subject under study. |
|  | Packard KA, Wurdeman RL, Arouni AJ. ACE inhibitor-induced bronchial reactivity in patients with respiratory dysfunction. Annals of Pharmacotherapy. 2002;36(6):1058-67.https://doi.org/10.1345/aph.1A332 | Excluded | Lack of relation between the title and the abstract of the article with the subject under study. |
|  | Pada S, Lye DC, Leo YS, Barkham T. Utility of 16S ribosomal DNA sequencing in the diagnosis of Staphylococcus lugdunensis native valve infective endocarditis: case report and literature review. International Journal of Infectious Diseases. 2009;13(6):e511-e3.https://doi.org/10.1016/j.ijid.2009.02.019 | Excluded | Lack of relation between the title and the abstract of the article with the subject under study. |
|  | Padovano I, Pazzola G, Pipitone N, Cimino L, Salvarani C. Anterior ischaemic optic neuropathy in eosinophilic granulomatosis with polyangiitis (Churg-Strauss syndrome): A case report and review of the literature. Clinical and Experimental Rheumatology. 2014;32(SUPPL.82):S62-S5 | Excluded | Lack of relation between the title and the abstract of the article with the subject under study. |
|  | Page K, Brownie S, Wohlmuth H. Natural management options for menopause. Integrative Medicine. 2005;4(1):20-8 | Excluded | Lack of relation between the title and the abstract of the article with the subject under study. |
|  | Pai M, Key NS, Skinner M, Curtis R, Feinstein M, Kessler C, et al. NHF-McMaster Guideline on Care Models for Haemophilia Management. Haemophilia. 2016;22:6-16.https://doi.org/10.1111/hae.13008 | Excluded | Lack of relation between the title and the abstract of the article with the subject under study. |
|  | Panasiti I, Costa S, Caminiti L, Crisafulli G, Pajno GB, Pellegrino S, et al. Association of wheat allergy and coeliac disease through pediatric and adult age: A review of literature. Current Nutrition and Food Science. 2021;17(6):553-7.https://doi.org/10.2174/1573401316999201105145739 | Excluded | Lack of relation between the title and the abstract of the article with the subject under study. |
|  | Pao M, Bosk A. Anxiety in medically ill children/adolescents. Depression and Anxiety. 2011;28(1):40-9.https://doi.org/10.1002/da.20727 | Excluded | Lack of relation between the title and the abstract of the article with the subject under study. |
|  | Papamichael MM, Erbas B, Tsoukalas D, Itsiopoulos C, Katsardis C. Impact of Pediatric Obesity on Lung Physiology. Advances in Health and Disease. 332021. p. 1-41. | Excluded | Lack of relation between the title and the abstract of the article with the subject under study. |
|  | Papoutsakis C, Priftis KN, Drakouli M, Prifti S, Konstantaki E, Chondronikola M, et al. Childhood Overweight/Obesity and Asthma: Is There a Link? A Systematic Review of Recent Epidemiologic Evidence. Journal of the Academy of Nutrition and Dietetics. 2013;113(1):77-105.https://doi.org/10.1016/j.jand.2012.08.025 | Excluded | Lack of relation between the title and the abstract of the article with the subject under study. |
|  | Parama D, Boruah M, Yachna K, Rana V, Banik K, Harsha C, et al. Diosgenin, a steroidal saponin, and its analogs: Effective therapies against different chronic diseases. Life Sciences. 2020;260.https://doi.org/10.1016/j.lfs.2020.118182 | Excluded | Lack of relation between the title and the abstract of the article with the subject under study. |
|  | Pardo LC, Gonzalez-Estrada A, Lang DM. Diagnostic utility of challenge procedures for physical urticaria/angioedema syndromes: A systematic review. Journal of Allergy and Clinical Immunology. 2016;137(2):AB242 | Excluded | Lack of relation between the title and the abstract of the article with the subject under study. |
|  | Parekh AB. House dust mite allergens, store-operated Ca2+ channels and asthma. Journal of Physiology. 2024;602(22):6021-38.https://doi.org/10.1113/JP284931 | Excluded | Lack of relation between the title and the abstract of the article with the subject under study. |
|  | Parikh K, Keller S, Ralston S. Inpatient quality improvement interventions for asthma: a meta-analysis. Pediatrics 2018 May;141(5):e20173334. 2018 | Excluded | Lack of relation between the title and the abstract of the article with the subject under study. |
|  | Parisod H, Pakarinen A, Kauhanen L, Aromaa M, Leppanen V, Liukkonen TN, et al. Promoting Children's Health with Digital Games: A Review of Reviews. Games for Health Journal. 2014;3(3):145-+.https://doi.org/10.1089/g4h.2013.0086 | Excluded | Lack of relation between the title and the abstract of the article with the subject under study. |
|  | Park JH, Yim BK, Lee JH, Lee S, Kim TH. Risk associated with bee venom therapy: A systematic review and meta-analysis. PLoS ONE. 2015;10(5).https://doi.org/10.1371/journal.pone.0126971 | Excluded | Lack of relation between the title and the abstract of the article with the subject under study. |
|  | Park KB, Chapman T, Aldinger KA, Mirzaa GM, Zeiger J, Beck A, et al. The spectrum of brain malformations and disruptions in twins. American Journal of Medical Genetics, Part A. 2021;185(9):2690-718.https://doi.org/10.1002/ajmg.a.61972 | Excluded | Lack of relation between the title and the abstract of the article with the subject under study. |
|  | Parker CH, Yuan Y, Liu LWC. Linaclotide: A new option for the treatment of irritable bowel syndrome with constipation and chronic idiopathic constipation in adults. Clinical Medicine Insights: Gastroenterology. 2013;6:21-32.https://doi.org/10.4137/CGast.S10550 | Excluded | Lack of relation between the title and the abstract of the article with the subject under study. |
|  | Parkerson J, Ledford D. Mannitol as an indirect bronchoprovocation test for the 21st century. Annals of Allergy, Asthma and Immunology. 2011;106(2):91-6.https://doi.org/10.1016/j.anai.2010.11.010 | Excluded | Lack of relation between the title and the abstract of the article with the subject under study. |
|  | Parnell Prevost C, Gleberzon B, Carleo B, Anderson K, Cark M, Pohlman KA. Manual therapy for the pediatric population: a systematic review. BMC Complementary and Alternative Medicine 2019 Mar 13;19(60):Epub. 2019 | Excluded | Lack of relation between the title and the abstract of the article with the subject under study. |
|  | Parreira VF, Janaudis-Ferreira T, Evans RA, Mathur S, Goldstein RS, Brooks D. Measurement properties of the incremental shuttle walk test: A systematic review. Chest. 2014;145(6):1357-69.https://doi.org/10.1378/chest.13-2071 | Excluded | Lack of relation between the title and the abstract of the article with the subject under study. |
|  | Parreira VF, Vieira DSR, Myrrha MAC, Pessoa IMBS, Lage SM, Britto RR. Optoelectronic plethysmography: A review of the literature. Brazilian Journal of Physical Therapy. 2012;16(6):439-53.https://doi.org/10.1590/S1413-35552012005000061 | Excluded | Lack of relation between the title and the abstract of the article with the subject under study. |
|  | Parry SM, Knight LD, Connolly B, Baldwin C, Puthucheary Z, Morris P, et al. Factors influencing physical activity and rehabilitation in survivors of critical illness: a systematic review of quantitative and qualitative studies. Intensive Care Medicine. 2017;43(4):531-42.https://doi.org/10.1007/s00134-017-4685-4 | Excluded | Lack of relation between the title and the abstract of the article with the subject under study. |
|  | Partonen T. Chronotype and Health Outcomes. Current Sleep Medicine Reports. 2015;1(4):205-11.https://doi.org/10.1007/s40675-015-0022-z | Excluded | Lack of relation between the title and the abstract of the article with the subject under study. |
|  | Passalacqua G, Bousquet PJ, Carlsen K-H, Kemp J, Lockey RF, Niggemann B, et al. ARIA update: I - Systematic review of complementary and alternative medicine for rhinitis and asthma. Journal of Allergy and Clinical Immunology. 2006;117(5):1054-62.https://doi.org/10.1016/j.jaci.2005.12.1308 | Excluded | Lack of relation between the title and the abstract of the article with the subject under study. |
|  | Passalacqua G, Compalati E, Schiappoli M, Senna G. Complementary and alternative medicine for the treatment and diagnosis of asthma and allergic diseases. Monaldi Archives for Chest Disease - Pulmonary Series. 2005;63(1):47-54.https://doi.org/10.4081/monaldi.2005.657 | Excluded | Lack of relation between the title and the abstract of the article with the subject under study. |
|  | Patel KR, Ghosh SK, Matcham J. Lack of dose-response effect of terfenadine on resting bronchomotor tone in patients with asthma. Clinical and experimental allergy : journal of the British Society for Allergy and Clinical Immunology. 1991;21(3):363-6.https://doi.org/10.1111/j.1365-2222.1991.tb01669.x | Excluded | Lack of relation between the title and the abstract of the article with the subject under study. |
|  | Patel NM, Puri A, Sounderajah V, Ferri L, Griffiths E, Low D, et al. Quality of life and symptom assessment in paraesophageal hernias: A systematic literature review of reporting standards. Diseases of the Esophagus. 2021;34(7).https://doi.org/10.1093/dote/doaa134 | Excluded | Lack of relation between the title and the abstract of the article with the subject under study. |
|  | Patel RR, Venediktov R, Schooling T, Wang B. Evidence-Based Systematic Review: Effects of Speech-Language Pathology Treatment for Individuals With Paradoxical Vocal Fold Motion. American Journal of Speech-Language Pathology. 2015;24(3):566-84.https://doi.org/10.1044/2015_ajslp-14-0120 | Excluded | Lack of relation between the title and the abstract of the article with the subject under study. |
|  | Patel RV, Shelling ML, Prodanovich S, Federman DG, Kirsner RS. Psoriasis and vascular disease-risk factors and outcomes: A systematic review of the literature. Journal of General Internal Medicine. 2011;26(9):1036-49.https://doi.org/10.1007/s11606-011-1698-5 | Excluded | Lack of relation between the title and the abstract of the article with the subject under study. |
|  | Patel VH, Thannir S, Dhanani M, Augustine I, Sandeep SL, Mehadi A, et al. Current Limitations and Recent Advances in the Management of Asthma. Disease-a-Month. 2023;69(7).https://doi.org/10.1016/j.disamonth.2022.101483 | Excluded | Lack of relation between the title and the abstract of the article with the subject under study. |
|  | Pathare N, Burgess K, Flynn J, Jones SR. Water-based exercise in individuals with chronic obstructive pulmonary disease: A systematic review. Cardiopulmonary Physical Therapy Journal. 2022;33(1):e6-e7.https://doi.org/10.1097/CPT.0000000000000197 | Excluded | Lack of relation between the title and the abstract of the article with the subject under study. |
|  | Pattarini JM, Blue RS, Alexander DJ. Thermal Regulation of Emergency Oxygen Supplies in Commercial Space Vehicles. Aerospace Medicine and Human Performance. 2018;89(10):918-22.https://doi.org/10.3357/amhp.5126.2018 | Excluded | Lack of relation between the title and the abstract of the article with the subject under study. |
|  | Patterson SW. Addiction to endocrine gland extracts. British Medical Journal. 1935;2(3896):442-5.https://doi.org/10.1136/bmj.2.3896.442 | Excluded | Lack of relation between the title and the abstract of the article with the subject under study. |
|  | Paudyal P, Hine P, Theadom A, Apfelbacher CJ, Jones CJ, Yorke J, et al. Written emotional disclosure for asthma. Cochrane Database of Systematic Reviews. 2014(5).https://doi.org/10.1002/14651858.CD007676.pub2 | Excluded | Lack of relation between the title and the abstract of the article with the subject under study. |
|  | Paudyal P, Jones C, Grindey C, Dawood R, Smith H. Meditation for asthma: Systematic review and meta-analysis. Journal of Asthma. 2018;55(7):771-8.https://doi.org/10.1080/02770903.2017.1365887 | Excluded | Lack of relation between the title and the abstract of the article with the subject under study. |
|  | Pavone F, Pavone M, Falcone A, Anania A, Cavallaro G, Fama M. Asthmatic child and sports: Review of the literature and our experience. Aggiornamento Pediatrico. 1998;1(1):11-8 | Excluded | Lack of relation between the title and the abstract of the article with the subject under study. |
[truncated: 663,536 more chars]
