# Supplementary material for: The effectiveness of physical activity in asthma management: An overview of systematic reviews
Source: PLoS One. 2025 Jul 3;20(7):e0325488. doi: 10.1371/journal.pone.0325488 (PMC12225870; doi:10.1371/journal.pone.0325488)
Supplement: S3 Appendix — (DOCX) [file pone.0325488.s003.docx]

**Quality of Evidence in Included Systematic Reviews with GRADE**

| **Outcome**  **Measures** | | **Systematic Reviews** | **N/n** | **MD/SMD (95%CI)** | **Quality Assessment** | | | | | **Quality of**  **Evidence** |
| --- | --- | --- | --- | --- | --- | --- | --- | --- | --- | --- |
|  |  |  |  |  | **Risk of**  **Bias** | **Inconsistency** | **Indirectness** | **Imprecision** | **Publication**  **Bias** |  |
| **QOL** | AQLQ | **PA** | | | | | | | | |
|  |  | Feng, 2021 | 4(198) | 0.39(0.02, 0.76)* | 0 | 0 | 0 | -1^c^ | 0 | Moderate |
|  |  | Shi, 2022 | 7(590) | −0.03(−0.61, 0.54) | 0 | -1b | 0 | 0 | -1d | Low |
|  |  | Zhu, 2022 | 9(534) | 0.41(0.32,0.51)* | -1a | 0 | 0 | 0 | 0 | moderate |
|  |  | McLoughlin, 2022 | 2(142) | -0.65(-0.95,-0.35)* | 0 | 0 | 0 | -1c | -1e | Low |
|  |  | Jing, 2023 | 5(298) | 1.38(0.26,2.50) | -1a | -1b | 0 | -1c | 0 | Very Low |
|  |  | Osadnik, 2022 | 2(442) | 0.87(-0.13, 1.86) | 0 | -1b | 0 | -1c | -1e | Very Low |
|  |  | Liu Fang, 2021 | 10(794) | 1.38(0.65,2.12)* | -1a | -1b | 0 | 0 | 0 | Low |
|  |  | Yang, 2022 | 4(149) | -0.97(-3.55,1.61)* | -1a | -1b | 0 | -1c | 0 | Very Low |
|  |  | Liu, 2021 | 10(714) | 1.28(0.60,1.95)* | -1a | -1b | 0 | 0 | 0 | Low |
|  |  | **AE** | | | | | | | | |
|  |  | Wu, 2020 | 5(233) | 0.81(0.32, 1.30) * | -1a | -1b | 0 | 0 | 0 | Low |
|  |  | Ma, 2024 | 12(647) | 0.7(0.14,1.26)* | -1a | -1b | 0 | 0 | 0 | Low |
|  |  | Zhu, 2022 | 9(192) | 0.52(0.37,0.67)* | -1a | 0 | 0 | -1c | 0 | low |
|  |  | **Yoga** | | | | | | | | |
|  |  | Anshu, 2023 | 5(645) | 0.26(0.18,0.34)* | -1a | -1b | 0 | 0 | -1e | Very Low |
|  |  | Yang, 2016 | 5(375) | 0.57(0.37,0.77)* | -1a | 0 | 0 | 0 | 0 | Low |
|  |  | **BE** | | | | | | | | |
|  |  | Burgess, 2011 | 5(447) | 0.35(0.00, 0.70) * | 0 | -1b | 0 | 0 | 0 | Moderate |
|  |  | Santino, 2020 | 4(974) | 0.42(0.17, 0.68) * | 0 | -1b | 0 | 0 | 0 | Moderate |
|  |  | Freitas, 2013 | 2(172) | 0.79(0.50,1.08)* | -1a | 0 | 0 | -1c | -1e | Very Low |
|  |  | **IMT** | | | | | | | | |
|  |  | Yi,2021 | 2(70) | 1.21(-2.11,4.52) | 0 | -1b | 0 | -1c | -1e | Very Low |
| **Asthma control** | ACQ | **PA** | | | | | | | | |
|  |  | Feng, 2021 | 5(215) | −0.25(0.51, 0.02)* | 0 | 0 | 0 | -1c | 0 | Moderate |
|  |  | McLoughlin, 2022 | 3(88) | 0.56(0.10,1.01)* | 0 | 0 | 0 | -1c | -1e | Low |
|  |  | Osadnik, 2022 | 2(93) | -0.46(-0.76, -0.17) * | -1a | 0 | 0 | 0 | -1e | Low |
|  |  | **yoga** | | | | | | | | |
|  |  | Anshu, 2023 | 3(166) | 0.16(0.15,0.48) | -1a | 1b | 0 | -1c | -1e | Very Low |
|  |  | **BE** | | | | | | | | |
|  |  | Santino, 2020 | 1(115) | -0.21(-2.92, 2.5) | 0 | 0 | 0 | -1c | -1e | Low |
|  | Asthma symptom  free days | **PA** | | | | | | | | |
|  |  | Eichenberger, 2013 | 2(109) | 8.90(8.18, 9.61) * | -1a | -1b | 0 | -1c | -1e | Very Low |
|  |  | Feng, 2021 | 2(94) | 3.35 (−0.21, 6.90) | 0 | 0 | 0 | -1c | -1e | Low |
|  |  | Shi, 2022 | 4(216) | 0.93(−0.39, 2.25) | 0 | -1b | 0 | -1c | -1d | Very Low |
| **Lung**  **function** | FEV_1_ | **PA** | | | | | | | | |
|  |  | Li, 2022 | 14(1009) | 0.29(0.08, 0.51)* | -1a | -1b | 0 | 0 | -1d | Very Low |
|  |  | Feng, 2021 | 5(242) | 0.10(−0.08, 0.29) | 0 | -1b | 0 | -1c | 0 | Low |
|  |  | Shi, 2022 | 10(796) | 0.24(−0.13 ,0.60) | 0 | -1b | 0 | 0 | -1d | Very Low |
|  |  | Jing,2023 | 6(292) | 3.17(-2.82,9.15)* | -1a | -1b | 0 | -1c | 0 | Very Low |
|  |  | Yin,2019 | 4(311) | -0.31(-0.53,-0.09)* | 0 | 0 | 0 | -1c | -1e | Low |
|  |  | Zhou, 2023 | 12(642) | 2.12(0.70,3.53)* | -1a | 0 | 0 | 0 | 0 | moderate |
|  |  | Ram, 2005 | 5(129) | 0.01(-0.14, 0.16) | -1a | 0 | 0 | -1c | 0 | Low |
|  |  | Eichenberger, 2013 | 11(432) | 0.09(0.00, 0.17) | -1a | 0 | 0 | 0 | 0 | Moderate |
|  |  | **AE** | | | | | | | | |
|  |  | Wu, 2020 | 12(455) | 0.12 (0.05, 0.20)* | -1a | 0 | 0 | 0 | 0 | Moderate |
|  |  | Li, 2022 | 8(657) | 0.41 (0.11, 0.71)* | -1a | -1b | 0 | 0 | -1d | Very Low |
|  |  | Ma, 2024 | 7(319) | 0.4(-0.29,1.09) | -1a | -1b | 0 | -1c | 0 | Very Low |
|  |  | **yoga** | | | | | | | | |
|  |  | Cramer, 2014 | 5(264) | 0.21(0.23, 0.64) | -1a | -1b | 0 | -1c | 0 | Low |
|  |  | Anshu, 2023 | 4(513) | 0.96(0.77,1.14)* | 0 | -1b | 0 | 0 | 0 | moderate |
|  |  | Yang, 2019 | 10(583) | 0.31(-0.08,0.70) | -1a | -1b | 0 | 0 | 0 | Low |
|  |  | **BE** | | | | | | | | |
|  |  | Holloway, 2004(9) | 2(38) | -0.19(-0.70, 0.31) | 0 | 0 | 0 | -1c | -1e | Low |
|  |  | Burgess, 2011(11) | 6(416) | -0.01(-0.15, 0.14) | -1a | 0 | 0 | 0 | 0 | Moderate |
|  |  | Santino, 2020(16) | 2(491) | -0.02(-0.08, 0.04) | 0 | 0 | 0 | 0 | -1e | Moderate |
|  |  | **IMT** | | | | | | | | |
|  |  | Yi,2021 | 8(376) | 0.62(0.41,0.83) | -1a | 0 | 0 | 0 | 0 | moderate |
|  |  | Xiang,2024 | 4(213) | 4.96(2.6,7.32) | -1a | 0 | 0 | -1c | 0 | Low |
|  |  | **Aquatic exercise** | | | | | | | | |
|  |  | Franciele,2024 | 4(157) | 0.2(0.02,0.38) | -1a | 0 | 0 | -1c | 0 | Low |
|  | FVC | **PA** | | | | | | | | |
|  |  | Li, 2022(21) | 14(1009) | 0.40(0.15, 0.66)* | -1a | -1b | 0 | 0 | -1d | Very Low |
|  |  | Feng, 2021(18) | 3(163) | 0.23(0.08, 0.38) * | 0 | 0 | 0 | -1c | -1e | Low |
|  |  | Shi, 2022(23) | 8(717) | -0.02(−0.70, 0.65) | 0 | -1b | 0 | 0 | -1d | Low |
|  |  | Jing, 2023 | 6(292) | 7.62(3.46,11.78) | -1a | 0 | 0 | -1c | 0 | Low |
|  |  | Yin, 2019 | 4(311) | 0.44(-0.61,1.49) | 0 | -1b | 0 | -1c | 0 | Low |
|  |  | Zhou, 2023 | 11(628) | 2.78(1.26,4.31)* | -1a | -1b | 0 | 0 | 0 | Low |
|  |  | Ram, 2005 | 4(93) | 0.09(-0.12, 0.30)* | -1a | 0 | 0 | -1c | 0 | Low |
|  |  | **AE** | | | | | | | | |
|  |  | Wu, 2020 | 10(373) | 0.18(0.09,0.27) * | -1a | 0 | 0 | -1c | 0 | Low |
|  |  | Li, 2022 | 8(657) | 0.44(0.07, 0.81)* | -1a | -1b | 0 | 0 | -1d | Very Low |
|  |  | Ma, 2024 | 6(246) | 0.04(-0.23,0.31) | -1a | 0 | 0 | -1c | 0 | Low |
|  |  | **yoga** | | | | | | | | |
|  |  | Cramer, 2014(15) | 3(164) | 0.09 (0.70, 0.52) | -1a | -1b | 0 | -1c | -1e | Very Low |
|  |  | Anshu, 2023 | 4(381) | 0.35(0.14,0.55)* | 0 | -1b | 0 | -1c | 0 | Low |
|  |  | Yang, 2019 | 6(376) | 0.67(0.2,1.14)* | -1a | -1b | 0 | -1c | 0 | Low |
|  |  | **BE** | | | | | | | | |
|  |  | Holloway, 2004 | 2(38) | 0.16 (-0.41, 0.73) | 0 | 0 | 0 | -1c | -1e | Low |
|  |  | **IMT** | | | | | | | | |
|  |  | Chen, 2022 | 3(83) | 2.09(-0.70, 4.89) | 0 | -1b | 0 | -1c | -1e | Very Low |
|  |  | Lista, 2022 | 5(112) | 0.39(-0.24, 1.41) | 0 | -1b | 0 | -1c | 0 | Low |
|  |  | Wang, 2022 | 3(85) | 0.21(0.03, 0.40) * | -1a | 0 | 0 | -1c | -1e | Very Low |
|  |  | Yi, 2021 | 8(376) | 0.9(0.68,1.11) | 1a | 0 | 0 | 0 | 0 | moderate |
|  |  | Silva, 2013 | 1(18) | 0.54(-0.32, 1.4) | -1a | 0 | 0 | -1c | -1e | Very Low |
|  |  | Ram, 2003 | 2(48) | SMD 1.14(0.51, 1.77) * | -1a | -1b | 0 | -1c | -1e | Very Low |
|  |  | Xiang,2024 | 4(213) | 3.9(1.86,5.93) | 1a | 0 | 0 | -1c | 0 | Very Low |
|  |  | **Aquatic exercise** | | | | | | | | |
|  |  | Franciele,2024 | 3(134) | 0.32(0.08,0.56) | 1a | 0 | 0 | -1c | 1e | Very Low |
|  | PEF | **PA** | | | | | | | | |
|  |  | Jing,2023 | 2(134) | 0.44(-6.71,7.58) | 1a | 0 | 0 | -1c | 1e | Very Low |
|  |  | Yin,2019 | 7(641) | 1.5(0.90,2.10)* | 0 | -1b | 0 | 0 | 0 | moderate |
|  |  | Zhou, 2023 | 7(469) | 1.84(-2.83,6.51)* | 1a | -1b | 0 | -1c | 0 | Low |
|  |  | Feng, 2021 | 2(112) | 0.39(0.21, 0.57) * | 0 | 0 | 0 | -1c | -1e | Low |
|  |  | Li, 2022 | 10(740) | 0.69(0.11, 1.28)* | -1a | -1b | 0 | 0 | -1d | Very Low |
|  |  | Eichenberger, 2013 | 6(283) | 0.45(-0.16, 1.07) | -1a | -1b | 0 | -1c | 0 | Very Low |
|  |  | **AE** | | | | | | | | |
|  |  | Wu, 2020 | 6(113) | 0.66(0.24, 1.09) * | -1a | -1b | 0 | -1c | 0 | Very Low |
|  |  | Li, 2022 | 6(479) | 1.27(0.30, 2.24)* | -1a | -1b | 0 | 0 | -1d | Very Low |
|  |  | Ma,2024 | 4(187) | 1.02(-0.28,2.32) | 1a | -1b | 0 | -1c | 0 | Very Low |
|  |  | **BE** | | | | | | | | |
|  |  | Burgess, 2011 | 2(109) | 16.66(-25.92, 59.23) | 0 | 0 | 0 | -1c | -1e | Low |
|  |  | **IMT** | | | | | | | | |
|  |  | Lista, 2022 | 4(82) | -4.54(-26.74,17.67) | -1a | 0 | 0 | -1c | 0 | Low |
|  |  | Yi,2021 | 5(184) | 0.32(0.01,0.62)* | -1a | 0 | 0 | -1c | 0 | Low |
|  |  | You,2024 | 2(68) | 98.74(71.76,125.19)* | -1a | 0 | 0 | -1c | -1e | Very Low |
|  |  | Xiang,2024 | 2(156) | 56.45(-33.10,145.99)* | -1a | -1b | 0 | -1c | -1e | Very Low |
| Exercise performance | 6 MWD | **PA** | | | | | | | | |
|  |  | Feng, 2021 | 3(94) | 34.09(2.51, 65.66) * | 0 | 0 | 0 | -1c | -1e | Low |
|  |  | Osadnik, 2022 | 5(529) | 79.79(66.47, 93.11) * | -1a | 0 | 0 | 0 | 0 | Moderate |
|  |  | Liu,2021 | 5(256) | 110.65(31.95,189.34)* | -1a | -1b | 0 | -1c | -1e | Very Low |
|  |  | Liu Fang,2021 | 4(166) | 71.59(54.25,88.93)* | -1a | 0 | 0 | -1c | 0 | Low |
|  |  | **IMT** | | | | | | | | |
|  |  | Yi,2021 | 3(200) | 77.13(24.58,129.67)* | -1a | -1b | 0 | -1c | -1e | Very Low |
|  |  | Lista, 2022 | 2(79) | 1.73(-0.61, 4.08) | 0 | -1b | 0 | -1c | -1e | Very Low |
|  | VO_2max_ | **PA** | | | | | | | | |
|  |  | Feng, 2021 | 3(141) | 4.45(3.32, 5.58) * | 0 | 0 | 0 | -1c | -1e | Low |
|  |  | Shi, 2022 | 5(237) | 1.18(0.87, 1.48) * | 0 | 0 | 0 | -1c | 0 | Moderate |
|  |  | Ram, 2005 | 7(175) | 5.43(4.24, 6.61) * | -1a | 0 | 0 | -1c | 0 | Low |
|  |  | Eichenberger, 2013 | 7(238) | 4.06(3.02, 5.10) * | -1a | 0 | 0 | -1c | 0 | Low |
|  |  | Carson, 2013 | 8(267) | 4.92(3.98, 5.87) * | -1a | 0 | 0 | -1c | 0 | Low |
|  |  | Ram,2000 | 5(114) | 5.56(3.94,7.19)* | -1a | 0 | 0 | -1c | 0 | Low |
|  |  | Yang,2022 | 4(149) | 5.86(2.53,9.19)* | -1a | -1b | 0 | -1c | 0 | Very Low |
| respiratory muscle strength | PI_max_ | **IMT** | | | | | | | | |
|  |  | Chen, 2022 | 4(136) | 3.32(1.73, 4.91) * | 0 | -1b | 0 | -1c | 0 | Low |
|  |  | Lista, 2022 | 9(214) | 21.95(15.05, 28.85) * | 0 | -1b | 0 | -1c | 0 | Low |
|  |  | Wang, 2022 | 7(202) | 27.62(6.50, 48.74) * | -1a | -1b | 0 | -1c | 0 | Very Low |
|  |  | You,2024 | 6(183) | 26.21(1.71,50.72)* | -1a | -1b | 0 | -1c | 0 | Very Low |
|  |  | Ram, 2003 | 3(76) | 23.07(15.65, 30.50) * | 0 | 0 | 0 | -1c | -1e | Low |
|  |  | Silva, 2013 | 4(84) | 13.34(4.70,21.98) * | -1a | 0 | 0 | -1c | 0 | Low |
|  |  | Xiang,2024 | 5(227) | 25.36(2.47,48.26)* | -1a | -1b | 0 | -1c | 0 | Very Low |

Notes: N: number of studies; n: number of participants; *p≤0.05; (a) the design of the experiment with a large bias in random, distributive hiding, or blind; (b) the confidence interval overlaps less, the heterogeneity test P is very small, and I2 is larger; (c) the sample size is small, and the confidence interval is wide; (d) funnel graph asymmetry; (e) fewer studies are included, and there may be greater publication bias.
