# Supplementary material for: The effectiveness of physical activity in asthma management: An overview of systematic reviews
Source: PLoS One. 2025 Jul 3;20(7):e0325488. doi: 10.1371/journal.pone.0325488 (PMC12225870; doi:10.1371/journal.pone.0325488)
Supplement: Attachment — Submitted filename: Comments on PA annd Ashma review.docx [file pone.0325488.s006.docx]

**Comments on a manuscript entitled “The effectiveness of physical activity in asthma: an overview of systematic reviews”.**

PONE-D-24-54330

13 December 2024

**Overall comments**

The paper has many unclear statements. In other words, the paper was written pretty loosely. The language used is unclear and often incomprehensible, with numerous grammatical errors and inconsistent formatting e.g. line and between-word spacing. To be honest, I’m quite frustrated reading it. So, I sort of scanned through it rather than reading it in details.

**Specific comments**

**Title**

- Make it more clearly if you mean asthma treatment or prevention or what perspectives?

**Abstract**

- What is the targeted population?

**Intro section**

- P3 L54-58 this sentence is too long to follow. Try breaking down.
- P3-L58-60 this sentence is incomprehensible
- P3-L60 “An” overview, not “a”

**Method section**

- Identify the keywords used to search for papers.
- Any limitation about the language of publication included in the review
- Identify age of the samples
- Identify clearly if the samples were people with current asthma condition or patients at hospital or?
- Identify clearly study design that were included in the review.
- Identify clearly measurement of PA whether the study included both device-based and subjective approaches.
- This study used only 2 reviewers. Usually 3 reviewers are used to screen the papers, in case there are some conflicting ideas when trying to classify the papers. Explain strategies in managing the challenges with 2 reviewers.

**Result section**

- Identify the colours use in figure 3 and what they represented
- Figure 2 showed that there were so many critically low-quality papers, why were they included in the study?

**Conclusions**

- The conclusion didn’t make a good sense of what have been found from the review.
