# Supplementary material for: The effectiveness of physical activity in asthma management: An overview of systematic reviews
Source: PLoS One. 2025 Jul 3;20(7):e0325488. doi: 10.1371/journal.pone.0325488 (PMC12225870; doi:10.1371/journal.pone.0325488)
Supplement: Attachment — Submitted filename: Response to Reviewers.docx [file pone.0325488.s008.docx]

# Response to Reviewer’s Comments

**Dear Editors and Reviewers**

On behalf of all the contributing authors, I would like to express our sincere appreciation of your letter and reviewers’ constructive comments concerning our article entitled “The effectiveness of physical activity in asthma: an overview of systematic review”(Manuscript ID PONE-D-24-54330). Those comments are all valuable and very helpful for revising and improving our paper, as well as the important guiding significance to our researches. In the following, we have provided detailed responses to each of the reviewers' comments. Revised portion are marked in blue in the paper. Additionally, all relevant data are within the paper and its Supporting Information files. In this response letter, the reviewers' comments are presented in italics, and our corresponding changes and additions to the manuscript are highlighted in blue text. If we missed any one of the comments, please let us know. This document includes our responses to your comments, and the revised portion are presented in our main document. We have tried our best to make all the revisions clear, and we hope that the revised manuscript meets the requirements for publication.

Thanks again for your consideration of publishing our manuscript in your journal. We are looking forward to hearing from you soon.

Best wishes,

Sincerely,

Zhenzhen Feng

**Contents**

**[Reviewer #1	4](#_Toc20471)**

**[Reviewer #2	8](#_Toc16781)**

**[Reviewer #3	10](#_Toc20874)**

# Reviewer #1

**Comment 1:** *Overall, the language used is unclear and often incomprehensible, with numerous grammatical errors and inconsistent formatting e.g. line and between-word spacing.*

**Response 1:** Thanks for the suggestion. We have collaborated with a certified professional editing service to conduct rigorous proofreading and substantive revisions during this round of manuscript preparation. The text has been systematically refined to ensure grammatical accuracy, improve readability, and align with academic writing standards. A copy of the language-editing certificate issued by the service provider has been uploaded to the Supplementary Materials section.

**Comment 2:** *Title: Make it more clearly if you mean asthma treatment or prevention or what perspectives?*

**Response 2:** Thank you for your valuable feedback. We appreciate your suggestion to clarify the scope of our review. As recommended, we have revised the title to "The effectiveness of physical activity in asthma management: An overview of systematic reviews" to better reflect the comprehensive nature of our analysis, which examines multiple perspectives including asthma quality of life, asthma control, lung function, exercise capacity, and respiratory muscle strength. Should you feel further refinements would enhance clarity, we would be grateful for any additional guidance. We believe this modified title more accurately represents the synthesized evidence from systematic reviews regarding PA's multidimensional role in asthma care.

**Comment 3:** *What is the targeted population?*

**Response 3:** Thank you for raising this important point. We have refined our inclusion criteria to explicitly state: "Participants diagnosed with asthma, irrespective of age, sex, or severity."(Page4, Line 98-99).

**Comment 4:***(1) P3 L54-58 this sentence is too long to follow. Try breaking down. (2) P3-L58-60 this sentence is incomprehensible (3) P3-L60 “An” overview, not “a”.*

**Response 4:** Thank you for your meticulous review.We have checked and revised this paragraph and it will read as follows：Physical activity, a non-pharmacological intervention, has been widely used to improve quality of life, mitigate asthma symptoms, and enhance exercise capacity, cardiopulmonary fitness, and muscular strength. The World Health Organization characterizes physical activity as "any bodily movement generated by skeletal muscles that necessitates energy expenditure exceeding resting levels." Numerous SRs have investigated the effects of physical activity on asthma. However, their results are not wholly consistent and have not been thoroughly synthesized . Consequently, a synthesis derived from published SRs is crucial to mitigate the shortcomings of individual studies and offer a more thorough comprehension of the impacts of physical activity on asthma.(Page3, Line 64-73).

**Comment 5:** *Method section: (1) Identify the keywords used to search for papers. (2)Any limitation about the language of publication included in the review.*

**Response 5:** Thank you for your suggestion, and we have added a description of the language limitations, search strategy, age of the study population, and the type of original study design that you mentioned, and the revised content is as follows:No restrictions were placed on the origin of the SRs, the date of publication, or the language of publication. The search strategy included relevant words related to asthma and physical activity (including 'physical activity', 'exercise training', 'exercise therapy', 'endurance training', 'resistance training', 'muscle stretching exercises', 'upper limb training', 'Running', 'Jogging', 'Swimming', 'Walking', 'Qigong', 'Yoga', 'Pilates' 'Gymnastics', 'Neuromuscular Electrical Stimulation', 'Physiotherapy') and Systematic Review and was adjusted for each database. Comprehensive search methodologies are outlined in Appendix S1.（Page3-4, Line 85-92）

**Comment 6:** *Method section: (1) Identify age of the samples (2) Identify clearly if the samples were people with current asthma condition or patients at hospital or? (3) Identify clearly study design that were included in the review. (4) Identify clearly measurement of PA whether the study included both device-based and subjective approaches.*

**Response 6:** Thank you for your suggestion, we have added information such as the age of the study population you mentioned, the type of original study design, etc., to the inclusion criteria, and the revised content is as follows:The inclusion criteria encompassed all the subsequent aspects: (1) Participants diagnosed with asthma, irrespective of age, sex, or severity; (2) the intervention encompassed any form of physical activity(defined as any bodily movement generated by skeletal muscles that necessitates energy expenditure exceeding resting levels); (3) studies compared physical activity against placebo, blank control, usual care, conventional drug therapy, or other non-pharmacological therapies; (4) at least one outcome was measured among quality of life (AQLQ), asthma control (ACQ), forced expiratory volume in one second (FEV1), forced vital capacity (FVC), peak expiratory flow (PEF), six-minute walk distance (6MWD), maximal oxygen consumption (VO2max), and maximal inspiratory pressure (PImax); (5) The SRs were founded on randomized controlled trials concerning physical activity for asthma. (P4, Line98-108)

**Comment 7:** *This study used only 2 reviewers. Usually 3 reviewers are used to screen the papers, in case there are some conflicting ideas when trying to classify the papers. Explain strategies in managing the challenges with 2 reviewers.*

**Response 7：**Thank you for raising this important point. We sincerely appreciate your attention to methodological rigor. To clarify, our manuscript explicitly outlines the screening process in the Study Selection section:The eligibility screening was performed independently by two researchers, Wenrui Liu and Shangyue Song. A third author, Zhenzhen Feng, was designated to resolve any disputes concerning screening. (Page 4, Lines 94–97)

**Comment 8:** *Result section:Identify the colours use in figure 3 and what they represented.*

**Response 8:** We sincerely apologize for the confusion caused by the unclear color annotations in the original Figure 3. To enhance clarity and conciseness, we have removed the bubble plot and the GRADE evaluation table. Instead, we now present the results through forest plots (revised Figure 3-7), which clearly display effect sizes, 95% confidence intervals, statistical significance, and GRADE assessment outcomes. Detailed GRADE evaluation procedures are provided in Supplementary File S3 to ensure transparency. These revisions aim to improve the clarity of the study results. We deeply appreciate your constructive feedback and welcome any further suggestions to refine this work.

**Comment 9** *Figure 2 showed that there were so many critically low-quality papers, why were they included in the study?*

**Response 9:** Thank you for your thoughtful observation. We acknowledge that a significant proportion of included studies were rated as "critically low-quality" in the methodological assessment (Figure 2). This reflects a broader challenge in the field of physical activity research, where methodological rigor remains inconsistent—a key finding highlighted in our discussion. Excluding low-quality studies prematurely could have skewed the representation of existing evidence and obscured critical gaps in the literature. Instead, we analyzed these studies’ limitations in the discussion section (Page 18, Lines 335–342) to contextualize their impact on overall conclusions and advocate for improved methodological standards in future research. We appreciate your feedback, as it underscores the importance of addressing quality heterogeneity in evidence synthesis. Please let us know if further clarification is needed.

**Comment 10:** Conclusions: The conclusion didn’t make a good sense of what have been found from the review.

**Response 10:** Thanks for your careful suggestion, we have revised the conclusion section. Physical activity may be an effective complementary therapy for asthma. IMT, yoga, breathing exercises, and aquatic exercises, which show focused enhancement in specific areas. Since most clinical evidence was low quality, there remains an urgent need for rigorous studies to strengthen the evidence base.(P2, L41-44).

# Reviewer #2

**Comment 1:** *This was an interesting review that evaluates the effect of physical activity on quality of life, asthma control, lung function, exercise performance and muscle strength in people with asthma. The manuscript is certainly unique in its own right, however, there are some major concerns worth raising.My primary concern with this review involves the Methods section.The use of additional electronic academic databases would likely have added to the study in both complexity and sample size. If the scope of this study were expanded to use additional databases, more sources might have been identified and explored. For example, the following electronic databases could have been used for a more thorough and inclusive search; “ArticleFirst; Biomed Central; BioOne; BIOSIS; CINAHL; EBSCOHost; JSTOR; ProQuest; SAGE Reference Online; Scopus; google scholar; ScienceDirect; SpringerLink; Taylor & Francis; and Wiley Online.” These databases would have likely added to the overall literature search in their academic rigor, aim, and biomedical scope. While PubMed, Embase, the Cochrane Library, and Web of Science are excellent databases, the use of more databases would have added to the study sample size. Overall, this paper does not merit publication in its current form without a more thorough search of more databases.*

**Response 1:** Thank you for your thoughtful feedback and guidance on strengthening our literature search. To ensure a more comprehensive and interdisciplinary review, we added 8 specialized databases beyond our initial search (PubMed, Embase, Cochrane Library, Web of Science): PEDro (focused on physical therapy and exercise intervention studies), CINAHL (nursing and allied health literature, critical for patient-reported outcomes), Scopus (multidisciplinary coverage, including non-English and grey literature), Sportdiscus (sport science research for exercise prescription insights), Chinese databases (CNKI, Wan Fang, SinoMed, VIP) to capture China-specific evidence. The date of the search was updated to 2025.2.1. In addition, during this supplementary search, we discovered some valuable literature regarding the efficacy of physical activity on childhood asthma. In previous studies, due to limited searches, we found that the quality of articles on childhood asthma was generally low, and considering that we only conducted a descriptive summary, the heterogeneity of childhood asthma was quite high, thus we excluded studies that solely focused on childhood asthma. This search and selection expanded the age range targeted by the review, ultimately adding 17 articles. Among them, there were 9 articles related to adults and mixed populations, and 8 articles specifically concerning childhood asthma. The screening process for supplementary retrieval is shown in Figure 1 (P6), and the list of excluded literature can be found in Appendix S2.

Thank you again for your affirmation and support of our work and manuscript, and thank you for your contribution and dedication to our manuscript. Your affirmation and encouragement are the source of our unremitting progress. As a researcher, it is our common pursuit to produce more researches that are of interest to readers and have clinical value. With your help and guidance, the quality of our manuscripts has been qualitatively improved, and we are looking forward to your further suggestions and criticisms.

# Reviewer #3

**Comment 1:** *Revise Manuscript for Conciseness: Rework the manuscript to focus on critical elements, ensuring it is succinct and directly addresses the research questions and findings. Create a structured abstract with distinct sections (Background, Methods, Results, Conclusions), clearly outlining each component.*

**Response 1:** Thank you for your valuable guidance on improving manuscript clarity. We have restructured the abstract to align with your recommendations, adopting a formalized framework with distinct sections: **Background:** Physical activity (PA) has become a promising complementary non-pharmacological intervention to improve exercise capacity, cardiopulmonary fitness, and quality of life in individuals with asthma. This overview systematically consolidates existing evidence to assess the clinical viability of physical activity as a scalable supplementary therapy for asthma management. **Methods:** We searched 12 electronic databases to identify systematic reviews (SRs) from inception until February 12, 2025, concerning the efficacy of physical activity in asthma management. Literature was independently reviewed, data extracted and verified by two researchers. A third author was designated to mediate any disputes concerning screening. The methodological quality of SRs was assessed using the A Measurement Tool to Assess SRs 2 (AMSTAR 2) checklist, and the certainty of evidence for key outcomes was evaluated using the Grading of Recommendations Assessment, Development and Evaluation (GRADE) framework.

**Results:** This study analyzed 34 SRs (published 2000–2024) that included quantitative synthesis involving 113–2280 participants from asthma populations: adults (9 SRs), children (8 SRs), and mixed adult-child cohorts (17 SRs), with disease severity varying from mild to severe. Ten SRs were assessed as moderate to high quality by AMSTAR 2, whereas the other SRs were classified as low or very low quality. We evaluated the quality of evidence for SRs utilizing the GRADE evidence quality assessment framework. Thirteen moderate-quality evidence, and 51 low or very low-quality evidence support the improvement of PA on the outcomes of asthma quality of life, asthma control, lung function, exercise capacity, and respiratory muscle strength. **Conclusion:** Physical activity may be an effective complementary therapy for asthma. IMT, yoga, breathing exercises, and aquatic exercises, which show focused enhancement in specific areas. Since most clinical evidence was low quality, there remains an urgent need for rigorous studies to strengthen the evidence base.(P1-2, L14-44)

**Comment 2:***While the abstract mentions that six systematic reviews were rated as moderate to high quality, it lacks specific details about the findings or overall effect sizes, which could provide more context and depth to the results. Enhance the details by including specific findings or statistics from the systematic reviews regarding the effectiveness of physical activity on measured outcomes.*

**Response 2** Thank you for your valuable suggestion. To address the need for clearer presentation of findings, we have revised the Results section by forest plots (P11-16, Fig3-7) that explicitly display key information from the included systematic reviews, including:The number of studies analyzed in each SR, sample sizes, Effect sizes (MD/SMD) with 95% confidence intervals and GRADE evidence quality ratings.This revised format aims to provide a more transparent visualization of the pooled effect estimates and methodological rigor while maintaining conciseness. We would be grateful for further guidance if additional statistical details should be highlighted.

**Comment 3:***The abstract briefly touches on limitations concerning study protocols and exclusion criteria but could benefit from a more detailed discussion of how these limitations may affect overall findings and interpretations or influence future research and clinical practice.*

**Response 3:** Thank you for your constructive and valuable comments. We have added a discussion of the impact of research limitations on the evidence, as detailed below. Several constraints must be acknowledged when analyzing the results of this study. First, the methodological quality of the included SRs was predominantly inadequate, which diminished the overall certainty of the evidence. Second, although this review performed subgroup analyses by exercise interventions and offered a descriptive synthesis of age, significant heterogeneity was present among studies concerning asthma severity, exercise modalities, intensity, frequency, and intervention duration. This heterogeneity constrained the capacity to formulate conclusive determinations concerning the ideal exercise protocol (type and dosage) for asthma management.（P18, L332-339）

**Comment 4:** *Consider clarifying Methodology: Although the abstract mentions the use of AMSTAR 2 and GRADE, it could briefly explain how these tools were applied, providing clarity on the assessment process.*

**Response 4：**Thank you for highlighting this need for methodological clarity. We have revised the Methods section to explicitly outline the application of AMSTAR 2 and GRADE tools as follows: 0-1 non-critical item non-conformities are considered to be of high quality, > 1 non-critical item non-conformance is considered medium quality, 1 critical item non-conformance (with or without non-critical item non-conformity) is considered low quality, and > 1 critical item non-conformance is considered to be of critical low quality, regardless of non-critical item non-conformity.(P5, L123-127). The GRADE system was used to evaluate the quality of evidence. RCTs were first set as high quality evidence, and then the outcomes were downgraded by considering five aspects: limitations, inconsistency, indirectness, imprecision, and publication bias. if there is no downgrading, high-quality evidence; One was downgraded to moderate, two to low, and three or more to very low.(P5, L127-131) The full assessment outcomes are visualized in Figure 2 and tabulated in Supplementary File 3. We appreciate your guidance in strengthening the transparency of our quality appraisal process and welcome further suggestions.

**Comment 5:** *Lack of Specific Results: Although the methodology is well-articulated, the manuscript seems weak to present specific results or outcomes from the systematic reviews, which are essential for understanding the effectiveness of physical activity in asthma.*

**Response 5：**Thank you for this constructive feedback. We have revised the Results section to provide specific outcomes as follows: This study analyzed 34 SRs (published 2000–2024) that included quantitative synthesis involving 113–2280 participants from asthma populations: adults (9 SRs), children (8 SRs), and mixed adult-child cohorts (17 SRs), with disease severity varying from mild to severe. Ten SRs were assessed as moderate to high quality by AMSTAR 2, whereas the other SRs were classified as low or very low quality. We evaluated the quality of evidence for SRs utilizing the GRADE evidence quality assessment framework. Thirteen moderate-quality evidence, and 51 low or very low-quality evidence support the improvement of PA on the outcomes of asthma quality of life, asthma control, lung function, exercise capacity, and respiratory muscle strength.(P2, L31-39) Full outcome-specific effect sizes are displayed in Fig3-7. We appreciate your guidance in strengthening the results presentation and welcome further refinements.

**Comment 6:** *recommend concluding with a statement on the practical implications of the findings for healthcare practitioners or policymakers. This would enhance the relevance of the research and may include recommendations for physical activity guidelines for asthma patients.*

**Response 6：**Thanks for your careful suggestion, we have revised the conclusion section. Physical activity may be an effective complementary therapy for asthma. IMT, yoga, breathing exercises, and aquatic exercises, which show focused enhancement in specific areas. Since most clinical evidence was low quality, there remains an urgent need for rigorous studies to strengthen the evidence base.(P2, L41-44).

**Comment 7:** *Highlighting Limitations: Briefly mention the limitations of the current evidence to provide a balanced view of the findings.*

**Response 7:** We sincerely appreciate your suggestion to strengthen the critical appraisal of our findings. We have made modifications to the limitations. The revised content is as follows. Several constraints must be acknowledged when analyzing the results of this study. First, the methodological quality of the included SRs was predominantly inadequate, which diminished the overall certainty of the evidence. Second, although this review performed subgroup analyses by exercise interventions and offered a descriptive synthesis of age, significant heterogeneity was present among studies concerning asthma severity, exercise modalities, intensity, frequency, and intervention duration. This heterogeneity constrained the capacity to formulate conclusive determinations concerning the ideal exercise protocol (type and dosage) for asthma management. (P18, L3312-339)

**Comment 8:** *Future Research Directions: Consider mentioning specific areas where future research is needed or what types of studies would enhance understanding in this field.*

**Response 8:** Thank you for this critical suggestion. We have strengthened the Discussion section by adding the following research priorities in discussion section. The revised content is as follows. To improve the quality of evidence, forthcoming clinical trials and SRs must conform to standardized methodologies and adhere to AMSTAR and GRADE guidelines. Enhancing methodological rigor will augment the reliability of findings, thus informing clinical practice and addressing current evidence deficiencies in asthma management.(P18, L311-314) Since most clinical evidence was low quality, there remains an urgent need for rigorous studies to strengthen the evidence base. Additionally, acknowledging the individual variability among asthma patients, formulating customized exercise prescriptions for each patient would be clinically advantageous.(P19, L340-348)

**Comment 9:** *Language Refinement & Proofreading: Consider thorough proofreading to enhance clarity and address grammatical issues, improving sentence structure for professionalism.*

**Response 9:** We appreciate your valuable feedback regarding the need for enhanced linguistic precision in our manuscript. We acknowledge that the original draft contained grammatical inconsistencies and suboptimal sentence structures that impacted clarity. We have collaborated with a certified professional editing service to conduct rigorous proofreading and substantive revisions during this round of manuscript preparation. The text has been systematically refined to ensure grammatical accuracy, improve readability, and align with academic writing standards. A copy of the language-editing certificate issued by the service provider has been uploaded to the Supplementary Materials section.
