# Supplementary material for: The effectiveness of physical activity in asthma management: An overview of systematic reviews
Source: PLoS One. 2025 Jul 3;20(7):e0325488. doi: 10.1371/journal.pone.0325488 (PMC12225870; doi:10.1371/journal.pone.0325488)
Supplement: Attachment — Submitted filename: Response_to_Reviewers_auresp_2.docx [file pone.0325488.s009.docx]

# Response to Reviewer’s Comments

**Dear Editors and Reviewers**

I would like to extend our heartfelt gratitude to you and the reviewers for their insightful and constructive feedback on our manuscript, "The effectiveness of physical activity in asthma management: An overview of systematic reviews" (Manuscript ID: PONE-D-24-54330R1). This letter is on behalf of all contributing authors. These comments have been indispensable in directing the revision process, thereby significantly improving the clarity and rigour of our work and providing valuable guidance for our ongoing and future research. We have provided comprehensive responses to the comments of each reviewer in the section below. The manuscript has been clearly marked in blue to indicate all revised sections. The reviewers' comments are presented in italicized text in this response letter, while our corresponding responses and modifications are presented in plain text. We have conducted a comprehensive review of the references to ensure their accuracy and completeness in accordance with your editorial requirements. In particular, we added PMID or Web of Science (WOS) numbers to relevant references, removed one inappropriately cited reference, eliminated several duplicates, and added three new references to bolster our arguments—particularly in response to the reviewers' recommendations for enriching the discussion. Please do not hesitate to inform us if we have inadvertently overlooked any comments. Our point-by-point responses are detailed in this document, and all revisions have been integrated into the primary manuscript. We have made every effort to guarantee the transparency and clarity of the revisions, and we hope that the revised manuscript meets the journal's publication standards.

We are grateful for your continued consideration of our work for publication in your journal. Your reply is eagerly anticipated.

Best wishes,

Sincerely,

Zhenzhen Feng

**Contents**

**[Reviewer #2	8](#_Toc16781)**

**[Reviewer #3	10](#_Toc20874)**

# Reviewer #2

**Comment 1:** *Thank you for addressing these comments. the paper is much improved.*

**Response 1:** We sincerely appreciate your thoughtful feedback. We are genuinely appreciative of the time and expertise you dedicated to evaluating our manuscript. The revisions have enhanced the paper's quality, which is encouraging to note. If any further minor adjustments are necessary, we will gladly execute them promptly.

# Reviewer #3

**Comment 1:** *The document presents a comprehensive overview of the effectiveness of physical activity in asthma management, and your request for feedback on the conclusion, methodology, and findings is well noted. Methodology: The methodology is generally well-structured, detailing the search strategy and the criteria for including systematic reviews. However, there are areas for improvement. For instance, while the document mentions the use of AMSTAR 2 and GRADE frameworks for assessing quality, it could provide more clarity on how these assessments influenced the selection of studies.*

**Response 1:** We value your meticulous review and insightful feedback concerning the methodological rigour of our study. We appreciate the opportunity to elucidate this significant matter. Targeted reaction to methodology feedback are as follow: You accurately noted that, although we utilized the AMSTAR 2 and GRADE frameworks to evaluate the methodological quality and certainty of evidence in the included systematic reviews, these evaluations were not applied as exclusion criteria. This methodology was intentional and predetermined in our PROSPERO protocol (CRD42024520761), wherein the inclusion criteria were found on study design attributes (systematic reviews/meta-analyses) and their pertinence to asthma and physical activity, rather than quality benchmarks. Our rationale was twofold: (1) to transparently present the complete range of available evidence, including lower-quality studies—with relevant caveats indicated in Fig 2 and Table S3; and (2) to prevent potential selection bias that could result from excluding studies based on post hoc quality evaluations. To improve clarity, we have incorporated the following sentence in the Methods section: The AMSTAR-2 and GRADE tools were utilized to evaluate methodological quality and the certainty of evidence in the systematic reviews included. However, these instruments were employed exclusively for assessment purposes and did not affect the criteria for study selection (Page5, Line 128-131).Thank you again for your insightful critique that helps strengthen the paper's transparency.

**Comment 2:***Furthermore, the inclusion of a broader range of databases, as suggested by reviewers, could enhance the comprehensiveness of the literature search. Addressing the limitations regarding language and publication bias in more detail would also strengthen the methodology section.*

**Response 2** We express our sincere gratitude to the reviewer for this astute suggestion, which has enhanced the clarity of our methodology. We concur that explicit discourse on potential limitations fortifies the rigour of systematic reviews. Concerning issues of language and publication limitations, we clearly articulated in the Methods section: No restrictions were placed on the origin of the SRs, the date of publication, or the language of publication.(Page4, Line 91-92). We are especially grateful for the opportunity to refine our methodological rigor through this invaluable feedback.

**Comment 3:***The findings are presented clearly, with a good overview of the number of systematic reviews analyzed and the participant demographics. However, the results could be enhanced by including more specific data points, such as effect sizes and confidence intervals, to provide a clearer picture of the impact of physical activity on asthma outcomes.*

**Response 3:** We appreciate your valuable recommendation concerning the presentation of results. Consequently, we have amended the Results section to incorporate forest plots that explicitly present essential information from the systematic reviews included. The visualizations encompass the quantity of studies examined in each systematic review, sample sizes, effect sizes (MD/SMD) with 95% confidence intervals, and GRADE assessments of evidence quality (Pages 12-17, Figures 3-7).

**Comment 4:** *Additionally, discussing the implications of the findings in relation to existing literature would provide context and highlight the significance of the results. Overall, the document is a valuable contribution to the field, but refining these sections could improve clarity and impact.*

**Response 4：**We value your constructive feedback concerning the Discussion section. This section has been augmented to offer a more profound analysis of our findings within the framework of existing literature. Additionally, we have amended the References section to accurately represent the revised citations: The utilization of complementary and alternative medicine in asthma management is receiving worldwide acknowledgment. Physical activity, a scalable and economical adjunctive therapy, has been endorsed in the GINA report [1]. Physical activity encompasses any movement that expends energy, while exercise training denotes a systematic and organized type of physical activity aimed at improving physical fitness[50]. Many SRs have assessed the impact of physical activity on asthma-related outcomes, including quality of life, lung function, exercise capacity, and respiratory muscle strength. However, the results from these reviews are not wholly consistent. Huang F et al. indicated that exercise training markedly enhances the quality of life, exercise endurance, and pulmonary function in asthma patients. Nevertheless, the review omitted unstructured physical activity and failed to examine the varying effects of specific exercise modalities on health outcomes[51]. To our knowledge, this study is the inaugural systematic synthesis of evidence regarding the role of physical activity in asthma management, while concurrently addressing asthma severity, patient age, and specific exercise modalities. Aerobic exercise, yoga, breathing training, and respiratory muscle training have garnered the most focus among the examined exercise modalities[14, 29, 32, 38]. Evidence suggests that diverse forms of physical activity produce unique impacts on different health outcomes. Most SRs employ the AQLQ to evaluate patients' quality of life, and nearly all indicate enhancements subsequent to physical activity interventions, with the exception of those that concentrate solely on respiratory muscle training. Conversely, inspiratory muscle training seems especially efficacious in enhancing objective lung function metrics. SRs investigating breathing exercises and yoga typically do not demonstrate substantial enhancements in FEV1 and FVC, with some even suggesting negative trends. Consequently, to attain holistic enhancements in both subjective and objective results, the formulation of a multifaceted physical activity program is advised. In addition, AQLQ outcomes are derived from self-reported data, whereas lung function measures are based on objective physiological assessments. While enhancements in AQLQ are advantageous, dependence on self-reported outcomes may introduce bias and potentially exaggerate the actual impact of physical activity on health. Consequently, forthcoming research should utilize a blend of subjective and objective outcome measures to augment the reliability and validity of their results. Asthma often results in diminished physical activity levels [52], but limited research has investigated methods to improve adherence to physical activity in asthma patients. Based on this observation, we suggest that subsequent research investigate the impact of various physical activity programs on patient adherence, which may guide the creation of more sustainable and user-friendly exercise regimens. Physical activity has demonstrated beneficial effects on quality of life, pulmonary function, exercise capacity, respiratory muscle strength, and other dimensions. However, a considerable portion of the evidence is of low or very low quality. In light of these concerns, more rigorous, well-structured, large-scale randomized controlled trials are essential to yield superior evidence and to further evaluate the efficacy of physical activity in asthma management. Firstly, original RCTs must disclose randomization techniques, allocation concealment, and blinding, in accordance with CONSORT (Consolidated Standards of Reporting Trials) [53], enabling clinical practitioners to assess the evidence with greater precision. Secondly, the specific frequency, intensity, duration, and type of activity to be undertaken based on asthma severity, subtype, or diverse populations of asthma patients are essential factors that must be established. Thirdly, all SRs must be registered in advance to improve transparency in the process and mitigate the risk of methodological bias. In addition, significant variability is present among studies regarding asthma severity, exercise modality, intensity, frequency, and intervention duration. This variability hinders the capacity to establish conclusive determinations about the ideal type and dosage of physical activity for asthma management. Consequently, further high-quality primary studies that meticulously account for these variables are necessary to formulate definitive, evidence-based recommendations for specific asthma populations (Page19-20, Line 331-384).

**Comment 5:** *The conclusion effectively summarizes the potential of physical activity as a complementary therapy for asthma. However, it could benefit from a more explicit articulation of the implications of the findings. For instance, it might be helpful to emphasize the specific types of physical activities that showed the most promise and how they can be integrated into standard asthma management practices. Additionally, reiterating the need for further research could be strengthened by suggesting specific areas where more rigorous studies are necessary.*

Response 5：Thank you for your perceptive feedback on the Conclusion. We genuinely value the time and effort you dedicated to improving the clarity and effectiveness of our manuscript. In accordance with your recommendations, we have amended the Conclusion section as follows: Engagement in physical activity has been demonstrated to markedly enhance asthma-related outcomes. Specific interventions provide targeted advantages in various domains: aerobic exercise enhances AQLQ scores and lung function metrics, including FEV1, FVC, PEF; breathing exercises improve AQLQ, FVC, and PImax; yoga correlates with enhancements in AQLQ, FEV1, and FVC; aquatic exercise results in increased FEV1; and inspiratory muscle training yields improvements in FEV1, FVC, PEF, and PImax. Nonetheless, there exists an imperative requirement for more stringent studies to fortify the existing evidence base. Furthermore, due to the significant individual variability among asthma patients, the creation of personalized exercise prescriptions is expected to produce enhanced clinical results (Page 2, Line 41-50).
